# Rational Mutational Analysis of a Multidrug MFS Transporter CaMdr1p of *Candida albicans* by Employing a Membrane Environment Based Computational Approach

**DOI:** 10.1371/journal.pcbi.1000624

**Published:** 2009-12-24

**Authors:** Khyati Kapoor, Mohd Rehan, Ajeeta Kaushiki, Ritu Pasrija, Andrew M. Lynn, Rajendra Prasad

**Affiliations:** 1 School of Life Sciences, Jawaharlal Nehru University, New Delhi, India; 2 School of Information Technology, Jawaharlal Nehru University, New Delhi, India; Fox Chase Cancer Center, United States of America

## Abstract

CaMdr1p is a multidrug MFS transporter of pathogenic *Candida albicans*. An over-expression of the gene encoding this protein is linked to clinically encountered azole resistance. In-depth knowledge of the structure and function of CaMdr1p is necessary for an effective design of modulators or inhibitors of this efflux transporter. Towards this goal, in this study, we have employed a membrane environment based computational approach to predict the functionally critical residues of CaMdr1p. For this, information theoretic scores which are variants of Relative Entropy (Modified Relative Entropy RE_M_) were calculated from Multiple Sequence Alignment (MSA) by separately considering distinct physico-chemical properties of transmembrane (TM) and inter-TM regions. The residues of CaMdr1p with high RE_M_ which were predicted to be significantly important were subjected to site-directed mutational analysis. Interestingly, heterologous host *Saccharomyces cerevisiae*, over-expressing these mutant variants of CaMdr1p wherein these high RE_M_ residues were replaced by either alanine or leucine, demonstrated increased susceptibility to tested drugs. The hypersensitivity to drugs was supported by abrogated substrate efflux mediated by mutant variant proteins and was not attributed to their poor expression or surface localization. Additionally, by employing a distance plot from a 3D deduced model of CaMdr1p, we could also predict the role of these functionally critical residues in maintaining apparent inter-helical interactions to provide the desired fold for the proper functioning of CaMdr1p. Residues predicted to be critical for function across the family were also found to be vital from other previously published studies, implying its wider application to other membrane protein families.

## Author Summary

Membrane proteins belonging to the Major Facilitator Superfamily (MFS) transport molecules, including drugs, across the membrane and are known to be associated with drug resistance. CaMdr1p is one such MFS major multidrug efflux pump whose over-expression is linked to frequently encountered azole resistance in hospital isolates of *C. albicans*. Amino acid residues critical for a protein's function are conserved across members of the protein family. However, the traditional measure of conservation is not a useful parameter in mapping a functionally important residue in membrane proteins e.g., hydrophobically conserved stretches form helical transmembrane regions of the protein and are responsible for membrane localization, which individually have limited effect on binding and transport. We have developed a method that uses information theory to score the conservation of a residue relative to its context within the membrane and hypothesize that these residues would be critical for the protein's function. The relevance of predicted residues in the functioning of MFS is validated on CaMdr1p.

## Introduction

In yeasts, including the pathogenic *Candida*, an up-regulation of multidrug transporter genes belonging to either ATP Binding Cassette (ABC) or Major Facilitator Superfamily (MFS) is frequently observed in the cells exposed to the drugs leading to the phenomena of multidrug resistance (MDR) [1]. Among the 28 putative ABC and 95 MFS transporter genes identified in the *C. albicans* genome, only ABC transporters CaCdr1p and CaCdr2p and MFS transporter CaMdr1p, are found to be the major determinants of azole resistance [2],[3]. The reversal of the functionality of these multidrug efflux pump proteins represents an attractive strategy to combat azole resistance.

The major ABC transporters such as CaCdr1p, CaCdr2p bear similar topology and exist as two homologous halves. These, like any other member of the ABC superfamily have four distinct modules: two transmembrane domains (TMDs) each consisting of six transmembrane segments (TMSs) and two nucleotide binding domains (NBDs) located on the cytosolic side of the membrane. Though similar in topology and promiscuity towards substrate specificity, these ABC multidrug transporters of *C. albicans* also display selectivity to the range of substrates they can export [4].

The transporters belonging to MFS, consists of membrane proteins from bacteria to higher eukaryotes and these are involved in symport, antiport or uniport of various substrates [5],[6]. One of the 17 families of MFS transporters uses the proton motive force to drive drug transport and has been identified in both prokaryotes and eukaryotes [7]. Crystal structures of MFS proteins such as lactose permease (LacY), glycerol-3-phosphate (GlpT), EmrD and oxalate: formate antiporter (OxlT), suggest high structural resemblance among this family of proteins [8]. These consist of 12 TMS, arranged with a similar predicted topology, strongly supporting a common structural architecture or fold across all the MFS transporters [9]–[12]. The fungal MFS members particularly those involved in drug transport are poorly explored in terms of their structure and function [13]. The multidrug MFS transporter CaMdr1p belongs to DHA1 family which is widely distributed and includes both drug-specific and multidrug efflux pumps [14].

Random and site-directed mutational strategies have been extensively used to understand the structure and function of these MDR efflux proteins. For example, random mutational analysis of an ABC transporter, ScPdr5p of budding yeast identified several amino acid residues that alter its substrate specificity and sensitivity to various inhibitors [15],[16]. Tutulan-Cunita *et al.* observed that several point mutations led to significant changes in drug specificity of ScPdr5p which are distributed throughout the length of the protein [17]. Site-directed mutagenesis followed by an elegant screen done by Golin's group has revealed interactions between TMS 2 and the NBD which may help to define at least part of the translocation pathway for coupling ATP hydrolysis to drug transport mediated by ScPdr5p. Recently, Schmitt *et al.* have elucidated the role of H1068 in H-loop of ScPdr5p which couples ATP hydrolysis with drug transport [18].

Site-directed mutational analysis of multidrug ABC multidrug transporter CaCdr1p (a close homologue of ScPdr5p) has revealed insight into its drug binding and efflux properties. These studies have implicated some of the amino acid residues of TMS 5, 6, 11 and 12 as the components of the substrate binding pocket(s) of CaCdr1p [19],[20]. Together, these studies suggest that the drug binding sites in CaCdr1p are scattered throughout the protein and probably more than one residue of different helices are involved in binding and extrusion of drugs. However, there is still insufficient information available to predict where and how exactly the most common antifungals such as azoles bind and how are they extruded by CaCdr1p.

Site-directed mutational strategies rely on conservation of residues in a Multiple Sequence Alignment (MSA). The conservation of a residue is calculated from the amino acid frequency distribution in the corresponding column of a MSA. However, the physicochemical conservation is not necessarily responsible for a protein's structure and function but could reflect a more general function such as membrane localization. Thus conservation alone is not sufficient to distinguish between residues responsible for the protein function and membrane localization. Membrane proteins differ from soluble proteins because of their inter-TM hydrophilic and TM hydrophobic propensities, which have allowed the development of efficient membrane protein TM prediction methods [21] and of membrane protein specific substitution matrices [22].

The quantification of residue conservation has evolved over the last few years to the use of information theoretic measures [23]. Relative entropy is a distance measure commonly applied to multiple alignments by comparing the observed frequency distribution with a background distribution. In the present study, we have developed and employed a new method using information theory to rationalize mutation strategies and also applied it to a MFS multidrug transporter CaMdr1p [24]. Relative Entropy (RE) or the Kullback-Liebler divergence is an information theoretic measure of the difference between two probability distributions and has been increasingly applied in bioinformatics to identify functional residues [24],[25]. The use of RE with background frequencies [26] can improve the prediction of a protein's functional residues [27]–[32] as well as detect residues that determine the functional subtype of proteins [28]. Though the basic Kullback-Liebler equation has not changed, its intelligent application in our method calculates Relative Entropy (RE_M_) relative to its context within the membrane. The RE_M_ scoring scheme has been improved by treating TM and inter-TM regions of MFS proteins separately which has drastically increased the credibility over the existing methods [23]. In this manuscript, we have compared traditional treatment of conservation, and standard RE, with our improved method. We validated our predictions by replacing the predicted highest RE_M_ positions of CaMdr1p with alanine by site-directed mutagenesis. We show that most of these residues when replaced with alanine showed decreased resistance to drugs which was corroborated by abrogated efflux of drugs. Additionally, we could further confirm the functional relevance of each of these high RE_M_ residues by predicting their location in deduced 3D model of CaMdr1p and their role in maintaining apparent inter-helical interactions. With this approach, our method enabled us to accurately predict MFS-wide function-specific residues, validated by using CaMdr1p.

## Results

### RE_M_ considers conservation as well as the background probability of each alignment position of a MSA

**Figure 1:**
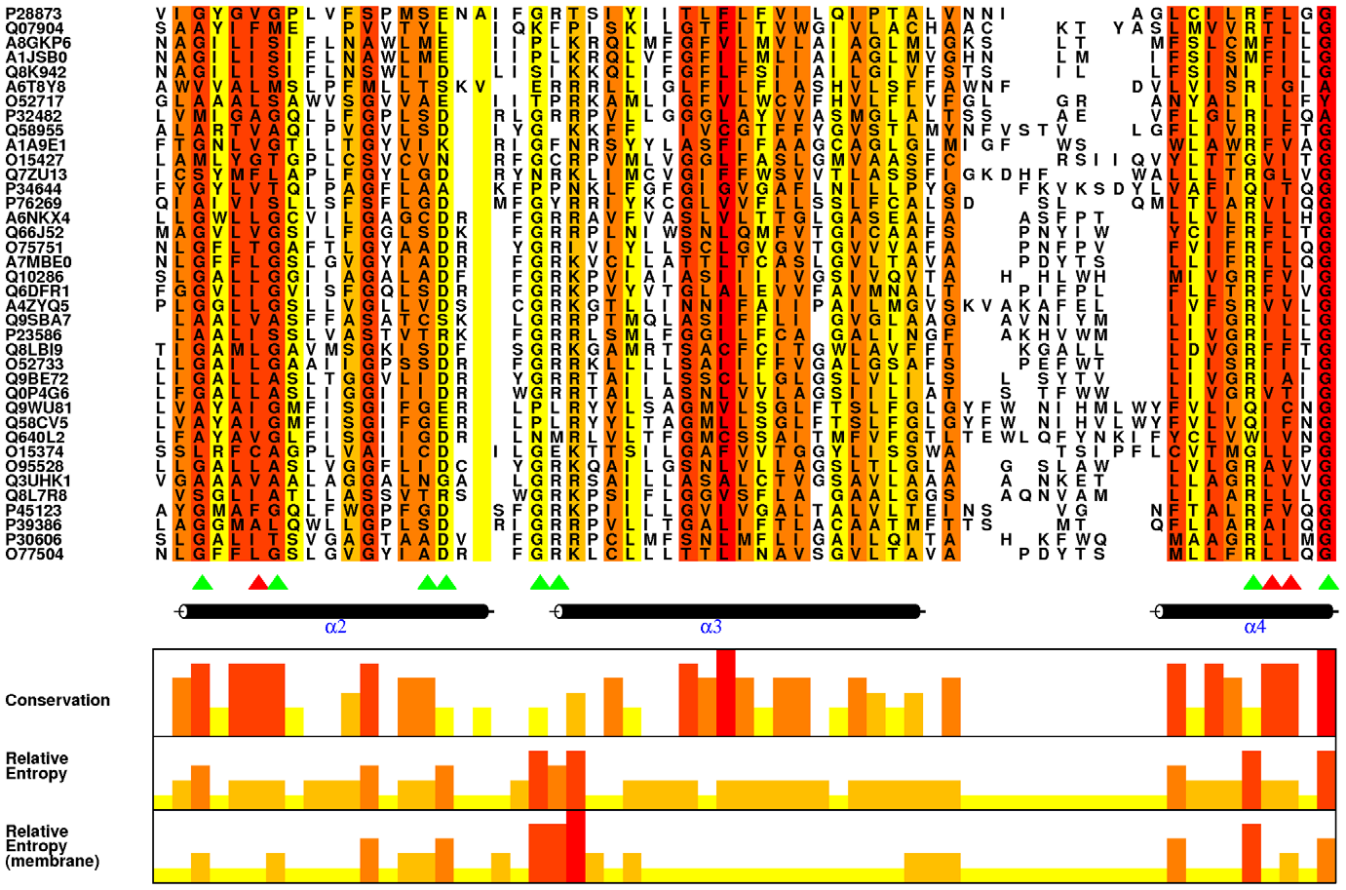
A portion of Multiple Alignment showing conservation and RE_M_ for each column. Figure showing a representative portion of the alignment of MFS sequences and is generated using Alscript [48]. The alignment is coloured in a gradient from red to yellow on the basis of decreasing conservation score. Conservation was calculated using a method by Livingstone *et al.* [49] and the scores are shown as a histogram. The histogram compares the conservation, RE and the RE_M_ scores for the alignment columns shows in the figure. Results of selected mutations in CaMdr1p are indicated by triangles, green being sensitive while red are resistant. RE_M_ scores are better indicators of functional relevance than physicochemical conservation.

**Figure 2:**
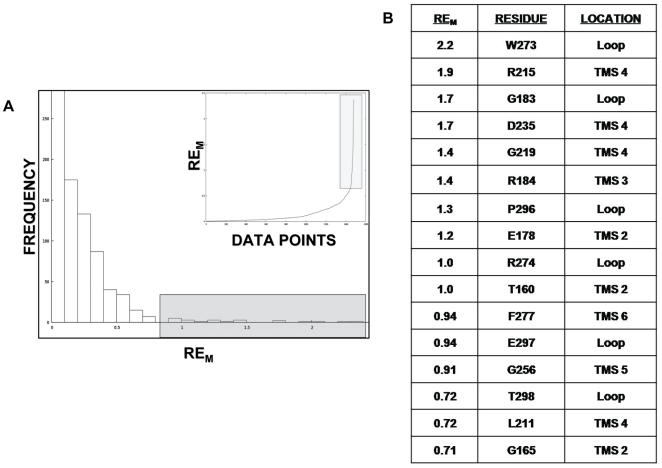
Distribution curve of RE_M_ values of complete MSA. Panel A: Histogram of the RE_M_ scores (+ve/background frequency) vs frequency for all positions of MSA of the MFS transporters. The top 30 RE_M_ positions depicted in the boxed region were selected for further analysis. The inset represents the same data by a line graph. B: The table shows 16 out of the top 30 high RE_M_ alignment positions where the residue in CaMdr1p matched with the most frequent amino acid at that particular position in a MSA of 342 MFS members. Their predicted location with respect to CaMdr1p is also displayed in the next column.

A comprehensive non-redundant data set, sourced from all MFS sequences present in the 56.2 release, was generated. This data set was then aligned using a membrane-specific multiple alignment program, which stacked the helices appropriately. A highly conserved residue in a multiple alignment is predicted to have a functional significance. We calculated conservation values using the algorithm from Jalview. Residues shown to be conserved dominate the TM helices, and on closer evaluation are largely hydrophobic residues associated with membrane localization. The traditional relative entropy and our modified treatment of the method (RE_M_) were calculated on the same alignment using scripts written in-house. (Fig. 1 shows a representative section of the alignment along with the RE_M_, RE and conservation scores; see supplementary Table S1 for the RE_M_, RE and conservation scores for the entire MSA). The distribution curve generated on the basis of RE_M_ from the MSA is shown in Fig. 2A. Notably the dominant signal using a conventional conservation measure lies in the TM helices and traditional RE also issues high scores to these residues which are less frequent in nature. Our treatment using a RE_M_ dampens the membrane localization signals further increasing the signal from atypically occurring conserved residues (Figure S1). Since RE_M_ considers conservation as well as the background probability of a residue at a particular alignment position, it is an improved index of the functional significance of a residue. To emphasize this fact, thirty residues with highest values were short-listed each from the conservation list, RE list and RE_M_ list. On comparison, it was found that there are thirteen residues shortlisted as both conserved and with high RE while it was found that only nine out of these short-listed columns are both conserved and have high RE_M_ (Supplementary Table S2). The thirty alignment positions with high RE_M_ were further studied to assess their functional relevance. Expectedly, all residues predicted using RE_M_, are not present in every protein in the family. In CaMdr1p, 16 residues were identical with the most frequently occurring residue in the thirty highest scored alignment columns, and were mutated to alanine to directly validate the prediction (Fig. 2B).

### Residues with high RE_M_ are part of the known motifs of Major Facilitator Superfamily

These sixteen out of the top thirty positions, wherein the residue in CaMdr1p matched with the most occurring residue across that alignment position in MSA were analyzed for further studies by site-directed mutagenesis. Interestingly, most of the sixteen residues with high RE_M_ turned out to be part of the well-known motifs of the MFS. These motifs are identified as Motif A (GxLaDrxGrkxxl), Motif B (lxxxRxxqGxgaa) which are conserved throughout the MFS, Motif C (gxxxGPxxGGxl) only in 12 and 14-TMS family and Motif D2 exclusive to 12-TMS family [7]. Three out of the sixteen residues short-listed for CaMdr1p; E178, G183 and R184 are a part of Motif A; residues L211, R215 and G219 are a part of Motif B and G256 is a part of motif C. In addition to the known motifs mentioned above, two new motifs have been identified by our study. The residues in these stretches ^273^Wrxxf^277^ and ^296^Pespr^300^ have high RE_M_ scores. However, the known motif D2 does not appear to be highly conserved in our alignment and is thus not predicted to be family-wide function-specific.

### Site-specific mutagenesis of residues with high RE_M_ shows that they are functionally critical

**Figure 3:**
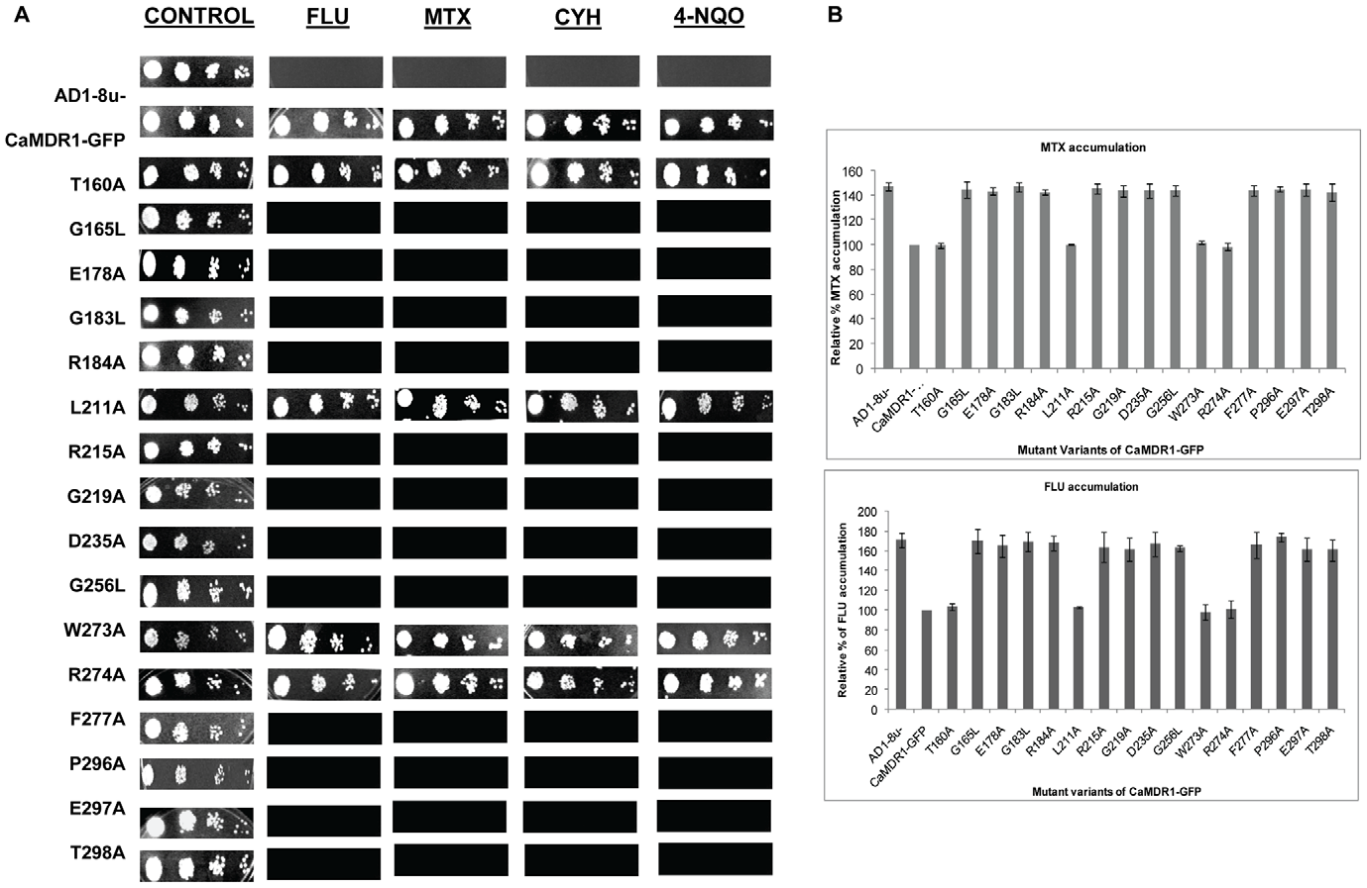
Drug susceptibility and transport assays of mutant variants of CaMDR1-GFP in *S. cerevisiae*. Panel A: Drug resistance profile of wild type and mutant CaMDR1-GFP yeast strains determined by spot assay. For spot assay, cells were freshly streaked, grown overnight and then resuspended in normal saline to an A_600_ of 0.1 (1×10^6^ cells) and 5 µl of five-fold serial dilutions, namely 1 (1∶5), 2 (1∶25), 3 (1∶125) and 4 (1∶625), of each strain was spotted on to YEPD plates in the absence (control) and presence of the following drugs: FLU (0.20 µg/ml), CYH (0.20 µg/ml), 4-NQO (0.20 µg/ml) and MTX (65 µg/ml). Growth differences were recorded following incubation of the plates for 48 hrs at 30°C. Growth was not affected by the presence of the solvents used for the drugs (data not shown). B: [^3^H] MTX and [^3^H] FLU accumulation in the different mutant variants of CaMdr1p-GFP. Controls AD1-8u^−^ and RPCaMDR1-GFP have also been included for comparison. The results are means±standard deviations for three independent experiments.

All the sixteen residues selected on the basis of high RE_M_ were mutated by employing site-directed mutagenesis and were replaced with alanine except G165, G183 and G256 which were replaced by leucine. For functional analysis of the mutant variants, a heterologous hyper-expression system, where GFP-tagged CaMdr1p (CaMDR1-GFP) was stably over-expressed from a genomic *PDR5* locus in a *S. cerevisiae* mutant AD1-8u^−^, was used [33]. The host AD1-8u^−^ developed by Goffeau's group, was derived from a *Pdr1-3* mutant strain with a gain-of-function mutation in the transcription factor Pdr1p, resulting in constitutive hyper-induction of the *PDR5* promoter [34]. A single-copy integration of each transformant at the *PDR5* locus was confirmed by Southern hybridization (data not shown). Two positive clones of each mutant were selected to rule out clonal variations. These residues with high RE_M_ score showed increased drug susceptibility and abrogated efflux of substrates such as [^3^H] MTX and [^3^H] FLU (Fig. 3). Of note, there were few exceptions to the list of residues with high RE_M_. For example residues T160, L211, W273 and R274 have high RE_M_ values but do not appear to be critical for function of CaMdr1p since the drug susceptibility and efflux are not affected upon replacement of these residues with alanine.

### All the mutant variants of CaMdr1p are properly surface localized

**Figure 4:**
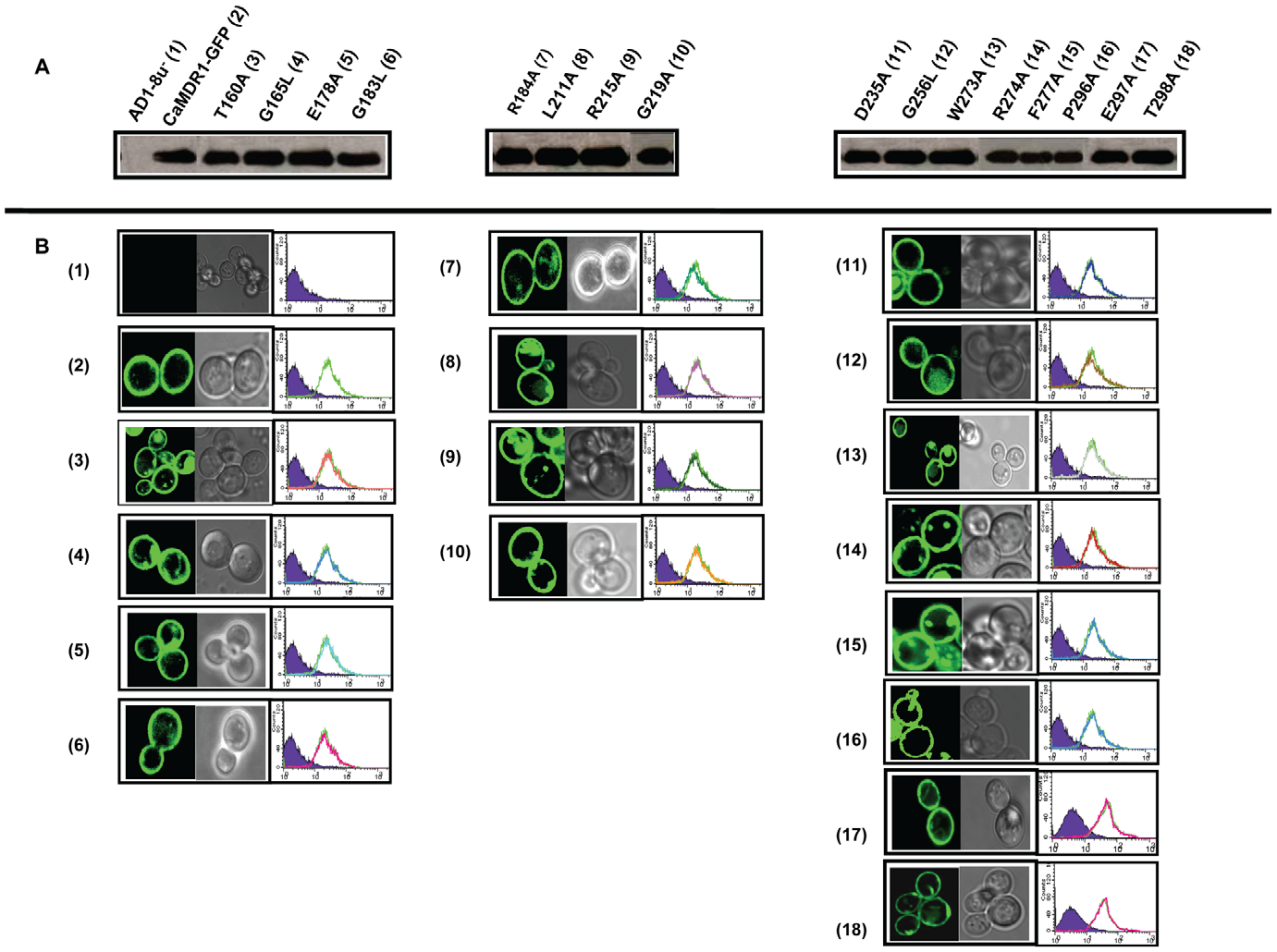
Protein expression profiles of CaMdr1p-GFP and its mutant variants in *S. cerevisiae*. Panel A: Western Blot analysis of the PM fraction of mutant variants with anti-GFP antibody. B: Confocal and FACS analysis of the all the mutant variants to check their expression and localization in comparison with AD1-8u^−^ (negative control) and RPCaMDR1-GFP (positive control) [46].

To confirm that the change in susceptibility observed in the mutant variants was not due to their poor expression or mislocalization, we compared the localization of GFP-tagged version of CaMdr1p (CaMDR1-GFP) and its mutant variants by FACS and confocal imaging. A proper localization of all the mutant variants was confirmed by both these methods which showed proper rimmed appearance of GFP-tagged CaMdr1p. The Western Blot analysis further confirmed that the expression levels of CaMdr1p of all the mutant variants were similar thus corroborating FACS and confocal data (Fig. 4).

### Distance plot of CaMdr1p from deduced 3D homology model reveals inter-helical interactions

**Figure 5:**
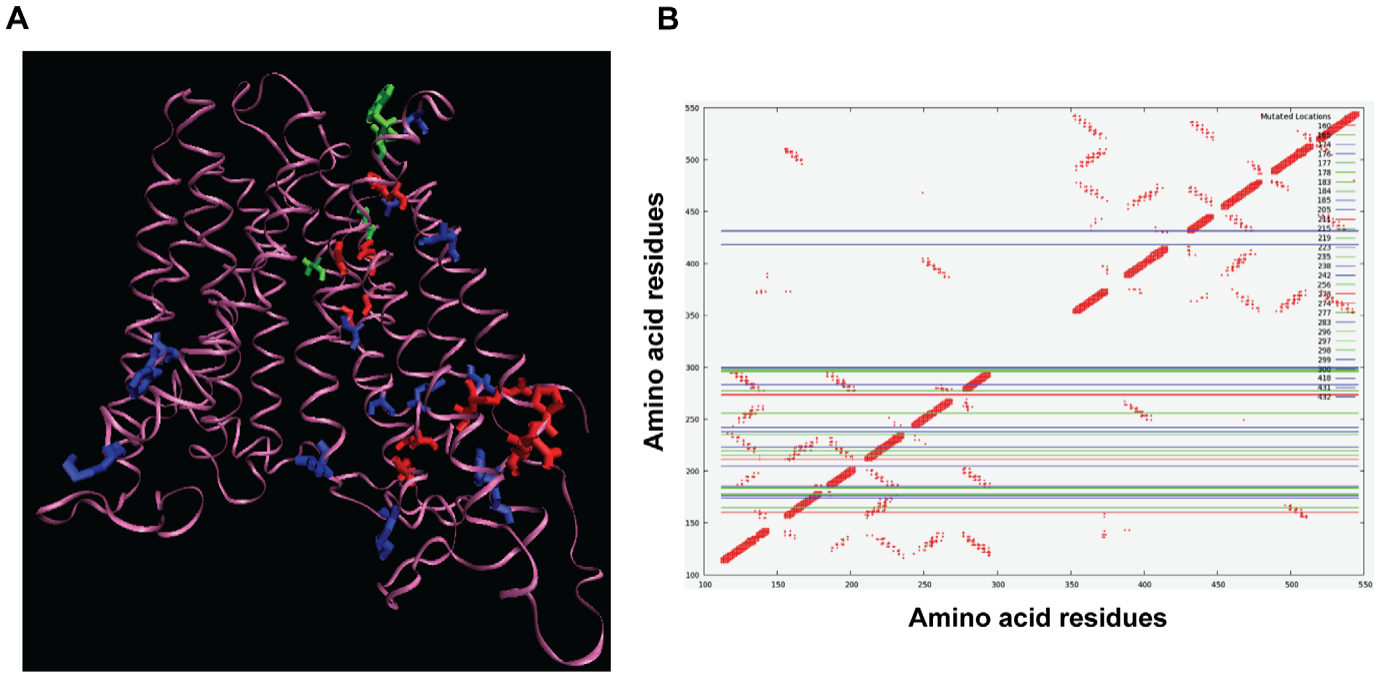
The 3D homology model of CaMdr1p and the contact map derived from it. Panel A: The 3D homology model of CaMdr1p wherein the mutated residues are marked onto the model and are coloured on the basis of the phenotypes exhibited upon mutation. Red denotes sensitive, green shows resistant and blue marks a position predicted to be important but not mutated as the CaMdr1p residues did not match the conserved residue in that alignment position. The structure is viewed using Visual Molecular Dynamic (VMD) software. B: The contact map of CaMdr1p is plotted between all the residues vs all the residues and displays the interactions between the beta carbon of each residue and beta carbon atoms of every other residue within and up to 8 A° of distance (Cα is used for glycine). Each cross points to an interaction between a residue on x-axis and a residue on y-axis. The lines represent the top thirty high RE_M_ residues and are coloured on the basis of the phenotypes where green represents the residues that are sensitive upon mutation while red are the ones that do not show any phenotype. The blue lines mark the residues in CaMdr1p that did not match with the most frequent residue in that particular column of the MSA.

For evaluating the relevance of high RE_M_ residues in CaMdr1p, a 3D homology model was constructed using available crystal structures of lac permease of *E. coli* (1pv6), glycerol-3-phophate of *E. coli* (1pw4) and oxalate: formate transporter of *O. formigenes* (1zc7) as described in Materials and Methods. All the top thirty positions of highest RE_M_ are marked in the model which mostly lie in the N-terminal half of the protein (Fig. 5A). Using the homology model, a symmetric contact map of CaMdr1p was generated as discussed in Materials and Methods (Fig. 5B). We exploited this distance plot to ascertain the role of these residues with high RE_M_.

**Figure 6:**
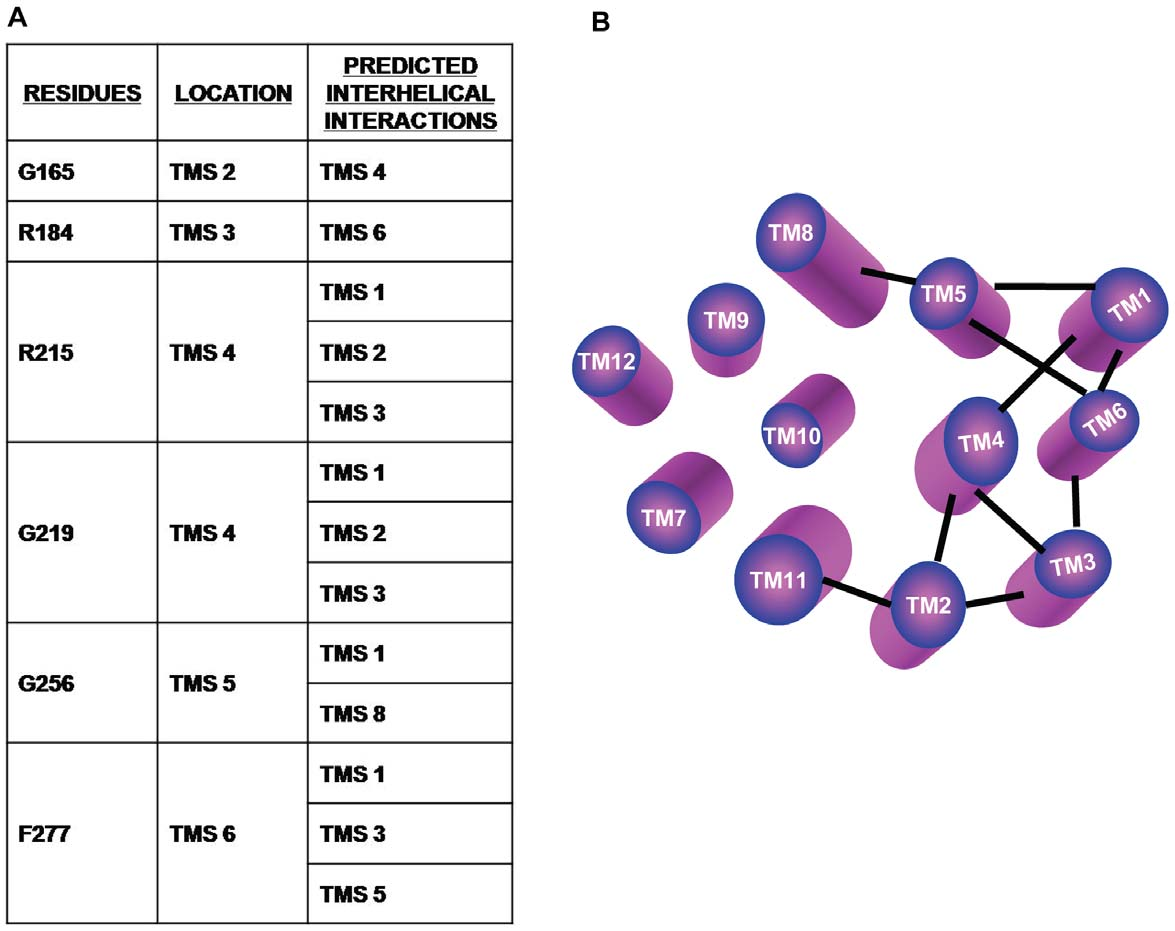
Summary of inter-helical interactions via high RE_M_ residues. Panel A: The table summarizes predicted inter-helical interactions mediated via selected residues with high RE_M_. More than one residue pair is predicted to be involved in maintaining the interactions between the helices. B: Pictorial representation of inter-helical interactions via these high RE_M_ residues. Figure shows that the residues involved in these interactions are majorly confined to the N-terminal half of the protein.

It is apparent that residues L211, R215 and G219 in TMS 4 are within 8 A° distance to many residues of TMS 1, 2 and 3. For example, it can be seen that residue G219 on TMS 4 lie on the same face of the helix and is within 8 A° to the residues G165 and G169 on TMS 2. Indeed, the mutation of predicted G165 and G169 on TMS 2 resulted in abrogated drug susceptibility and transport (data not shown). All the predicted interactions are summarized in Fig. 6A and shown in a pictorial representation of the homology model in Fig. 6B.

## Discussion

The multidrug MFS transporter CaMdr1p harbors a conserved antiporter ‘motif C’ within TMS 5. Our recent study has revealed that the conserved and critical residues of this motif and of TMS 5 are bunched together on the same face of its helical wheel projection and are critical in drug efflux [35]. However, the structure and function aspects of this major multidrug transporter remain poorly understood. To address some of these questions, in this study, we have rationalized conventional mutational strategy and applied computational approach to predict functionally critical residues of CaMdr1p.

The sequence set described in this manuscript represents a comprehensive non-redundant coverage of annotated MFS sequences from SWISSPROT. Many methods have been developed to improve the MSA of membrane protein families for accurate predictions of residues critical for structure and function [36]. Membrane proteins have fold signals which are easily mapped to the primary sequence as TM and inter-TM stretches. Considering the differences in physico-chemical properties of these two regions, membrane protein specific substitution matrices have been developed [22]. However, we argued that a conservation score on the basis of identity or physico-chemical similarity still remains inadequate as the background frequencies of their immediate environmental milieu are radically different with respect to hydrophilic and hydrophobic propensities. This is also apparent from the conservation scores of the MSA wherein a large proportion of the conserved columns correspond to hydrophobic TM regions. Notably, two CaMdr1p residues (F216 and L217) with high conservation but low RE_M_ were taken as controls, when replaced with alanine showed no change in the phenotype (data not shown). One of the most basic fold specific signals is the hydrophobic core in globular proteins, and the TM region in membrane proteins. Unlike globular proteins, the hydrophobic TM region is continuous in the membrane protein's primary structure, and indeed this still remains one of the preferred methods to identify membrane proteins, and map their TM regions. While it is intuitive that the synchronous stretch of hydrophobic residues is responsible for membrane localization, the application of a scoring method that can distinguish these residues from family-wide alignment columns associated with other functions has not yet been deployed. In essence, we require a method that can objectively separate the TM signals from other signals. To overcome these limitations, we improved existing method(s) of information theory wherein RE_M_ was calculated on the basis of MSA of MFS proteins, keeping in mind the differences in the environmental milieus. We thus treated TM and inter-TM regions by different background probabilities for calculation of RE_M_. These RE_M_ scores helped us to predict those sites which have amino acid distributions very different from the respective background distribution thereby statistically predicted to be functionally critical. Not all the residues predicted using RE_M_, are present in every protein in the family. In CaMdr1p, 16 residues were identical with the most frequently occurring residue in the thirty highest scored alignment columns, and were mutated to directly validate the prediction (Fig. 2B). Our results of drug susceptibility assays revealed that almost all of these matched residues with high RE_M_ when replaced with alanine displayed sensitivity to the tested drugs and showed abrogated drug transport (Fig. 3). Interestingly, when we mutated residues which had high conservation values but lower RE_M_ values (negative control); none showed alterations in drug susceptibilities and thus did not retain the functionally critical stringency as was evident from residues with higher RE_M_. For example, analysis of a few conserved columns of the MSA, such as F216, L217 and L171 having RE_M_ values between 0.57-0.44 revealed that their replacement with alanine did not affect the function of CaMdr1p (data not shown). This strengthens the fact that our method takes into account the conservation along with the background frequency and thus lists out residues which affect the function. Also, to check the efficiency of the method, another negative control used was to mutate residues which are having low conservation and low RE_M_ values but lie in the vicinity of one of the 16 selected high RE_M_ residues. For example, when C225 which is closer to the critical G219 and D235, was mutated to C225A, the functioning of the protein was not affected (data not shown). Similarly, for critical G256, when residues A248, A253 and V254 which are within its vicinity were mutated as A248G, A253G and V254A, the mutant variants continued to behave as WT-CaMDR1-GFP [35].

To further elucidate the role of predicted residues in the functionality of CaMdr1p, a homology model based on the available crystal structures of lac permease, glycerol-3-phophate and oxalate: formate transporter was deduced [9]-[11]. The RE_M_ method predicts the relative importance of a residue purely from sequence analysis and is independent of the protein's structure. However, the role a residue plays in the protein's function is not readily apparent from its sequence. We exploited the protein's 3D model as a guide to reason why a residue is functionally critical. The deduced 3D model suggested that similar to other MFS structures, the 12 TM helices of the CaMdr1p span the membrane in such a way that they form the channel pore particularly aligned by residues of TMS 2, 4, 5, 7, 8, 10 and 11. From the deduced homology model of CaMdr1p, a symmetric contact map was generated to highlight the inter-helical interactions of the protein (Fig. 5B). Based on the predictions from the distance map, we could show that many high RE_M_ residues are indeed a part of inter-helical interactions (Fig. 6B). It is apparent that more than one residue pair is predicted to be involved in maintaining the interactions between helices (Fig. 6A).

Our aim in developing this method was to identify residues with high specificity which would play a critical role across this entire MFS protein family. Although signals associated with antiporter motifs have been identified using this method, a finer granularity in function such as substrate specificity determining residues is not expected, as these signals would not be family-wide. Since the enlisted residues with high RE_M_ values which are functionally critical for CaMdr1p are expected to be family-wide function-specific and thus critical for the entire MFS protein data set, we validated their relevance from the earlier published work. It is known that Motif A of the MFS transporters span an eight residue long loop between TMS 2 and 3 and is suggested to be involved in maintaining a β-turn linking the adjacent TM helices [14]. In the present study, G183 and R184 in the loop between TMS 2 and TMS 3 of CaMdr1p were picked up as family-wide function-specific residues thus corroborating that these residues are a part of Motif A (GxLaDrxGrkxxl) which holds importance throughout the MFS transporters. The hypothesized rocking motion in MFS presumably requires conformational changes in the TMS and the β–turns. In this, the transporter inter-converts between C_i_ (inward facing) and C_o_ (outward facing) states for translocation of substrates. In glycerol-3-phosphate of *E. coli*, it was seen that D88 was involved in inter-conversion between these C_i_ and C_o_ states of the protein [10]. Interestingly, D88 corresponds to E178 of CaMdr1p which also lies in Motif A which upon mutation to alanine turns out to be critical for drug susceptibility and efflux (Table 1).

**Table 1:** High RE_M_ alignment positions shown to be critical for function in other MFS members.

MFS Transporter	Organism	Critical Residue	Location	Function	References
LacY	*E. coli*	G111 (1.9)	TM4	Residue at kink	[9]
LacY	*E. coli*	G150 (0.91)	TM5	Residue at kink	-do-
PcaK	*Pseudomonas putida*	R124 (1.9)	TM4	Helix Packing	[37]
PcaK	*Pseudomonas putida*	E144 (0.76)	TM4	Helix Packing	-do-
GlpT	*E. coli*	D88 (1.27)	TM2	Involved in the C_i_ and C_o_ inter-conversion	[10]
MdfA	*E. coli*	D77 (1.2)	TM2	No activity seen if mutated to alanine	[50]
EmrD	*E. coli*	R118 (0.59)	TM4	Role in defining topology	[14]

The residues with high RE_M_ positions and their predicted roles in the case of other MFS members are enlisted. The residues of CaMdr1p at the same position in the alignment are shown to be critical in this study.

Motif B (lxxxRxxqGxgaa) of all MFS has a role in energy coupling which spans the N-terminal half of TMS 4 [7]. CaMdr1p contact map reveals that residues in Motif B interface with residues ^165^GxxxG^169^ on TMS 2. Motifs rich in glycine and proline residues promote formation of special backbone conformation including kinks in TMS, tight interactions between TMS and very flexible β-turns. In human VAchT, Motif B and the adjacent sequences contain a total of nine notch signatures. A notch allows two helical TMS to approach each other unusually closely because small side chains are located at the interface. R124 of PcaK of *Pseudomonas putida* which is equivalent to high RE_M_ R215 of CaMdr1p of *C. albicans* is shown to be critical for helix packing [37]. Interestingly, G111 of LacY of *E.coli* which also occupies a position in the same alignment column is also critical and earlier shown to be a residue at a kink.

Residues from Motif C (gxxxGPxxGGxl) which is exclusive to 12-TMS family are also picked up by our calculations [7]. G150 of LacY of *E.coli* which is equivalent to high RE_M_ G256 of CaMdr1p is function-specific for LacY protein [38]. A stretch of conserved residues ^296^Pespr^300^, previously unidentified, at the end of TMS 6 were also predicted with high RE_M_. We have mutated equivalent residues P296A, E297A and T298A of CaMdr1p that overlap with the consensus residues in the stretch and found that cells expressing these mutated variants displayed increased sensitivity to drugs. However, the functional significance of these residues is yet to be established.

There are a few exceptions which emerged from our method. For example, our method did not pick up any residue of Motif D2. This could be an artifact of the method used for alignments in earlier studies. In this study we have employed a membrane protein specific alignment method whereas earlier reports have used standard multiple alignments substitution matrices with smaller data sets. However, when we repeated the alignment using MUSCLE [39] and with the standard substitution matrix (Blosum 62) [40] on the complete data set the motif still did not appear (data not shown). Motif D2 is assumed to have a structural significance as it holds a major kink within TMS 1 but mutations in this motif do not alter the backbone conformation. As an example of the possibly insignificant role of the motif, in human VAchT, the mutation of L49G in this motif completely eliminates propensity for a kink or notch and abolishes activity while normally a glycine itself is expected to be present at this position and is supposed to be involved in maintaining a major kink in this motif [41].

Out of the 16 residues that were mutated, T160A, L211A, W273A and R274A did not lead to any phenotypic changes. It is known that for some of the positions in alignment, the most frequent amino acid does not match with the residue of CaMdr1p at that site. One reason for this could be that some of the functionally important residues co-evolve i.e., these residues may mutate, with compensatory mutation occurring elsewhere in the protein to regain function [42]. T160 where the most frequent residue is a serine at that position may be one such case. Another reason may be that the alignment used in this study involved prediction of TMS with the possibility of errors in demarcating the edges of TM helices. Residues from columns lining the edges of the helices may be wrongly assigned to TM and inter-TM regions. This probably explains the lack of any effect of mutation on residues T160 of TMS 2 and L211 of TMS 4 which lie at the edge of the respective TMS. Other exceptions to our predictions are the mutation of W273 and R274 which though highly conserved and probably a part of the new motif but do not abrogate function upon mutation. Although a few tryptophans in an ABC transporter MRP1, have been shown to be involved in substrate binding and transport [43], generally, in a membrane protein tryptophan residues located on the surface of the molecule are mainly positioned to form hydrogen bonds with the lipid head groups while their hydrophobic rings are immersed in the lipid part of the bilayer [44]. We predict that W273 and R274 may be associated with membrane helix orientation and this function may not be perturbed by mutating them individually through alanine scanning. Alternatively, the tryptophan-arginine residues could be functionally critical in tandem and compensate each other for the loss of either one of them.

Of note, in our predictions, substrate specific residues with high RE_M_ are not picked up which predominantly occur in C-terminal of MFS proteins. It should be mentioned that since our alignment considers the entire MFS, residues responsible for substrate specificity would only be selectively conserved within a subfamily and would not have sufficiently strong signals to be visible in this present family-wide study. For this, the same method may be applied to a data set classified on the basis of substrate selectivity to identify residues critical to the functioning of that subfamily.

There are a number of conservation methods known but none has yet achieved both biological and statistical rigor. We have used RE_M_ to separate conserved residues from the background function of TM localization. The interpretations support the well-known fact that MFS has a conserved N-terminal half which has residues important for maintenance of a specific fold for this class of proteins while C-terminal half has a more specific role in substrate binding and recognition [7]. Taken together, our study provides an insight into the molecular details of MFS transporters in general and CaMdr1p in particular. Our method of scaled RE_M_ calculations improves its performance over other information theoretic methods. Additionally, this study also provides a method for rational mutational analysis not only for MFS proteins but can be applied to any class of membrane proteins and thus makes it possible to predict and locate family-wide functionally relevant residues.

## Materials and Methods

### Materials

Anti-GFP monoclonal antibody was purchased from BD Biosciences Clontech, Palo Alto, CA, USA. DNA modifying enzymes were purchased from NEB. The drugs cycloheximide (CYH), 4-Nitroquinoline oxide (4-NQO), Methotrexate (MTX) and Protease inhibitors (Phenylmethylsulfonyl fluoride, Leupeptin, Aprotinin, Pepstatin A, TPCK, TLCK) and other molecular grade chemicals were obtained from Sigma Chemicals Co. (St. Louis, MO, USA) Fluconazole (FLU) was generously provided by Ranbaxy Laboratories, India. [^3^H] Fluconazole was custom prepared and [^3^H] Methotrexate (MTX) was purchased from Amersham Biosciences, United Kingdom.

### Media and strains

Plasmids were maintained in *Escherichia coli* DH5α. *E.coli* was cultured in Luria-Bertani medium (Difco, BD Biosciences, NJ, USA) to which ampicillin was added (100 µg/ml). The *S. cerevisiae* strain used was AD1-8u^−^ (*MAT*a *pdr1-3 his1 ura3* Δ*yor1*::*hisG* Δ*snq2*::*hisG* Δ*pdr5*::*hisG* Δ*pdr10*::*hisG* Δ*pdr11*::*hisG* Δ*ycf1*::*hisG* Δ*pdr3*::*hisG* Δ*pdr15*::*hisG*), provided by Richard D. Cannon, University of Otago, Dunedin, New Zealand [33], [34]. The yeast strains used in this study are listed in the Supplementary Table S3. The yeast strains were cultured in YEPD broth (Bio101, Vista, CA, USA) or in SD-ura^−^ dropout media (0.67% yeast nitrogen base, 0.2% dropout mix, and 2% glucose; Difco). For agar plates, 2.5% (w/v) Bacto agar (Difco, NJ, USA) was added to the medium.

### Methods

#### Multiple sequence alignment

561 MFS sequences having 12 TMS as predicted by TMHMM [21] were extracted from SWISSPROT release 56.2. Redundancy in the sequences is reduced to 90% on the basis of identity using BLASTclust by setting the identity threshold S to 90 and keeping all other parameters to default values (http://genomes.ucsd.edu/manuals/blast/blastclust.html). The resulting 342 sequences were then aligned by PRALINETM using TMHMM as the method for predicting TM lengths and keeping all other parameters at their default values [36],[45] (See Supplementary Dataset S1).

#### Calculation of conservation score, RE and RE_M_

Conservation of an amino acid in an alignment column was calculated using Jalview using available scoring schemes which are restricted to soluble proteins, was modified to accommodate different background probabilities for TM and inter-TM regions.

RE is calculated as the deviation of the amino acid distribution *P_i_(a)* from a background disribution *f(a*).
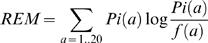
where ***P_i_(a)*** is the probability of the occurrence of amino acid ***a*** in column ***i*** of the MSA ***f(a)*** is the background probability of amino acid ***a*** and is classically estimated from SWISSPROT as the probability of occurrence of an amino acid in a large data set of proteins.

In our method RE_M_, ***f(a)*** is the background probability of amino acid ***a*** and is classically estimated as the probability of occurrence of an amino acid in a large data set of proteins. ***f(a)*** = ***f(a)_TM_*** or ***f(a)_iTM_*** where ***f(a)_TM_*** and ***f(a)_iTM_*** is the background probability calculated separately for TM and inter-TM regions of the alignment. The background probability is thus replaced with this environment specific background frequency. A classical description of entropy also does not take into consideration the absence of data points which are gaps in the case of a multiple alignment column. We used a scaling factor which is equal to number of amino acid positions excluding gaps in column ***i*** divided by total number of sequences. The scores were then divided by this scaling factor to give scaled RE_M_ score.

#### Site-directed mutagenesis of CaMdr1p

Site-directed mutagenesis was performed by using the Quick-Change Mutagenesis kit (Stratagene, La Jolla, CA, USA) as described previously [46]. The mutations were introduced into the plasmid pRPCaMDR1-GFP according to the manufacturer's instructions, and the desired nucleotide sequence alterations were confirmed by DNA sequencing of the ORF. The primers used for the purpose are listed in Supplementary Table S4. The mutated plasmid, after linearising with *Xba1*, was used to transform AD1-8u^−^ cells for uracil prototrophy by lithium acetate transformation protocol [46]. Integration was confirmed by Southern Blot analysis (data not shown).

#### Preparation of the plasma membranes and immunodetection of CaMdr1p and its mutant variants

The plasma membranes (PM) were prepared from *S. cerevisiae* cells, as described previously [46]. The PM protein concentration was determined by bicinchonic acid assay using bovine serum albumin as the standard. For Western Blot analysis the immunoblot was incubated with anti-GFP monoclonal antibody (1∶5000) (JL-8) (BD Biosciences) as described previously. Immunoreactivity of GFP antibody was detected using goat anti-mouse horseradish peroxidase-labelled antibody (1∶5,000) and was visualized using the enhanced chemiluminescence assay system (ECL kit, Amersham Biosciences, Arlington Heights, IL, USA) [46].

#### Drug susceptibility

The susceptibilities of yeast cells, harboring wild type CaMDR1-GFP and its mutant variants, were tested to different drugs by spot assay. For spot assay, 5 µl samples of five-fold serial dilutions of yeast culture each with cells suspended in normal saline to an OD of 0.1 (1×10^6^ cells) at A_600_ were spotted onto YEPD plates in the absence (control) or in the presence of the drugs [35]. Growth differences were recorded following incubation of the plates for 48 hrs at 30°C.

#### Drug transport of mutant variants

The accumulation of [^3^H] MTX (specific activity, 8.60 Ci/mmol) and [^3^H] FLU (specific activity, 19 Ci/mmol) was determined by protocol described previously [35]. Cells from mid-log phase were centrifuged at 500×g for 3 min and resuspended in fresh YEPD medium as 5% cell suspension. 100 µl of cell suspension was incubated in shaking water bath at 150 rpm at 30°C and [^3^H] MTX was added to a final concentration of. The cells were incubated in [^3^H] MTX (25 µM) or [^3^H] FLU (100 nM) for 30 min, filtered rapidly and washed twice with 1x PBS, pH 7.4 on Millipore manifold filter assembly using 0.45 µm nitrocellulose filter discs (Millipore, U.S.A). The filter discs were dried and put in cocktail-O and the radioactivity was measured in a liquid scintillation counter (Packard, Beckman, USA). The accumulation was expressed relative to the wild type CaMdr1p-GFP.

#### Molecular modeling of CaMdr1p

Structures of known MFS proteins [PDB id – 1pv6 [9], 1pw4 [10] and 1zc7 [11]] were retrieved from Protein Data Bank (www.rscb.org) and aligned using MODELLER9V5 [47]. MFS sequences containing CaMdr1p aligned earlier with PRALINETM were aligned to this structure alignment after removing the sequences which corresponded to known structures. These were aligned using the profile-profile alignment option of ClustalW. The Profile alignment was then manually refined based on helix packing information of known MFS structures. Manual refinement was restricted to incorporating gaps in the loop regions without disturbing either the structural alignment or PRALINE sequence alignment. The homology model for the target sequence CaMdr1p was generated using MODELLER9V5 [47] using 1pv6, 1pw4 and 1zc7 as template sequences. The initial 90 residues from N-terminal of CaMdr1p did not align with any of the templates, and were omitted from further study. The model was evaluated and validated by PROCHECK.

#### Generating the contact map

The contact map displays the distances between Cβ of one residue and Cβ of every other residue within 8 A° distance (Cα is considered in case of glycine). Using the homology model a symmetric contact map for CaMdr1p was generated using a PERL program written in-house.

## Supporting Information

Dataset S1 The PRALINETM alignment of 342 MFS sequences as described in Materials and Methods.
(0.77 MB DOC)

Table S1 RE_M_, RE and conservation scores for all the positions of the MSA of 342 MFS sequences.
(2.01 MB DOC)

Table S2 Comparison of RE_M_ and conservation scores.
(0.26 MB DOC)

Table S3 List of yeast strains used in this study
(0.04 MB DOC)

Table S4 List of oligonucleotides used for site-directed mutagenesis.
(0.05 MB DOC)

Figure S1 Comparative plot of conservation (red), RE (green) and RE_M_ (blue) across the entire alignment. The conservation scores are scaled for comparison with RE and REM. Positions of the highest scoring alignment columns by each method are shown above the graph, along with the results of mutation of the matching residues. Out of these top scoring positions by three different calculations, the mutated positions showing resistant phenotype are marked in red triangles, those showing sensitive on all drugs are marked in green triangles while those which were not mutated are marked by empty triangles. Locations of the transmembrane regions are marked by black bars on the x-axis.
(0.03 MB PDF)
