# Supplementary material for: Rational Mutational Analysis of a Multidrug MFS Transporter CaMdr1p of Candida albicans by Employing a Membrane Environment Based Computational Approach
Source: PLoS Comput Biol. 2009 Dec 24;5(12):e1000624. doi: 10.1371/journal.pcbi.1000624 (PMC2789324; doi:10.1371/journal.pcbi.1000624)
Supplement: Dataset S1 — The PRALINETM alignment of 342 MFS sequences as described in Materials and Methods. (0.77 MB DOC) [file pcbi.1000624.s001.doc]

**The PRALINETM alignment of 342 MFS sequences as described in Materials and Methods.**

P28873 -----MHYRFLRDSFVGRVTYHLSKHKYFAH-----------------------------

A4WFG6 ------------------------------------------------------------

A1JSB0 ------------------------------------------------------------

A8GKP6 ------------------------------------------------------------

P57601 ------------------------------------------------------------

Q8K942 ------------------------------------------------------------

Q89A60 ------------------------------------------------------------

P76198 ------------------------------------------------------------

O05390 ------------------------------------------------------------

O34864 ------------------------------------------------------------

O52717 ------------------------------------------------------------

O52718 ------------------------------------------------------------

A9MJT5 ------------------------------------------------------------

A6TG19 ------------------------------------------------------------

A0L190 ------------------------------------------------------------

P0AEY8 ------------------------------------------------------------

Q7CP73 ------------------------------------------------------------

P39386 ------------------------------------------------------------

Q68WD6 ------------------------------------------------------------

Q4UMJ9 ------------------------------------------------------------

Q1RI77 ------------------------------------------------------------

P32482 ------------------------------------------------------------

P37597 ------------------------------------------------------------

P45123 ------------------------------------------------------------

P28246 ------------------------------------------------------------

P31442 ------------------------------------------------------------

P37482 ------------------------------------------------------------

P76242 ------------------------------------------------------------

P17583 ------------------------------------------------------------

P0C105 ------------------------------------------------------------

O25788 ------------------------------------------------------------

P11551 ------------------------------------------------------------

P44776 ------------------------------------------------------------

A1A9E1 ------------------------------------------------------------

A9MHY5 ------------------------------------------------------------

A4W8S1 ------------------------------------------------------------

A1JMG4 ------------------------------------------------------------

Q0TK80 ------------------------------------------------------------

O06473 ------------------------------------------------------------

Q2FI61 ------------------------------------------------------------

Q5HQE8 ------------------------------------------------------------

Q49WE5 ------------------------------------------------------------

Q4L523 ------------------------------------------------------------

P33026 ------------------------------------------------------------

P31436 ------------------------------------------------------------

Q9S3K0 ------------------------------------------------------------

P31675 ------------------------------------------------------------

P31126 ------------------------------------------------------------

Q58955 ------------------------------------------------------------

P02920 ------------------------------------------------------------

Q4UK37 ------------------------------------------------------------

Q1RKF6 ------------------------------------------------------------

Q4ULW4 ------------------------------------------------------------

Q1RI01 ------------------------------------------------------------

Q68WQ5 ------------------------------------------------------------

Q4UL88 ------------------------------------------------------------

Q92HQ3 ------------------------------------------------------------

Q1RIL0 ------------------------------------------------------------

Q4UMU2 ------------------------------------------------------------

Q68W71 ------------------------------------------------------------

Q1RHK8 ------------------------------------------------------------

Q1LTM2 ------------------------------------------------------------

Q2NTK5 ------------------------------------------------------------

P55705 ------------------------------------------------------------

Q7Z3Q1 ------------------------------------------------------------

Q05B81 ------------------------------------------------------------

P76470 ------------------------------------------------------------

P32135 ------------------------------------------------------------

P38358 ------------------------------------------------------------

A4WAE6 ------------------------------------------------------------

A6T8Y8 ------------------------------------------------------------

Q6CZ44 ------------------------------------------------------------

Q888L8 ------------------------------------------------------------

Q1IB51 ------------------------------------------------------------

A6UZY0 ------------------------------------------------------------

Q4QP52 ------------------------------------------------------------

Q9CM87 ------------------------------------------------------------

O25797 ------------------------------------------------------------

Q17YP7 ------------------------------------------------------------

P77389 ------------------------------------------------------------

O31577 ------------------------------------------------------------

O34367 ------------------------------------------------------------

A1AHK2 ------------------------------------------------------------

A9MWE8 ------------------------------------------------------------

P23910 ------------------------------------------------------------

P31141 ------------------------------------------------------------

P43531 ------------------------------------------------------------

Q8K902 ------------------------------------------------------------

P57648 ------------------------------------------------------------

Q89A23 ------------------------------------------------------------

P37498 ------------------------------------------------------------

P0A0J4 ------------------------------------------------------------

Q07282 ------------------------------------------------------------

P02982 ------------------------------------------------------------

P02981 ------------------------------------------------------------

P70187 ------------------------------------------------------------

Q5SR56 ------------------------------------------------------------

P77726 ------------------------------------------------------------

Q89AA9 ------------------------------------------------------------

Q8K999 ------------------------------------------------------------

P57538 ------------------------------------------------------------

Q5HIA2 ------------------------------------------------------------

Q4L3Q4 ------------------------------------------------------------

Q5HRH0 ------------------------------------------------------------

P0C0L7 ------------------------------------------------------------

Q47421 ------------------------------------------------------------

P0A2G3 ------------------------------------------------------------

P16482 ------------------------------------------------------------

P0AEX3 ------------------------------------------------------------

P76350 ------------------------------------------------------------

P41036 ------------------------------------------------------------

Q9SYQ1 ------------------------------------------------------------

Q9S735 ------------------------------------------------------------

P76230 ------------------------------------------------------------

P38055 ------------------------------------------------------------

Q46909 ------------------------------------------------------------

P31679 ------------------------------------------------------------

O24723 ------------------------------------------------------------

O30513 ------------------------------------------------------------

Q43975 ------------------------------------------------------------

Q51955 ------------------------------------------------------------

Q9I6Q3 ------------------------------------------------------------

P77589 ------------------------------------------------------------

P94131 ------------------------------------------------------------

P71369 ------------------------------------------------------------

O34691 ------------------------------------------------------------

P0AGC0 ------------------------------------------------------------

P27669 ------------------------------------------------------------

P96335 ------------------------------------------------------------

P08194 ------------------------------------------------------------

P37948 ------------------------------------------------------------

P12681 ------------------------------------------------------------

Q5M7K3 ------------------------------------------------------------

Q9WU81 ------------------------------------------------------------

Q58CV5 ------------------------------------------------------------

Q7SY29 ------------------------------------------------------------

P57057 ------------------------------------------------------------

Q17QZ3 ------------------------------------------------------------

Q3TIT8 ------------------------------------------------------------

Q640L2 ------------------------------------------------------------

Q5F3N0 ------------------------------------------------------------

Q09037 -------------MTERR-DNVSH-APDAIEGPNDGAHAEETSPGFFSFENLG-------

Q06222 -------------MTERR-DNVSH-APDAIEGPNDGAHAEDTSPGFFSFENLG-------

P13865 -------MSDRVEVNERRSDSVSEKEPARDDARKDVTDDQEDAPPFMTANNAR-------

P46499 -------------------------------------------MSFLNINRFH-------

P54219 --------------------MLRTIL-------DAPQRLLKE--GRASRQLVL-------

Q6NT16 ------------------MEALGDLEGPRAPGGDDPAGSAGETPGWLSREQVF-------

Q8R0G7 -----------MAGSDTAPFLSQADDPDDGPAPGHPGLPGPMGNPKSGELEVPDCEGLQR

Q5XGK0 -------MTSRRSHGDVTPFLTQADNTEE------EGVRDPESQSSDEEEEEGKDHGKET

Q7ZU13 ---------MSQADADITPFF--ADD-NEGEGPVENGVGSPL--PEDEEEESP-------

A2CER7 -------MASDQHRP--KPRLSLRSSTT-------IRYGSMSSEHPDGDPSTPQ------

Q9D232 -----------MSTECLKPQTGGPQSQSLSQGGQYGALASGTCLPPSTPVP---------

Q6ZMD2 -------MAGGMSAECPEPGPGGLQGQSPGPGRQ--------CPPPITPTS---------

Q6GPQ3 -------MASIDDDDDERTPL--LQDSHIGE-----------LVETQKQLK---------

A5IVG9 -----------------------------------------------------MYK----

Q5HLK7 -----------------------------------------------------MNM----

P46907 ------------------------------------------------------MI----

P10903 ---------------------MSHSSAPERA--------TGAVITDWRPEDPAFWQ----

P37593 ---------------------MTRQN--ENY--------NRYLLSDWRPENPAFWE----

P37758 ---------------------MALQN--EKN--------SRYLLRDWKPENPAFWE----

Q9P3K8 -------MASPTPAPRPDQISASTPLLQSDS--------TSSCASSIRSLSPSRRRHRNG

Q6FWD4 -------------------------MALSSI--------EHYLSYHLRVLLP--------

Q6CPY8 -------------------------MRLSNI--------EHWLTYHIRTWLS--------

P22152 --------------MDFAKLLVASPEVNPNN--------RKALT--IPVLNP--------

Q02563 --MEEGFRDRAAFIRGAKDIAK-EVKKHA---AKKVVKGLDRVQDEYSRRSYSRFEEEED

Q496J9 --MEDSYKDRTSLMKGAKDIAR-EVKKQT---VKKVNQAVDRAQDEYTQRSYSRFQDEED

Q63564 -------------------------------------------MDDYRYR---------D

Q1JP63 -----------------------------------------MEEDLFQLR----------

Q2XWK0 -----------------------------------------MEDDLFQLR----------

Q1LVS8 ------------------------------------------------------------

O08966 ------------------------------------------------MPTVDDVLEHVG

O15245 ------------------------------------------------MPTVDDILEQVG

O77504 ------------------------------------------------MPTVDDVLEQVG

A7MBE0 ------------------------------------------------MLTVDDVLEQVG

Q9R0W2 ------------------------------------------------MSTVDDILEHIG

O02713 ------------------------------------------------MLTVDDILEHTG

Q8MJI6 ------------------------------------------------MPTVDDILEQVG

O88446 ------------------------------------------------MPTFDQALRKAG

O75751 ------------------------------------------------MPSFDEALQRVG

Q9U539 --------MSFQAMETFAEISQ------------EILM------SATKPPDFDFVLEQVG

O76082 ------------------------------------------------MRDYDEVTAFLG

Q497L8 -------------------------------------M------ESCN---VELIFDHIG

Q86VW1 -------------------------------------M------GSRH---FEGIYDHVG

Q17QN9 -------------------------------------M------GSSN---LELIFDSVG

Q95R48 -------------------------------------M------GYD-----EAII-HLG

Q9Y267 MAGEENFKEELRSQDASRNLNQHEVAGHPHSWSLEMLLRRLRAVHTKQDDKFANLLDAVG

Q6A4L0 ------------------------------------------------MAQFAQVMAEVG

Q9Y226 ------------------------------------------------MAQFVQVLAEIG

Q8IVM8 -------------------------------------------------MAFQDLLGHAG

Q66J52 -------------------------------------------------MAFQEILESLG

Q91WU2 -------------------------------------------------MGFEELLHKVG

Q8IZD6 -------------------------------------------------MEVEEAFQAVG

Q6DFR1 -------------------------------------------------MEVEGALKLVG

Q6NUB3 -------------------------------------------------MEVEGALKLVG

Q28ES4 -------------------------------------------------MDLDEAFLYIG

A6NKX4 ------------------------------------------------MEQEARVLRAAG

P47185 ----------------------------------MASEQ-------SSPEINADNLNSSA

P13181 ----------------------------------MAVEENNMPVVSQQPQAGEDVISSLS

P23585 ------------------------------------MSE----FATSRVESG-----SQQ

Q92339 ------------------------------------------------------------

O74969 ------------------------------------------------------------

P10870 --MDPNSNSSSETLRQ-EKQGFLDKA--------LQRVKGIALRRNNSNKDHTTDDTTGS

Q12300 ------MNDSQNCLRQREENSHLNPGN------DFGHHQGAECTINHNNMPHRNAYTEST

P42833 ---------MTAQIPYQHSSGYISH------------------FHNNELDAGR----GRD

P49374 ------------------------------------------------------------

O74713 ------------------------------------------------------------

Q9BE72 --------------------------------------------------------MVPV

Q5J316 --------------------------------------------------------MVPV

Q32NG5 ------------------------------------------------------------

Q6NWF1 -------------------------------------------------------MDAPE

Q0P4G6 ------------------------------------------------------------

Q6GN01 ------------------------------------------------------------

O95528 ------------------------------------------------------------

Q8VHD6 ------------------------------------------------------------

Q3UHK1 --------------------------------------------------MGERRRRQPE

Q96QE2 --------------------------------------------------MGERRRKQPE

Q9C757 ------------------------------------------------------------

Q8VZR6 ------------------------------------------------------------

P30606 --MKNSTAASSRWTKSRLSHFFPSYTNSSGMGAASTDQSSTQGEELHHRKHCEEDNDGQK

P30605 -----------------MGIHIPYLT-------SKTSQSNV-GDAVGNADSVEFNSEHDS

Q10286 ------MSISSKDFQNVTSAGFADDT-----FAADTFAA----DKKSPFESSVFEN----

Q04162 ------------------------------------------------------------

Q01440 ------------------------------------------------------------

P11166 ------------------------------------------------------------

P46896 ------------------------------------------------------------

P47843 ------------------------------------------------------------

P14672 ------------------------------------------------------------

Q90592 ------------------------------------------------------------

P11168 ------------------------------------------------------------

Q5RB09 -------------------------------------------------MARKQNRNSKE

A4ZYQ5 ------------------------------------------------------------

P22732 ------------------------------------------------------------

P58353 ------------------------------------------------------------

Q863Y9 ------------------------------------------------------------

P43427 ------------------------------------------------------------

Q9WV38 ------------------------------------------------------------

P15686 ------------------------------------------------------------

Q39525 ------------------------------------------------------------

Q94AZ2 ------------------------------------------------------------

Q10710 ------------------------------------------------------------

Q41144 ------------------------------------------------------------

P23586 ------------------------------------------------------------

O65413 ------------------------------------------------------------

Q9SX48 ------------------------------------------------------------

Q9LT15 ------------------------------------------------------------

Q9FMX3 ------------------------------------------------------------

Q9SBA7 ------------------------------------------------------------

Q9SFG0 ------------------------------------------------------------

Q8L7R8 ------------------------------------------------------------

Q93Y91 ------------------------------------------------------------

P0AE24 ------------------------------------------------------------

P0AEP1 ------------------------------------------------------------

P96710 ------------------------------------------------------------

P54723 ------------------------------------------------------------

O34718 ------------------------------------------------------------

P46333 ------------------------------------------------------------

O52733 ------------------------------------------------------------

P0AGF4 ------------------------------------------------------------

P21906 ------------------------------------------------------------

P15729 ------------------------------------------------------------

Q6AWX0 ------------------------------------------------------MALDPE

Q93YP9 ----------------------------------------------------------MS

Q94AF9 ------------------------------------------------------------

O04036 ------------------------------------------------------MERQKS

Q94KE0 ------------------------------------------------------------

Q9SCW7 ------------------------------------------------------------

Q4F7G0 ------------------------------------------------------------

Q8LBI9 --------------------------------------------------------MAIR

P93051 ----------------------------------------------------------MS

Q0WQ63 ------------------------------------------------------------

Q3ECP7 ------------------------------------------------------MRGEID

Q9LTP6 ------------------------------------------------------MGDEPL

Q8GXK5 ------------------------------------------------------MAEESL

Q9JJZ1 -----------------------------------------------------------M

P58354 -----------------------------------------------------------M

Q9NY64 -----------------------------------------------------------M

Q9UGQ3 ------------------------------------------------------MQEPLL

P43562 ------------------------------------------MTAMKAIVWRLPK-----

Q9FYG3 ------------------------------------------MLGLQRETSSMYKRTSSR

P36035 --MSSSITDEKISGEQQQPAGRKLYYNTS-TFAEPPLVDGEGNPINYEPEVYNPDHEKLY

P47186 --MKN--LSFLINRRKENTSDSNVYPGKA-KSHEPSWIEMDDQT---KKDGLDIVHVEFS

A6QLI1 --------------------------------------MESVKQRILTPGKEGLKNFA--

Q5W8I7 --------------------------------------MDTVKERVLAPGKEKMRNLA--

A4FV52 ----------------------------------------------MEFRQEEFRKLA--

P34644 --------------------------------------MSSWNE-----AWDRGKQMV--

Q66GI9 -------MCYSLSIQSSIDFHNRNALKIHGDRAILTSNLPTLRRIPFLPERDRRRKLVLC

Q9FKV1 ------------------------------------------------------------

Q46916 ------------------------------------------------------------

Q91Y77 --------------------------------MVPSLEEPAAAER----ETNEAQP---P

Q8TF71 --------------------------------MVLSQEEPDSA-R----GTSEAQP---L

A1L1W9 ----------------------------------MTEPEPTLEQE----PTPEPEP---T

P36021 ---------MALQSQASEEAKGPWQEADQEQQEPVGSPEPESEPE----PEPEPEPVPVP

O35308 ------------------------------------------------------------

O95907 ------------------------------------------------------------

Q90632 ------------------------------------------------------------

O35910 ------------------------------------------------------------

O15427 ------------------------------------------------------------

P57788 ------------------------------------------------------------

O15375 ------------------------------------------------------------

Q6ZSM3 ------------------------------------------------------------

Q8BGC3 ------------------------------------------------------------

Q503M4 ------------------------------------------------------------

O15403 ------------------------------------------------------------

Q8NCK7 ------------------------------------------------------------

Q7RTY0 ------------------------------------------------------------

Q5R5M4 ------------------------------------------------------------

Q7TM99 ------------------------------------------------------------

Q5ZJU0 ------------------------------------------------------------

O15374 ------------------------------------------------------------

Q08777 --------------MSSDSLTPKDTIVPEEQTNQLRQPDLDEDSIHYDPEADDLESLETT

Q08268 --------------------MLNIPIIANSKRFLFSKDHEAQSTRDHDVELETREG--PS

P39709 --------MYSIVKEIIVDPYKRLKWGFIPVKRQVEDLPDDLNSTEIVTISNSIQSHETA

P25621 ------------------------------------------------------------

O13880 ---------------------MASEWPETSRASSVEENP-KLNIPEIVESV-SDSKPSLK

P40445 ---------------------MSVQKEE----YDIVEKA-QLSVS--AESLTSDSESISH

P15365 ---------------------MSADASTNSNASL--DEK-NLNITS-EAEIKNEDVT-AE

Q07904 ----------------------MKNMSQRS-MDV--EKK-AANADSCSVSTSSINVD-DA

P53322 ---------------------MSNKFTMESPKHLVDDVL-FISPTNDGSEEKPTEVTFQE

P32071 ------MAAFIKDSFWGQIIYRLSGRKLFRH-----------------------------

P38124 -----MVYT---STYRHTIVVDLLEYLGIVS-----------------------------

Q06451 MNRQESINSFNSDETSSLSDVESQQPQQYIPSESGSKSNMAPNQLKLTRTETVKSLQDMG

P53283 MSDQESVVSFNSQNT-SMVDVEGQQPQQYVP----SKTNSRANQLKLTKTETVKSLQDLG

Q9C0R8 -------MSHATDSTLDNASVDSEKVRDF-------GDDLQNHPVQPTRSIL--------

Q9C0Q6 -------MSDNT--TLDNISVNSEKVVDY----------TIHH---DDRKEL--------

Q9HF77 MN--NNTNSNHNDIAPEATITQNTTTSVSNDELQHITNNNNVNVQSYVG-PTDSVESSSN

Q5A0E9 ---------------------MPASGSISSEE-------PLMTLKPYEGIPT--IE----

Q07824 -------MSDHSPISNKENHLLPSDSSRSSS--------SDMHSTGTTG--TTGVEPVDF

O59698 MS--ESSVNADTP-KNTNDVLNGAYQSATTEPEGQY--RSATDNPSLYQVPTHG------

O74829 ------------------------MAERTSE--------SSSESASF-------------

P38776 ------------------------------------------------------------

Q9HDX4 ------------------------------------------------------------

P38227 -------MQAQGSQSNVGSLRSNCSDNSLPNNHVMMHCDESSGTPHSEHN----------

P38125 -------MGSEPFQKKNLGLQINSQESGTTRSTFHSLEDLGDDVINESWD----------

P40474 ------------------------------------------------------------

O94607 ------------------------------------------------------------

Q9HE13 ------------------------------------------------------------

Q04301 ------------------------------------------------------------

P13090 ------------------------------------------------------------

Q08902 ------------------------------------------------------------

P39886 ------------------------------------------------------------

P76269 ------------------------------------------------------------

Q8Y9K8 ------------------------------------------------------------

P28873 ------------PEEAKDYIVPEKYLADYKPTLADDTSINFEKEEIDNQGEPNSSQSSSS

A4WFG6 -----------------------------------------------------------M

A1JSB0 -----------------------------------------------------------M

A8GKP6 -----------------------------------------------------------M

P57601 -----------------------------------------------------------M

Q8K942 -----------------------------------------------------------M

Q89A60 ----------------------------------------------------------MI

P76198 ----------------------------------------------------------MK

O05390 ----------------------------------------------------------MN

O34864 ------------------------------------------------------------

O52717 ------------------------------------------------------------

O52718 ------------------------------------------------------------

A9MJT5 ------------------------------------------------------------

A6TG19 ------------------------------------------------------------

A0L190 ------------------------------------------------------------

P0AEY8 -----------------------------------------------------------M

Q7CP73 -----------------------------------------------------------M

P39386 -----------------------------------------------------------M

Q68WD6 -----------------------------------------------------------M

Q4UMJ9 -----------------------------------------------------------M

Q1RI77 -----------------------------------------------------------M

P32482 ------------------------------------------------------------

P37597 -----------------------------------------------------------M

P45123 ------------------------------------------------------------

P28246 -----------------------------------------------------------M

P31442 -----------------------------------------------------------M

P37482 ----------------------------------------------------------MP

P76242 ----------------------------------------------------------MT

P17583 ------------------------------------------------------------

P0C105 --------------------------------------------------MA-TSIPTNN

O25788 ------------------------------------------------------------

P11551 --------------------------------------------------MGNTSIQTQS

P44776 ----------------------------------------------------------MN

A1A9E1 ------------------------------------------------------------

A9MHY5 ------------------------------------------------------------

A4W8S1 ------------------------------------------------------------

A1JMG4 ------------------------------------------------------------

Q0TK80 --------------------------------------------------MNKQSWLLNL

O06473 --------------------------------------------------MDKTTQVNQK

Q2FI61 ----------------------------------------------------MQDSSLNN

Q5HQE8 ----------------------------------------------------MQDSSSSN

Q49WE5 ----------------------------------------------------MQSSSLNN

Q4L523 ----------------------------------------------------MPDSSLSN

P33026 --------------------------------------------------MHNSPAVSSA

P31436 --------------------------------------------------MQKT-ATTPS

Q9S3K0 --------------------------------------------------MTISSARTAR

P31675 ----------------------------------------------------MIWIMTMA

P31126 ----------------------------------------------------------MN

Q58955 ----------------------------------------------------------MG

P02920 ----------------------------------------------------------MY

Q4UK37 ---------------------------------------------------------MYK

Q1RKF6 --------------------------------------------------MFLIKKISID

Q4ULW4 ---------------------------------------------------MLITKI-FK

Q1RI01 ---------------------------------------------------MLITRI-FK

Q68WQ5 --------------------------------------------------------MLKN

Q4UL88 --------------------------------------------------------MLNN

Q92HQ3 --------------------------------------------------------MLNN

Q1RIL0 -----------------------------------------------------------M

Q4UMU2 --------------------------------------------------MNFNFSRFQY

Q68W71 --------------------------------------------------MNFKCVQFQS

Q1RHK8 --------------------------------------------------MISNFSRLQN

Q1LTM2 -----------------------------------------------------MSSVSQA

Q2NTK5 -----------------------------------------------------MSSVSQA

P55705 ----------------------------------------------------MTRTVAVL

Q7Z3Q1 ------------------------------------------------------------

Q05B81 --------------------------------------------------MEGRANSPGE

P76470 ------------------------------------------------------------

P32135 ------------------------------------------------------------

P38358 --------------------------------------------------MSISNWITTA

A4WAE6 ------------------------------MTTNT-------------------VS-RK-

A6T8Y8 ------------------------------MTTNT-------------------VS-RK-

Q6CZ44 ------------------------------MTRS---------------------P-RS-

Q888L8 ------------------------MITSPPNEQTG-------------------E--HT-

Q1IB51 ------------------------MNGPVPSNLPP-------------------TA-GN-

A6UZY0 ------------------------------MHSTS-------------------ET-RS-

Q4QP52 ------------------------------MSLYL-------------------KA-EK-

Q9CM87 ------------------------------MLPFQ-------------------AA-RQ-

O25797 -----------------------------MMITKQ-------------------SY-QK-

Q17YP7 -----------------------------MMITKQ-------------------SY-KK-

P77389 ----------------------------------------------------------M-

O31577 ---------------------------------MS-------------------IK-NP-

O34367 ---------------------------------MT-------------------SA-NK-

A1AHK2 ------------------------MSEFIAENRGA-------------------DAITR-

A9MWE8 ------------------------MNENIAEKFRA-------------------DGVAR-

P23910 ------------------------------------------------------------

P31141 ------------------------------------------------------------

P43531 ------------------------MSRTTTVDGAPASDTD---KQSISQPNQFIKRGTP-

Q8K902 ------------------------MDIYTYKKHILFKKCF---LYFWKIKNVIKKKNTK-

P57648 ------------------------MTLLKNKKQLLEKQ------YI--------KKNTK-

Q89A23 ------------------------MQLKIYSSN---KN------YI--------QRGTK-

P37498 --------------------------------------------------MTYIRKGTP-

P0A0J4 -------------------------------------------------------MNKQ-

Q07282 -------------------------------------------------------MNRT-

P02982 -----------------------------------------------------MKPNRP-

P02981 -----------------------------------------------------MKSNNA-

P70187 ------------------------MTQGKKKKRAANRSIMLAKKIIIKDGGTPQGIGSP-

Q5SR56 ------------------------MPEKRAGAQAAGSTWL-------------QGFGRP-

P77726 ------------------------MNDYKMTP-----------------------GERR-

Q89AA9 ------------------------MSFDEIKT-----------------------HNKK-

Q8K999 ------------------------MKNYKMNF-----------------------IELQ-

P57538 -----------------------------MNF-----------------------LELQ-

Q5HIA2 ------------------------MDFNKEN-----------------INMVDAKKAKK-

Q4L3Q4 ------------------------MDFKKDR-----------------INMVDGQTAKK-

Q5HRH0 --------------------------------------------------MVDGNNAKK-

P0C0L7 -------------------------MLKRKKVKP---------ITLRDVTIIDDGKLRK-

Q47421 ------------------------MKLKRKRVKP---------IALDDVTIIDDGRLRK-

P0A2G3 --------------------------------MA---------QHT-P----ATSRAGTF

P16482 ------------------------MPTARCSMRA---------SSTAPVRMMATAGGARI

P0AEX3 --------------------------MAESTV-------------TADSKLTSSDTRRRI

P76350 ------------------------MDSTLISTRP-------------DEGTLSLSRARR-

P41036 ------------------------MSTTTQN-----------------IPWYRHLNRAQW

Q9SYQ1 ------------------------MP-IK---------------VLSSLDVARTQWYHFK

Q9S735 ------------------------MPELS---------------LLSALDAARIQWYHFK

P76230 ------------------------MEQITKP------------HCGARLDRLPDCRWHSS

P38055 ------------------------MEQYDQ--------------IGARLDRLPLARFHYR

Q46909 ------------------------MNTSP-----------------VRMDDLPLNRFHCR

P31679 ------------------------MQPSR------------------NFDDLKFSSIHRR

O24723 ------------------------MNTSPGAPT----------GVRAFVDSQPIGRRQRI

O30513 ------------------------MSREIN--------------VNQMIDDSKLTPFHWR

Q43975 ------------------------MPKEANMASQDYATQRSSLDAQALINDAPLSRYQWL

Q51955 ----------------------------MNQAQNSVG---KSLDVQSFINQQPLSRYQWR

Q9I6Q3 ----------------------------MNSPSLPAV---ERLDVQAFINAQPLSPYQWR

P77589 ---------------------------------------------MSTRTPSSSSSRLML

P94131 --------------------------------------------MYSNNQRSRIGSHTWK

P71369 -------------------------------------------------MTNKVNSYGWK

O34691 --------------------------------------------------MGKQQPISQR

P0AGC0 -------------------------------------MLAFLNQVRKPTLDLPLEVRRKM

P27669 -------------------------------------MLSFLKAPANAPLITDKHEVDAR

P96335 -------------------------------------MFGPFK-PAPHIAELPAEKIDST

P08194 -------------------------------------MLSIFK-PAPHKARLPAAEIDPT

P37948 -------------------------------------MLNIFK-PAPHIERLDDSKMDAA

P12681 -------------------------------------MLTILK-TGQSAHKVPPEKVQAT

Q5M7K3 -------------------------------------------MRSSLAPAIRLMQSVSR

Q9WU81 -------------------------------------------MRSSLAPGVWFLRAFSR

Q58CV5 -------------------------------------------MRSSLAPGI--------

Q7SY29 --------------------------------------------MKSLAPGIKLITSFSR

P57057 --------------------------------------------MARLPAGIRFIISFSR

Q17QZ3 -----------------------------------------MAWP----RIFQRGALLSR

Q3TIT8 -----------------------------------------MAWP----RFLQRGALLTS

Q640L2 -----------------------------------------MDFPVNTRRMAGRRGFFSQ

Q5F3N0 -----------------------------------------MAVPGSSRRQPSNRGLVSH

Q09037 -----------VAQVQV-V-GG------------------------------TLNGYVIG

Q06222 -----------VAQVQV-V-GG------------------------------TLNGFSIG

P13865 -----------VMLVQA-I-GG------------------------------SLNGYSIG

P46499 -----------ILCFFLWQ-FG------------------------------LFYACQLI

P54219 -----------VVVFVALL-LD------------------------------NML-FTVV

Q6NT16 -----------VLISAASVNLG------------------------------SMMCYSIL

Q8R0G7 -----------ITGLSRGHSTL------------------------------IVVVLCYI

Q5XGK0 HL---------LTGISYKRSVI------------------------------IVIILFYI

Q7ZU13 ------------SGVTDRRAIM------------------------------TVIVLCYI

A2CER7 -----------TTSISQRRSYI------------------------------AVAVLCYI

Q9D232 ------------WSLPRWRAYL------------------------------AAAVLCYI

Q6ZMD2 ------------WSLPPWRAYV------------------------------AAAVLCYI

Q6GPQ3 ---------------SRWWSIR------------------------------VMYLTMFL

A5IVG9 -----------TKGGF----QL------------------------------TLQTLSLV

Q5HLK7 -----------KKGVS----QL------------------------------TLQTLSLV

P46907 -----------NRQHI----QL------------------------------SLQSLSLV

P10903 -----------QRGQRIASRNL------------------------------WISVPCLL

P37593 -----------NKGKGIARRNL------------------------------WISVSCLL

P37758 -----------NKGKHIARRNL------------------------------WISVSCLL

Q9P3K8 RT---------SPAAAASARNL------------------------------SFASALLS

Q6FWD4 -----------QVLSSKSSHNI------------------------------AYIFALFA

Q6CPY8 -----------NSFEWSTIHNS------------------------------TFIISLFS

P22152 --------------FNTYGRVF------------------------------FFSWFGFM

Q02563 DD-------DFPAPADGYYRGEGAQDEEEGGASSDATEGHDEDDEIYEGEYQGIPRAES-

Q496J9 DD-------DY-YPAGETYNGE-ANDDE---GSSEATEGHDEDDEIYEGEYQGIP-SMN-

Q63564 NY-------EGYAPNDGYYRGNEQNPEED--AQSDVTEGHDEEDEIYEGEYQGIPHPDDV

Q1JP63 -----------QLPVVKFRRTGESARSEDDTASGE----HEVQIEGVRAGLEAVELDD--

Q2XWK0 -----------HLPVVKFRRTGESSKSEDDNISGE----HEIQIGPVQTELEAVELED--

Q1LVS8 ---------------MALKRSSSMKTQLVDAIQLE-----EVEME---EEITTTSNNN--

O08966 EF-------GWFQKQAFLLLCL-ISASLAPIYVGIVFLGFTPDHH-CRSPGVAELSQRCG

O15245 ES-------GWFQKQAFLILCL-LSAAFAPICVGIVFLGFTPDHH-CQSPGVAELSQRCG

O77504 EF-------GWFQKRTFLFLCL-ISAILAPIYLGIVFLGFTPDHR-CRSPGVDELSQRCG

A7MBE0 EF-------GWFQKQTFLILCL-LSAAFAPIYVGIVFLAFTPDHR-CRSPGVAELSRRCG

Q9R0W2 EF-------HLFQKQTFFLLAL-LSGAFTPIYVGIVFLGFTPDHH-CWSPGAAKLSQRCG

O02713 EF-------NFFQKQTFFLLAL-LSAAFTPIYVGIVFLGFIPDHR-CRSPGVAELSQRCG

Q8MJI6 HF-------HFFQKQTFFLLAL-ISAAFTPIYVGIVFLGFTPDHR-CRSPGVAELSQRCG

O88446 EF-------GRFQRRVFLLLCL-TGVTFAFLFVGVVFLGSQPDYYWCRGPRATALAERCA

O75751 EF-------GRFQRRVFLLLCL-TGVTFAFLFVGVVFLGTQPDHYWCRGPSAAALAERCG

Q9U539 NY-------GTYQIVFFFIICLPTSLPSAFSAFNIPFVVGNPPHT-CHIPEGKEYLR--P

O76082 EW-------GPFQRLIFFLLSA-SIIPNGFTGLSSVFLIATPEHR-CRVPDAANLSS--A

Q497L8 HF-------GRFQIVLYLICAY-QSLSCGIHYLSSVFLSIIPEHA-CKPPGMVRKAVFHN

Q86VW1 HF-------GRFQRVLYFICAF-QNISCGIHYLASVFMGVTPHHV-CRPPGNVSQVVFHN

Q17QN9 HF-------GRYQIFLYFICAF-QNISCGIHYLASVFLSVSPQHT-CRPPGNVSQVLFQD

Q95R48 DF-------GRYQKIIYFLICL-TSIPVAFHKLAGVFLLAKPDFR-CALP-------FEN

Q9Y267 EF-------GTFQQRLVALTFI-PSIMSAFFMFADHFVFTAQKPY-CNTSWILAVGP--H

Q6A4L0 DF-------GRFQVRLTILMGI-PNFLAAFFIFGQVFMVLDEAHH-CSVSWVKNHTF--N

Q9Y226 DF-------GRFQIQLLILLCV-LNFLSPFYFFAHVFMVLDEPHH-CAVAWVKNHTF--N

Q8IVM8 DL-------WRFQILQTVFLSI-FAVATYLHFMLENFTAFIPGHR-CWVHILDNDTVSDN

Q66J52 GM-------GRYQVIHVVLLSL-PVFMLASHNLMQNFTAATPSHH-CRINGTYEET---N

Q91WU2 GF-------GPFQLRNLVLLAL-PRFLLPMHFLLPIFMAAVPAHH-CALPDAPA-----N

Q8IZD6 EM-------GIYQMYLCFLLAVLLQLYVATEAILIALVGATPSYHWDLAELLPNQSHGNQ

Q6DFR1 EM-------GIYQIYLSFLLAVLLQLYSATEAIIITILGATPPYHWMNDSLTANGSRGKQ

Q6NUB3 EM-------GVYQIYLSFLLAVLLQLYSATEAILITIVGVTPPYHWVNDSFVANGSQRNE

Q28ES4 EF-------GCCQKRLTAFL-TLLQVYVACQSMLIVLVGAVPEY------LIDNED----

A6NKX4 GF-------GRARRLLASASWV-PCIVLGLVLSSEELLTAQPAPH-CRPDPTLLPPALRA

P47185 AD-------VHVQPPGEKEWSD----GFYDKEVI-------NGNTPDAPKRGFLGYLIIY

P13181 KD-------SHLSAQSQKYSNDELKAGESGSEGS-------QSVPIEIPKKPMSEYVTVS

P23585 TS-------IHSTPIVQKLETDESPI-QTKSEYT-------NA---ELPAKPIAAYWTVI

Q92339 -----------------------------------------------------MNRFITS

O74969 -------------------------------------------------MGFKRGKNFTL

P10870 IR-------TPTSLQRQNSDRQSNMTSVFTDDIS---TIDDNSILFSEPP-QKQSMMMSI

Q12300 ND-------TEAKSIVMCDDPNAYQISYTNNEPAGDGAIETTSILLSQPL-PLRSNVMSV

P42833 YN-------VTIKYLDDKEENIEGQAAKISHN---------------------ASLHIPV

P49374 ----------MSLKNWLLLRDIQYE-GTFYKK--------------------FPHVYNIY

O74713 ----------MSSKIERIFSGPALKINTYLDK--------------------LPKIYNVF

Q9BE72 EN-------TEGPNLLNQKGTAVETEGSYRAS--------GSRHP---PWARGCGMFTFL

Q5J316 EN-------AEGPSLLKPKGRAAETDGSDRAS--------GGPHP---PWARGCGMYTLL

Q32NG5 ---------------MLAHSTAQDLILQQRSS--------DD-HPQTNPRQTGCGAFIIL

Q6NWF1 ES-------IRMTSDPQSKIYVQNPDTHIHLE--------QGPSA-----KSGNGRALVL

Q0P4G6 ---------------------------------------------------MGLSSPTLI

Q6GN01 ---------------------------------------------------MGLRSTTLV

O95528 ---------------------------------------------------MGHSPPVLP

Q8VHD6 ---------------------------------------------------MGLRPAVLL

Q3UHK1 PG-------A--PGGERSLL-AAESAASLQGA--------ELERAARRQFQRDETPAFVY

Q96QE2 PD-------AASAAGECSLLAAAESSTSLQSAGAGGGGVGDLERAARRQFQQDETPAFVY

Q9C757 --------------MEGGIIHGGADE---S--------------AFKECFSLTWKNPYVL

Q8VZR6 --------------MTLTIPNAPGSSGYLD--------------MFPERRMSYFGNSYIL

P30606 P--------KKSPVSTSTMQIKSRQDEDEDDGRIVIKPVNDEDDTSVIITFNQSISPFII

P30605 PS-------KRGKITLESHEIQ-RAPASDDEDRIQIKPVNDEDDTSVMITFNQSLSPFII

Q10286 ---------KTQVLPVDSVSRLSNGARSRSNSNISLSEPHALNDT----VEDQPVSKWVW

Q04162 -------------MSTDESEDV-YSDLYSIISQVTSNTANDIEQLPYALTFKTSL-----

Q01440 ---------------------------------------------------------MRA

P11166 --------------------------------M-------EP--------SSKKLTGRLM

P46896 --------------------------------M-------E---------SGSKMTARLM

P47843 -----------------------------------------M--------GTTKVTTPLI

P14672 --------------------MPSGFQQIGSEDG-------EP--------PQQRVTGTLV

Q90592 ---------------------MDGKSKMQAEK---------------------HLTGTLV

P11168 ----------------------------MTED---------------------KVTGTLV

Q5RB09 LG-------LAPLADDTSHAGPPGPGRALLECD-------HLRSGLPDGRRRKDWSCSLL

A4ZYQ5 -----------------------------MENK-------EAGTPPPIPSREGRLQPTLL

P22732 -----------------------------MEQQ-------DQ------SMKEGRLTLVLA

P58353 -----------------------------MEPQ-------DP------VKREGRLTPVIV

Q863Y9 -----------------------------MEQQ-------DP------IKKEGRLTPVLA

P43427 -----------------------------MEKE-------DQ-------EKTGKLTLVLA

Q9WV38 -----------------------------MEEK-------HQ-------EETGELTLVLA

P15686 -----------MAGGGVVVVSGRGLSTGDYR---------------------GGLTVYVV

Q39525 -----------MA-GGAIVASGGASRSSEYQ---------------------GGLTAYVL

Q94AZ2 -----------MTGGGFATSA----NGVEFE---------------------AKITPIVI

Q10710 -----------MAGGSLAPAGVAKERAEQYQ---------------------GKVTFAVF

Q41144 -----------MPAVGGIPPS--GGNRKVYP---------------------GNLTLYVT

P23586 -----------MPAGGFV--V--GDGQKAYP---------------------GKLTPFVL

O65413 -----------MPSVGIV--I--GDGKKEYP---------------------GKLTLYVT

Q9SX48 -----------MAGGAFVSEG--GGGGNSYE---------------------GGVTVFVI

Q9LT15 -----------MAGGAFVSEG--GGGGRSYE---------------------GGVTAFVI

Q9FMX3 -----------MAGGAFIDES--GHGG-DYE---------------------GRVTAFVM

Q9SBA7 --------------MAVVISS--NGNSKSFD---------------------AKMTVYVF

Q9SFG0 --------------MAVVV-S--NANAPAFE---------------------AKMTVYVF

Q8L7R8 -----------MVAEEARKEA---MAKSVSG---------------------GKITYFVV

Q93Y91 -----------MAGGGLALDV---SSAGNID---------------------AKITAAVV

P0AE24 -------------------------MVTINTES------------ALTPRSLRDTRRMNM

P0AEP1 --------------------------------M------------PDAKKQGRSNKAMTF

P96710 -------------------------MKNTPTQL-----------EPNVPVTRSHSMGFVI

P54723 -------------------------MSTKKKE-------------AVIGKESLAHKGLLR

O34718 -----------------------------------------------MNKQG-NQMSFLR

P46333 -----------------------------------------------MKKDT---RKYM-

O52733 -----------------------------------------------MRKVS---TGF--

P0AGF4 -------------------------MNTQYNS-----------------S-------YIF

P21906 -------------------------MSSESSQ-----------------G-------LVT

P15729 -------------------------MNPSSSP-----------------SQSTANVKFVL

Q6AWX0 QQ-------QPISSVSREFGKSSGEISPEREPL-----------IKENHVPENYSVVAAI

Q93YP9 FR-------DDNTEEGRNDLRRPF-----LHTGSWYRMGSRQSSMLESSQVIRDSSISVL

Q94AF9 --------------MVVEEENRS-------MEEGLL--------QHQNDRDDRRITACVI

O04036 ME-------KGLLRKSLSIRERKFPNEDAFLESGLSRKSPREVKKPQNDDGECRVTASVF

Q94KE0 --------------MTMSENSRN-------LEAGLL------LRKNQNDINECRITAVVL

Q9SCW7 ME-------SGSMKTPL-------------------------VNNQEEARSSSSITCGLL

Q4F7G0 ME-------SERLESHL-------------------------LNKQEE--EASSFTSGLL

Q8LBI9 EI-------KDVERGEIVNKVED-------LGKPFLT-----HEDDEKESENNESYLMVL

P93051 KA-------SDAVREPLVDKNMA-------GSKP-------------------DQPWMVY

Q0WQ63 ----------METRKDDMEKRND-------KSEPLLL-----PENGSDVSE--EASWMVY

Q3ECP7 EA-------NLAPETSLINKENQ-------DSSATIT-------------------TTLL

Q9LTP6 LQ-------KVKIQEDIESVPLLQKVKIQEDIESVKG-----IRVNNDGEEDGPVTLILL

Q8GXK5 LP-------SHTEDVSASPNK----------SSSLL------------SEISNASTRPFV

Q9JJZ1 SP-------EDPQ-ETQPLLRS----------------------PGARAPGG----RRVF

P58354 TP-------EDQE-ETQPLLRP----------------------PGGSAPRG----RRVF

Q9NY64 TP-------EDPE-ETQPLLGP----------------------PGGSAPRG----RRVF

Q9UGQ3 GA-------EGPDYDTFPEKPPP--------------------SPGDRARVGTLQNKRVF

P43562 ---------MPKIKITKT-----------YEV-------------------TK-------

Q9FYG3 DY-------SPMIDVEDSSGLLENDVDNEMET-------------------TNPSWKCSL

P36035 HN-------PSLPAQSIQDTRDDELLERVYSQDQGVEYEEDEEDKPNLSAASIKSYALTR

P47186 PD-------TRAPSDS------NKVITEIFDATEDAK-EADESERGMPLATALNTYPKAA

A6QLI1 -G---------KSLGQIYRVLEKKQD-AG---------------ETIELTEDG--KPLEV

Q5W8I7 -G---------KTLGHMHRVMERKQK-TG---------------EVIELTEDG--RPMHM

A4FV52 -G---------RALGKLHRLLEKRQE-GA---------------ETLELSADG--RPVTT

P34644 -G---------EPLAKMTAAAASATG-AA---------------PPQQMQEEGNENPMQM

Q66GI9 TG---------RVVNSLKFTGNTSVDLCG---------------IPRHRLRVSCSDARRT

Q9FKV1 ------------------------------------------------------------

Q46916 ---------------------------------------------------------MSS

Q91Y77 GP---------APSDDAPLPVPGPSDVSD---------------GSVE---KVEVELT-R

Q8TF71 GP---------APTGAAPPPGPGPSDSPE---------------AAVE---KVEVELAGP

A1L1W9 -Q---------EPTPE-PTPEPEPTQEPE---------------SEPELELKQENGCTKA

P36021 PP---------EPQPE-PQPLPDPAPLPE---------------LEFESERVHEPEPTPT

O35308 ------------------------------------------------------MGAGGP

O95907 ------------------------------------------------------MGAGGP

Q90632 ------------------------------------------------------MGRADR

O35910 ------------------------------------------------------MGGAVV

O15427 ------------------------------------------------------MGGAVV

P57788 ------------------------------------------------------MGAVVV

O15375 ------------------------------------------------------------

Q6ZSM3 ------------------------------------------------------MAKVNR

Q8BGC3 ------------------------------------------------------MTKITR

Q503M4 ------------------------------------------------------MAQEKK

O15403 ------------------------------------------------------MTQNKL

Q8NCK7 ------------------MPAPQRKHRRG---------------G-------FSHRCFPT

Q7RTY0 ------------------------------------------------------------

Q5R5M4 ------------------------------------------------------------

Q7TM99 ------------------------------------------------------------

Q5ZJU0 ------------------------------------------------------------

O15374 ----------------------------------------------------------ML

Q08777 AS-------YASTSVSAKVYTKKEVNK-G---------------TDIESQPHWGENTSST

Q08268 SG-------YNPNFNAADAILKKNSDQVD---------------LDVNKLTNVTSRVLNT

P39709 EN-------FITTTSEKDQLHFETSSYSEHKDNVNVTRSYEYRDEADRPWWRFFDEQEYR

P25621 -------------------MMKESKSITQH--------------EVERE----------S

O13880 NQ-------FSTTVIDSSDLNVF-NDGA----------------ETTVK-----EQEFTS

P40445 NP-------FDDFHKAERWRKVYESSGY----------------EGLSKF----DPEFTW

P15365 PV-------LSTVLSPNGKIVYISDKVD----------------EAMKLA---EEAKEIE

Q07904 DV-------ALRFLKQNG--LDESSTAN----------------EDDVVA---GEEANFY

P53322 DE-------GHDASLHNRSHDKKSELAT----------------EREIMATTTDDDGIPS

P32071 ------------NDELPDYVVPEKYLLD--P-----------KEEVLNSSD--KSQ-SSE

P38124 ------------NLETLQSAREDE---TRKPE-------NTDKKECKPDYDIECGPNRSC

Q06451 VSSKAPVPDVNAPQSSKNKIFPEEYTLETPTGLVPVATLHSIGRTSTAISRTRTRQIDGA

P53283 VTSAAPVPDINAPQTAKNNIFPEEYTMETPSGLVPVATLQSMGRTASALSRTRTKQLNRT

Q9C0R8 --SKIRSRDDDARSLVSNNKGVERIISDLQEGAGQLGPLEQ----PYDIKKIETHPDPHT

Q9C0Q6 --ER----------LVSHNKGVEKIVSELAEGAGQLGPLEQ----PYDIHKVETHPDPHT

Q9HF77 TADEENEINSFNAQNVKDYEANVGGELPPDDEL---SRIESNTELSRRATRSIMNTESLL

Q5A0E9 -GDE---------QYLQNYSTAQAGE----NEL---LRIESNVQASKALSRIISDSDPVL

Q07824 TGE--------GAKYTTATEGNGGADLAIQRTTTMNSAAESEVNITRRLTKILTGSVNEP

O59698 -------------SLYRNLSNSASAYYPANGNM-------NSREPANELSDISSLAEKGE

O74829 -------------DLEKQQSNHHDRYQ----------------------SSVSS-----E

P38776 -------------------------------------MVAEFQIASAQSSALTSTEEEHC

Q9HDX4 --------------------------------------------------------MSVS

P38227 -----------DYSYEKTNLESTASNSREHR------DN------QLSRLKSEEYVV--P

P38125 -----------QVNQKRANIDHDVFH--EHP------DSSPSLSAQKAKTKEEEVAVKSS

P40474 --------------------------------------MAGATSSIIRENDFEDELAESM

O94607 ---------------------------------MTENYGSMEHRKKSFRNNENLEQQFHP

Q9HE13 ------------------------------MNPSTSPNRNLASPKSLQLYTTGSEVAWYP

Q04301 -----------------------------------------------------------M

P13090 ----------------------------------MGNQSLVVLTESKGEYENETELPVKK

Q08902 ---------------------------------------------------MSTSSSVTQ

P39886 ------------------------------------------------------------

P76269 ------------------------------------------------------------

Q8Y9K8 ------------------------------------------------------------

P28873 NNTIVDNNNNNNDNDVDG-----------------DKIVVTWDGD-DDPEN-PQNWPTLQ

A4WFG6 TNSNR----------------------------------------------------IKL

A1JSB0 NNSNR----------------------------------------------------LRL

A8GKP6 NDSNR----------------------------------------------------LRL

P57601 TNINR----------------------------------------------------IGL

Q8K942 QNINQ----------------------------------------------------IKL

Q89A60 NKSNL----------------------------------------------------IGL

P76198 MSQNKAFST----------------------------------------------PFILA

O05390 TVHAKGNVL----------------------------------------------NKIGI

O34864 -------ML----------------------------------------------DKIGI

O52717 ---MSVNNK----------------------------------------------QWYGL

O52718 ---MSINNK----------------------------------------------QWLGL

A9MJT5 -----------------------------------------------------------M

A6TG19 -----------------------------------------------------------M

A0L190 -----------------------------------------------------------M

P0AEY8 QNKLASGAR----------------------------------------------LGRQA

Q7CP73 QRIIQFFSQ-----------------------------------------------RATT

P39386 PR---FFTR-----------------------------------------------HAAT

Q68WD6 KIIVKIP-----------------------------------------------------

Q4UMJ9 KIIAKIP-----------------------------------------------------

Q1RI77 KIIAKIP-----------------------------------------------------

P32482 MSSKNFSWR----------------------------------------------YSLAA

P37597 QPGKRF------------------------------------------------------

P45123 MNQQKST---------------------------------------------------FI

P28246 TTRQHSS---------------------------------------------------FA

P31442 KRQRNV------------------------------------------------------

P37482 HPHNKKI--------------------------------------------------QS-

P76242 CSTSLSG--------------------------------------------------KNR

P17583 ------------------------------------------------------------

P0C105 --PLHTETS----------------------------------------------SQKNY

O25788 ----MQKTS----------------------------------------------N----

P11551 YRAVDKDAG----------------------------------------------QSRSY

P44776 AKVLEK----------------------------------------------------KF

A1A9E1 --MST------------------------------------------------------Y

A9MHY5 --MST------------------------------------------------------Y

A4W8S1 --MST------------------------------------------------------Y

A1JMG4 --MSA------------------------------------------------------Y

Q0TK80 SLLKT------------------------------------------------------H

O06473 TGLLS------------------------------------------------------Q

Q2FI61 YANHK------------------------------------------------------N

Q5HQE8 YSSNR------------------------------------------------------N

Q49WE5 YNSNK------------------------------------------------------N

Q4L523 KGISK------------------------------------------------------N

P33026 KSFDL------------------------------------------------------T

P31436 KILDL------------------------------------------------------T

Q9S3K0 RLPDL------------------------------------------------------T

P31675 RRMNG------------------------------------------------------V

P31126 LSLRR------------------------------------------------------S

Q58955 KLEK-------------------------------------------------------N

P02920 YLKNT------------------------------------------------------N

Q4UK37 KLYLI------------------------------------------------------G

Q1RKF6 KIYLL------------------------------------------------------G

Q4ULW4 DYRLF------------------------------------------------------E

Q1RI01 DYRLF------------------------------------------------------E

Q68WQ5 SHVCI------------------------------------------------------I

Q4UL88 SHL-L------------------------------------------------------I

Q92HQ3 SRLC-------------------------------------------------------I

Q1RIL0 SRSLS------------------------------------------------------I

Q4UMU2 ISNIF------------------------------------------------------F

Q68W71 IFNIL------------------------------------------------------F

Q1RHK8 IYNIL------------------------------------------------------F

Q1LTM2 RSLGK------------------------------------------------------Y

Q2NTK5 RSLGK------------------------------------------------------Y

P55705 RGFGP------------------------------------------------------V

Q7Z3Q1 ------MKI----------------------------------------------LF---

Q05B81 PRAWPTRSV----------------------------------------------LCRGC

P76470 ----MSTAL----------------------------------------------LDAVV

P32135 ---------------------------------------------------------MLT

P38358 YLITSTSFQ----------------------------------------------PLYGS

A4WAE6 -VAWL-------------------------------------------------------

A6T8Y8 -VAWL-------------------------------------------------------

Q6CZ44 -TAWL-------------------------------------------------------

Q888L8 -GSWL-------------------------------------------------------

Q1IB51 -GSWL-------------------------------------------------------

A6UZY0 -GSWL-------------------------------------------------------

Q4QP52 -IQSW-------------------------------------------------------

Q9CM87 -RQFA-------------------------------------------------------

O25797 -FALM-------------------------------------------------------

Q17YP7 -LALM-------------------------------------------------------

P77389 -KINY-------------------------------------------------------

O31577 -SVKF-------------------------------------------------------

O34367 -SNIP-------------------------------------------------------

A1AHK2 -PNWS-------------------------------------------------------

A9MWE8 -PNWS-------------------------------------------------------

P23910 --MKK-------------------------------------------------------

P31141 --MPL-------------------------------------------------------

P43531 -QFMR-------------------------------------------------------

Q8K902 -KFNQ-------------------------------------------------------

P57648 -KFNQ-------------------------------------------------------

Q89A23 -AFTE-------------------------------------------------------

P37498 -VFRK-------------------------------------------------------

P0A0J4 --IFV-------------------------------------------------------

Q07282 --VMM-------------------------------------------------------

P02982 --LIV-------------------------------------------------------

P02981 --LIV-------------------------------------------------------

P70187 -SVYH-------------------------------------------------------

Q5SR56 -SVYH-------------------------------------------------------

P77726 -ATWG-------------------------------------------------------

Q89AA9 -AIIG-------------------------------------------------------

Q8K999 -VTLS-------------------------------------------------------

P57538 -VTLS-------------------------------------------------------

Q5HIA2 -TVVA-------------------------------------------------------

Q4L3Q4 -TVFA-------------------------------------------------------

Q5HRH0 -TVIA-------------------------------------------------------

P0C0L7 -AITA-------------------------------------------------------

Q47421 -AITA-------------------------------------------------------

P0A2G3 GAILR-------------------------------------------------------

P16482 GAILR-------------------------------------------------------

P0AEX3 WAIVG-------------------------------------------------------

P76350 -AALG-------------------------------------------------------

P41036 RAFSA-------------------------------------------------------

Q9SYQ1 -AIIV-------------------------------------------------------

Q9S735 -AIIV-------------------------------------------------------

P76230 -MFAI-------------------------------------------------------

P38055 -IFGI-------------------------------------------------------

Q46909 -IAAL-------------------------------------------------------

P31679 -ILLW-------------------------------------------------------

O24723 -IVLM-------------------------------------------------------

O30513 -VIIL-------------------------------------------------------

Q43975 -IAIV-------------------------------------------------------

Q51955 -VVLL-------------------------------------------------------

Q9I6Q3 -IVLL-------------------------------------------------------

P77589 -TIGL-------------------------------------------------------

P94131 IAFLF-------------------------------------------------------

P71369 -ALIG-------------------------------------------------------

O34691 KLLGV-------------------------------------------------------

P0AGC0 WFKPFM------------------------------------------------------

P27669 YRYWRR------------------------------------------------------

P96335 YKRLRW------------------------------------------------------

P08194 YRRLRW------------------------------------------------------

P37948 YKRLRL------------------------------------------------------

P12681 YGRYRI------------------------------------------------------

Q5M7K3 DSCYRG------------------------------------------------------

Q9WU81 DSWFRG------------------------------------------------------

Q58CV5 --WYRA------------------------------------------------------

Q7SY29 DSWYRF------------------------------------------------------

P57057 DQWYRA------------------------------------------------------

Q17QZ3 FSHHHM------------------------------------------------------

Q3TIT8 FSHHHL------------------------------------------------------

Q640L2 YTHHHL------------------------------------------------------

Q5F3N0 CTHHHI------------------------------------------------------

Q09037 YVAVY-------------------------------------------------------

Q06222 FVAVY-------------------------------------------------------

P13865 FVGVY-------------------------------------------------------

P46499 FPIFY-------------------------------------------------------

P54219 VPIVP-------------------------------------------------------

Q6NT16 GPFFP-------------------------------------------------------

Q8R0G7 NLLNY-------------------------------------------------------

Q5XGK0 NLLNY-------------------------------------------------------

Q7ZU13 NLLNY-------------------------------------------------------

A2CER7 NLLNY-------------------------------------------------------

Q9D232 NLLNY-------------------------------------------------------

Q6ZMD2 NLLNY-------------------------------------------------------

Q6GPQ3 SSVGF-------------------------------------------------------

A5IVG9 VGFMA-------------------------------------------------------

Q5HLK7 AGFMA-------------------------------------------------------

P46907 AGFMV-------------------------------------------------------

P10903 LAFCV-------------------------------------------------------

P37593 LAFCV-------------------------------------------------------

P37758 LAFCV-------------------------------------------------------

Q9P3K8 SLCAG-------------------------------------------------------

Q6FWD4 AITSG-------------------------------------------------------

Q6CPY8 SISAG-------------------------------------------------------

P22152 LAFLS-------------------------------------------------------

Q02563 GGKGERMADGAPLAGVRGGLSDGEGPPGGRGEAQRRKDREELAQQYETILRECGHGRFQW

Q496J9 QAKDSIVSVGQP----KG----DEYKDRRELESERRADEEELAQQYELIIQECGHGRFQW

Q63564 KSKQTKMAPSRA-DGLRG---------QADLMAERMEDEEQLAHQYETIIDECGHGRFQW

Q1JP63 ----------------------------GAAVPKEFANPTDDTFMVEDAVEAIGFGKFQW

Q2XWK0 ----------------------------GTTVPKEFANPTDDTFMVEDAVEAIGFGKFQW

Q1LVS8 ----------------------------NPVEPAQVKEP--KCYTVEEAVESIGFGCFHI

O08966 WSPAEELNYTVPGLGSAGEASFLSQCMKYEV-DWNQ-----STLDCVDPLSSLAANRSHL

O15245 WSPAEELNYTVPGLGPAGEA-FLGQCRRYEV-DWNQ-----SALSCVDPLASLATNRSHL

O77504 WSPEEELNYTVPGLGATDGA-FVRQCMRYEV-DWNQ-----SSLGCVDPLASLAPNRSHL

A7MBE0 WSLAEELNYTVPGPGPE------SQCLRYEV-DWNQ-----STLGCLDPLASLATNGSPL

Q9R0W2 WSQAEELNYTVPGLGPSDEASFLSQCMRYEV-DWNQ-----STLDCVDPLSSLAADRNQL

O02713 WSLAEELNYTVPGPGPAGQA-FPRQCRRYEV-DWNQ-----STLGCVDPLAGLAANSSHL

Q8MJI6 WSPGEELNYTVPGLGAADGA-FARQCMRYEV-DWNQ-----SSPGCVDPLASLAPNRSHL

O88446 WSPEEEWNLTTPELHVPAERRGQGHCHRYLLEDTNTS----SELSC-DPLAAF-PNRS-A

O75751 WSPEEEWNRTAPASRGPEPPERRGRCQRYLLEAANDSASATSALSCADPLAAF-PNRS-A

Q9U539 LT-----NDTQI-L----------SCKQYNETQINVF----RAFT-SAP-VDTYSDR--I

O76082 WR-----NHTVP-LRLRDGREVPHSCRRYRLATIANF----SALG-LEPGRDVDLGQ--L

Q497L8 VS-AWRLEDILALRSPEHKDHIMVELQDGEIWELTRC----SRTWRENTSH-LGYEYSGY

Q86VW1 HS-NWSLEDTGALLSSGQKDYVTVQLQNGEIWELSRC----SRNKRENTSS-LGYEYTGS

Q17QN9 LS-TWQLEDIWTQFSVGREDRILVQLQDGAIWELTSC----QRFRRDDQSS-LDYEYSGQ

Q95R48 GS-SYDLP---------------THLWNLSYPENERC----SYYDVDYTEEYLNGSIPRS

Q9Y267 LSKA----EQLNLTIPQAPNGSFLTCFMYLPVPWN---------LD-SIIQFGLNDTD--

Q6A4L0 LSAA----EQLAISIPNDTAGRPESCLMFRPPPDSA-------SLE-DILSHRFNETQ--

Q9Y226 LSAA----EQLVLSVPLDTAGHPEPCLMFRPPPANA-------SLQ-DILSHRFNETQ--

Q8IVM8 DTGALSQDALLRISIPLDSNMRPEKCRRFVHPQ-WQ-------LLHLNGTFPNTSDADME

Q66J52 FTG-----PWLRALLPMTPTGEFSKCLRYTTPQ-YE-------LLENNLT-QSYDDLETE

Q91WU2 LSH---QDLWLKTHLPRETDGSFSSCLRFAYPQALP-------NVTLGTEVYNSGEPEGE

Q8IZD6 SAGEDQAFGDWLLTANGSE-----------------------------------------

Q6DFR1 YGSQ--------LPAEGE------------------------------------------

Q6NUB3 YGGQ--------LPAEGQ------------------------------------------

Q28ES4 ------------ISASKEE-----------------------------------------

A6NKX4 LRGPALLDAAIPRLGPTRAP--------------------------AEALGVLSPSYLAP

P47185 LLCYPV------------------------------------------------------

P13181 LLCLCV------------------------------------------------------

P23585 CLCLMI------------------------------------------------------

Q92339 ILVVFI------------------------------------------------------

O74969 VMLIFV------------------------------------------------------

P10870 CVGVFV------------------------------------------------------

Q12300 LVGIFV------------------------------------------------------

P42833 LLCLVI------------------------------------------------------

P49374 VIGFIA------------------------------------------------------

O74713 FIASIS------------------------------------------------------

Q9BE72 SSV-TA------------------------------------------------------

Q5J316 SSV-TA------------------------------------------------------

Q32NG5 SSV-IA------------------------------------------------------

Q6NWF1 CSVSVA------------------------------------------------------

Q0P4G6 LAATVS------------------------------------------------------

Q6GN01 LAATSS------------------------------------------------------

O95528 LCASVS------------------------------------------------------

Q8VHD6 LCASVS------------------------------------------------------

Q3UHK1 AAAAFS------------------------------------------------------

Q96QE2 VVAVFS------------------------------------------------------

Q9C757 RLAFSA------------------------------------------------------

Q8VZR6 GLTVTA------------------------------------------------------

P30606 TLTFVA------------------------------------------------------

P30605 TLTFVA------------------------------------------------------

Q10286 VLAFAA------------------------------------------------------

Q04162 -IFVGA------------------------------------------------------

Q01440 SVMLCA------------------------------------------------------

P11166 LAVGGA------------------------------------------------------

P46896 LAVGGA------------------------------------------------------

P47843 FAISIA------------------------------------------------------

P14672 LAVFSA------------------------------------------------------

Q90592 LSVFTA------------------------------------------------------

P11168 FTVITA------------------------------------------------------

Q5RB09 VASLAG------------------------------------------------------

A4ZYQ5 LATLSA------------------------------------------------------

P22732 LATLIA------------------------------------------------------

P58353 LATLIA------------------------------------------------------

Q863Y9 LATLIA------------------------------------------------------

P43427 LATFLA------------------------------------------------------

Q9WV38 LATLIA------------------------------------------------------

P15686 MVAFMA------------------------------------------------------

Q39525 LVALVA------------------------------------------------------

Q94AZ2 ISCIMA------------------------------------------------------

Q10710 VACMVA------------------------------------------------------

Q41144 VTCVVA------------------------------------------------------

P23586 FTCVVA------------------------------------------------------

O65413 VTCIVA------------------------------------------------------

Q9SX48 MTCIVA------------------------------------------------------

Q9LT15 MTCIVA------------------------------------------------------

Q9FMX3 ITCIVA------------------------------------------------------

Q9SBA7 ICVIIA------------------------------------------------------

Q9SFG0 ICVMIA------------------------------------------------------

Q8L7R8 ASCVMA------------------------------------------------------

Q93Y91 MSCIVA------------------------------------------------------

P0AE24 FVSVAA------------------------------------------------------

P0AEP1 FVCFLA------------------------------------------------------

P96710 LISCAA------------------------------------------------------

P54723 TITLVS------------------------------------------------------

O34718 TIILVS------------------------------------------------------

P46333 -IYFFG------------------------------------------------------

O52733 -VYFFG------------------------------------------------------

P0AGF4 SITLVA------------------------------------------------------

P21906 RLALIA------------------------------------------------------

P15729 LISGVA------------------------------------------------------

Q6AWX0 LPFLFP------------------------------------------------------

Q93YP9 ACVLIV------------------------------------------------------

Q94AF9 LSTFVA------------------------------------------------------

O04036 LSTFVA------------------------------------------------------

Q94KE0 FSTFVS------------------------------------------------------

Q9SCW7 LSTSVA------------------------------------------------------

Q4F7G0 LSTSVV------------------------------------------------------

Q8LBI9 FSTFVA------------------------------------------------------

P93051 LSTFVA------------------------------------------------------

Q0WQ63 LSTIIA------------------------------------------------------

Q3ECP7 LTTFVA------------------------------------------------------

Q9LTP6 FTTFTA------------------------------------------------------

Q8GXK5 LAFTVG------------------------------------------------------

Q9JJZ1 LATFAA------------------------------------------------------

P58354 LAAFAA------------------------------------------------------

Q9NY64 LAAFAA------------------------------------------------------

Q9UGQ3 LATFAA------------------------------------------------------

P43562 -ITAIL------------------------------------------------------

Q9FYG3 PHVLVA------------------------------------------------------

P36035 FTSLLHIHE----------------------------------FSWENVNPIPELRKMTW

P47186 AWSLL-------------------------------------------------------

A6QLI1 PEKKA-PLCDCTCFG--------------------------------------LPR--RY

Q5W8I7 PEKKA-PLVDCTCFG--------------------------------------LPR--RY

A4FV52 QTRDP-PVVDCTCFG--------------------------------------LPR--RY

P34644 HSNKVLQVMEQTWIG--------------------------------------KCRK-RW

Q66GI9 PEETAAELTAQPNFS------------------------------------EFITSE-RV

Q9FKV1 --------MKLSN----------------------------------------IPQ--RY

Q46916 LSQAASSVEKRTNA--------------------------------------------RY

Q91Y77 -STGN-QEPPEPPEG----------------------------------------GW-GW

Q8TF71 -ATAEPHEPPEPPEG----------------------------------------GW-GW

A1L1W9 -SEQKSPEEFEPPEG----------------------------------------GW-GW

P36021 VETRGTARGFQPPEG----------------------------------------GF-GW

O35308 RR-GA-----GPPDG----------------------------------------GW-GW

O95907 RR-GE-----GPPDG----------------------------------------GW-GW

Q90632 EE-GQLPAPVKPPDG----------------------------------------GW-GW

O35910 DE-G--PTGIKAPDG----------------------------------------GW-GW

O15427 DE-G--PTGVKAPDG----------------------------------------GW-GW

P57788 DD-G--PSGVKAPDG----------------------------------------GW-GW

O15375 -----MPQALERADG----------------------------------------SW-AW

Q6ZSM3 AR-S-----TSPPDG----------------------------------------GW-GW

Q8BGC3 VS-L-----ASPPDG----------------------------------------GW-GW

Q503M4 KG-G-----VLPPDG----------------------------------------GW-GW

O15403 KLCSKANVYTEVPDG----------------------------------------GW-GW

Q8NCK7 PQTAMTPQPAGPPDG----------------------------------------GW-GW

Q7RTY0 -----MARRTEPPDG----------------------------------------GW-GW

Q5R5M4 ------MELKKSPDG----------------------------------------GW-GW

Q7TM99 ------MEFQKSPDG----------------------------------------GW-GW

Q5ZJU0 ------MVYRKPPDG----------------------------------------GW-GW

O15374 KREGKVQPYTKTLDG----------------------------------------GW-GW

Q08777 HDSDKEEDSNEEIES------------------------------------FPEGGFKAW

Q08268 PEASLIYDDDRE---------------------------------------FPDGGLKAW

P39709 INEKERSHNKWYSWF------------------------------------KQGTSFKEK

P25621 VSSKR--------------------------------------------------AIKKR

O13880 SELRR-----------------------------------------------------LQ

P40445 TKDEE------------------------------------------------------K

P15365 VTPEE-----------------------------------------------------DR

Q07904 GSHEL-----------------------------------------------------SP

P53322 PSHPM-----------------------------------------------------EK

P32071 NKEQTEGDQATIQNEPAS-----------------EHIIVTWDGD-DDPEN-PYNWPFAW

P38124 SESSTDSDSSGSQIE-KN-----------------DPFRVDWNGP-SDPEN-PQNWPLLK

Q06451 SSPSSNEDALESDNNEKGKEGDSSGANDEAPDLDPEIEFVTF-VT-GDPEN-PHNWPAWI

P53283 ATNSSSTGKEEMEEEETEEREDQSGEN----ELDPEIEFVTF-VT-GDPEN-PHNWPSWV

Q9C0R8 DYNEADPWKYPIDSESG-------------------LRLVEW-VD-GDKHN-PKNISKAK

Q9C0Q6 DYNDADPWKYPIDKETK-------------------LRLVDW-TE-GDKHN-PKNFGKGF

Q9HF77 RTASQSSKPLPPMGGGKEYPPMLGS---------RDPYVVAFDGP-DDPDH-PHNYPTWK

Q5A0E9 QRSLLLNEPLPLMGGGRPYPPLLGS---------RDPYAVTFDGP-DDAGF-PQNFPFWK

Q07824 DRVEVDYTNCAPMGGDRPYPPSLPS---------RDLYEVTFDGP-NDPLH-PFNWPMKK

O59698 LEPPMAKLLSDPDFQGKQF-PTVEA---------PELFIFEL-AP-DSPSI-ALNWTFWR

O74829 LEESL-----------KKY-PVISN---------PQDFIVTLDGP-DDPDL-AVNWPLAK

P38776 SINSDKAAKLDLELTSERKNDG------------KQSHEVTFNEDIADPEDIARHMSTAR

Q9HDX4 DKDFKDIELLPVKS-------------------------IESKDSIDQCD--PLTWPIRI

P38227 KNQRRGLLPQL-----------------------AIIPEFKDARDYP-------PMMKKM

P38125 NSQSRDPSPDT----------------------QAHIPYTYFSKD------------QRL

P40474 QSYNRETADKLALTR-------------------TESVKPEPEITAPPH--SRFSRSFKT

O94607 LR-EELDNAGPINDRT----------SELSLEGVSKAEAIAS----------TWSKRSII

Q9HE13 TALDEANEIKSINSKAFSFKNQDQIYGAGSLKSRFSTHEISSEKNDSPDFTLVLPSNRLY

Q04301 QTLDETSNLLPPPEEA----------EAPPLEQKF--HE----------YNLALPK---F

P13090 SSRD--NNIGESLTA------------TAFTQSEDEMVDSNQKWQNPNYFKYAWQE---Y

Q08902 KNLD--TN-AEALKK------------EDKVLSEFDIQDERPK-------SLLW-E---S

P39886 ----------------------------MSTETHDEPSGVAHTP------ASGL-RGRPW

P76269 ---------------------------------------MPKVQ------ADGLPLPQRY

Q8Y9K8 ----------------------------------------MQQE------ATGGQKIRPI

P28873 KAFFIFQISFLTTSVYMGSAVYTPGIEELM------------------------------

A4WFG6 TWISFFSYALTGALVIVTGMVMGDIAN---------------------------------

A1JSB0 TWISYFSYALTGALVIVTGMVMGNIAE---------------------------------

A8GKP6 TWISYFSYALTGALVIVTGMVMGNIAE---------------------------------

P57601 TWISFLSYAFTGALVVVTGMIMGNISN---------------------------------

Q8K942 TWISFFSYAFTGALIVITGMIMGDIAD---------------------------------

Q89A60 TCISFLSYALTGALITITGIFLENISK---------------------------------

P76198 VLCIYFSYFLHGISVITLAQNMSSLAE---------------------------------

O05390 PSHMVWGYIGVVIFMVGDGLEQGWLSP---------------------------------

O34864 PKRLAWGFLGVVLFMMGDGLEQGWLSP---------------------------------

O52717 PLNLIWGYVAIAVFMTGDGFELAFLSH---------------------------------

O52718 PLNLLWGYIAIAVFMTGDGFELAFLSH---------------------------------

A9MJT5 KRFLLCSFTLILLYPAGIDMYLVGLPR---------------------------------

A6TG19 TRFLLCSFALVLLYPSGIDMYLVGLPR---------------------------------

A0L190 FRYLLCCFGLVLMYPTGIDMYLVGLPQ---------------------------------

P0AEY8 LLFPLCLVLYEFSTYIGNDMIQPGMLA---------------------------------

Q7CP73 LFFPMALILYDFAAYLTTDLIQPGIIN---------------------------------

P39386 LFFPMALILYDFAAYLSTDLIQPGIIN---------------------------------

Q68WD6 -VW--MLLSLFILSPTTETIYTSGLPS---------------------------------

Q4UMJ9 -AW--MLLCLFILSPTTETIYTSGLPS---------------------------------

Q1RI77 -AW--MLLCLFTLSPITETIYTSGLPS---------------------------------

P32482 TVL--LLSPFDLLASLGMDMYLPAVPF---------------------------------

P37597 -LV--WLAGLSVLGFLATDMYLPAFAA---------------------------------

P45123 FIL--TLGILSMLPPFGVDMYLPSFLE---------------------------------

P28246 IVF--ILGLLAMLMPLSIDMYLPALPV---------------------------------

P31442 -NLLLMLVLLVAVGQMAQTIYIPAIAD---------------------------------

P37482 FWLITGIIFIAFNLRPAITSVGPVISS---------------------------------

P76242 IVLIAGILMIATTLRVTFTGAAPLLDT---------------------------------

P17583 --MLLVLVLIGLNMRPLLTSVGPLLPQ---------------------------------

P0C105 GFALTSLTLLFFMWGFITCLNDILIPH---------------------------------

O25788 TLALGSLTALFFLMGFITVLNDILIPH---------------------------------

P11551 IIPFALLCSLFFLWAVANNLNDILLPQ---------------------------------

P44776 IVPFVLITSLFALWGFANDITNPMVAV---------------------------------

A1A9E1 TRPVMLLLSGLLLLTLAIAVLNTLVPL---------------------------------

A9MHY5 TRPVKLLLCGLLLLTLAIAVLNTLVPL---------------------------------

A4W8S1 SRPVLLLLCGLLLLTLAIAVLNTLVPL---------------------------------

A1JMG4 SRPVLLLLCGLLLFTISIAVLNTLVPL---------------------------------

Q0TK80 P-AFRAVFLARFISIVSLGLLGVAVPV---------------------------------

O06473 PKAVWAVAFACVISFMGIGLVDPILPA---------------------------------

Q2FI61 FILMLIILFLMEFARGMYILSYINF-----------------------------------

Q5HQE8 FVMMLVILFLMEFARGMYILSYINF-----------------------------------

Q49WE5 FIIMLIILFLMEFARGMYILSYLAL-----------------------------------

Q4L523 FKIMLVILFLMEFARGMYVLSYVNY-----------------------------------

P33026 STAFLIVAFLTGIAGALQTPTLSIF-----------------------------------

P31436 AAAFLLVAFLTGIAGALQTPTLSIF-----------------------------------

Q9S3K0 SSAFLVIAFLTGIAGALQLPTLSLF-----------------------------------

P31675 YAAFMLVAFMMGVAGALQAPTLSLF-----------------------------------

P31126 TSALLASSLLLTIGRGATLPFMTIY-----------------------------------

Q58955 VFVIWITTFTTMLGVGFIAPIMAIY-----------------------------------

P02920 FWMFGLFFFFYFFIMGAYFPFFPIW-----------------------------------

Q4UK37 ILLLGLISGLTFNLIFFTVPYQLSEAK---------------------------------

Q1RKF6 ILLLGLISGFTFNLIFFTVPYQLSEAR---------------------------------

Q4ULW4 IFILGIVSGMPLVIIFSTLSVWLKESG---------------------------------

Q1RI01 ILILGIVSGMPLAIIFTTISVWLKESG---------------------------------

Q68WQ5 IWLFGFISGFNIMITGNTLNYWFAKKD---------------------------------

Q4UL88 IWLFGLISGFNLMITGNTLNYWLAKED---------------------------------

Q92HQ3 IWLFGLISGFNLMITGNTLNYWLAKED---------------------------------

Q1RIL0 IWLFGLISGFNIMITGNTLNYWLAKEN---------------------------------

Q4UMU2 ILIISFPGGLIYLLTGSTLSFWLRESG---------------------------------

Q68W71 ILIISFPGGLIYLLTGSTLSFWLRESE---------------------------------

Q1RHK8 ILFISLPGGLIYLLTGSTLSFWLRESG---------------------------------

Q1LTM2 FLLMDNI-LVVMGFYMV---FPLISIR---------------------------------

Q2NTK5 FLLMDNM-LVVMGFYVV---FPLISIR---------------------------------

P55705 FALLFGLQFISMGAMEMNGPFWPIQIK---------------------------------

Q7Z3Q1 VEPAIFLSAFAMTLTGPLTTQYVYRRIWEETG-NYTF-----------------------

Q05B81 VEPLVFLANFALVLQGPVTTQYLWHRFSADLGYNGTR-----------------------

P76470 KKNRVRLIPFMLALYVLAFLDRSNIGF---------------------------------

P32135 KKKWALFSLLTLCGGTIYKLPSLKDAFYIP------------------------------

P38358 FSDALGRRNCLFFANGAFTIGCLACGF---------------------------------

A4WAE6 RVVTLAIAAFIFNTTEFVPVGLLSDI---A------------------------------

A6T8Y8 RVVTLAIAAFIFNTTEFAPVGLLSDI---A------------------------------

Q6CZ44 RVVSLSLAAFIFNTAEFAPVALLSDI---A------------------------------

Q888L8 GVFALALAAFIFNTTEFVPIGLLSNI---G------------------------------

Q1IB51 SVIALALAAFIFNTTEFVPVALLSDI---G------------------------------

A6UZY0 SVIALALAAFIFNTTEFVPVGLLSDI---G------------------------------

Q4QP52 RVLIMACAGFIFNTTEFVPVAMLSDI---A------------------------------

Q9CM87 RVITFALAGFVFNTTEFIPVALLSDI---A------------------------------

O25797 RVFVFSLSAFIFNTTEFVPVALLSDI---A------------------------------

Q17YP7 RVFVFSLSAFIFNTTEFVPIALLSDI---A------------------------------

P77389 PLLALAIGAFGIGTTEFSPMGLLPVI---A------------------------------

O31577 IIFVLMICTFSIGYTEYAVMGILTSI---A------------------------------

O34367 ALLALAVSAFAIGTTEFISVGLLPLI---A------------------------------

A1AHK2 AVFSVAFCVACLIIVEFLPVSLLTPM---A------------------------------

A9MWE8 AVFAVAFCVACLITVEFLPVSLLTPM---A------------------------------

P23910 VILSLALGTFGLGMAEFGIMGVLTEL---A------------------------------

P31141 PLYLLAVAVCAMGTSEFMLAGLVPDI---A------------------------------

P43531 VTLALFSAGLATFALLYCVQPILPVL---S------------------------------

Q8K902 IVLSLFLGGFSSFSILYCVQSILPVF---S------------------------------

P57648 VILALFSGGFATFSILYCVQSILPMF---S------------------------------

Q89A23 VTIAFFLAGFSTFSTLYCVQPILFLF---S------------------------------

P37498 ITFAFFAAGFNTFAILYCVQPLMEEF---T------------------------------

P0A0J4 LYFNIFLIFLGIGLVIPVLPVYLKDL----------------------------------

Q07282 ALVIIFLDAMGIGIIMPVLPALLREF---V------------------------------

P02982 ILSTVALDAVGIGLIMPVLPGLLRDL---V------------------------------

P02981 ILGTVTLDAVGIGLVMPVLPGLLRDI---V------------------------------

P70187 AVIVIFLEFFAWGLLTAPTLVVLHET---F------------------------------

Q5SR56 AAIVIFLEFFAWGLLTTPMLTVLHET---F------------------------------

P77726 LGTVFSLRMLGMFMVLPVLTTYGMAL---Q------------------------------

Q89AA9 IFMIFSLRVFGMFMIVPVLSTYGMCL---K------------------------------

Q8K999 FCVVFLLRMLGIFSVLPILSKYGLYL---N------------------------------

P57538 FCVIFLLRMLGMFMILPILSKYGMLL---D------------------------------

Q5HIA2 TGIGNAMEWFDFGVYAYTTAYIGANFFSPV------------------------------

Q4L3Q4 TGIGNAMEWFDFGVYAYTTAYIGANFFSPV------------------------------

Q5HRH0 TGIGNAMEWFDFGLYSYLAVIISKNFFSQV------------------------------

P0C0L7 ASLGNAMEWFDFGVYGFVAYALGKVFF-PG------------------------------

Q47421 AALGNAMEWFDFGVYGFVAYALGQVFF-PG------------------------------

P0A2G3 VTSGNFLEQFDFFLFGFYATYIARTFF-PA------------------------------

P16482 VTSGNFLEQFDFFLFGFYATYIAHTFF-PA------------------------------

P0AEX3 ASSGNLVEWFDFYVYSFCSLYFAHIFF-PS------------------------------

P76350 SFAGAVVDWYDFLLYGITAALVFNREFFPQ------------------------------

P41036 AWLGYLLDGFDFVLIALVLTEVQGEF----------------------------------

Q9SYQ1 AGMGLFTDAYDLFCIAPVMKMISHVY---Y------------------------------

Q9S735 AGMGLFTDAYDLFCIAPIMKMISQIY---Y------------------------------

P76230 VAFGLLVC-WSNAVGGLILAQLKALG---W------------------------------

P38055 ISFSLLLTGFLSYSGNVVLAKLVSNG---W------------------------------

Q46909 TFGAHLTDGYVLGVIGYAIIQLTPAM---Q------------------------------

P31679 GSGGPFLDGYVLVMIGVALEQLTPAL---K------------------------------

O24723 VFLIMVADGMDITLASHLFPPVIRDW---G------------------------------

O30513 STLIIIFDGYDLVIYGVALPLLMKEW---A------------------------------

Q43975 CFLIVFVDGIDTAAMGFIAPALAQDW---G------------------------------

Q51955 CFLIVFLDGLDTAAMGFIAPALSQEW---G------------------------------

Q9I6Q3 CFLIVFLDGLDTAAMGFIAPALTQDW---G------------------------------

P77589 CFLVALMEGLDLQAAGIAAGGIAQAF---A------------------------------

P94131 AFLALLVDGADLMLLSYSLNSIKAEF---N------------------------------

P71369 SAVGYGMDGFDLLILGFMLSAISADL---N------------------------------

O34691 AGLGWLFDAMDVGILSFIIAALHVEW---N------------------------------

P0AGC0 QSYLVVFIGYLTMYLIRKNFNIAQNDMIST------------------------------

P27669 HILITIWLGYALFYFTRKSFNAAAPEILAS------------------------------

P96335 QVFAGIFFGYAAYYFVRANFDLAQPGLIQA------------------------------

P08194 QIFLGIFFGYAAYYLVRKNFALAMPYLVEQ------------------------------

P37948 QVFIGIFIGYAGYYLLRKNFAFAIPYLQEQ------------------------------

P12681 QALLSVFLGYLAYYIVRNNFTLSTPYLKEQ------------------------------

Q5M7K3 FIIVMTFLFYTCYHMSRKPISIVKGQLHRNCSALPSPPN--ISAN---------------

Q9WU81 FILLLTFLIYACYHMSRKPISIVKSRLHQNCSEMVRPVNDTHDLN---------------

Q58CV5 FILLITFLIYTCYHMSRKPISVVKSRLHHNCSEVIQPVNSTHSLN---------------

Q7SY29 SILFLTFVFYTSYHLSRKPISIVKSQLHRNCSNVVHPANLNITDN---------------

P57057 FIFILTFLLYASFHLSRKPISIVKGELHKYCTAWDEADVRFSSQN---------------

Q17QZ3 VVFLLTFFSYSLLHASRKTFSNVKVSISKQWTPSAFNKST-ELLP---------------

Q3TIT8 AVFLLTFFSYSLLHASRKTFSNVKVSISKQWTPNAFNTSL-D-LP---------------

Q640L2 AAFLLTFFSYSLLHASRKSFSNVKVSVSSQWTPSDLNSSAYIVLP---------------

Q5F3N0 VVFLLTFFSYSLLHASRKTFSNVKVSISSQWTPSCLNSTTFELRP---------------

Q09037 LLLYLTATECK-FT-TEGACGGRKIYGCKW----SGT-TCK-FEN---------------

Q06222 ILLYEVATNCSLFK-TTEACKAVGSYGCEW----KDTEVCS-WKK---------------

P13865 STLFGYSTNCASFL-QENSCTTVPNADCKWFVSPTGSSYCG-WPEVTCRKEYAYSSPAEM

P46499 NFHPGLSCEDPGFNFSKPKCKLSKVEICT-----ELTANCSKWH----------------

P54219 TFLYDMEFKEVNSSLHLGHAGSSPHALASPAFSTIFSFFNNNTVAVEESVPSGIAWMNDT

Q6NT16 --------KEAEKK------GAS------------------NTI----------------

Q8R0G7 MDRFTVAGVLTDIEQF--------------------------------------------

Q5XGK0 MDRFTVAGVLPDIKKA--------------------------------------------

Q7ZU13 MDRFTVAGVLPDIEHF--------------------------------------------

A2CER7 MDRYTIAGVLLRIQKF--------------------------------------------

Q9D232 MNWFIIPGVLLDVQKY--------------------------------------------

Q6ZMD2 MNWFIIAGVLLDIQEV--------------------------------------------

Q6GPQ3 S--IVMTSIWPYLQKV--------------------------------------------

A5IVG9 WSIIAPLMPFI-KQD---------------------------------------------

Q5HLK7 WSIISPLMPFI-SQD---------------------------------------------

P46907 WVLISSLISQI-TLD---------------------------------------------

P10903 WMLFSAVAVNLPKVG---------------------------------------------

P37593 WMLFSAVAVNLNKIG---------------------------------------------

P37758 WMLFSAVTVNLNKIG---------------------------------------------

Q9P3K8 SITIFSMYGHIFQER---------------------------------------------

Q6FWD4 FVSLISLYAQPWQEH---------------------------------------------

Q6CPY8 LFSMISVYAVPWELR---------------------------------------------

P22152 WYAFPPLLTVTIRDD---------------------------------------------

Q02563 TLYFVLGLALMADGVEVFVVGFVLPSAEK-------------------------------

Q496J9 ALFFVLGMALMADGVEVFVVGFVLPSAET-------------------------------

Q63564 TLFFVLVLALMADGVEVFVVSFALPSAEK-------------------------------

Q1JP63 KLSVLTGLAWMADAMEMMILSILAPQLHC-------------------------------

Q2XWK0 KLSMLTGLAWMADAMEMMILSILAPQLHC-------------------------------

Q1LVS8 LLFVIMGSANIVEAMEIMLLAVVSPEIRC-------------------------------

O08966 PLS-PCEHGWVYDTPG--SSIVTEFNLVC-------------------------------

O15245 PLG-PCQDGWVYDTPG--SSIVTEFNLVC-------------------------------

O77504 PLG-PCQHGWVYDTPG--SSIVTEFNLVC-------------------------------

A7MBE0 PLG-PCEQGWVYDTPG--SSIVTEFNLVC-------------------------------

Q9R0W2 PLG-PCEHGWVYNTPG--SSIVTEFNLVC-------------------------------

O02713 PLG-PCRYGWVYDTPG--SSIVTEFDLVC-------------------------------

Q8MJI6 PLG-PCQHGWVYDTPG--SSIVTEFNLVC-------------------------------

O88446 PLV-PCSGDWRYVETH--STIVSQFDLVC-------------------------------

O75751 PLV-PCRGGWRYAQAH--STIVSEFDLVC-------------------------------

Q9U539 SLV-PCQNGWDYDNSTYLDSLVTEFNLVC-------------------------------

O76082 EQE-SCLDGWEFSQDVYLSTIVTEWNLVC-------------------------------

Q497L8 KHDSPCFDGYVYDQSKWRNSAVRNFNLVC-------------------------------

Q86VW1 KKEFPCVDGYIYDQNTWKSTAVTQWNLVC-------------------------------

Q17QN9 KSSFPCLDGYIYDRSKWLSTVVTQWDLVC-------------------------------

Q95R48 SNETKTCSSYVYDRSKYLNSAVTEWNLVC-------------------------------

Q9Y267 ----TCQDGWIYPDAKKRS-LINEFDLVC-------------------------------

Q6A4L0 ----ACDSGWDYPENRPQS-LKKEFDLVC-------------------------------

Q9Y226 ----PCDMGWEYPENRLPS-LKNEFNLVC-------------------------------

Q8IVM8 ----PCVDGWVYDRISFSSTIVTEWDLVC-------------------------------

Q66J52 ----PCLDGWVYDHSEFASTIITQWDLVC-------------------------------

Q91WU2 PLTVPCSQGWEYDRSEFSSTIATEWDLVC-------------------------------

Q8IZD6 ------IHKHVHFSSSF-TSIASEWFLIA-------------------------------

Q6DFR1 -------HGHVVFDGNF-TSIVSEWFLVG-------------------------------

Q6NUB3 -------HQYLVFDGNF-TSIVSEWFLVG-------------------------------

Q28ES4 ------YTKHLHDTNNF-TSIVSEWHLIK-------------------------------

A6NKX4 LTRAPRPSSWASCSG---AAAGPTWNLVC-------------------------------

P47185 SFGG-FLPGWDSGITAG--FINMDNFKMNFG-S---------------------------

P13181 AFGG-FMFGWDTGTISG--FVVQTDFLRRFG-M---------------------------

P23585 AFGG-FVFGWDTGTISG--FVNQTDFKRRFG-Q---------------------------

Q92339 SMSG-WLQGADTGSISG--ILGMRDFQSRFADR---------------------------

O74969 SMAG-WMFGADTGSIGG--VTSMRDFRERYADR---------------------------

P10870 AVGG-FLFGYDTGLINS--ITSMNYVKSHVA-----------------------------

Q12300 AVGG-FLFGYDTGLINS--ITDMPYVKTYIA-----------------------------

P42833 SLGG-FIFGWDIGTIGG--MTNMVSFQEKFGTT---------------------------

P49374 CISG-LMFGFDIASMSS--MIGTDVYKDYFS-----------------------------

O74713 TIAG-MMFGFDISSMSA--FIGAEHYMRYFN-----------------------------

Q9BE72 AVSG-LLVGYELGIISG--ALLQIK----TL-----------------------------

Q5J316 AVSG-FLVGYELGIISG--ALLQIR----TL-----------------------------

Q32NG5 AISG-LLVGYELGIISG--ALLQLQ----SL-----------------------------

Q6NWF1 CLSG-LLMGYEMSLISG--ALLQLR----DV-----------------------------

Q0P4G6 LLGG-IVFGYELGIISG--ALLVLK----TV-----------------------------

Q6GN01 LLGG-LIFGYELGIISG--ALLMLK----TV-----------------------------

O95528 LLGG-LTFGYELAVISG--ALLPLQ----LD-----------------------------

Q8VHD6 LLGG-LTFGYELAVISG--ALLPLQ----LN-----------------------------

Q3UHK1 ALGG-FLFGYDTGVVSG--AMLLLR----RQ-----------------------------

Q96QE2 ALGG-FLFGYDTGVVSG--AMLLLK----RQ-----------------------------

Q9C757 GIGG-LLFGYDTGVISG--ALLYIR-DDFKS-----------------------------

Q8VZR6 GIGG-LLFGYDTGVISG--ALLYIK-DDFEV-----------------------------

P30606 SISG-FMFGYDTGYISS--ALISIN-RDLDN-----------------------------

P30605 SISG-FMFGYDTGYISS--ALISIG-TDLDH-----------------------------

Q10286 GIGG-LLFGYDTGVISG--ALVVIG-TSLGG-----------------------------

Q04162 TIGG-LLFGYDTGVISG--VLLSLKPEDLSL-----------------------------

Q01440 ALGG-FLFGYDTGVINA--ALFQMK--DHFG-----------------------------

P11166 VLGS-LQFGYNTGV-----INAPQKVIEEFYNQ---------------------------

P46896 VLGS-LQFGYNTGV-----INRPQKVIEDFYNH---------------------------

P47843 TIGS-FQFGYNTGV-----INAPEAIIKDFLNY---------------------------

P14672 VLGS-LQFGYNIGV-----INAPQKVIEQSYNE---------------------------

Q90592 VLGF-FQYGYSLGV-----INAPQKVIEAHYGRMLGAIPM-VRHATNTSRDNATITVTIP

P11168 VLGS-FQFGYDIGV-----INAPQQVIISHYRHVLG-VPLDDRKAINNYVINS--TDELP

Q5RB09 AFGSSFLYGYNLSV-----VNAPTPYIKAFYNE---------------------------

A4ZYQ5 AFGSAFQYGYNLSV-----VNTPHKVFKSFYNE---------------------------

P22732 AFGSSFQYGYNVAA-----VNSPALLMQQFYNE---------------------------

P58353 AFGSSFQYGYNVAA-----INSPSEFMKDFYNY---------------------------

Q863Y9 AFGSSFQYGYNVAA-----VNSPAELMKAFYNE---------------------------

P43427 AFGSSFQYGYNVAA-----VNSPSEFMQQFYND---------------------------

Q9WV38 AFGSSFQYGYNVAA-----VNSPSEFMQQFYND---------------------------

P15686 ACGG-LLLGYDNGVTGG--VVSLEAFEKKFFPD---------------------------

Q39525 ACGG-MLLGYDNGVTGG--VASMEQFERKFFPD---------------------------

Q94AZ2 ATGG-LMFGYDVGVSGG--VTSMPDFLEKFFPV---------------------------

Q10710 AVGG-SIFGYDIGISGG--VISMDAFLEKFFRS---------------------------

Q41144 AMGG-LIFGYDIGISGG--VTSMDSFLKKFFPS---------------------------

P23586 AMGG-LIFGYDIGISGG--VTSMPSFLKRFFPS---------------------------

O65413 AMGG-LIFGYDIGISGG--VTTMDSFQQKFFPS---------------------------

Q9SX48 AMGG-LLFGYDLGISGG--VTSMEEFLSKFFPE---------------------------

Q9LT15 AMGG-LLFGYDLGISGG--VTSMEEFLTKFFPQ---------------------------

Q9FMX3 AMGG-LLFGYDIGISGG--VISMEDFLTKFFPD---------------------------

Q9SBA7 AVGG-LIFGYDIGISGG--VTAMDDFLKEFFPS---------------------------

Q9SFG0 AVGG-LIFGYDIGISGG--VSAMDDFLKEFFPA---------------------------

Q8L7R8 AMGG-VIFGYDIGVSGG--VMSMGPFLKRFFPK---------------------------

Q93Y91 ASCG-LIFGYDIGISGG--VTTMKPFLEKFFPS---------------------------

P0AE24 AVAG-LLFGLDIGVIAG--ALPFITD----------------------------------

P0AEP1 ALAG-LLFGLDIGVIAG--ALPFIAD----------------------------------

P96710 GLGG-LLYGYDTAVISG--AIGFLKD----------------------------------

P54723 TFGG-LLFGYDTGVING--ALPFMATAG--------------------------------

O34718 TFGG-LLFGYDTGVLNG--ALPYMGEPD--------------------------------

P46333 ALGG-LLYGYDTGVISG--ALLFINN----------------------------------

O52733 ALGG-LLFGYDTGVISG--AILFIQK----------------------------------

P0AGF4 TLGG-LLFGYDTAVISG--TVESLNTVFVAPQN---------------------------

P21906 AIGG-LLFGYDSAVIAA--IGTPVDIHFIAPRH---------------------------

P15729 ALGG-FLFGFDTAVING--AVAALQKHF--------------------------------

Q6AWX0 ALGG-LLYGYEIGATSC--ATISLQSPSLSGIS---------------------------

Q93YP9 ALGP-IQFGFTCGYSSP--TQAAITK----------------------------------

Q94AF9 VCSA-FSYGCAAGYTSG--AETAIMK----------------------------------

O04036 VSGS-FCTGCGVGFSSG--AQAGITK----------------------------------

Q94KE0 VCGS-FCFGCAAGYSSV--AQTGIIN----------------------------------

Q9SCW7 VTGS-FVYGCAMSYSSP--AQSKIME----------------------------------

Q4F7G0 VAGS-FCYGCAMSYSSP--AQSKIME----------------------------------

Q8LBI9 VCGS-FEFGSCVGYSAP--TQSSIRQ----------------------------------

P93051 VCGS-FAFGSCAGYSSP--AQAAIRN----------------------------------

Q0WQ63 VCGS-YEFGTCVGYSAP--TQFGIME----------------------------------

Q3ECP7 VSGS-FVFGSAIGYSSP--VQSDLTK----------------------------------

Q9LTP6 LCGT-FSYGTAAGFTSP--AQTGIMA----------------------------------

Q8GXK5 SCGA-LSFGCIVGYTAP--TQSSIMK----------------------------------

Q9JJZ1 ALGP-LSFGFALGYSSP--AIPSLRRTAPP------------------------------

P58354 ALGP-LSFGFALGYSSP--AIPSLRRAAPP------------------------------

Q9NY64 ALGP-LSFGFALGYSSP--AIPSLQRAAPP------------------------------

Q9UGQ3 VLGN-FSFGYALVYTSP--VIPALERSLDP------------------------------

P43562 TLVG-FIMGLEVPSLAT--FLTNKTFNEYF------------------------------

Q9FYG3 TISS-FLFGYHLGVVNE--PLESISSDLGF------------------------------

P36035 QNWNYFFMGYFAWLSAAWAFFCVSVSVAPLAEL---------------------------

P47186 VSTTLIMEGYDTAILGA--FYALPIFQRKFGSQ---------------------------

A6QLI1 IIAIMSGLGFCISFGIRCNLGVAIVDMVNNSTI---------------------------

Q5W8I7 IIAIMSGLGFCISFGIRCNLGVAIVDMVNNSTI---------------------------

A4FV52 IIAIMSGLGFCISFGIRCNLGVAIVSMVNNSTT---------------------------

P34644 LLAILANMGFMISFGIRCNFGAAKTHMYKNYTD---------------------------

Q66GI9 KVVAMLALALALCNADRVVMSVAIVPLS----L---------------------------

Q9FKV1 VIVFLTFLSTCVCYIERVGFSIAYTVAADAAGI---------------------------

Q46916 WIVVMLFIVTSFNYGDRATLSIAGSEMAKDIGL---------------------------

Q91Y77 LVMLAAMWCNGSVFGIQNAYGVLFVSMLETFGA---------------------------

Q8TF71 LVMLAAMWCNGSVFGIQNACGVLFVSMLETFGS---------------------------

A1L1W9 VVMLASMWCNGSVFGIQNAFGIMFVYLLNEFGS---------------------------

P36021 VVVFAATWCNGSIFGIHNSVGILYSMLLEE-EK---------------------------

O35308 VVLGACFVVTGFAYGFPKAVSVFFRELKRDFGA---------------------------

O95907 VVLGACFVVTGFAYGFPKAVSVFFRALMRDFDA---------------------------

Q90632 IVLFGCFVITGFSYAFPKAVSVYFKELMKDFHV---------------------------

O35910 AVLFGCFIITGFSYAFPKAVSVFFKELMHEFGI---------------------------

O15427 AVLFGCFVITGFSYAFPKAVSVFFKELIQEFGI---------------------------

P57788 AVLFGCFIITGFSYAFPKAVSVFFKELIREFGV---------------------------

O15375 VVLLATMVTQGLTLGFPTCIGIFFTELQWEFQA---------------------------

Q6ZSM3 MIVAGCFLVTICTRAVTRCISIFFVEFQTYFTQ---------------------------

Q8BGC3 MIVAGCFLVTICTRAVTRCISIFFVEFQTYFAQ---------------------------

Q503M4 MIVAGCFVVTVCTRAVTRCISIFFVEFQMHFAK---------------------------

O15403 AVAVSFFFVEVFTYGIIKTFGVFFNDLMDSFNE---------------------------

Q8NCK7 VVAAAAFAINGLSYGLLRSLGLAFPDLAEHFDR---------------------------

Q7RTY0 VVVLSAFFQSALVFGVLRSFGVFFVEFVAAFEE---------------------------

Q5R5M4 VIVFVSFFTQFLCYGSPLAVGVLYIEWLDAFGE---------------------------

Q7TM99 VIVVVSFFTQFLSYGSPLAVGVLYVEWLDAFGE---------------------------

Q5ZJU0 VIVIVSFFTQFLCYGSPLAVGVLYLEWLDAFGE---------------------------

O15374 MIVIHFFLVNVFVMGMTKTFAIFFVVFQEEFEG---------------------------

Q08777 VVTFGCFLGLIACFGLLNSTGVIESHLQDNQLS---------------------------

Q08268 LVVFGAFMGLVPVFGLINSLGAIESYISKHQLA---------------------------

P39709 KLLIKLDVLLAFYSCIAYWVKYLDTVNINNAYV---------------------------

P25621 LLLFKIDLFVLSFVCLQYWINYVDRVGFTNAYI---------------------------

O13880 KLRLKMDLRIIPCLWILYFLSCCLRFTVSLSFT---------------------------

P40445 KLVRKMDLKIFLWVFIMFAFLDLIRKNIARAVS---------------------------

P15365 KLRWKIDYCMFPLMCILYAVQFMDKISTSSAAV---------------------------

Q07904 KVLRKVDLFILPFLCCTYLLMFLDKALLNYAAS---------------------------

P53322 RVLRKMDIYLIPLMGMLYFLSNLDKSNIGNAEV---------------------------

P32071 KAIAAMQIGFLTVSVYMASAIYTPGVEEIM------------------------------

P38124 KSLVVFQIMLLTCVTYMGSSIYTPGQEYIQ------------------------------

Q06451 RWSYTVLLSILVICVAYGSACISGGLGTVE------------------------------

P53283 RWSYTVLLSILVICVAYGSACISGGLGTVE------------------------------

Q9C0R8 KWLYTLVLGAICFVVALGSAIVTGDMERPA------------------------------

Q9C0Q6 KWLCTVLLGMICFVVALGSAIVTGDLERPA------------------------------

Q9HF77 KILYCASVGLAALSVSMGSAMFSQASADIM------------------------------

Q5A0E9 KMVYSLGPLFTALSVSLGSAMFSQASPEIM------------------------------

Q07824 KVLLCLVLCLDSIAIAMCSSIFASAVPQIC------------------------------

O59698 KMKTTSIYAYASLTIAWGSSVLSPASATLA------------------------------

O74829 KLRNVAVMGSACLCAGFGSSIFSGAVPEVM------------------------------

P38776 RYYISSLITFTSMVITMISSSWTLPSTHII------------------------------

Q9HDX4 RIINTIIISFMTMLCMYGSSVFMPSIPELC------------------------------

P38227 IVF-LIAFSSMMGPMGTSIIFPAINSITTEFKT---------------------------

P38125 IIFGIIIFIGFLGPMSGNIYIPALPLLQREYDV---------------------------

P40474 VLIAQCAFTGFFSTIAGAIYYPVLSVIERKFDI---------------------------

O94607 VAYLGLYLLSFASSLEQQTTYSLQRYATSNF-----------------------------

Q9HE13 IVIPGLMLSIFLAALDQTVITTAIPTIVANL-----------------------------

Q04301 PILFSLWLGSFLSSLDSTIVANIMNRVAEEF-----------------------------

P13090 LFIFTCMISQLLNQAGTTQTLSIMNILSDSFG----------------------------

Q08902 AFVGVLCSAQLMTQAGLGQSLAPLHIIGNSFGT---------------------------

P39886 PTLLAVAVGVMMVALDSTIVAIANPAIQQDLHA---------------------------

P76269 GAILTIVIGISMAVLDGAIANVALPTIATDLHA---------------------------

Q8Y9K8 PIIASFLMAGFIGLFSETALNMALSDLIQVFDI---------------------------

P28873 ----------------------HDFGIGRVVATL-PLTLFVIGYGVGPLV-FSPMSENAI

A4WFG6 ---------------------YF---QLPVSSMSNTFTFLNAGILISIFL-NAWLME--I

A1JSB0 ---------------------YF---NLPIASMSNTFTFLNAGILISIFL-NAWLME--I

A8GKP6 ---------------------YF---NLPVSSMSNTFTFLNAGILISIFL-NAWLME--I

P57601 ---------------------YF---HLSISQMSNIFTFLNAGILVSIFI-NSWLIE--I

Q8K942 ---------------------YF---NLSVSEMSNIFTFLNAGILISIFL-NSWLID--L

Q89A60 ---------------------YF---NIPITDMGNTFTFLNAGILSSIFI-SSWITN--I

P76198 ---------------------KF---STDNAGIAYLISGIGLGRLISILF-FGVISD--K

O05390 ---------------------FLVDHGLSMQQSASLFTMYGIAVTISAWL-SGTFVQ--T

O34864 ---------------------FLIENGLTVQQSASIFSIYGIALAIASWF-SGVCLE--A

O52717 ---------------------YIKALGFTPAQASFAFTLYGLAAALSAWV-SGVVAE--I

O52718 ---------------------YIKALGFSPAEASFAFTLYGLAAALSAWI-SGVVAE--I

A9MJT5 ---------------------IAADLNASEAQLHIAFSVYLAGMATAMLF-AGKIAD--R

A6TG19 ---------------------IAQDLGASEAQLHIAFSVYLAGMASAMLF-AGRIAD--R

A0L190 ---------------------IANQLGATEAQLHIAFSVYLAGMATTMLF-AGSLAD--R

P0AEY8 ---------------------VVEQYQAGIDWVPTSMTAYLAGGMFLQWL-LGPLSD--R

Q7CP73 ---------------------VVRDFNADVSLAPASVSLYLAGGMALQWL-LGPLSD--R

P39386 ---------------------VVRDFNADVSLAPAAVSLYLAGGMALQWL-LGPLSD--R

Q68WD6 ---------------------LTKYFSIDGCITQITSTLYFLGFAVGILS-LGRLSD--I

Q4UMJ9 ---------------------LTKYFGIDGGITQTTSTLYFLGFALGILT-LGRLSD--I

Q1RI77 ---------------------ITEYFNTDGSTTQITSSLYYLGFALGILT-LGRLSD--I

P32482 ---------------------MPNALGTTASTIQLTLTTYLVMIGAGQLL-FGPLSD--R

P37597 ---------------------IQADLQTPASAVSASLSLFLAGFAAAQLL-WGPLSD--R

P45123 ---------------------IAKDLDVSPEQVQHTLTSFAYGMAFGQLF-WGPFGD--S

P28246 ---------------------ISAQFGVPAGSTQMTLSTYILGFALGQLI-YGPMAD--S

P31442 ---------------------MARDLNVREGAVQSVMGAYLLTYGVSQLF-YGPISD--R

P37482 ---------------------IRAELHMSNGAAGFLTALPLLSFAVLSPL-APKLGQ--R

P76242 ---------------------IRSAYSLTTAQTGLLTTLPLLAFALISPL-AAPVAR--R

P17583 ---------------------LRQASGMSFSVAALLTALPVVTMGGLALA-GSWLHQ--H

P0C105 ---------------------LKNVFQLNYTQSMLIQFCFFGAYFIVSLP-AGQLVK--R

O25788 ---------------------LKPIFDLTYFEASLIQFCFFGAYFIMGGV-FGNVIS--K

P11551 ---------------------FQQAFTLTNFQAGLIQSAFYFGYFIIPIP-AGILMK--K

P44776 ---------------------FQTVMEIPASEAALVQLAFYGGYGTMAIP-AALFAS--R

A1A9E1 ---------------------WLAQEHMSTWQVGVVSSSYFTGNLVGTLL-TGYVIK--R

A9MHY5 ---------------------WLAQASLPTWQVGMVSSSYFTGNLVGTLF-TGYLIK--R

A4W8S1 ---------------------WLAHENLPTWQVGMVSSSYFTGNLLGTLL-TGKLIK--R

A1JMG4 ---------------------WLSHQQLPTWQVGMVSSSYFSGNLVGTLI-AGRIIQ--Q

Q0TK80 ---------------------QIQMMTHSTWQVGLSVTLTGGAMFVGLMV-GGVLAD--R

O06473 ---------------------IAAQLHASPSEVSLLFTSYLLVTGFMMFF-SGAISS--R

Q2FI61 ---------------------LPTVTSIAVAITSLAFSIHFIADASTNFV-IGFLLK--K

Q5HQE8 ---------------------LPTVTSIAIAITSFAFSIHFIADAATNFV-IGFLLK--K

Q49WE5 ---------------------LPTATSIAVGITSIAISIHFIADATTNFV-IGFLLK--R

Q4L523 ---------------------LPTVTSIAVAVTSAALSIHFISDAATNFV-IGFLLK--K

P33026 ---------------------LTDEVHARPAMVGFFFTGSAVIGILVSQF-LAGRSD--K

P31436 ---------------------LADELKARPIMVGFFFTGSAIMGILVSQF-LARHSD--K

Q9S3K0 ---------------------LSTEVQVRPFMVGLFYTGSAVIGIVVSQI-LATYSD--R

P31675 ---------------------LSREVGAQPFWIGLFYTVNAIAGIGVSLW-LAKRSD--S

P31126 ---------------------LSRQYSLSVDLIGYAMTIALTIGVVFSLG-FGILAD--K

Q58955 ---------------------AQTLG-ATNLEIGLIFGSFALARTVAQIP-VGVLSD--I

P02920 ---------------------LHDINHISKSDTGIIFAAISLFSLLFQPL-FGLLSD--K

Q4UK37 ---------------------YTTDIIGSISLAAFPYCLKVIWSPFIDKYSIPFLCS--K

Q1RKF6 ---------------------YTTDIIGLISLAAFPYCLKVVWSPFIDKYSIPFLCS--K

Q4ULW4 ---------------------IDIAVITTFAVARLSYSLKVFWSPLVDNFKIPFL-S--R

Q1RI01 ---------------------VDIAVITTFGIAKLSYSLKVFWSPLIDNFKVPFL-S--R

Q68WQ5 ---------------------IALQTIGILSFITLPYSINFLLAPVFDTVQIKCLNK--I

Q4UL88 ---------------------IALQTIGILSFITLPYSINFLLAPIFDAVQIKYLNK--I

Q92HQ3 ---------------------IALQTIGILSFITLPYSINFLLAPIFDAVQIKYLNK--I

Q1RIL0 ---------------------IALQTIGLLSLITLPYSINFLFAPIFDSLKIKYLDK--I

Q4UMU2 ---------------------FDKITIGLFSLVNFIHIFKFLWGPLLEKVSFAPLSK--R

Q68W71 ---------------------FDKITIGLFGLVNFIYILKFLWGPLLEKISFSTLSN--R

Q1RHK8 ---------------------FDKITIGLFSLVNFIHIFKFLWGPLLEKISFIPSNS--R

Q1LTM2 ---------------------FVDQLGWTALLVGIALGLRQFIQQGL-GIFGGAFAD--K

Q2NTK5 ---------------------FVDQLGWAALLVGIALGLRQFIQQGL-GIFGGAFAD--R

P55705 ---------------------ALSPSDSVFGLAGIGVYVCPMLGVSLTSAFWGRMGD--R

Q7Z3Q1 -SSDSNISECEKNKSSPIFAFQEEVQKKVSRFNLQMDISGLIPGLVSTFI-LLSISD--H

Q05B81 -HRDS----CSNHSVDPI---AQEVETLTSHWTLYMNVGGFLVGLFSSTL-LGAWSD--C

P76470 ---------------------AKQTYQIDTGLSNEAYALGAGIFFVVYAF-LGVPAN--L

P32135 ---------------------MQEYFHLTNGQIGNAMSVNSFVTTVGFFL-SIYFAD--K

P38358 ---------------------SKNIYMLSFMRALTGIGGGGLITLSTIVNSDVIPSS--K

A4WAE6 -----------------------QSFQMETAQVGIMLTIYAWVVALMSLP-FMLLTSQM-

A6T8Y8 -----------------------DSFGMETAQVGMMLTIYAWVVALMSLP-FMLLTSKV-

Q6CZ44 -----------------------ASFSMSAAQVGLIITIYAWVVGLMSLP-CMLLSSDM-

Q888L8 -----------------------QSFDMTPAQVGLMLTIYAWVVSLMSLP-MMLATRNI-

Q1IB51 -----------------------RSFDMTTAQVGLMLTIYAWVVALASLP-MMLMTRNI-

A6UZY0 -----------------------HSFDMPTSQVGLMLTIYAWVVSLASLP-MMLLTRNI-

Q4QP52 -----------------------QSFDMQTADTGLMMTVYAWTVLIMSLP-AMLATGNM-

Q9CM87 -----------------------QSFAMPVSQTGLIITVYAWVVSLMSLP-FMLLTAKA-

O25797 -----------------------KSFEMESATVGLMITAYAWVVSLGSLP-LMLLSAKI-

Q17YP7 -----------------------KSFEMESASVGLMITLYAWIVSLGSLP-LMLLSAKI-

P77389 -----------------------RGVDVSIPAAGMLISAYAVGVMVGAPL-MTLLLSHR-

O31577 -----------------------NDFHIQVSSAGLLVTAYAASVCLTGPL-VTIISVKL-

O34367 -----------------------DDLDIPVTTAGLTVSLYALGVTFGAPI-LTSLTSSM-

A1AHK2 -----------------------QDLGISEGVAGQSVTVTAFVAMFASLF-ITQTIQAT-

A9MWE8 -----------------------QDLGISEGVAGQSVTVTAFVAMFSSLF-ITQIIQAT-

P23910 -----------------------HNVGISIPAAGHMISYYALGVVVGAPI-IALFSSRY-

P31141 -----------------------SDLGVTVGTAGTLTSAFATGMIVGAPL-VAALARTW-

P43531 -----------------------QEFGLTPANSSISLSISTAMLAIGLLF-TGPLSDAI-

Q8K902 -----------------------KQFCLTATESSLSLSAATATMSIGTLF-IGPLSDRI-

P57648 -----------------------KQFYLTPAESSLALSAATITMSLGMLF-TGPLSDII-

Q89A23 -----------------------KEFSLNPAQSSLSLSASTAMMAFGMLF-TGPLSDSI-

P37498 -----------------------REFHVTPTAASLSLSVTTMLLAVSMLV-FGSLSEVW-

P0A0J4 --------------------------GLTGSDLGLLVAAFALSQMIISPF-GGTLADKL-

Q07282 -----------------------GK-ANVAENYGVLLALYAMMQVIFAPL-LGRWSDRI-

P02982 -----------------------HS-NDVTAHYGILLALYALMQFACAPV-LGALSDRF-

P02981 -----------------------HS-DSIASHYGVLLALYALMQFLCAPV-LGALSDRF-

P70187 -----------------------PK-HTFLMN-GLIQGVKGLLSFLSAPL-IGALSDVW-

Q5SR56 -----------------------SQ-HTFLMN-GLIQGVKGLLSFLSAPL-IGALSDVW-

P77726 -----------------------GA-SEALI--GIAIGIYGLTQAVFQIP-FGLLSDRI-

Q89AA9 -----------------------NS-NIFLV--GVAIGIYGIFQIIFQIP-YGWLSDKY-

Q8K999 -----------------------GG-NKFLI--GLAVGIYGATQIVFQIP-FGILSDRF-

P57538 -----------------------GG-NKFLI--GLSMGIYGISQVIFQIP-FGILSDKF-

Q5HIA2 -----------------------ENADIRQMLTFAALAIAFLLRPIGGVV-FGIIGDKY-

Q4L3Q4 -----------------------QNPEIQQIFTFAALAIAFLLRPIGGIV-FGIIGDKY-

Q5HRH0 -----------------------DNDQLKLVFTFATFAIAFLLRPIGGIV-FGIIGDKY-

P0C0L7 -----------------------ADPSVQMVAALATFSVPFLIRPLGGLF-FGMLGDKY-

Q47421 ------------------------DPGVQSIAALATFSVPFLM-PLGGVF-FGALGDKY-

P0A2G3 -----------------------ESEFASLMLTFAVFGSGFLMRPVGAIV-LGAYIDRI-

P16482 -----------------------SSEFASLMMTFAVFGAGFLMRPIGAIV-LGAYIDKV-

P0AEX3 -----------------------GNTTTQLLQTAGVFAAGFLMRPIGGWL-FGRIADKH-

P76350 -----------------------VSPAMGTLAAFATFGVGFLFRPLGGVI-FGHFGDRL-

P41036 --------------------------GLTTVQAASLISAAFISRWFGGLM-LGAMGDRY-

Q9SYQ1 -----------------------NGDSINTAVLSTSYAIALLGTATGQLV-FGYLGDRV-

Q9S735 -----------------------HKDSIGTALLSTSYAIALLGTALGQLI-FGYLGDRV-

P76230 ---------------------------TDNSTTATFSAITTAGMFLGALV-GGIIGDKT-

P38055 ---------------------------SNNFLNAAFTSALMFGYFIGSLT-GGFIGDYF-

Q46909 ---------------------------LTPFMAGMIGGSALLGLFLGSLV-LGWISDHI-

P31679 ---------------------------LDADWIGLLGAGTLAGLFVGTSL-FGYISDKV-

O24723 ---------------------------VPVSAVTLVVSLGVVAMAIGALV-SGPVADRW-

O30513 ---------------------------IDPVTAGFIGSIALFGMMFGALI-FGTIADKLE

Q43975 ---------------------------VDRSQLGPVMSAALGGMIIGALV-SGPTADRF-

Q51955 ---------------------------IDRASLGPVMSAALIGMVFGALG-SGPLADRF-

Q9I6Q3 ---------------------------IDRASLGPVMSAALIGMVFGALG-SGPLADRY-

P77589 ---------------------------LDKMQMGWIFSAGILGLLPGALV-GGMLADRY-

P94131 ---------------------------LSTVEAGMLGSFTLAGMAIGGIF-GGWACDRF-

P71369 ---------------------------LTPAQGGSLVTWTLIGAVFGGIL-FGALSDKY-

O34691 ---------------------------LSPEEMKWIGSVNSIGMAAGAFL-FGLLADRI-

P0AGC0 -------------------------YGLSMTQLGMIGLGFSITYGVGKTL-VSYYADG--

P27669 -------------------------GILTRSDIGLLATLFYITYGVSKFV-SGIVSDR--

P96335 -------------------------GLYSKAELGVIGSAAGLAYGLSKFV-MAGMSDR--

P08194 -------------------------G-FSRGDLGFALSGISIAYGFSKFI-MGSVSDR--

P37948 -------------------------G-FSKTELGLVLAAVSIAYGFSKFI-MGMVSDR--

P12681 -------------------------LDLSATQIGLLSSCMLIAYGISKGV-MSSLADK--

Q5M7K3 -------------DTTWCSWAPFENSNYKEL-LGSLDTAFLVSYAIGMFF-SGIFGER--

Q9WU81 -------------DTTWCSWSPFDKDDYKEL-LGAVDNAFLVAYAIGMFI-SGIFGER--

Q58CV5 -------------DTTWCNWAPFDKSNYKEL-LGAVDNAFLVAYAIGMFI-SGIFGER--

Q7SY29 -------------D-TWCDWVPFDGKNYQNL-FGVLDNCFLVAYAVGMFF-SGIFGER--

P57057 RKSGSAAPHQLPDNETDCGWAPFDKNNYQQL-LGALDYSFLCAYAVGMYL-SGIIGER--

Q17QZ3 -------------VEIWSSNHLFPSAEEATLFLGMLDTIFLFSYAVGLFI-SGIVGDR--

Q3TIT8 -------------AEIWSSNHLFPSTEEATLFLGTLDTVFLFSYAVGLFI-SGIIGDR--

Q640L2 -------------NETWNGNTLFPNTKSATLFLGLLDTIFLFAYAVGLFI-SGIIGDR--

Q5F3N0 -------------NELWNSNHLFPNAEEATLFLGTLDTIFLFSYAVGLFV-SGIVGDR--

Q09037 ----PKCSEGSD-----PSDSCKNEVAYTSVYSGIFACAMIVGSMVGSII-AGKCIT--T

Q06222 -----ECDSDSD-----GVNPCESLIGYSSLYSGIFASAMIVGSMVGSII-AGKCIT--M

P13865 PGALARCEADSRCRWSYSDEECQNPSGYSSSESGIFAGSMIAGCLIGSVF-AGPLAS--K

P46499 ---IEPAPFHS------MVQDFKMFCGTKAYDSAWIATIQFIGALVGALV-YGHLGD--H

P54219 ASTIPPPATEAISAHKNNCLQGTGFLEEEITRVGVLFASKAVMQLLVNPF-VGPLTN--R

Q6NT16 --------------------------------IGMIFGCFALFELLASLV-FGNYLV--H

Q8R0G7 ------------------------FNIGDGS-TGLIQTVFISSYMVLAPV-FGYLGD--R

Q5XGK0 ------------------------FNISDSN-SGLVQTVFICSYMFLAPV-FGYLGD--R

Q7ZU13 ------------------------FGIGDGT-SGLLQTVFICSYMFLAPL-FGYLGD--R

A2CER7 ------------------------FFISDST-SGLLQTVFICSFMFLAPV-FGYLGD--R

Q9D232 ------------------------FHISDSH-AGLLQTVFISCLLVSAPV-FGYLGD--R

Q6ZMD2 ------------------------FQISDNH-AGLLQTVFVSCLLLSAPV-FGYLGD--R

Q6GPQ3 ------------------------DQSADASFLGWVIASFSLGQMVASPL-FGLWSN--H

A5IVG9 -------------------------VNVTEGQISIILAIPVILGSVLRVP-FGYLTN--I

Q5HLK7 -------------------------VDISPGQISVILAIPVILGSVLRVP-FGYLTN--I

P46907 -------------------------IHLSKGEISLVTAIPVILGSLLRIP-LGYLTN--R

P10903 -------------------------FNFTTDQLFMLTALPSVSGALLRVP-YSFMVP--I

P37593 -------------------------FNFTTDQLFLLTALPSLSGAILRVP-YSFMVP--L

P37758 -------------------------FNFTTDQLFLLTALPSVSGALLRVP-YSFMVP--I

Q9P3K8 -------------------------LHYTQFEVNGLSSAASFATYMPVPL-LGYMCD--R

Q6FWD4 -------------------------LSYSSWQINMIVTVINMGMYLTPPI-LGIIAD--I

Q6CPY8 -------------------------LGYSSLDVNLLYAAGNLGAYLTPPL-LGILSD--S

P22152 -------------------------LDMSQTQIANSNIIALLATLLVRLI-CGPLCD--R

Q02563 ------------------------DMCLSDSNKGMLGLIVYLGMMVGAFL-WGGLADR--

Q496J9 ------------------------DLCIPNSGSGWLGSIVYLGMMVGAFF-WGGLADK--

Q63564 ------------------------DMCLSSSKKGMLGLIVYLGMMAGAFI-LGGLADK--

Q1JP63 ------------------------EWRLPSWQVALLTSVVFVGMMSSSTL-WGNISDQ--

Q2XWK0 ------------------------EWRLPSWQVALLTSVVFIGMMASSSL-WGNVSDQ--

Q1LVS8 ------------------------EWHLEDWQVALVSTMVFFGFMVCGVL-CGYIADK--

O08966 ------------------------G---DAWKVDLFQSCVNLGFFLGSLV-VGYIADR--

O15245 ------------------------A---DSWKLDLFQSCLNAGFFFGSLG-VGYFADR--

O77504 ------------------------A---DAWKVDLFQSCVNLGFFLGSLG-VGYIADR--

A7MBE0 ------------------------D---DSWKVDLFQSCVNLGFFLGSLG-VGYIADR--

Q9R0W2 ------------------------A---HSWMLDLFQSVVNVGFFIGAMM-IGYLADR--

O02713 ------------------------A---NSWLLDLFQSAVNVGFFIGSVG-IGYIADR--

Q8MJI6 ------------------------A---RSWMLDLFQSAVNIGFFIGSVG-IGYLADR--

O88446 ------------------------G---NAWMLDLTQAILNLGFLAGAFT-LGYAADR--

O75751 ------------------------V---NAWMLDLTQAILNLGFLTGAFT-LGYAADR--

Q9U539 ------------------------D---QQAWIEISTTSFYVGSFIGNCL-FGYVADK--

O76082 ------------------------E---DDWKAPLTISLFFVGVLLGSFI-SGQLSDR--

Q497L8 ------------------------D---QKWYARMIQPLIIFGVMLGSIT-FSYLSDR--

Q86VW1 ------------------------D---RKWLAMLIQPLFMFGVLLGSVT-FGYFSDR--

Q17QN9 ------------------------N---REWFGRLIQPTFMFGVLLGAVI-FGYLSDR--

Q95R48 ------------------------G---RDFMAATSDSLFMLGVLLGSIV-FGQLSDK--

Q9Y267 ------------------------G---METKKDTAQIMFMAGLPIGSLI-FRLITDK--

Q6A4L0 ------------------------D---RKNLKKTSQSVFMAGLLVGALV-FGPVCDW--

Q9Y226 ------------------------D---RKHLKDTTQSVFMAGLLVGTLM-FGPLCDR--

Q8IVM8 ------------------------D---SQSLTSVAKFVFMAGMMVGGIL-GGHLSDR--

Q66J52 ------------------------N---HRRMRQVAQSIYMAGVLVGSIL-FGGLSDK--

Q91WU2 ------------------------E---QRGLNKVTSTCFFIGVLLGAVV-YGYLSDR--

Q8IZD6 ------------------------N---RSYKVSAASSFFFSGVFVGVIS-FGQLSDR--

Q6DFR1 ------------------------G---AAYEVSVSSSVYFGGVLIGVIS-FGQLSDR--

Q6NUB3 ------------------------G---AAYEVGVSSSVYFGGVLIGVIC-FGQLSDR--

Q28ES4 ------------------------N---EAYKVNLASSLFFAGLLIGNIL-FGPLSDK--

A6NKX4 ------------------------G---DGWKVPLEQVSHLLGWLLGCVI-LGAGCDR--

P47185 ------------YKHSTG------EYYLSNVRMGLLVAMFSVGCSIGGVA-FARLADT--

P13181 -------------KHKDG------THYLSNVRTGLIVAIFNIGCAFGGII-LSKGGDM--

P23585 -------------MKSDG------TYYLSDVRTGLIVGIFNIGCAFGGLT-LGRLGDM--

Q92339 ------------YNPISN------SYSYSAWRQALLTGTINAGCLFGAML-SSPFTER--

O74969 ------------YDPITD------QYSLSSARQGLLTGMVNVGSLFGCII-SSPIADR--

P10870 ----------------PN------HDSFTAQQMSILVSFLSLGTFFGALT-APFISDS--

Q12300 ----------------PN------HSYFTTSQIAILVSFLSLGTFFGALI-APYISDS--

P42833 ------------NIIHDDETIFVSTKKLTDLQIGLIISIFNISCGVGALT-LSKIGDW--

P49374 --------------------------NPDSLTYGGITASMAGGSFLGSLI-SPNFSDA--

O74713 --------------------------SPGSDIQGFITSSMALGSFFGSIA-SSFVSEP--

Q9BE72 -------------------------LTLSCHEQEMVVSSLLIGALLASLT-GGVLIDR--

Q5J316 -------------------------LVLTCHEQEMVVSSLLIGALLASLI-GGVLIDR--

Q32NG5 -------------------------LELTCQQQEIVVSALLIGALVASLV-GGCLIDL--

Q6NWF1 -------------------------LTLSCPEQEQVVGSLLLGAFLLSLG-GGTILDH--

Q0P4G6 -------------------------YQLTCFEQEALVSAVLFGALLASLI-GGIIIDR--

Q6GN01 -------------------------FQLTCFEQEALVSAVLFGALLASLI-GGFIIDR--

O95528 -------------------------FGLSCLEQEFLVGSLLLGALLASLV-GGFLIDC--

Q8VHD6 -------------------------FGLSCLEQELLVGSLLLGALLASLV-GGFLIDC--

Q3UHK1 -------------------------MRLGAMWQELLVSGAVGAAAVAALA-GGALNGA--

Q96QE2 -------------------------LSLDALWQELLVSSTVGAAAVSALA-GGALNGV--

Q9C757 -------------------------VDRNTWLQEMIVSMAVAGAIVGAAI-GGWANDK--

Q8VZR6 -------------------------VKQSSFLQETIVSMALVGAMIGAAA-GGWINDY--

P30606 -------------------------KVLTYGEKELITAATSLGALITSVG-AGTAADV--

P30605 -------------------------KVLTYGEKEIVTAATSLGALITSIF-AGTAADI--

Q10286 -------------------------HELTNGGKEFITSATSLGALLGGII-AGALADF--

Q04162 -------------------------VVLTDVQKELITSSTSVGSFFGSIL-AFPLADR--

Q01440 -------------------------FSEHSWQYALIVAIAIAGAFVGAFI-SGFISAA--

P11166 -TWVHRYG----ESI---------LPTTLTTLWSLSVAIFSVGGMIGSFS-VGLFVNR--

P46896 -TWLYRYE----EPI---------SPATLTTLWSLSVAIFSVGGMIGSFS-VGLFVNR--

P47843 -TLEERSE----TPP---------SSVLLTSLWSLSVAIFSVGGMIGSFS-VGLFVNR--

P14672 -TWLGRQGPEGPSSI---------PPGTLTTLWALSVAIFSVGGMISSFL-IGIISQW--

Q90592 GTEAWGSSEGTLAPSAGFEDPTV-SPHILTMYWSLSVSMFAVGGMVSSFT-VGWIGDR--

P11168 -TISYSMNP---KPTPWAEEETVAAAQLITMLWSLSVSSFAVGGMTASFF-GGWLGDT--

Q5RB09 -SWERRHG----RPI---------DPDTLTLLWSVTVSIFAIGGLVGTLM-VKMIGKV--

A4ZYQ5 -TYFERHA----TFM---------DGKLMLLLWSCTVSMFPLGGLLGSLL-VGLLVDS--

P22732 -TYYGRTG----EFM---------EDFPLTLLWSVTVSMFPFGGFIGSLL-VGPLVNK--

P58353 -TYYDRVG----EYM---------NEFYLTLLWSVTVSMFPFGGFLGSLM-VGPLVNN--

Q863Y9 -THYSRFS----EYI---------SEFSLTLLWSISVSMFPFGGFVGSLM-VGPLVNR--

P43427 -TYYDRNK----ENI---------ESFTLTLLWSLTVSMFPFGGFIGSLM-VGFLVNN--

Q9WV38 -TYYDRNE----ENI---------ESFTLTLLWSLTVSMFPFGGFISSLV-VGNLVNK--

P15686 -VWAKKQE----VH-E----DSPYCTYDNAKLQLFVSSLF-LAGLVSCLF-ASWITRN--

Q39525 -VYEKKQQ----IV-E----TSPYCTYDNPKLQLFVSSLF-LAGLISCIF-SAWITRN--

Q94AZ2 -VYRKVVA----GADK----DSNYCKYDNQGLQLFTSSLY-LAGLTATFF-ASYTTRT--

Q10710 -VYLKKKH----A--H----ENNYCKYDDQRLAAFTSSLY-LAGLAASLV-AGPITRI--

Q41144 -VYRKKKA----DE-S----SNQYCQYDSQTLTMFTSSLY-LAALIASLV-ASTITRK--

P23586 -VYRKQQE----DA-S----TNQYCQYDSPTLTMFTSSLY-LAALISSLV-ASTVTRK--

O65413 -VYEKQKK----DH-D----SNQYCRFDSVSLTLFTSSLY-LAALCSSLV-ASYVTRQ--

Q9SX48 -VDKQMHE----AR-R----ETAYCKFDNQLLQLFTSSLY-LAALASSFV-ASAVTRK--

Q9LT15 -VESQMKK----AK-H----DTAYCKFDNQMLQLFTSSLY-LAALVASFM-ASVITRK--

Q9FMX3 -VLRQMQN----KRGR----ETEYCKYDNELLTLFTSSLY-LAALFASFL-ASTITRL--

Q9SBA7 -VYERKKH----A--H----ENNYCKYDNQFLQLFTSSLY-LAALVASFF-ASATCSK--

Q9SFG0 -VWERKKH----V--H----ENNYCKYDNQFLQLFTSSLY-LAALVASFV-ASATCSK--

Q8L7R8 -VYKLQEE----DRRRRGNSNNHYCLFNSQLLTSFTSSLY-VSGLIATLL-ASSVTRS--

Q93Y91 -VLKKASE----AK------TNVYCVYDSQLLTAFTSSLY-VAGLVASLV-ASRLTAA--

P0AE24 ------------------------HFVLTSRLQEWVVSSMMLGAAIGALF-NGWLSFR--

P0AEP1 ------------------------EFQITSHTQEWVVSSMMFGAAVGAVG-SGWLSFK--

P96710 ------------------------LYSLSPFMEGLVISSIMIGGVVGVGI-SGFLSDR--

P54723 ------------------------QLNLTPVTEGLVASSLLLGAAFGAMF-GGRLSDR--

O34718 ------------------------QLNLNAFTEGLVTSSLLFGAALGAVF-GGRMSDF--

P46333 ------------------------DIPLTTLTEGLVVSMLLLGAIFGSAL-SGTCSDR--

O52733 ------------------------QMNLGSWQQGWVVSAVLLGAILGAAI-IGPSSDR--

P0AGF4 -----------------------LSESAANSLLGFCVASALIGCIIGGAL-GGYCSNR--

P21906 -----------------------LSATAAASLSGMVVVAVLVGCVTGSLL-SGWIGIR--

P15729 --------------------------QTDSLLTGLSVSLALLGSALGAFG-AGPIADR--

Q6AWX0 ------------------------WYNLSSVDVGLVTSGSLYGALFGSIV-AFTIADV--

Q93YP9 ------------------------DLGLTVSEYSVFGSLSNVGAMVGAIA-SGQIAEY--

Q94AF9 ------------------------ELDLSMAQFSAFGSFLNVGGAVGALF-SGQLAVI--

O04036 ------------------------DLSLSVAEYSMFGSILTLGGLIGAVF-SGKVADV--

Q94KE0 ------------------------DLGLSVAQYSMFGSIMTFGGMIGAIF-SGKVADL--

Q9SCW7 ------------------------ELGLSVADYSFFTSVMTLGGMITAAF-SGKIAAV--

Q4F7G0 ------------------------ELGLSVADYSFFTSVMTLGGMITAVF-SGKISAL--

Q8LBI9 ------------------------DLNLSLAEFSMFGSILTIGAMLGAVM-SGKISDF--

P93051 ------------------------DLSLTIAEFSLFGSLLTFGAMIGAIT-SGPIADL--

Q0WQ63 ------------------------ELNLSYSQFSVFGSILNMGAVLGAIT-SGKISDF--

Q3ECP7 ------------------------ELNLSVAEYSLFGSILTIGAMIGAAM-SGRIADM--

Q9LTP6 ------------------------GLNLSLAEFSFFGAVLTIGGLVGAAM-SGKLADV--

Q8GXK5 ------------------------DLNLSIADFSFFGSILTVGLILGALI-CGKLADL--

Q9JJZ1 ------------------------ALRLGDTAASWFGAVVTLGAAAGGVL-GGWLLDR--

P58354 ------------------------APHLDEDAASWFGAIVTLGAAAGGVL-GGWLLDR--

Q9NY64 ------------------------APRLDDAAASWFGAVVTLGAAAGGVL-GGWLVDR--

Q9UGQ3 ------------------------DLHLTKSQASWFGSVFTLGAAAGGLS-AMILNDL--

P43562 -------------------------KYPTPLQQGLLMGSTPLGGIMGCFI-CCIMNDR--

Q9FYG3 -------------------------SGDT-LAEGLVVSVCLGGAFLGSLF-SGGVADG--

P36035 -------------------YDRPTK---DITWGLGLVLFVRSAGAVIFGL-W---TDK--

P47186 -------------------NDKTGEWEISASWQIGLTLCYMAGEIVGLQL-TGPSVDL--

A6QLI1 ------------HRGGK-VIKEKAKFNWDPETVGMIHGSFFWGYIITQIP-GGYIAS--R

Q5W8I7 ------------HKGGKIIIKGKAKFNWDPETVGMIHGSFFWGYTVTQIP-GGYISS--R

A4FV52 ------------HRGGH-VVMQKAQFNWDPETVGLIHGSFFWGYIVTQIP-GGFICQ--K

P34644 ------------PYGK----VHMHEFNWTIDELSVMESSYFYGYLVTQIP-AGFLAA--K

Q66GI9 ------------SRG------------WSKSFSGIVQSSFLWGYLISPIA-GGTLVD--R

Q9FKV1 ------------NQ----------------SSKGTILSTFFVGYACSQVP-GGWAAQ--K

Q46916 ----------------------------DPVGMGYVFSAFSWAYVIGQIP-GGWLLD--R

Q91Y77 ------------KDD-----------DNMAFKAAWVGSLSMGMIFFCCPI-VSVFTD--M

Q8TF71 ------------KDD-----------DKMVFKTAWVGSLSMGMIFFCCPI-VSVFTD--L

A1L1W9 ------------EHD-----------ADLRFKTAWVGSLSMGMIFFCSPI-VSVFTD--L

P36021 ------------EKN-----------RQVEFQAAWVGALAMGMIFFCSPI-VSIFTD--R

O35308 ------------GYS-----------D-----TAWVSSIMLAMLYGTGPL-SSILVT--R

O95907 ------------GYS-----------D-----TAWVSSIMLAMLYGTGPV-SSILVT--R

Q90632 ------------GYS-----------D-----TAWISSIMLAMLYGTGPV-CSIMVN--Q

O35910 ------------GYS-----------D-----TAWISSILLAMLYGTGPL-CSMCVN--R

O15427 ------------GYS-----------D-----TAWISSILLAMLYGTGPL-CSVCVN--R

P57788 ------------GYS-----------D-----TAWISSILLAMLYGTGPL-CSVCVN--R

O15375 ------------SNS-----------E-----TSWFPSILTAVLHMAGPL-CSILVG--R

Q6ZSM3 ------------DYA-----------Q-----TAWIHSIVDCVTMLCAPL-GSVVSN--H

Q8BGC3 ------------DYS-----------Q-----TAWIHSIVDCMTMLCAPL-GSVVSN--Q

Q503M4 ------------DYS-----------G-----TAWIHSLVDCTTMLCAPL-GSLIGN--Q

O15403 ------------SNS-----------R-----ISWIISICVFVLTFSAPL-ATVLSN--R

Q8NCK7 ------------SAQ-----------D-----TAWISALALAVQQAASPV-GSALST--R

Q7RTY0 ------------QAA-----------R-----VSWIASIGIAVQQFGSPV-GSALST--K

Q5R5M4 ------------GKG-----------K-----TAWVGSLASGVGLLASPV-CSLCVS--S

Q7TM99 ------------GKG-----------K-----TAWVGSLASGVGLLASPV-CSLFVS--S

Q5ZJU0 ------------GKG-----------K-----TAWVGSLANGIGLLASPV-CSICVS--S

O15374 ------------TSE-----------Q-----IGWIGSIMSSLRFCAGPL-VAIICD--I

Q08777 ------------SES--------------VSTIGWLFSLFLFVCSASCII-SGTYFD--R

Q08268 ------------NIS--------------SSTISWIFSLYLAISFLSCIL-SGGYFD--R

P39709 ------------SG------MKEDLGFQGNDLVHTQ-VMYTVGNIIFQL--PFLIYL--N

P25621 ------------SG------MKEDLKMVGNDLTVSN-TVFMIGYIVGMV--PNNLML--L

O13880 ------------MNTAQGHSLIQTLSGYSAHYLALGLALFYVGYIIFEV--PSNLMM--A

P40445 ------------DN------FIVDLKMNTNDY-NLGQTVYLVIFLASEL--PGNLLS--K

P15365 ------------MG------LRTDLKMHGDQYSWVT-SAFYFGYLFMNLG-PVQFIF--Q

Q07904 ------------MG------IKDHLK--GNEFSNLG-TIFSAAYIFME---PVVTYL--I

P53322 ------------AG------LSKDIHLVGTQYNTCV-TVFFATYVLFD---PIGTNL--L

P32071 ----------------------NQFNINSTLATL-PLTMFVIGYGIGPLF-WSPLSENSR

P38124 ----------------------EEFHVGHVVATL-NLSLYVLGYGLGPII-FSPLSETAR

Q06451 ----------------------KKYHVGMEAAIL-SVSLMVIGFSLGPLI-WSPVSD--L

P53283 ----------------------KKYHVGMEAAIL-SCSLMVIGFSLGPLI-WSPVSD--L

Q9C0R8 ----------------------EYFGVSEEVIILASVTVFVIGFGVGPLV-FAPMSE--E

Q9C0Q6 ----------------------ADFGVSEEVIILASVTMFVIGFGVGPLV-FAPMSE--E

Q9HF77 ----------------------QIYHIGWTPATL-TTSLFVFGFASGPVI-YGPLSE--L

Q5A0E9 ----------------------AIYHIGVTPAAL-TTALFVFGFASGPVI-YGPLSE--L

Q07824 ----------------------EIYHVIEVVAIL-GITLFVLGFAASPVI-YAPLSE--L

O59698 ----------------------KKYHIGMTTSLL-NVSLFMLGYCLGPIC-WAPMSE--I

O74829 ----------------------VKFHVCRTVALL-GISLYVLGFASGPVV-WAPMCE--L

P38776 ----------------------EHFHISHEVSTL-GITLYVFGLGIGPLF-LSPLSE--L

Q9HDX4 ----------------------EKFGEPETLVVL-GATLYVIGVMLGPLI-FSPLSE--L

P38227 ------------SVI---------------MVNV-SIGVYLLSLGVFPLW-WSSLSE--L

P38125 ------------SAT---------------TINA-TVSVFMAVFSVGPLF-WGALAD--F

P40474 ------------DEE---------------LVNV-TVVVYFVFQGLAPTF-MGGFAD--S

O94607 ------------SAH---------------SNLATINLVGNILLAVVRAP-MVKAAD--V

Q9HE13 ------------DGG---------------SSYSWIGTAYSLAETSILPF-CGIMSE--V

Q04301 ------------SES---------------SKKQWIATSFLLTNTAFQPL-YGKLSD--I

P13090 ------------SEG---------------NSKSWLMASFPLVSGSFILI-SGRLGD--I

Q08902 ------------TNA---------------GQLSWFASAYSLTVGTFILI-AGRLGD--I

P39886 ------------SLA---------------DV-QWITNGYLLALAVSLIT-AGKLGD--R

P76269 ------------TPA---------------SS-IWVVNAYQIAIVISLLS-FSFLGD--M

Q8Y9K8 ------------SSA---------------TV-QWLTTGYLLTLGILVPI-SGLLLQ--W

P28873 ---FGRT--SIYIITLFLFVILQIPTALVNNI----------------------AGLCIL

A4WFG6 ---VPLK--TQLRFGFVLMVAAVAGLMVSHS---IA-------------------LFSVS

A1JSB0 ---IPLK--RQLVFGFILMLIAIAGLMVGHN---LM-------------------IFSIS

A8GKP6 ---IPLK--RQLMFGFVLMVLAIAGLMLGKS---LT-------------------MFSLC

P57601 ---ISLK--KQLIFSFILTIIAVIGIVLCNS---IF-------------------LFSIN

Q8K942 ---ISIK--KQLIFGFLFSIIAILGIVFSTS---IL-------------------LFSIN

Q89A60 ---INLK--TQLIFGFILTIIATLILIFSHN---LT-------------------YFSIS

P76198 ---FGRR--AVILMAVIMYLLFFFGIPACPN---LT-------------------LAYGL

O05390 ---WGPR--KTMTVGLLAFILGSAAFIGWAI---PH-------------------MYYPA

O34864 ---FGAK--RTMFMGLLFYVIGTAAFIVFGF---EQ-------------------LNLPV

O52717 ---ITPR--KAMLIGFVLWCVFHVLFLVFGL---GR-------------------ANYAL

O52718 ---ITPL--KTMMIGFVLWCVFHVLFLVFGL---GH-------------------ANYAL

A9MJT5 ---SGRK--PVAIVGAIVFMMASLLCSQATE---GS-------------------LFLSG

A6TG19 ---SGRK--PVAIVGAAIFVIASLICAQVHT---SS-------------------HFLIG

A0L190 ---IGRK--PITLFSALLFALASYFAARSQS---SD-------------------LFLVA

P0AEY8 ---IGRR--PVMLAGVVWFIVTCLAILLAQN---IE-------------------QFTLL

Q7CP73 ---IGRR--PVLIAGALIFTLACAATLLTTS---MT-------------------QFLVA

P39386 ---IGRR--PVLITGALIFTLACAATMFTTS---MT-------------------QFLIA

Q68WD6 ---YGRR--PIVLLGLFIYIVSSIISIFSVN---IE-------------------MLMIA

Q4UMJ9 ---YGRR--PIALLGLFIYVISSIISIFAVN---IE-------------------MLMIA

Q1RI77 ---YGRR--PVVLFGLCIYAISSIISIFAPN---IE-------------------TLMLA

P32482 ---LGRR--PVLLGGGLAYVVASMGLALTSS---AE-------------------VFLGL

P37597 ---YGRK--PVLLIGLTIFALGSLGMLWVEN---AA-------------------TLLVL

P45123 ---FGRK--PIILLGVIVGALTALVLTEINS---VG-------------------NFTAL

P28246 ---FGRK--PVVLGGTLVFAAAAVACALANT---ID-------------------QLIVM

P31442 ---VGRR--PVILVGMSIFMLATLVAVTTSS---LT-------------------VLIAA

P37482 ---LGNE--RTLWLGLVILLIGVLTRSTGYT---AA-------------------LFFGT

P76242 ---FGME--RSLFAALLLICAGIAIRSLPSP---YL-------------------LFGGT

P17583 ---VSER--RSVAISLLLIAVGALMRELYPQ---SA-------------------LLLSS

P0C105 ---ISYK--RGIVVGLIVAAIGCALFIPAASYRVYA-------------------LFLGA

O25788 ---IGYP--FGVVLGFVITATGCALFYPAAHFGSYG-------------------FFLGA

P11551 ---LSYK--AGIITGLFLYALGAALFWPAAEIMNYT-------------------LFLVG

P44776 ---YSYK--AGILLGLALYAIGAFLFWPAAQYEIFN-------------------FFLVS

A1A9E1 ---IGFN--RSYYLASFIFAAGCAGLGLMIGF--WS--------------------WLAW

A9MHY5 ---IGFN--RSYYLASLIFAAGCVGLGVMVGF--WS--------------------WMSW

A4W8S1 ---FGFN--RSYYLASLIFAAGCVGLGLMVGF--WS--------------------WMTW

A1JMG4 ---LGFN--RSYHYSCILFALATCGLMLSVDF--WS--------------------WLGW

Q0TK80 ---YERK--KVILLARGTCGIGFIGLCLNALL--PE-------------------PSLLA

O06473 ---IGAK--WTLILG-LIFIIVFAGLGGSSSS--IA-------------------QLVGY

Q2FI61 ---FGT---KIV--LTTGFILAFTSLFLVIWFPASP------------------FVIIFS

Q5HQE8 ---FGS---KLV--LTSGFLLAFISLFLVIWFPASP------------------FIIIFS

Q49WE5 ---LGT---KLV--LTLGFLLAFASLFLVIWFPTSP------------------FVLIAS

Q4L523 ---FGT---KLV--LTLGFLLAFISLFLVIWFPTNP------------------IVIILS

P33026 ---RGDR--KSL--IVFCCLLGVLACTLFAWN-RNY------------------FVLLFV

P31436 ---QGDR--KLL--ILLCCLFGVLACTLFAWN-RNY------------------FILLST

Q9S3K0 ---QGDR--KTL--ILQCCLLGALACLLYAWN-RNY------------------FVLLFI

P31675 ---QGDR--RKL--IIFCCLMAIGNALLFAFN-RHY------------------LTLITC

P31126 ---FDKK--RYM--LLAITAFASGFIAITLVN-NVT------------------LVVLFF

Q58955 ---YG-K--KFF--IVCGTFFYGVSTLMYNFVSTV-------------------LGFLIV

P02920 ---LGLR--KYLLWIITGMLVMFAPFFIFIFGPLLQYN--------------ILVGSIVG

Q4UK37 ---FGH---RRGWALVSQIFLILAMTGFLNVSPCNN------------------LYITAI

Q1RKF6 ---FGA---RRGWAIASQLCLILAMTGFLSFSPCDN------------------IYITAT

Q4ULW4 ---WGH---RKSWLILCSSLMVLVLIAMSKENPEVS------------------LTSLYF

Q1RI01 ---WGH---RKGWLILCSSLMALVLIAMGRENPAAS------------------LTALYF

Q68WQ5 ---LGH---RLSWICLTSTTLISLTSILSFLDPGTD------------------LVLLSF

Q4UL88 ---FGH---RLSWICLTSTALIFLIYIFSFLDPRTN------------------LVLFTF

Q92HQ3 ---FGH---RLSWICLTSSALIFLIYIFSFLDPSTN------------------LLLFAF

Q1RIL0 ---FGH---RLSWICLTSIALVFFVYILSFLNPFDN------------------LLLFAS

Q4UMU2 ----GY---KYC-LIIALVSCICCVYILTNFNPNTH------------------FIPFAL

Q68W71 ----GY---KYC-LVITLINCIVCVYVLTSFNPNTN------------------FTPFVL

Q1RHK8 ----GY---KYC-LIFSLLSCICCVYILTGFNPTTN------------------FISFSL

Q1LTM2 ---LGAK--PMIITGMLMRALGFIFMGIADKP----------------------W-LLWV

Q2NTK5 ---LGAK--PMIIAGMLMRALGFVLMGIADEP----------------------W-LLWL

P55705 ---YGNR--LMMVRALLGLAITQLLVAFAQDV----------------------WTILAL

Q7Z3Q1 ---YGRK--FPMILSSVGALATSVWLCLLC-Y--FAFP--------------FQLLIAST

Q05B81 ---VGRR--PLLVLASLGLLLQTVLSIFVV-Q--LHLH--------------IGYLVLGR

P76470 ---LMRK--LGARTWIGTTTLLWGFLSAAM-A--WADT--------------EAKFLIVR

P32135 ---LPRR--YTMSFSLIATGLLGVYLTTMPGY--WG-------------------ILFVW

P38358 ---RGIF--QAFQNLLLGFGAICGASFGGTIASSIGWR--------------WCFLIQVP

A4WAE6 ----ERR--KLLIGLFVLFIASHVLSFLAWNF----------------------NVLVIS

A6T8Y8 ----ERR--RLLIGLFILFIASHVLSFFAWNF----------------------DVLVIS

Q6CZ44 ----ERR--SLLIKIFILFAISNVLSGLAWNY----------------------WVLIMA

Q888L8 ----ERR--KLLMFVFGLFVVSHVISAMASSF----------------------AILLVS

Q1IB51 ----ERR--RLLLFVFVVFILSHLMSWLSQSF----------------------AMLLVS

A6UZY0 ----ERR--KLLVGVFLLFIASHVLSGLAWNF----------------------PVLMLS

Q4QP52 ----ERK--SLLIKLFIIFIVGHILLVIAWNF----------------------WILLLA

Q9CM87 ----ERR--GLLIKLLVLFILSHLLSVIAWDF----------------------WVLVLA

O25797 ----ERK--RLLLFLFALFILSHILSALAWNF----------------------WVLLLS

Q17YP7 ----ERK--RLLLFLFGLFIVSHILSVVAWDF----------------------WVLLIS

P77389 ----ARR--SALIFLMAIFTLGNVLSAIAPDY----------------------MTLMLS

O31577 ----PRK--PVLLGLMAIFILSNLMSALAPNF----------------------AVLAIS

O34367 ----SRK--TLLLWIMFIFIAGNTMAATASSI----------------------GILLAA

A1AHK2 ----DRR--YVVILFAVLLTLSCLLVSFANSF----------------------SLLLIG

A9MWE8 ----DRR--YIVILFAVLLTASCLMVSFANSF----------------------TLLLLG

P23910 ----SLK--HILLFLVALCVIGNAMFTLSSSY----------------------LMLAIG

P31141 ----PRR--SSLLGFILAFAAAHAVGAGTTSF----------------------PVLVAC

P43531 ----GRK--PVMVTALLLASICTLLSTMMTSW----------------------HGILIM

Q8K902 ----GRK--SIMSSSLLIAAVLTIICSISNNW----------------------TVIVFL

P57648 ----GRK--SIMSTSLFIAAMLTMICSMMTSW----------------------ISIVLL

Q89A23 ----GRK--VVMSSSLFLASFCTFCCSNMNSW----------------------ESIIFM

P37498 ----GRK--PIMGISMLAASVLCLASAFSPSF----------------------HTLLVL

P0A0J4 ----GKK--LIICIGLILFSVSEFMFAVGHNF----------------------SVLMLS

Q07282 ----GRR--PVLLLSLLGATLDYALMATASVV----------------------WVLYLG

P02982 ----GRR--PVLLVSLAGAAVDYAIMATAPFL----------------------WVLYIG

P02981 ----GRR--PVLLASLLGATIDYAIMATTPVL----------------------WILYAG

P70187 ----GRK--SFLLLTVFFTCAPIPLMKISP------------------------WWYFAV

Q5SR56 ----GRK--PFLLGTVFFTCFPIPLMRISP------------------------WWYFAM

P77726 ----GRK--PLIVGGLAVFAAGSVIAALSDSI----------------------WGIILG

Q89AA9 ----GQK--LIINIGLLCFLLGNIIAWSSNSI----------------------WGIILG

Q8K999 ----GRK--QIIIFGLFIFFIGSLIVVSTNSI----------------------FGLIIG

P57538 ----NRK--KIILLGLFMFFIGNIISASIHSI----------------------WGLIIG

Q5HIA2 ----GRK--VVLTSTIILMAFSTLTIGLLPSYDQIGLW--------------APILLLLA

Q4L3Q4 ----GRK--VVLTTTIILMALSTLTIGVLPNYDMIGLW--------------APALLLLA

Q5HRH0 ----GRK--IVLTTTIILMAFSTLLIGVLPTYNEIGVW--------------APILLLLA

P0C0L7 ----GRQ--KILAITIVIMSISTFCIGLIPSYDTIGIW--------------APILLLIC

Q47421 ----GRQ--KILAITIIIMSISTFCIGLIPSYERIGIW--------------APILLLLA

P0A2G3 ----GRR--KGLMVTLAIMGCGTLLIALVPGYQTIGLA--------------APALVLLG

P16482 ----GRR--KGLIVTLSIMATGTFLIVLIPSYQTIGLW--------------APLLVLIG

P0AEX3 ----GRK--KSMLLSVCMMCFGSLVIACLPGYETIGTW--------------APALLLLA

P76350 ----GRK--RMLMLTVWMMGIATALIGILPSFSTIGWW--------------APILLVTL

P41036 ----GRR--LAMVTSIVLFSAGTLACGFAPGY----------------------ITMFIA

Q9SYQ1 ----GRR--RVYGLCLIIMILSSFGCGFSVCTTRRSC---------------VMVSLGFF

Q9S735 ----GRR--KVYGLSLLIMVFSSFGCGFSVCTTRRSC---------------VMVSLGFF

P76230 ----GRR--NAFILYEAIHIASMVVGAFSPNM----------------------DFLIAC

P38055 ----GRR--RAFRINLLIVGIAATGAAFVPDM----------------------YWLIFF

Q46909 ----GRQ--KIFTFSFLLITLASFLQFFATTP----------------------EHLIGL

P31679 ----GRR--KMFLIDIIAIGVISVATMFVSSP----------------------VELLVM

O24723 ----GRK--GVTVVGFVLFCLATAGLGLTGDI----------------------HSFAAL

O30513 HLGVSRK--KVIAVCIILFSLCTVLCGFSETT----------------------TQFSIF

Q43975 ----GRK--IVLSMSMLVFGGFTLACAYSTNL----------------------DSLVIF

Q51955 ----GRK--GVLVGAVLVFGGFSLASAYATNV----------------------DQLLVL

Q9I6Q3 ----GRK--LVLVAAVFLFGLFSLASAYSTNV----------------------EQLLAL

P77589 ----GRK--RILIGSVALFGLFSLATAIAWDF----------------------PSLVFA

P94131 ----GRV--RIVVISILTFSILTCGLGLTQSF----------------------IQFGVL

P71369 ----GRV--RVLTWTILLFAVFTGLCAIAQGY----------------------WDLLIY

O34691 ----GRK--KVFIITLLCFSIGSGISAFVTSL----------------------SAFLIL

P0AGC0 ---KNTK--QFLPFMLILSAICMLGFSASM-GSGSVSL----------------FLMIAF

P27669 ---SNAR--YFMGIGLIATGVVNILFGFS-----T-SL----------------WAFALL

P96335 ---SNPR--VFLPFGLLLSGLCMTLMGLFP-WATS-GI----------------AIMWVM

P08194 ---SNPR--VFLPAGLILAAAVMLFMGFVP-WATS-SI----------------AVMFVL

P37948 ---CNPR--YFLATGLFLSAIVNILFVSMP-WVTS-SV----------------TIMFIF

P12681 ---ASPK--VFMACGLVLCAIVNVGLGFS-----S-AF----------------WIFAAL

Q5M7K3 ---LPLR--YYLSGGMIICGIFTSFMGLGYYW-NIHAL----------------WYYILF

Q9WU81 ---LPLR--YYLSAGMVLSGLFTSLFGLGYFW-NIHML----------------WYFVLI

Q58CV5 ---LPLR--YYLTAGMLLSGLFTSLFGLGYFW-NIHVL----------------WYFVLV

Q7SY29 ---LPLR--YYLSTGMLLSGLFTALFGLGFYW-QIHSL----------------WYYCLV

P57057 ---LPIR--YYLTFGMLASGAFTALFGLGYFY-NIHSF----------------GFYVVT

Q17QZ3 ---LNLR--WVLSFGMCSSALVVFVFGTLTEWLHFYNK----------------GLYCSL

Q3TIT8 ---LNLR--WVLSFGMCSSAFVVFVFGTLTEWLHFYNK----------------WFYCGL

Q640L2 ---LNMR--LVLTFGMCSSAITMFVFGTLTEWLQFYNK----------------IFYCLV

Q5F3N0 ---LNLR--WVLSFGMCSSALVVFFFGTLTEWLHFYNK----------------WFYCCL

Q09037 ---FGLK--KSFIIVSITCTIACVVVQVAIEYNN------------------Y-YALCTG

Q06222 ---FGLK--KSFIIVGVMSVVASALNHISVATNE------------------F-WVLCAG

P13865 ---IGAR--LSFLLVGLVGVVASVMYHASCAADE------------------F-WVLIVG

P46499 ---FGRK--PVSFVGISIGIIFGVASGFAPSWE----------------------VFAVL

P54219 ---IGYH--IPMFAGFVIMFLSTVMFAFSGTY----------------------TLLFVA

Q6NT16 ---IGAK--FMFVAGMFVSGGVTILFGVLDRVPDGPVF--------------I-AMCFLV

Q8R0G7 ---YNRK--YLMCGGIAFWSLVTLGSSFIPREHF--------------------WLLLLT

Q5XGK0 ---YNRK--LIMCVGISFWSLVTLLSSFVSNQYF--------------------WLFLIT

Q7ZU13 ---YNRK--LIMCVGIFFWSVVTLASSFIGKDHF--------------------WALLLT

A2CER7 ---YDRK--LIMIVGLVMWIVTTLGSSFVRKSHF--------------------WVLVAT

Q9D232 ---YNRK--AILSFGILLWSGAGLSSSFISYQYS--------------------WLFFLS

Q6ZMD2 ---HSRK--ATMSFGILLWSGAGLSSSFISPRYS--------------------WLFFLS

Q6GPQ3 ---RPRREPLVVSITILVAASCLYAYVHVPASHN------------------K-YYMLLA

A5IVG9 ---VGAK--WVFFTSFIVLLFPIFFLSQA-QTP---------------------GMLMAS

Q5HLK7 ---VGAK--WVFFWSFIVLLLPIFLLGQA-QSP---------------------GMLMLS

P46907 ---FGAR--LMFMVSFILLLFPVFWISIA-DSL---------------------FDLIAG

P10903 ---FGGR--RWTAFSTGILIIPCVWLGFAVQDTST-----------------PYSVFIII

P37593 ---FGGR--KWTVLSTVILIIPCAWLGFAVQNPAT-----------------PFGVFMLI

P37758 ---FGGR--RWTVFSTAILIIPCVWLGIAVQNPNT-----------------PFGIFIVI

Q9P3K8 ---VGP---GPLSFVSALFFAAGYGLAAGVYKREADGAALGNGADGEDTGRLAYAAMITA

Q6FWD4 ---HGP---ITLSLSSVLGFIPSYAYLAYTFHR---DHSFTNESDG------SFTPTLVC

Q6CPY8 ---HGP---VILSWLSFIGFVPSYLYLSCVFQL---G------SDP------CFALSVVA

P22152 ---FGPR--LVFIGLLLVGSIPTAMAGLVT----------------------SPQGLIAL

Q02563 ---LGRR--QCLLISLSVNSVFAFFSSFV---QGYGT-------------------FLFC

Q496J9 ---VGRK--QSLLICMSVNGFFAFLSSFV---QGYGF-------------------FLFC

Q63564 ---LGRK--KVLSMSLAINASFASLSSFV---QGYGA-------------------FLFC

Q1JP63 ---YGRK--TGLKISVLWTLYYGILSAFA---PVYSW-------------------ILVL

Q2XWK0 ---YGRR--TGLKISVIWTLYYGILSAFA---PVYSW-------------------ILVL

Q1LVS8 ---YGRW--KVVFGGFVWASYFSFLTSFS---TSYGW-------------------FIFL

O08966 ---FGRK--LCLLVTTLVTSLSGVLTAVA---PDYTS-------------------MLLF

O15245 ---FGRK--LCLLGTVLVNAVSGVLMAFS---PNYMS-------------------MLLF

O77504 ---FGRK--LCLLLTTLINAVSGVLTAVA---PDYTS-------------------MLLF

A7MBE0 ---FGRK--VCLLATTLTCASLGVLTAVA---PDYTS-------------------LLIF

Q9R0W2 ---FGRK--FCLLVTILINAISGALMAIS---PNYAW-------------------MLVF

O02713 ---FGRK--LCLLLTILINAVSGVLMAIS---PTYTW-------------------MLVF

Q8MJI6 ---FGRK--LCLLVTILINAAAGVLMAVS---PNYTW-------------------MLIF

O88446 ---YGRL--IVYLISCFGVGITGVVVAFA---PNFSV-------------------FVIF

O75751 ---YGRI--VIYLLSCLGVGVTGVVVAFA---PNFPV-------------------FVIF

Q9U539 ---FGRR--RSFFVILTVLIVCGTASSFA---KDIES-------------------FIIL

O76082 ---FGRK--NVLFVTMGMQTGFSFLQIFS---KNFEM-------------------FVVL

Q497L8 ---FGRR--MALWCTSIGVFFFGIASLFI---FDYLS-------------------FMIT

Q86VW1 ---LGRR--VVLWATSSSMFLFGIAAAFA---VDYYT-------------------FMAA

Q17QN9 ---AGRR--LVLWASSTGVFLFGIAAAFT---FDYYS-------------------FIVA

Q95R48 ---YGRK--PILFASLVIQVLFGVLAGVA---PEYFT-------------------YTFA

Q9Y267 ---MGRY--PAILLSLLGLIIFGFGTAFM---NSFHL-------------------YLFF

Q6A4L0 ---IGRR--PSLLMQVLLSGITSMATAFV---SSFEL-------------------YLAL

Q9Y226 ---IGRK--ATILAQLLLFTLIGLATAFV---PSFEL-------------------YMAL

Q8IVM8 ---FGRR--FVLRWCYLQVAIVGTCAALA---PTFLI-------------------YCSL

Q66J52 ---FGRR--PLNIWSNLQMFVTGICAAFS---PNYIW-------------------YCIF

Q91WU2 ---FGRR--RLLLVAYVSTLALGLMSAAS---VNYIM-------------------FVTT

Q8IZD6 ---FGRK--KVYLTGFALDILFAIANGFS---PSYEF-------------------FAVT

Q6DFR1 ---FGRK--PVYVTGLALEVVFAVMNALT---PIFPL-------------------FLLT

Q6NUB3 ---FGRK--PVYVTGLALEVTFAVMNALT---PIFSM-------------------FLIT

Q28ES4 ---LGRR--PVYLSGLFFDITFGYCTALA---PSYEV-------------------FAVS

A6NKX4 ---FGRR--AVFVASLVLTTGLGASEALA---ASFPT-------------------LLVL

P47185 ---LGRR--LAIVIVVLVYMVGAIIQISS--NHKWYQ-------------------YFVG

P13181 ---YGRK--KGLSIVVSVYIVGIIIQIAS--INKWYQ-------------------YFIG

P23585 ---YGRR--IGLMCVVLVYIVGIVIQIAS--SDKWYQ-------------------YFIG

Q92339 ---IGKK--YSICFFSGVYIIAELLLVTA--VPSWIQ-------------------VLVG

O74969 ---FGKR--LSIIGFCAVYIIGIIVQVTA--VPSWVQ-------------------IMVA

P10870 ---YGRK-PTIIFSTIFIFSIGNSLQVGA--G-GITL-------------------LIVG

Q12300 ---YGRK-PTIMFSTAVIFSIGNSLQVAS--G-GLVL-------------------LIVG

P42833 ---IGRK--GGIWFALVVYCIGITIQILS--YGRWYF-------------------LTLG

P49374 ---FGRK--VSLHICAALWIIGAILQCAA--QDQ-AM-------------------LIVG

O74713 ---FGRR--LSLLTCAFFWMVGAAIQSSV--QNR-AQ-------------------LIIG

Q9BE72 ---YGRR--TAIILSSCLLGLGSLVLILS--L-SYTV-------------------LIVG

Q5J316 ---YGRR--AAIILSSCLLGLGSLVLIIS--L-SYTT-------------------LIGG

Q32NG5 ---YGRR--TTIIFTSILLVFANLLPVVV--V-SYGS-------------------LIAG

Q6NWF1 ---YGRR--FTIILTALLCVLGTLLSVCV--V-SFWA-------------------LVVG

Q0P4G6 ---WGRR--TAILASNLVVLAGSIILIAT--S-TFWW-------------------LIVG

Q6GN01 ---SGRR--TSIMGSNLVVLAGSIILIAT--S-SFWW-------------------LVVG

O95528 ---YGRK--QAILGSNLVLLAGSLTLGLA--G-SLAW-------------------LVLG

Q8VHD6 ---YGRR--RAILGSNAVLLAGSLILGLA--S-SLPW-------------------LLLG

Q3UHK1 ---LGRR--SAILLASALCTVGSAVLAAA--A-NKET-------------------LLAG

Q96QE2 ---FGRR--AAILLASALFTAGSAVLAAA--N-NKET-------------------LLAG

Q9C757 ---LGRR--SAILMADFLFLLGAIIMAAA--P-NPSL-------------------LVVG

Q8VZR6 ---YGRK--KATLFADVVFAAGAIVMAAA--P-DPYV-------------------LISG

P30606 ---FGRR--PCLMFSNLMFLIGAILQITA--H-KFWQ-------------------MAAG

P30605 ---FGRK--RCLMGSNLMFVIGAILQVSA--H-TFWQ-------------------MAVG

Q10286 ---FGRK--PVIAIASIIIIVGSIVQVTA--H-HLWH-------------------MIVG

Q04162 ---YGRR--ITLAICCSIFILAAIGMAIA--R-TLTF-------------------LICG

Q01440 ---FGRR--PCIAVADALFVIGSVLMGAA--P-NVEV-------------------VLVS

P11166 ---FGRR--NSMLMMNLLAFVSAVLMGFSKLGKSFEM-------------------LILG

P46896 ---FGRR--NSMLMSNILAFLAAVLMGFSKMALSFEM-------------------LILG

P47843 ---FGRR--NSMLIVNLLAIAGGCLMGFCKIAESVEM-------------------LILG

P14672 ---LGRK--RAMLVNNVLAVLGGSLMGLANAAASYEM-------------------LILG

Q90592 ---LGRV--KAMLVVNVLSIAGNLLMGLAKMGPSHIL-------------------IIAG

P11168 ---LGRI--KAMLVANILSLVGALLMGFSKLGPSHIL-------------------IIAG

Q5RB09 ---LGRK--HTLLANNGFAISAALLMACSLQAGAFEM-------------------LIVG

A4ZYQ5 ---CGRK--GTLLINNIFAIIPAILMGVSKVAKAFEL-------------------IVFS

P22732 ---FGRK--GALLFNNIFSIVPAILMGCSRVATSFEL-------------------IIIS

P58353 ---LGRK--GTLLFNNIFSIVPALLMGFSELAKSFEM-------------------IIVA

Q863Y9 ---LGRK--GTLLFNNIFSIVPAILMGTSKTARSYEM-------------------IILS

P43427 ---LGRK--GALLFNNIFSILPAILMGCSKIAKSFEI-------------------IIAS

Q9WV38 ---LGKK--RALLFNNIFSILPAIFMGCSQIAQSFEL-------------------IIIS

P15686 ---WGRK--VTMGIGGAFFVA-GGLVNAF--AQDMAM-------------------LIVG

Q39525 ---WGRK--ASMGIGGIFFIAAGGLVNAF--AQDIAM-------------------LIVG

Q94AZ2 ---LGRR--LTMLIAGVFFII-GVALNAG--AQDLAM-------------------LIAG

Q10710 ---YGRR--ASIISGGISFLI-GAALNAT--AINLAM-------------------LLLG

Q41144 ---FGRK--LSMLFGGVLFCA-GAIINGA--AKAVWM-------------------LILG

P23586 ---FGRR--LSMLFGGILFCA-GALINGF--AKHVWM-------------------LIVG

O65413 ---FGRK--ISMLLGGVLFCA-GALLNGF--ATAVWM-------------------LIVG

Q9SX48 ---YGRK--ISMFVGGVAFLI-GSLFNAF--ATNVAM-------------------LIVG

Q9LT15 ---HGRK--VSMFIGGLAFLI-GALFNAF--AVNVSM-------------------LIIG

Q9FMX3 ---FGRK--VSMVIGSLAFLS-GALLNGL--AINLEM-------------------LIIG

Q9SBA7 ---LGRR--PTMQLASIFFLI-GVGLAAG--AVNIYM-------------------LIIG

Q9SFG0 ---LGRR--PTMQFASIFFLI-GVGLTAG--AVNLVM-------------------LIIG

Q8L7R8 ---WGRK--PSIFLGGVSFLA-GAALGGS--AQNVAM-------------------LIIA

Q93Y91 ---YGRR--TTMILGGFTFLF-GALINGL--AANIAM-------------------LISG

P0AE24 ---LGRK--YSLMAGAILFVLGSIGSAFA---TSVEM-------------------LIAA

P0AEP1 ---LGRK--KSLMIGAILFVAGSLFSAAA---PNVEV-------------------LILS

P96710 ---FGRR--KILMTAALLFAISAIVSALS---QDVST-------------------LIIA

P54723 ---HGRR--KTILYLALLFIAATLGCTFS---PNASV-------------------MIAF

O34718 ---NGRR--KNILFLAVIFFISTIGCTFA---PNVTV-------------------MIIS

P46333 ---WGRR--KVVFVLSIIFIIGALACAFS---QTIGM-------------------LIAS

O52733 ---FGRR--KLLLLSAIIFFVGALGSAFS---PEFWT-------------------LIIS

P0AGF4 ---FGRR--DSLKIAAVLFFISGVGSAWP---ELGFTSINPDNTVPVYLAG-YVPEFVIY

P21906 ---FGRR--GGLLMSSICFVAAGFGAALT---EKLFGT--GGSALQI---------FCFF

P15729 ---HGRI--KTMILAAVLFTLSSIGSGLP---FTIW-------------------DFIFW

Q6AWX0 ---IGRR--KELILAALLYLVGALVTALA---PTYSV-------------------LIIG

Q93YP9 ---VGRK--GSLMIAAIPNIIGWLSISFA---KDTSF-------------------LYMG

Q94AF9 ---LGRR--RTLWACDFFCVFGWLSIAFA---KNVFW-------------------LDLG

O04036 ---LGRK--RTMLFCEFFCITGWLCVALA---QNAMW-------------------LDCG

Q94KE0 ---MGRK--GTMWFAQIFCIFGWVAVALA---KDSMW-------------------LDIG

Q9SCW7 ---IGRR--QTMWIADVFCIFGWLAVAFA---HDKML-------------------LNIG

Q4F7G0 ---VGRR--QTMWISDVCCIFGWLAVAFA---HDIIM-------------------LNTG

Q8LBI9 ---SGRK--GAMRTSACFCITGWLAVFFT---KGALL-------------------LDVG

P93051 ---VGRK--GAMRVSSAFCVVGWLAIIFA---KGVVA-------------------LDLG

Q0WQ63 ---IGRK--GAMRLSSVISAIGWLIIYLA---KGDVP-------------------LDFG

Q3ECP7 ---IGRR--ATMGFSEMFCILGWLAIYLS---KVAIW-------------------LDVG

Q9LTP6 ---FGRR--GALGVSNSFCMAGWLMIAFS---QATWS-------------------LDIG

Q8GXK5 ---VGRV--YTIWITNILVLIGWLAIAFA---KDVRL-------------------LDLG

Q9JJZ1 ---AGRK--LSLLLCTVPFVTGFAVITAA---RDVWM-------------------LLGG

P58354 ---AGRK--LSLVLCALPFVAGFAVITAA---QNLWM-------------------LLGG

Q9NY64 ---AGRK--LSLLLCSVPFVAGFAVITAA---QDVWM-------------------LLGG

Q9UGQ3 ---LGRK--LSIMFSAVPSAAGYALMAGA---HGLWM-------------------LLLG

P43562 ---FSRI--YQFQSGIIIWNIVTLLNFCI---WDILG-------------------LLIC

Q9FYG3 ---FGRR--RAFQICALPMILGAFVSGVS---NSLAV-------------------MLLG

P36035 ---SSRK--WPYITCLFLFVIAQLCTPWC---DTYEK-------------------FLGV

P47186 ---VGNR--YTLIIALFFLAAFTFILYFC---NSLGM-------------------IAVG

A6QLI1 ---LAAN--RVFGAAILLTSTLNMLIPSA---ARVHYG-----------------CVIFV

Q5W8I7 ---LAAN--RVFGAAILLTSTLNMFIPSA---ARVHYG-----------------CVMFV

A4FV52 ---FAAN--RVFGFAIVATSTLNMLIPSA---ARVHYG-----------------CVIFV

P34644 ---FPPN--KLFGFGIGVGAFLNILLPYG---FKVKSD----------------YLVAFI

Q66GI9 ---YGGK--VVMAWGVALWSLATFLTPWA---ADSSL-----------------WALLAA

Q9FKV1 ---IGGR--KVLLLSFVLWSSTCFLVPLD---PN-RVG-----------------LLVVA

Q46916 ---FGSK--RVYFWSIFIWSMFTLLQGFVDIFSGFGII----------------VALFTL

Q91Y77 ---FGCR--RTAVLGAAVGFVGLMSSSFV-----SSIE----------------PLYFTY

Q8TF71 ---FGCR--KTAVVGAAVGFVGLMSSSFV-----SSIE----------------PLYLTY

A1L1W9 ---LGCR--ITAVGGAAVGCVGLLASSFV-----TSLG----------------PMYFTY

P36021 ---LGCR--ITATAGAAVAFIGLHTSSFT-----SSLS----------------LRYFTY

O35308 ---FGCR--PVMLAGGLLASAGMILASFA-----SRLV----------------ELYLTA

O95907 ---FGCR--PVMLAGGLLASAGMILASFA-----TRLL----------------ELYLTA

Q90632 ---FGCR--PVMLIGGLLASSGMILASFT-----TNII----------------ELYLTA

O35910 ---FGCR--PVMLVGGLFASLGMVAASFC-----RSII----------------QIYLTT

O15427 ---FGCR--PVMLVGGLFASLGMVAASFC-----RSII----------------QVYLTT

P57788 ---FGCR--PVMLVGGLFASMGMAIASFC-----TSIV----------------QIYLTA

O15375 ---FGCR--VTVMLGGVLASLGMVASSFS-----HNLS----------------QLYFTA

Q6ZSM3 ---LSCQ--VGIMLGGLLASTGLILSSFA-----TSLK----------------HLYLTL

Q8BGC3 ---LSCQ--AGIMLGGLLASTGFILGSFA-----TSLK----------------HLYLSL

Q503M4 ---LSCR--IAVILGGFLASVGLVLSSFA-----TSLE----------------YLYATL

O15403 ---FGHR--LVVMLGGLLVSTGMVAASFS-----QEVS----------------HMYVAI

Q8NCK7 ---WGAR--PVVMVGGVLASLGFVFSAFA-----SDLL----------------HLYLGL

Q7RTY0 ---FGPR--PVVMTGGILAALGMLLASFA-----TSLT----------------HLYLSI

Q5R5M4 ---FGAR--PVTIFSGFMVAGGLMLSSFA-----PNIY----------------FLFFSY

Q7TM99 ---FGAR--PVTIFSGFLVAGGLMLSSLA-----PNIY----------------FLFFSY

Q5ZJU0 ---FGAR--PVAIFSGFMVAGGLMMSSFA-----PNIY----------------FLYLSY

O15374 ---LGEK--TTSILGAFVVTGGYLISSWA-----TSIP----------------FLCVTM

Q08777 ---NGFR--TIMIVGTVFHVAGLFATANS-----TKYW----------------HFILSF

Q08268 ---NGSI--GLMCTGTVIYAGGLFALANC-----KSVW----------------QFILAF

P39709 ---KL-P--LNYVLPSLDLCWSLLTVGAAYV----NSV----------------PHLKAI

P25621 ---CV-P--PRIWLSFCTFAWGLLTLGMYKV----TSF----------------KHICAI

O13880 ---FIEP--RIWVSRIQLTI-GVVGACHAVLGTKHGNA----------------QSYVAL

P40445 ---RFGP--ERVIP-VQIVLWSVICITQAGL----KNR----------------GQFIAT

P15365 ---RTSH--MSKMLAVFIVIWGMLLALHAAP---TVKY----------------PSFIVL

Q07904 ---QKFP--ISKILGTFITVWGIVLACHAAC---KT-Y----------------ASLMVV

P53322 ---KIMG--PPLMMSICLTCFG-AISLGTAW---VKNY----------------AQLIVV

P32071 ---IGRT--PLYIITLFIFFILQIPTALSNHI----------------------AGLSVL

P38124 ---YGRL--NLYMVTLFFFMIFQVGCATVHNI----------------------GGLIVM

Q06451 ---YGRR--VAYFVSMGLYVIFNIPCALAPNL----------------------GSLLAC

P53283 ---YGRR--VAYFVSMGLYVIFNIPCALAPNL----------------------GCLLAC

Q9C0R8 ---VGRK--PIYVVTLFIAVVFIVPCGAAKNI----------------------ATLIVC

Q9C0Q6 ---VGRK--PIYVVTLFVAVVFIVPCGAAQNI----------------------ATLLIC

Q9HF77 ---FGRK--LVMVPSCLGYVCFSFAVATAKDI----------------------QTIMIC

Q5A0E9 ---FGRK--IIMVISSFLYVCFSFAVATAKDI----------------------QTIMIC

Q07824 ---YGRK--GVLVLSAFGFALFQFAVATAENL----------------------QTIFIC

O59698 ---TGRK--TPLYIGLFLFSVFQIAVATAQDI----------------------QTIMIC

O74829 ---FGRR--RPMIIAVFIFCIFHIAVATAKDI----------------------QTVMIC

P38776 ---YGRR--ITFLYALTLSIIWQCLTIWSKTI----------------------TGVMFG

Q9HDX4 ---YGRR--PLNIFGYTLFALMQIPTALSVNL----------------------AMFVVF

P38227 ---EGRR--TTYITSFALLFAFNIGSALAP---DI-------------------NSFIAL

P38125 ---GGRK--FLYMVSLSLMLIVNILLAAVPV--NI-------------------AALFVL

P40474 ---LGRR--PVVLVAIVIYFGACIGLACAQ---TY-------------------AQIIVL

O94607 ---FGRS--ESLSLALGMTVLGYLSLAFSR---NI-------------------QMFTVA

Q9HE13 ---VGRK--IVLYTSIVLFLFGSAMCGAAQ---NM-------------------LWLVLC

Q04301 ---TGRK--SALLTAQFFFGLGCLLTCFAR---NV-------------------TEFSIA

P13090 ---YGLK--KMLLVGYVLVIIWSLICGITK---YSGSD----------------TFFIIS

Q08902 ---FGHK--KFFVLGFFWYALWSLLAGFSV---YSN-Q----------------IFFDCC

P39886 ---FGHR--QTFLVGVAGFAVTSAAIGLSG---SV-------------------AAIVVF

P76269 ---FGYR--RIYKCGLVVFLLSSLFCALSD---SL-------------------QMLTLA

Q8Y9K8 ---FTTR--GLFFTAVSFSIAGTLIAALSP---TF-------------------AMLMIG

P28873 RFLGGFFASPCLATGGASVADVVKFWNLPVGL------AAW--------SLGAVCGPSFG

A4WFG6 MFVLGLVSGIT--MSIGTFLITHMYEGRQR--G-----ARL-------LFTDSFFSMAGM

A1JSB0 MFILGVVSGIT--MSIGTFLITHMYEGRQR--G-----SRL-------LFTDSFFSMAGM

A8GKP6 MFILGVVSGIT--MSIGTFLITHMYAGRQR--G-----SRL-------LFTDSFFSMAGM

P57601 MFILGLVSGIT--MSIGTFIITHLYSGSKR--G-----SLL-------LLTDSFFSMSGM

Q8K942 IFILGLVSGIT--MSIGTFIITCLYSGEKR--G-----SQL-------LLTDSFFSMSGM

Q89A60 MFMLGIISGIT--MSIGTYIITNLYTDQTR--A-----SML-------LLTDSFFSMSGI

P76198 AVCVGIANSAL--DTGGYPALMECFP-KAS--G-----SAV-------ILVKAMVSFGQM

O05390 LLGSYALRGLGYPLFAYSFLVWVSYSTSQNILG-----KAV-------GWFWFMFTCGLN

O34864 MYVTYFVKGLGYPLFAYSFLTWVIYRTPQSKLS-----TAV-------GWFWIAYCLGMF

O52717 ILLFYGIRGLAYPLFLYSFIVAIIHNVRSDSSS-----SAL-------GWFWAVYSVGIG

O52718 ILLFYGIRGFAYPLFLYSFIVAIVHNVKSDNAS-----SAI-------GWFWAVYSIGIG

A9MJT5 RFLQGVGAGSC--YVVAFAILRDTLDERRR--A------KV-------LSLLNGITCIVP

A6TG19 RFIQGIAAGSC--YVVAFAILRDTLDDRRR--A------KV-------LSLLNGITCIIP

A0L190 RFVQGVGAGCC--YVVAFAILRDALDDKRR--A------KV-------LSMVNGVTCIIP

P0AEY8 RFLQGISLCFI--GAVGYAAIQESF-EEAV--C-----IKI-------TALMANVALIAP

Q7CP73 RFVQGTSICFI--ATVGYVTVQEAF-GQTK--A-----IKL-------MAIITSIVLVAP

P39386 RAIQGTSICFI--ATVGYVTVQEAF-GQTK--G-----IKL-------MAIITSIVLIAP

Q68WD6 RFIQAFGVSVG--SVIGQSMARDSYQG-AE--L-----SYV-------YAILSPWLLFIP

Q4UMJ9 RFVQAFGVSVG--SVIGQAMARDSYQG-SE--L-----SYV-------YASLSPWLLFIP

Q1RI77 RFVQAFGVSVG--SVIGQAMARDSYQG-SE--L-----SYV-------YASLSPWLLFIP

P32482 RILQACGASAC--LVSTFATVRDIYAGREE--S-----NVI-------YGILGSMLAMVP

P37597 RFVQAVGVCAA--AVIWQALVTDYYPS-QK--V-----NRI-------FAAIMPLVGLSP

P45123 RFVQGFFGAAP--VVLSGALLRDLFS-KDQ--L-----SKV-------MSTITLVFMLAP

P28246 RFFHGLAAAAA--SVVINALMRDIYP-KEE--F-----SRM-------MSFVMLVTTIAP

P31442 SAMQGMGTGVG--GVMARTLPRDLYE-RTQ--L-----RHA-------NSLLNMGILVSP

P37482 ALIGV-GIAIG--NVLLPSLIKHKYPEKP---------GIM-------ISLYTTSMNIFA

P76242 AVIGG-GIALG--NVLLPGLIKRDFPHSV---------ARL-------TGAYSLTMGAAA

P17583 ALLGGVGIGII--QAVMPSVIKRRFQQRT---------PLV-------MGLWSAALMGGG

P0C105 LFVLASGVTILQ-VAANPYVTILGKPETAASRL-----TLT-------QAFNSLGTTVAP

O25788 LFILASGIVCLQ-TAGNPFVTLLSKGKEARN-L-----VLV-------QAFNSLGTTLGP

P11551 LFIIAAGLGCLE-TAANPFVTVLGPESSGHFRL-----NLA-------QTFNSFGAIIAV

P44776 LYILTFGLAFLE-TTANPYILAMGDPQTATRRL-----NFA-------QSFNPLGSITGM

A1A9E1 RFVAGIGCAMIW-VVVESALMCSGTS----RNR-----GRL-------LAAYMMVYYVGT

A9MHY5 RFIAGIGCAMIW-VVVESALMCSGTS----HNR-----GRL-------LAAYMMVYYVGT

A4W8S1 RFIAGVGCAMIW-VVVESALMCSGTS----RNR-----GRL-------LAAYMMVYYVGT

A1JMG4 RFFAGVACALIW-VIVESALLRSGTV----TNR-----GQL-------LAAYMMVYYLGT

Q0TK80 IYLLGLWDGFFA-SLGVTALLAATPALVGRENL-----MQA-------GAITMLTVRLGS

O06473 RGGWGLGNALFI-STALAVIVGVS---VG--GS-----AQA-------IILYEAALGLGI

Q2FI61 AMMLGIAVSPIWVIMLS----SVEEDKRGKQM------------------GYVYFSWLLG

Q5HQE8 AIMLGIAVSPIWVIMLS----SVDERNRGKQM------------------GYVYFSWLLG

Q49WE5 AILLGIAVSPIWVIMLA----SVDENNRGKQM------------------GYVYFSWLLG

Q4L523 AIMLGIAVSPIWVIMLS----SVEEAQRGKQM------------------GYVYFAWLLG

P33026 GVFLSSFGSTANPQMFALAREHADKTGREAVMF-----SSF-------LRAQVSLAWVIG

P31436 GVLLSSFASTANPQMFALAREHADRTGRETVMF-----STF-------LRAQISLAWVIG

Q9S3K0 GVLLSSFGSTANPQLFALAREHADRTGRGAAMF-----SSV-------MRAQISLSWVIG

P31675 GVLLASLANTAMPQLFALAREYADNSAREVVMF-----SSV-------MRAQLSLAWVIG

P31126 ALINCAYSVFATVLKAWFA-DNLSSTSKTKIF-------SI-------NYTMLNIGWTIG

Q58955 RIFTGIFSAFVTPVAGSYIAAIAPKTRLGEYM-------GI-------FNSAITLGFGIG

P02920 GIYLGFCFNAGAPAVEAFIEKVSRRSNF-EF--------GR-------ARMFGCVGWALC

Q4UK37 ILFIISFCSSTQDIVLDAYRIERPTSKEELSMA-----FTF-------SSIGFRLGMLLG

Q1RKF6 ILFIISFCAATQDIVLDAYRIERPTSKKELSLA-----FTF-------GSIGFRLGMLLG

Q4ULW4 LTIALGFLSSTFDIAVDALRIDK-FDQETQTIA-----SAT-------AVFGYRIGMLIT

Q1RI01 LTIALGFLSSTFDIAVDALRIDK-FEEETQGIA-----SAT-------AVFGYRIGMLIT

Q68WQ5 IAFIISFFSATQDTILSALRTEI-VPKELLGFT-----SGI-------YIFGYRVGMLLA

Q4UL88 TALIISFFSAAQDTILSALRTEI-VPKESLGFT-----SGI-------YIFGYRIGMLLA

Q92HQ3 TALIISFFSAAQDTILSALRTEI-VPKESLGFT-----SGI-------YIFGYRVGMLLA

Q1RIL0 ISLIISFFSSMQDTILSAFRTEI-VNKESLGFA-----SGI-------YIFGYRFGMLLA

Q4UMU2 CLVAVAFFSSIYDMLLQSSQMLL-ITNKNWGIS-----EAA-------CTTGFRIGILIA

Q68W71 CLIVLAFFSSIYDMLIQSSQMLL-ITDKNWGIS-----EAA-------CTTGFRIGILIS

Q1RHK8 CLIILAFFSSIYDMLLQSSQMLL-INNKNWGIS-----EAA-------CTSGFRIGILIS

Q1LTM2 SCILSALGGTLFDPPRTALVMKLTRPWERGRFY-----SLL-------MIQDSTCAMVGA

Q2NTK5 SCALSALGGTLFDPPRTALVIKLTRPWERGRFY-----SLL-------MMQDSACSVIGA

P55705 RFLQGACAGYIA--PAQAYGVEVTGGRDRARLF-----AWL-------QVATNVGSLGGA

Q7Z3Q1 FIGAFCGNYTTFWGACFAYIVDQCKEHKQKTIR-----IAI-------IDFLLGLVTGLT

Q05B81 ILCALLGDFSGLLAASFASVAD-VSSSRTRTIR-----MAL-------LEACIGVAGMLA

P76470 TLLRA-AEAGFFPG--MIYLTSQWFPQRNRASI-----MGL-------FYMGAPLALTLG

P32135 ALFGVTCDMMNWPV--LLKSVSRLGNSEQQGRL-----FGF-------FETGRGIVDTVV

P38358 ISVISSILMNYYVPNQKEYNRQNSSIFQNPGKI-----LRD-------IDVMGSILIITG

A4WAE6 RIGIAFAHAIFWSITASLAIRLAPAGKRAQAL------SLL--------ATGTALAMVLG

A6T8Y8 RIGIAFAHAVFWSITSALAIRMAPPGKRAQAL------SLI--------ATGTALAMVFG

Q6CZ44 RIGVALSHAVFWSITASLVVRLAPADKKAQAL------SLL--------ATGTALALVLG

Q888L8 RVGIAFAHAVFWSVTASLAVRIAPPGKQVQAL------GLL--------ATGTSLAMVLG

Q1IB51 RIGIALAHAVFWAITASLAVRVAPPGQQAKAL------GLL--------ATGTTLAMVMG

A6UZY0 RIGIAFAHAVFWAITASLAVRVAPPGQQAKAL------GLL--------ATGTTLAMVLG

Q4QP52 RMCIALAHSVFWSITASLVMRISPKHKKTQAL------GML--------AIGTALATILG

Q9CM87 RIGVALTHSIFWAITASLVIRVAPKDKKSQAI------GLL--------AIGCSLAMILG

O25797 RMGIAFAHSIFWSITASLVIRVAPRNKKQQAL------GLL--------ALGSSLAMILG

Q17YP7 RMGIALAHSVFWSITASLVIRVAPIGRKQQAL------GLL--------ALGSSLAMILG

P77389 RILTSLNHGAFFGLGSVVAASVVPKHKQASAV------ATM--------FMGLTLANIGG

O31577 RILSASIHGAFFAIAMVFASEMVPPEKRAAAA------ASM--------NGGLTVALMLG

O34367 RVISAFSHGVFMSIGSTIAADIVPEDKRASAI------SIM--------FTGLTVATVTG

A1AHK2 RACLGLALGGFWAMSASLTMRLVPPRTVPKAL------SVI--------FGAVSIALVIA

A9MWE8 RACLGLALGGFWAMSASLTMRLVPARTVPKAL------SVI--------FGAVSIALVIA

P23910 RLVSGFPHGAFFGVGAIVLSKIIKPGKVTAAV------AGM--------VSGMTVANLLG

P31141 RVVAALANAGFLAVALTTAAALVPADKQGRAL------AVL--------LSGTTVATVAG

P43531 RALIGLSLSGVAAVGMTYLSEEIHPSFVAFSM------GLY--------ISGNSIGGMSG

Q8K902 RALTGLALSGVVAVAMTYIVEEVHPNSVSFCM------GLY--------ISGNTIGGCSG

P57648 RALTGLALSGVVAVAMTYISEEIHPNSLSFCM------GLY--------ISGNTIGGFLG

Q89A23 RALTGLALSGVAAVAMTYLSEEMHPSVLSFSI------GLY--------ISGNTIGGFLG

P37498 RTIQGVALAGLPSIAMAYLGEEIEPGSLGSAM------GLY--------ISGNAIGAVFG

P0A0J4 RVIGGMSAGMVMPGVTGLIADISPSHQKAKNF------GYM--------SAIINSGFILG

Q07282 RLIAGIT-GATGAVAASTIADVTPEESRTHWF------GMM--------GACFGGGMIAG

P02982 RIVAGIT-GATGAVAGAYIADITDGDERARHF------GFM--------SACFGFGMVAG

P02981 RIVAGIT-GATGAVAGAYIADITDGEDRARHF------GLM--------SACFGVGMVAG

P70187 ISVSGVF-AVTFSVVFAYVADITQEHERSMAY------GLV--------SATFAASLVTS

Q5SR56 ISVSGVF-SVTFSVIFAYVADVTQEHERSTAY------GWV--------SATFAASLVSS

P77726 RALQGS--GAIAAAVMALLSDLTREQNRTKAM------AFI--------GVSFGITFAIA

Q89AA9 RGLQGS--GAISSVCMTLLSELVLPHNRIKIM------GLL--------GVSFGISFFLA

Q8K999 RAIQGS--GAISGVSMALLSDLIRKENRIKSI------SII--------GVSFAVSFLIS

P57538 RFFQGS--GAISGVCMAFLSDLIREENRVKSI------AAI--------GVSFAISFLIA

Q5HIA2 RVLQGFSTGGEYAGAMTYVAESSPDKRRNSLG------SGL--------EIGTLSGYIAA

Q4L3Q4 RILQGFSTGGEYAGAMTYIAEISPDKKRNSLG------SGL--------EIGTLSGYIAA

Q5HRH0 RVLQGFSTGGEYAGAMVYVAESSPDRKRNSLG------CGL--------EIGTLSGYIAA

P0C0L7 KMAQGFSVGGEYTGASIFVAEYSPDRKRGFMG------SWL--------DFGSIAGFVLG

Q47421 KMAQGFSVGGEYTGASIFVAEYSPDRKRGFMG------SWL--------DFGSIAGFVLG

P0A2G3 RLLQGFSAGVELGGVSVYLSEIATPGNKGFYT------SWQ--------SASQQVAIVVA

P16482 RLLQGFSAGAELGGVSVYLAEIATPGRKGFYT------SWQ--------SGSQQVAIMVA

P0AEX3 RLFQGLSVGGEYGTSATYMSEVAVEGRKGFYA------SFQ--------YVTLIGGQLLA

P76350 RAIQGFAVGGEWGGAALLSVESAPKNKKAFYS------SGV--------QVGYGVGLLLS

P41036 RLVIGMGMAGEYGSSATYVIESWPKHLRNKAS------GFL--------ISGFSVGAVVA

Q9SYQ1 RFFLGLGIGGDYPLSATIMSEFANKRTRGAFI------AAV--------FSMQGLGILVS

Q9S735 RFVLGLGIGGDYPLSATIMSEFANKRTRGAFI------AAV--------FSMQGLGILMS

P76230 RFVMGVGLGALLVTLFAGFTEYMPGRNRGTWS------SRV--------SFIGNWSYPLC

P38055 RFLMGTGMGALIMVGYASFTEFIPATVRGKWS------ARL--------SFVGNWS-PML

Q46909 RILIGIGLGGDYSVGHTLLAEFSPRRHRGILL------GAF--------SVVWTVGYVLA

P31679 RVLIGIVIGADYPIATSMITEFSSTRQRAFSI------SFI--------AAMWYVGATCA

O24723 RIISCFGLGAVMPVALTIVADWMPKARRAQMV------SIA--------FAGVGVGSIIG

O30513 RFLAGVGIGGVMPNVIALVSEYAPKKFKSFFV------TLM--------FSGYAIGGMTA

Q43975 RFLTGIGLGAAMPNATTLFSEYCPARIRSLLV------TCM--------FCGYNLGMAIG

Q51955 RFLTGLGLGAGMPNATTLLSEYTPERLKSLLV------TSM--------FCGFNLGMAGG

Q9I6Q3 RFLTGLGLGAAMPNATTLLSEYTPERLKSLLV------TSM--------FCGFNLGMACG

P77589 RLMTGVGLGAALPNLIALTSEAAGPRFRGTAV------SLM--------YCGVPIGAALA

P94131 RFFASLGLGSLYIACNTLMAEYVPTKYRTTVL------GTL--------QAGWTVGYIVA

P71369 RTIAGIGLGGEFGIGMALAAEAWPARHRAKAA------SYV--------ALGWQVGVLGA

O34691 RFVIGMGLGGELPVASTLVSEAVVPEKRGRVI------VLL--------ESFWAVGWLAA

P0AGC0 YALSGFFQSTGGSCSYSTITKWTPRRKRGTFL------GFW--------NISHNLGGAGA

P27669 WALNAFFQGFGSPVCARLLTAWYSRTERGGWW------ALW--------NTAHNVGGALI

P96335 IFLNGWFQGMGWPPCGRTMVHWWSKSERGTIV------SIW--------NTAHNIGGMVP

P08194 LFLCGWFQGMGWPPCGRTMVHWWSQKERGGIV------SVW--------NCAHNVGGGIP

P37948 MFINGWFQGMGWPPCGRTMAHWFSISERGTKM------SIW--------NVAHNIGGGIL

P12681 VVFNGLFQGMGVGPSFITIANWFPRRERGRVG------AFW--------NISHNVGGGIV

Q5M7K3 QILNGLAQTTGWPSVVTCMGNWFGKGKRGFIM------GIW--------NSHTAVGNILG

Q9WU81 QICNGLVQTTGWPSVVTCVGNWFGKGKRGFIM------GIW--------NSHTSVGNILG

Q58CV5 QIFNGLVQTTGWPAVVSCVGNWFGKGKRGLIM------GIW--------NSHTSVGNILG

Q7SY29 QALNGLVQTTGWPAVVACVGNWFGKGKRGFIM------GVW--------NSHTSVGNILG

P57057 QVINGLVQTTGWPSVVTCLGNWFGKGRRGLIM------GVW--------NSHTSVGNILG

Q17QZ3 WIVNGLLQSTGWPCVVAVMGNWFGKAGRGVVF------GLW--------SACASVGNILG

Q3TIT8 WIVNGLLQSTGWPCVVAVMGNWFGKAGRGVVF------GLW--------SACASVGNILG

Q640L2 WIVNGLLQSTGWPCVVAIMGNWFGKSGRGFVF------GLW--------SACASVGNILG

Q5F3N0 WVVNGLLQSTGWPCVVAVMGNWFGKAGRGFVF------GLW--------SACASVGNILG

Q09037 RVLIGLGVGILCSVFPMYVNENAHPKLCKMDG------VLF--------QVFTTLGIMLA

Q06222 RVLMGIGLGVVCVICPMYVNENAHPKLSKVDG------VLF--------QVFITFGIMLA

P13865 RFVIGLFLGVICVACPVYTDQNAHPKWKRTIG------VMF--------QVFTTLGIFVA

P46499 LFICGTSVACIMIVFYAYILEFIEPEQRVFLR------TFFNWVRYNKPDLGVIMGFQGY

P54219 RTLQGIGSSFSSVAGLGMLAS-VYTDDHERGR------AMGT------ALGGLALGLLVG

Q6NT16 RVMDAVSFAAAMTASSSILAK-AFPNN--VAT------VLGS------LETFSGLGLILG

Q8R0G7 RGLVGVGEASYSTIAPTLIADLFVADQRSRML------SIF--------YFAIPVGSGLG

Q5XGK0 RGLVGVGEASYSTIAPTIIADLFLADQRTRML------SFF--------YFATPVGCGLG

Q7ZU13 RGLVGVGEASYSTIAPTIIADLFVKEKRTNML------SIF--------YFAIPVGSGMG

A2CER7 RALVGTGEASYSTIAPTIIGDLFAGSKRTLMI------SFF--------YIFIPVGSGLG

Q9D232 RGFVGTGAASYSTIAPTVLGDLFVKDQRTCAL------AVF--------YIFIPVGSGLG

Q6ZMD2 RGIVGTGSASYSTIAPTVLGDLFVRDQRTRVL------AVF--------YIFIPVGSGLG

Q6GPQ3 RTFVGFGSGNVAVVRSYVAGATSLSERTGAMANISAFQAMG--------FILGPAFQAAL

A5IVG9 GFFLGVG-GAIFSVGVTSVPKYFPKEKVGLAN------GIY---------GMGNIGTAVS

Q5HLK7 GFFLGIG-GAIFSVGVTSVPKYFSKDKVGLAN------GIY---------GVGNIGTAVS

P46907 GFFLGIG-GAVFSIGVTSLPKYYPKEKHGVVN------GIY---------GAGNIGTAVT

P10903 SLLCGFA-GANFASSMANISFFFPKQKQGGAL------GLN--------GGLGNMGVSVM

P37593 ALLCGFA-GANFASSMGNISFFFPKARQGSAL------GIN--------GGLGNLGVSVM

P37758 ALLCGFA-GANFASSMGNISFFFPKAKQGSAL------GIN--------GGLGNLGVSVM

Q9P3K8 FVFIGVGTCSMYMSAVATCAKNFGR---GKHR------GLA--------LAVPIAAFGLS

Q6FWD4 FFIIGVATSGLYFSALITCAKLFPG---TK--------LLS--------ISIPTTCYGLS

Q6CPY8 FAIVGIATSSLYFCALITCAKLYPD---TK--------LLS--------ISFPTTCFGLS

P22152 RFFIGI-LGGTFVPCQVWCTGFFDKSIVGTAN------SLA--------AGLGNAGGGIT

Q02563 RLLSGVGIGGSIPIVFSYFSEFLAQEKR-GEH------LSW-------LCMFWMIGGVYA

Q496J9 RLLSGFGIGGAIPTVFSYFAEVLAREKR-GEH------LSW-------LCMFWMIGGIYA

Q63564 RLISGIGIGGSLPIVFAYFSEFLSREKR-GEH------LSW-------LGIFWMTGGIYA

Q1JP63 RGLVGFGIGG-VPQSVTLYAEFLPMKAR-AKC------ILL-------IEVFWAIGTVFE

Q2XWK0 RGLVGFGIGG-VPQSVTLYAEFLPMKSR-AKC------ILL-------IEIFWALGTVFE

Q1LVS8 RCMVGCGVAA-TSQGFVLKTEFIPAKYR-AYL------LPL-------ASIFWMMGSILI

O08966 RLLQGMVSKGSWVSGYTLITEFVGSGYRRT-T------AIL-------YQVAFTVGLVGL

O15245 RLLQGLVSKGNWMAGYTLITEFVGSGSRRT-V------AIM-------YQMAFTVGLVAL

O77504 RLLQGLVSKGSWMSGYTLITEFVGSGYRRT-V------AIL-------YQVAFSVGLVAL

A7MBE0 RLLQGLVSKGSWTAGYTLITEFVGLGYRRT-V------AIL-------YQMAFTVGLVLL

Q9R0W2 RFLQGLVSKAGWLIGYILITEFVGLGYRRM-V------GIC-------YQIAFTVGLLIL

O02713 RLIQGLVSKAGWMIGYILITEFVGLSYRRT-V------GIF-------YQVAFTFGLLVL

Q8MJI6 RLIQGLVSKAGWLIGYILITEFVGLNYRRT-V------GIL-------YQVAFTVGLLVL

O88446 RFLQGVFGKGAWMTCFVIVTEIVGSKQRRI-V------GIV-------IQMFFTLGIIIL

O75751 RFLQGVFGKGTWMTCYVIVTEIVGSKQRRI-V------GIV-------IQMFFTLGIIIL

Q9U539 RFFTGLAFPALFQIPFIICMEFMGNSGR-IFS------GLM-------TSLFFGAAMALL

O76082 FVLVGMGQISNYVAAFVLGTEILGKSVRIIFS------TLG-------VCIFYAFGYMVL

Q497L8 RFFLVMASSGYFVVVFVYVMEIIGKKAR-TWA------SIH-------LNTFFAIGAMLV

Q86VW1 RFFLAMVASGYLVVGFVYVMEFIGMKSR-TWA------SVH-------LHSFFAVGTLLV

Q17QN9 RFLLAISGSGYLVVVFVYVTEFVGMKSR-TWA------SIH-------LHSFFAFGTMVV

Q95R48 RLMVGATTSGVFLVAYVVAMEMVGPDKR-LYA------GIF-------VMMFFSVGFMLT

Q9Y267 RFGISQSVVGYAISSISLATEWLVGEHR-AHA------IIL-------GHCFFAVGAMLL

Q6A4L0 RFVLATANAGFLLSTNVLISEWVGPSWR-TQA------VVL-------AQSNVALGQMVL

Q9Y226 RFAVATAVAGLSFSNVTLLTEWVGPSWR-TQA------VVL-------AQCNFSLGQMVL

Q8IVM8 RFLSGIAAMSLITNTIMLIAEWATHRFQ-AMG------ITL-------GMCPSGIAFMTL

Q66J52 RFLTGVAFSGIVLNSYSLTVEWIPTGNR-AFT------STA-------TGYCYTMGQLVL

Q91WU2 RMLTGSALAGFTIIVLPLELEWLDVEHR-TVA------GVI-------STTFWTGGVLLL

Q8IZD6 RFLVGMMNGGMSLVAFVLLNECVGTAYW-ALA------GSI-------GGLFFAVGIAQY

Q6DFR1 RFLVGVMNGGMSLVAFVLLNECIGASYW-AAA------GSL-------GSLCFAVGIAQF

Q6NUB3 RFLVGVMNGGMSLVAFVLLNECIGSSYW-AIA------GSL-------GSMCFAVGIAQF

Q28ES4 RFFVGIMNGGMALVSFVLTQEYVGKSYW-ALT------GSL-------TNLIFAVGIAFY

A6NKX4 RLLHGGTLAGALLALYLARLELCDPPHR-LAF------SMG-------AGLFSVVGTLLL

P47185 KIIYGLGAGGCSVLCPMLLSEIAPTDLR-GGL------VSL-------YQLNMTFGIFLG

P13181 RIISGLGVGGIAVLCPMLISEIAPKHLR-GTL------VSC-------YQLMITAGIFLG

P23585 RIISGMGVGGIAVLSPTLISETAPKHIR-GTC------VSF-------YQLMITLGIFLG

Q92339 KILAGVGIGALSVLSPGYQSEVAPPQIR-GAV------VAT-------YQIFSTGAALVA

O74969 KIWTGIGIGALSVLAPGYQSETAPPSIR-GTV------VVT-------YQLFVTGGIFIA

P10870 RVISGIGIGAISAVVPLYQAEATHKSLR-GAI------IST-------YQWAITWGLLVS

Q12300 RVISGIGIGIISAVVPLYQAEAAQKNLR-GAI------ISS-------YQWAITIGLLVS

P42833 RAVTGIGVGVTTVLVPMFLSENSPLKIR-GSM------VST-------YQLIVTFGILMG

P49374 RVISGMGIGFGSSAAPVYCSEISPPKIR-GTI------SGL-------FQFSVTVGIMVL

O74713 RIISGIGVGFGSAVAPVYGAELAPRKIR-GLI------GGM-------FQFFVTLGIMIM

Q9BE72 RIAIGVSISLSSIATCVYIAEIAPQHRR-GLL------VSL-------NELMIVIGILSA

Q5J316 RIAIGVFISLSSTATCVYIAEIAPQHRR-GLL------VSL-------NELMIVIGILFA

Q32NG5 RIFIGVSISLSAIATCVYIAELSPQDKR-GML------VSL-------NELMIVAGILLA

Q6NWF1 RMLVGMSVALSGTASCLYAAEVAPAAWR-GRC------VCV-------YELMVVLGMLLG

Q0P4G6 RVTIGFAISISSMACCIYVSEIVRPHQR-GML------VSL-------YETGITVGILIS

Q6GN01 RVTVGFAISISSMACCIYVSEIVRPHQR-GTL------VSL-------YETGITVGILIS

O95528 RAVVGFAISLSSMACCIYVSELVGPRQR-GVL------VSL-------YEAGITVGILLS

Q8VHD6 RLSVGFAISLSSMACCIYVSELVGPRQR-GVL------VSL-------YEVGITVGILFS

Q3UHK1 RLVVGLGIGIASMTVPVYIAEVSPPNLR-GRL------VTI-------NTLFITGGQFFA

Q96QE2 RLVVGLGIGIASMTVPVYIAEVSPPNLR-GRL------VTI-------NTLFITGGQFFA

Q9C757 RVFVGLGVGMASMTAPLYISEASPAKIR-GAL------VST-------NGFLITGGQFLS

Q8VZR6 RLLVGLGVGVASVTAPVYIAEASPSEVR-GGL------VST-------NVLMITGGQFLS

P30606 RLIMGFGVGIGSLISPLFISEIAPKMIR-GRL------TVI-------NSLWLTGGQLIA

P30605 RLIMGFGVGIGSLIAPLFISEIAPKMIR-GRL------TVI-------NSLWLTGGQLVA

Q10286 RFVIGWGVGIASLIIPLYLSEIAPSKIR-GRL------VII-------YVLLITAGQVIA

Q04162 RLLVGIAVGVSAQCVPLFLSEISPSRIR-GFM------LTL-------NIIAITGGQLVS

Q01440 RVIVGLAIGISSATIPVYLAEVTSPKHR-GAT------IVL-------NNLFLTGGQFVA

P11166 RFIIGVYCGLTTGFVPMYVGEVSPTALR-GAL------GTL-------HQLGIVVGILIA

P46896 RFIIGLYSGLTTGFVPMYVGEVSPTALR-GAL------GTF-------HQLGIVLGILIA

P47843 RLIIGLFCGLCTGFVPMYIGEISPTALR-GAF------GTL-------NQLGIVIGILVA

P14672 RFLIGAYSGLTSGLVPMYVGEIAPTHLR-GAL------GTL-------NQLAIVIGILIA

Q90592 RAITGLYCGLSSGLVPMYVSEVSPTALR-GAL------GTL-------HQLAIVTGILIS

P11168 RSISGLYCGLISGLVPMYIGEIAPTALR-GAL------GTF-------HQLAIVTGILIS

Q5RB09 RFIMGIDGGIALSVLPMYLSEISPKEIR-GSL------GQV-------TAIFICIGVFTG

A4ZYQ5 RVVLGVCAGISYSALPMYLGELAPKNLR-GMV------GTV-------TEVFVIVGVFLA

P22732 RLLVGICAGVSSNVVPMYLGELAPKNLR-GAL------GVV-------PQLFITVGILVA

P58353 RVLVGICAGLSSNVVPMYLGELAPKNWR-GAL------GVV-------PQLFITIGILVA

Q863Y9 RLLVGICAGLSSNVVPMYLGELSPKNLR-GAL------GVV-------PQLFITVGILVA

P43427 RLLVGICAGISSNVVPMYLGELAPKNLR-GAL------GVV-------PQLFITVGILVA

Q9WV38 RLLVGICAGISSNVVPMYLGELAPKNLR-GAL------GVV-------PQLFITVGILVA

P15686 RVLLGFGVGLGSQVVPQYLSEVAPFSHR-GML------NIG-------YQLFVTIGILIA

Q39525 RVLLGFGVGLGSQVVPQYLSEVAPFSHR-GML------NIG-------YQLFVTIGILIA

Q94AZ2 RILLGCGVGFANQAVPLFLSEIAPTRIR-GGL------NIL-------FQLNVTIGILFA

Q10710 RIMLGVGIGFGNQAVPLYLSEMAPTHLR-GGL------NIM-------FQLATTSGIFTA

Q41144 RILLGFGIGFANQSVPLYLSEMAPYKYR-GAL------NIG-------FQLSITIGILVA

P23586 RILLGFGIGFANQAVPLYLSEMAPYKYR-GAL------NIG-------FQLSITIGILVA

O65413 RLLLGFGIGFTNQSVPLYLSEMAPYKYR-GAL------NIG-------FQLSITIGILVA

Q9SX48 RLLLGVGVGFANQSTPVYLSEMAPAKIR-GAL------NIG-------FQMAITIGILIA

Q9LT15 RLLLGVGVGFANQSTPVYLSEMAPAKIR-GAL------NIG-------FQMAITIGILVA

Q9FMX3 RLFLGVGVGFANQSVPLYLSEMAPAKIR-GAL------NIG-------FQLAITIGILAA

Q9SBA7 RILLGFGVGFGNQAVPLFLSEIAPARLR-GGL------NIV-------FQLMVTIGILIA

Q9SFG0 RLFLGFGVGFGNQAVPLFLSEIAPAQLR-GGL------NIV-------FQLMVTIGILIA

Q8L7R8 RLLLGVGVGFANQSVPLYLSEMAPAKYR-GAI------SNG-------FQLCIGIGFLSA

Q93Y91 RILLGFGVGFTNQAAPVYLSEVAPPRWR-GAF------NIG-------FSCFISMGVVAA

P0AE24 RVVLGIAVGIASYTAPLYLSEMASENVR-GKM------ISM-------YQLMVTLGIVLA

P0AEP1 RVLLGLAVGVASYTAPLYLSEIAPEKIR-GSM------ISM-------YQLMITIGILGA

P96710 RIIGGLGIGMGSSLSVTYITEAAPPAIR-GSL------SSL-------YQLFTILGISAT

P54723 RFLLGLAVGCASVTVPTFLAEISPAERR-GRI------VTQ-------NELMIVIGQLLA

O34718 RFVLGIAVGGASVTVPAYLAEMSPVESR-GRM------VTQ-------NELMIVSGQLLA

P46333 RVILGLAVGGSTALVPVYLSEMAPTKIR-GTL------GTM-------NNLMIVTGILLA

O52733 RIILGMAVGAASALIPTYLAELAPSDKR-GTV------SSL-------FQLMVMTGILLA

P0AGF4 RIIGGIGVGLASMLSPMYIAELAPAHIR-GKL------VSF-------NQFAIIFGQLLV

P21906 RFLAGLGIGVVSTLTPTYIAEIAPPDKR-GQM------VSG-------QQMAIVTGALTG

P15729 RVLGGIGVGAASVIAPAYIAEVSPAHLR-GRL------GSL-------QQLAIVSGIFIA

Q6AWX0 RVIYGVSVGLAMHAAPMYIAETAPSPIR-GQL------VSL-------KEFFIVLGMVGG

Q93YP9 RLLEGFGVGIISYTVPVYIAEIAPQTMR-GAL------GSV-------NQLSVTIGIMLA

Q94AF9 RISLGIGVGLISYVVPVYIAEITPKHVR-GAF------TAS-------NQLLQNSGVSLI

O04036 RLLLGIGVGIFSYVIPVYIAEIAPKHVR-GSF------VFA-------NQLMQNCGISLF

Q94KE0 RLSTGFAVGLLSYVIPVYIAEITPKHVR-GAF------VFA-------NQLMQSCGLSLF

Q9SCW7 RGFLGFGVGLISYVVPVYIAEITPKAFR-GGF------SFS-------NQLLQSFGISLM

Q4F7G0 RLFLGFGVGLISYVVPVYIAEITPKTFR-GGF------SYS-------NQLLQCLGISLM

Q8LBI9 RFFTGYGIGVFSYVVPVYIAEISPKNLR-GGL------TTL-------NQLMIVIGSSVS

P93051 RLATGYGMGAFSYVVPIFIAEIAPKTFR-GAL------TTL-------NQILICTGVSVS

Q0WQ63 RFLTGYGCGTLSFVVPVFIAEISPRKLR-GAL------ATL-------NQLFIVIGLASM

Q3ECP7 RFLVGYGMGVFSFVVPVYIAEITPKGLR-GGF------TTV-------HQLLICLGVSVT

Q9LTP6 RLFLGVAAGVASYVVPVYIVEIAPKKVR-GTF------SAI-------NSLVMCASVAVT

Q8GXK5 RLLQGISVGISSYLGPIYISELAPRNLR-GAA------SSL-------MQLFVGVGLSAF

Q9JJZ1 RLLTGLACGVASLVAPVYISEIAYPAVR-GLL------GSC-------VQLMVVTGILLA

P58354 RLLTGLACGIASLVAPVYISEIAYPEVR-GLL------GSC-------VQLMVVTGILLA

Q9NY64 RLLTGLACGVASLVAPVYISEIAYPAVR-GLL------GSC-------VQLMVVVGILLA

Q9UGQ3 RTLTGFAGGLTAACIPVYVSEIAPPGVR-GAL------GAT-------PQLMAVFGSLSL

P43562 RMIKGMILGNFSILVASYANEVIPRGKR-GST------MSY-------IQLCLTIGILVM

Q9FYG3 RFLVGTGMGLGPPVAALYVTEVSPAFVR-GTY------GSF-------IQIATCLGLMAA

P36035 RWITGIAMGGIYGCASATAIEDAPVKAR-SFL------SGL-------FFSAYAMGFIFA

P47186 QALCGMPWGCFQCLTVSYASEICPLALR-YYL------TTY-------SNLCWLFGQLFA

A6QLI1 RILQGLVEGVTYPACHGIWSKWAPPLERSRLA------TTS-------FCGSYAGA-VIA

Q5W8I7 RILQGLVEGVTYPACHGIWSKWAPPLERSRLA------TTS-------FCGSYAGA-VVA

A4FV52 RILQGLVEGVTYPACHGIWSKWAPPLERSRLA------TTA-------FCGSYAGA-VVA

P34644 QITQGLVQGVCYPAMHGVWRYWAPPMERSKLA------TTA-------FTGSYAGA-VLG

Q66GI9 RAMVGVAEGVALPCMNNMVARWFPPTERSRAV------GIA-------MAGFQLGN-VVG

Q9FKV1 RLLVGVAQGFIFPSIHTVLAQWVPPHERSRLV------SIT-------TSGMYLGA-ALG

Q46916 RFLVGLAEAPSFPGNSRIVAAWFPAQERGTAV------SIF-------NSAQYFAT-VIF

Q91Y77 GVVFACGCSFAYQPSLVILGHYF-KKRLGLVN------GIV-------TAGSSVFT-ILL

Q8TF71 GIIFACGCSFAYQPSLVILGHYF-KKRLGLVN------GIV-------TAGSSVFT-ILL

A1L1W9 GIVFACGCSFAYQPSLVILGHYF-KRRLGLVN------GIV-------TAGSSVFT-ITL

P36021 GILFGCGCSFAFQPSLVILGHYF-QRRLGLAN------GVV-------SAGSSIFS-MSF

O35308 GVLTGLGLALNFQPSLIMLGLYF-ERRRPLAN------GLA-------AAGSPVFL-SML

O95907 GVLTGLGLALNFQPSLIMLGLYF-ERRRPLAN------GLA-------AAGSPVFL-SAL

Q90632 GVLTGLGMALNFQPSLIMLGTYF-DKRRPLAN------GLA-------AAGSPVFL-SSL

O35910 GVITGLGLALNFQPSLIMLNRYF-NKRRPMAN------GLA-------AAGSPVFL-CAL

O15427 GVITGLGLALNFQPSLIMLNRYF-SKRRPMAN------GLA-------AAGSPVFL-CAL

P57788 GVITGLGLALNFQPSLIMLNRYF-DKRRPLAN------GLS-------AAGSPVFL-CAL

O15375 GFITGLGMCFSFQSSITVLGFYF-VRRRVLAN------ALA-------SMGVSLGI-TLW

Q6ZSM3 GVLTGLGFALCYSPAIAMVGKYF-SRRKALAY------GIA-------MSGSGIGT-FIL

Q8BGC3 GVLTGLGFALCYSPAIAMVGKYF-SRRKALAY------GIA-------MSGSGIGT-FIL

Q503M4 GLLTGLGFALCYTPAIAMVGIYF-CERKALAY------GIA-------MSGSGIGT-FIL

O15403 GIISGLGYCFSFLPTVTILSQYF-GKRRSIVT------AVA-------STGECFAV-FAF

Q8NCK7 GLLAGFGWALVFAPALGTLSRYF-SRRRVLAV------GLA-------LTGNGASS-LLL

Q7RTY0 GLLSGSGWALTFAPTLACLSCYF-SRRRSLAT------GLA-------LTGVGLSS-FTF

Q5R5M4 GIVVGLGCGLLYTATVTITCLYF-DDRRGLAL------GLI-------STGSSVGL-FIY

Q7TM99 GIVVGLGCGLLYTATVTITCQYF-DSRRGLAL------GLI-------STGSSVGL-FIY

Q5ZJU0 GIVVGLGCGLLYNATVTITCQYF-DKRRGLAL------GLI-------STGSSVGL-FIY

O15374 GLLPGLGSAFLYQVAAVVTTKYF-KKR--LAL------STA-------IARSGMGLTFLL

Q08777 AIVCGFGNGIVLSPLVSVPAHYF-FKRRGTAL------AMA-------TIGGSVGG-VVF

Q08268 SVCSGLGTGILMTPLIGTVATWF-LKRRGIAT------SIS-------TMGGSIGG-IVF

P39709 RFFIGAFEAPSYLAYQYLFGSFYKHDEMVRRS------AFY-------YLGQYIGI-LSA

P25621 RFFQALFESCTFSGTHFVLGSWYKEDELPIRS------AIF-------TGSGLVGS-MFS

O13880 RFFLGVAESGLWPGLAYYMSRWYRGKHLGKRI------GWY-------YTAAQIAA-AAV

P40445 RCLLGMVQGGFIPDNILYLSYYYTGAELTFRL------SFF-------WCAIPLFQ-ILG

P15365 RVLLGCAESVVTPCFTIITAQYWKTEEQFTRV------SIW-------FGMNGLGS-ILI

Q07904 RTLLGLFESSSAVGCIAISGMYYTKSEQSARI------GFW-------ATQAGTGY-IVG

P53322 RLLLGAFEGMIYPAINMYLSVCYRREQYALRF------AFV-------FSAACLSS-SFG

P32071 RVIAGFFAAPALSTGGASYGDFIAMHYYSIAL------GVW--------SIFAVAGPSIG

P38124 RFISGILCSPSLATGGGTVADIISPEMVPLVL------GMW--------SAGAVAAPVLA

Q06451 RFLCGVWSSSGLCLVGGSIADMFPSETRGKAI------AFF--------AFAPYVGPVVG

P53283 RFLCGVWSSSGLCLVGGSIADMFPSETRGKAI------AFF--------AFAPYVGPVVG

Q9C0R8 RLIDGIAFSAPMTLIGGSLADIWEGPERGTAM------AIF--------SAAPFLGPVCG

Q9C0Q6 RLIDGTAFSAPMTLIGGSLADIWEGPERGTAM------AVF--------SAAPFLGPVCG

Q9HF77 RFFAGFIGAAPLVVAPAVMADMFNNRYRGTAI------AIF--------SMLLFGGPMLA

Q5A0E9 RFFSGFVGSAAFVVSPAIFSDLFSTEQRGTAI------STF--------AGVLFGGPMLA

Q07824 RFFGGFIGAAPMAVVPAAFADMFDTNVRGKAI------ALF--------SLGVFVGPILS

O59698 RFFGGYGACVPLCVVAAAFADMYPNRYRGTAI------TIF--------AAVIFVGPLVA

O74829 RFFCGFFGSSPITTVAGSFSDMFSARTRGLVI------AVY--------SAIIFNGPLMS

P38776 RFLSGFFGSAFLSVAGGAIADIFDKDQIGIPM------AIY--------TTSAFLGPSLG

Q9HDX4 RFFSGFFGSVGLGIGAGSLSDMFSKRDRGKYI------GIY--------FLGICLGPAIA

P38227 RMLCGAASASVQSVGAGTVADLYISE--DRGK------NLS-------YYYLGPLLAPLL

P38125 RIFQAFASSSVISLGAGTVTDVVPPK--HRGK------AIA-------YFMMGPNMGPII

P40474 RCLQAAGISPVIAINSGIMGDVTTRA--ERGG------YVG-------YVAGFQVLGSAF

O94607 YILYICGQTGL--GLLSQLIIADTSSLLNRGI------LSA-------IPELPYLATVWI

Q9HE13 RAVQGIGGGGI--MSLVTIVIADITPLQTRPY------YTG-------CMGVTWGVASVM

Q04301 RAICGIGAGGL--NAISSIAVSDICTARERGV------YQG-------YANIVFGFGQLL

P13090 RAFQGLGIAFVLPNVLGIIGNIYVGGTFRKNI------VIS-------FVGAMAPIGATL

Q08902 RAFQGMGPAFLLPNAIAILGRTYKPGR-RKNM------VFS-------LFGASAPGGFFL

P39886 RVLQGLFGALMQPSALGLLRVTFPPG--KLNM------AIG-------IWSGVVGASTAA

P76269 RVIQGFGGAALMSVNTALIRLIYPQR--FLGR------GMG-------INSFIVAVSSAA

Q8Y9K8 RVVQAVGTALLLPLMFNTILLIFPEH--KRGS------AMG-------MIGLVIMFAPAV

P28873 PFFGS-----ILTVKA--------------------------SWRWTF---WFMCIISGF

A4WFG6 IF-PMVAAVLLAR--SI-------------------------EWYWVY---ACIGLVYVA

A1JSB0 IF-PVAAAILLAR--HI-------------------------EWYWVY---ACIGLLYVG

A8GKP6 IF-PIVAAMLLAR--QI-------------------------GWYWVY---ACIGLLYVG

P57601 IF-PIVTAYLLEK--KI-------------------------IWYWSY---ICIGAIYLL

Q8K942 IF-PIISAYLLDR--KI-------------------------LWYWIY---VFLGIIYFL

Q89A60 IF-PIITALIISN--NM-------------------------KWYWVY---FIIGIIYLI

P76198 FY-PMLVSYMLLN--NI-------------------------WYGYGL---IIPGILFVL

O05390 VLGPFYSSYAVPAFGEI-------------------------NTLWSA---LLFVAAGGI

O34864 VFGAWYSSYAIKAFGYL-------------------------NTLWSS---IFWVCLGAF

O52717 VFGSYIPSFTIPHIGEM-------------------------GTLWLA---LLFCATGGI

O52718 VFGSYIPSFTIPHIGEM-------------------------GTLWLA---LAFCLTGGV

A9MJT5 VLAPVLGHLIML---RF-------------------------PWQSLF---YTMSAMGII

A6TG19 VLAPVLGHLIML---KY-------------------------PWQSLF---YTMTGMGVM

A0L190 VIAPVIGHLIML---RF-------------------------PWPSLF---YTMAVMGLL

P0AEY8 LLGPLVGAAWIH---VL-------------------------PWEGMF---VLFAALAAI

Q7CP73 VIGPLSGAALMH---FV-------------------------HWKVLF---GIIAVMGLL

P39386 IIGPLSGAALMH---FM-------------------------HWKVLF---AIIAVMGFI

Q68WD6 SLGSYIGGYIIE---YS-------------------------SWHYVF---VFFSLIGTI

Q4UMJ9 SLGSSIGGYIIE---YS-------------------------SWHYVF---VFFSLTGTI

Q1RI77 SLGSSIGGYIIE---YS-------------------------SWHYTF---VFFSLTGTV

P32482 AVGPLLGALVDM---WL-------------------------GWRAIF---AFLGLGMIA

P37597 ALAPLLGSWLLV---HF-------------------------SWQAIF---ATLFAITVV

P45123 LVAPIIGGYIVK---FF-------------------------HWHAIF---YVISLVGLL

P28246 LMAPIVGGWVLV---WL-------------------------SWHYIF---WILALAAIL

P31442 LLAPLIGGLLDT---MW-------------------------NWRACY---LFLLVLCAG

P37482 ALASGVSVPLATQMNG--------------------------GWKQAF---LLWGGLALL

P76242 ALGSAMVVPLA--LNGF-------------------------GWQGAL---LMLMCFPLL

P17583 GLGAAIT-PWLVQ-HSE-------------------------TWYQTL---AWWALPAVV

P0C105 VFGAVLILSA---ATDATV------------------NAEADAVRFPY---LLLALAFTV

O25788 IFGSLLIFSTTKMGDNASL---------------IDKLADAKSVQMPY---LGLAVFSLL

P11551 VFGQSLILSNVPHQSQDVLDKM------SPEQLSAYKHSLVLSVQTPY---MIIVAIVLL

P44776 FVASQLVLTNLESDKRDAAGNLIFHTLSEAEKMSIRTHDLA-EIRDPY---IALGFVVVA

A1A9E1 FLGQLLVSKVSTELMSV------------------------------L---PWVTGLTLA

A9MHY5 FLGQLLVSKVSGELLHV------------------------------L---PWVTGMILA

A4W8S1 VLGQLMISKLPTDLMSV------------------------------L---PWVTGMVLA

A1JMG4 VTGQLLLGVVSTQLLSV------------------------------I---PWVSALVIT

Q0TK80 VISPMIGGLLLA-TGGV-------------------------AWN--Y---GLAAAGTFI

O06473 SVGPLAGGEL----GSI-------------------------SWRAPF---FGVSVLMFI

Q2FI61 -LLVGMVFMNLL--IKV--------------------------HPTRF---AFMMSLVVL

Q5HQE8 -LLVGMVIMNLL--IKF--------------------------HPTRF---AFLMALVVL

Q49WE5 -LLVGMVGMNVI--FKF--------------------------HPTQF---AFLMSLVVL

Q4L523 -LLVGWAFMNVL--VKL--------------------------HPTRF---AFMMSLVVV

P33026 PPLAYALAMGFS--FTV--------------------------MYLSA---AVAFIVCGV

P31436 PPLAYELAMGFS--FKV--------------------------MYLTA---AIAFVVCGL

Q9S3K0 PPVAFALALGFG--FPA--------------------------MYLTA---AVVFVLCGL

P31675 PPLAFMLALNYG--FTV--------------------------MFSIA---AGIFTLSLV

P31126 PPLGTLLVMQ-S--INL--------------------------PFWLA---AICSAFPML

Q58955 PFIGGILADMYG--IKM--------------------------PFYFC---GFLGILAAI

P02920 ASIVGIMFTINN--QFV--------------------------FWLGS---GCALILAVL

Q4UK37 SVGALYLSIIFG--WNT--------------------------VYKFA---LFITVVGPI

Q1RKF6 SVGALYISVIFD--WST--------------------------VYKLA---ICITFLGPM

Q4ULW4 GAGALYLAEITGNNWQL--------------------------TFVII---AIIFAVATI

Q1RI01 GGWALYFAEITNDNWQL--------------------------TFFVI---GIIFAVSTI

Q68WQ5 SSGAIYLSIYLT--FNK--------------------------IYQIF---ACVIFVYLI

Q4UL88 GSGAIYLSIYFT--FNE--------------------------IYKIF---AGLVFIYLI

Q92HQ3 GSGAIYLSIYLT--FNE--------------------------IYKIF---AGLVFIYLI

Q1RIL0 NSGAIYLSIYLT--FNE--------------------------IYKIF---AILIFIYLI

Q4UMU2 GSGALYLSTIIS--WQD--------------------------VYRSM---AILCIPSLL

Q68W71 GSGALYLSTIIS--WQD--------------------------VYRTM---AILCIPSLL

Q1RHK8 GSGALYLSTIIS--WQE--------------------------VYRTM---AILCVPSLL

Q1LTM2 LLGSWLLQ-Y--------------------------------NFKLVC---LAGALLFLF

Q2NTK5 LLGSWLLR-Y--------------------------------DFKLVC---LAGAVLFVF

P55705 FLGGLILDAL--------------------------------PFAAVN---LTAGVICAF

Q7Z3Q1 GLSSGYFIRE-----LGFE-----------------------WSFLII---A--VSLAVN

Q05B81 SFIGGFLLQE-----QVYV-----------------------NPFWLA---L--AVLTVM

P76470 SPLSGALLEMHG--FMGHP-----------------------GWFWMF---VIEGLLAVG

P32135 AFSALAVFTWFGSGLLGFK-----------------------AGIWFY------SLIVIA

P38358 LTLQLLYLSLGCSTSKL-------------------------SWTSPS---VLLLLVGSV

A4WAE6 LPIGR-----IVG------------------------QYF--GWRTTF---FAIGLGALI

A6T8Y8 IPIGR-----IIG------------------------QYF--GWRMTF---LAIGLGALA

Q6CZ44 LPLGR-----VVG------------------------QYL--GWRVTF---VLIGLIAAV

Q888L8 IPLGR-----VLG------------------------EAL--GWRTTF---LGIAGVAAL

Q1IB51 IPLGR-----VVG------------------------EAL--GWRITF---LCIAGVALA

A6UZY0 IPLGR-----VVG------------------------EAL--GWRTTF---MAIAGLSVL

Q4QP52 LPIGR-----IVG------------------------QLV--GWRVTF---GIIAVLALS

Q9CM87 LPLGR-----LIG------------------------QFF--GWRATF---AIIALIAIG

O25797 LPLGR-----IIG------------------------QIL--DWRSTF---GVIGGVATL

Q17YP7 LPLGR-----IIG------------------------QML--DWRSTF---GMIGGVALL

P77389 VPAAT-----WLG------------------------ETI--GWRMSF---LATAGLGVI

O31577 VPFGS-----YLG------------------------DVL--NWRAVF---SIITALGVI

O34367 VPFGT-----FIG------------------------QQF--GWRFAF---MVIIAVGII

A1AHK2 APLGS-----FLG------------------------ELI--GWRNVF---NAAAAMGVL

A9MWE8 APLGS-----FLG------------------------GII--GWRNVF---NAAAVMGVL

P23910 IPLGT-----YLS------------------------QEF--SWRYTF---LLIAVFNIA

P31141 VPGGS-----LLG------------------------TWL--GWRATF---WAVAVCCLP

P43531 RLISG-----VFT------------------------DFF--NWRIAL---AAIGCFALA

Q8K902 RILSS-----ILA------------------------EYF--SWHIAF---IVIGFFSLM

P57648 RLLSS-----ILA------------------------EKF--SWSISL---MVIGLFSFI

Q89A23 RFLSS-----LFS------------------------EYF--SWNIAL---EFISFLAFT

P37498 RIVSG-----LLS------------------------EYL--NWHMAM---GTIGVISLI

P0A0J4 PGIGG-----FMA------------------------EV---SHRMPF---YFAGALGIL

Q07282 PVIGG-----FAG------------------------QL---SVQAPF---MFAAAINGL

P02982 PVLGG-----LMG------------------------GF---SPHAPF---FAAAALNGL

P02981 PVAGG-----LLG------------------------AI---SLHAPF---LAAAVLNGL

P70187 PAIGA-----YLG------------------------QMYGDSLVVVL---ATAIALLDI

Q5SR56 PAIGA-----YLS------------------------ASYGDSLVVLV---ATVVALLDI

P77726 MVLGP-----IIT------------------------HKL--GLHALF---WMIAILATT

Q89AA9 VILSP-----IIV------------------------NMF--GFYCLF---LINSLLSIF

Q8K999 VVSAP-----IIA------------------------ENF--GFFSIF---WISAVFSIF

P57538 VVSGP-----IIV------------------------HYF--GFFSIF---WISAFLSIV

Q5HIA2 SIMIA-----VLTFFLTDE------------------QMASFGWRIPF---LL-GLFLGL

Q4L3Q4 SIMIA-----LLSFFLSDA------------------QMEAWGWRIPF---IL-GLFLGL

Q5HRH0 SILVF-----ALNISLTET------------------QLNSWGWRIPF---LL-GMFLGL

P0C0L7 AGVVV-----LISTIVGEA------------------NFLDWGWRIPF---FI-ALPLGI

Q47421 AGVVV-----LISTLIGEQ------------------AFLAWGWRLPF---FL-ALPLGL

P0A2G3 ALIGY-----SLNITLGHD------------------AISEWGWRIPF---FI-GCMIIP

P16482 AAMGF-----ALNAVLEPS------------------AISDWGWRIPF---LF-GVLIVP

P0AEX3 LLVVV-----VLQHTMEDA------------------ALREWGWRIPF---AL-GAVLAV

P76350 TGLVS-----LISMMTTDE------------------QFLSWGWRIPF---LF-SIVLVL

P41036 AQVYS-----LVVPV--------------------------WGWRALF---FI-GILPII

Q9SYQ1 SAVTM-----AVCVAFKRSGGGLE-------VDAAAPTEADLAWRLIL---MIGALPAAL

Q9S735 SAVTM-----VVCLAFKNAGEGSSEKTNVAGLETLAPPESDIAWRLIL---MIGALPAAL

P76230 SLIAM-----GLTPLI----------------------SAEWNWRVQL---LIPAILSLI

P38055 S-AAI-----GV--VV----------------------IAFFSWRIMF---LLGGIGILL

Q46909 SIAGH-----HF--IS----------------------ENPEAWRWLL---ASAALPALL

P31679 DLVG------YW--LY----------------------DVEGGWRWML---GSAAIPCLL

O24723 AYLAA-----AV--------------------------IPTLGWQVMV---LIAGLAPLI

O30513 AFLGS-----IL--------------------------VPLYGWKIMF---MIAG-IPLV

Q43975 GFISS-----WL--------------------------IPAFGWHSLF---LLGGWAPLI

Q51955 GFISA-----KM--------------------------IPAYGWHSLL---VIGGVLPLL

Q9I6Q3 GFVSA-----KL--------------------------IPLFGWHSLL---LLGGLLPLV

P77589 ATLGF-----AG--------------------------ANL-AWQTVF---WVGGVVPLI

P94131 TLLAG-----WL--------------------------IPDHGWRVLF---YV-AIIPVL

P71369 ALLTP-----LL--------------------------LPHIGWRGMF---LV-GIFPAF

O34691 ALISY-----FV--------------------------IPSFGWQAAL---LL-TALTAF

P0AGC0 AGVALFGANYLFDGH----------------------------VIGMF---IFPSIIALI

P27669 -PLVMAAVALHYG-----------------------------WRVGMM---V-AGLLAIG

P96335 GAMVLLASAIFFSTHGIEA------------------QAKDVWQQSLY---F-PGIAAMI

P08194 PLLFLLGMAWF----------------------------ND-WHAALY---M-PAFCAIL

P37948 APLVTLGIAMFVT-----------------------------WKSVFF---F-PAIIAII

P12681 APIVGAAFAILGSEH---------------------------WQSASY---IVPACVAVI

Q5M7K3 SLIAGAFVSTAWG--------------------------------LSF---IVPGIIIAA

Q9WU81 SLIAGVWVNQHWG--------------------------------LSF---IVPGIITAI

Q58CV5 SLLAGVWVDQQWG--------------------------------LSF---VVPGVITAI

Q7SY29 SLIAGVYVSSAWG--------------------------------LSF---IVPGIIIAS

P57057 SLIAGYWVSTCWG--------------------------------LSF---VVPGAIVAA

Q17QZ3 ACLASSVLQYGYE--------------------------------YAF---LVTAAVQFA

Q3TIT8 AFLASSVLQYGYE--------------------------------YAF---LVTASVQFA

Q640L2 AFLASSVLKYGYE--------------------------------YAF---LVTASVQFA

Q5F3N0 AFLASCVLKYGYE--------------------------------YAF---LVTASVQFA

Q09037 AMLGL-----ILDKTGASKEE---------------AN--MAGRLHVF---SAVPLGLSV

Q06222 AMLGL-----ILDKTVNYDND---------------PD--MAGRFHGF---CAVSSVLSV

P13865 ALMGL-----ALGQSIRFDHD---------------GDQKVMARMQGL---CVFSTLFSL

P46499 ARLVF-----TL---VCF----------------------ICGYWRSA---AIATSLLAL

P54219 APFGS-----VM-----YE---------------------FVGKSAPF---LILAFLALL

Q6NT16 PPVGG-----FL-----YQ---------------------SFGYEVPF---IVLGCVVLL

Q8R0G7 YIAGS-----KVKDVAG-------------------------DWHWAL---RVTPGLGVL

Q5XGK0 YIVGS-----EMTSAAG-------------------------DWHWAL---RVTPGLGLL

Q7ZU13 YIVGS-----KVDTVAK-------------------------DWHWAL---RVTPGLGLL

A2CER7 YIIGA-----TVADATG-------------------------DWRWAL---RVSPALGGL

Q9D232 YVLGS-----TVAELTG-------------------------NWRWAL---RLMPCLDAM

Q6ZMD2 YVLGS-----AVTMLTG-------------------------NWRWAL---RVMPCLEAV

Q6GPQ3 SVIGE-----TGITINGIS--------------------LQVNMYTAP---ALMGALLGI

A5IVG9 SFLAP-----PIAGIIGWQ-------------------------TTVR---SYLIIIALF

Q5HLK7 SFCAP-----VLAGAIGWQ-------------------------NTVR---SYLIILSIF

P46907 TFAAP-----VIAQAVGWK-------------------------STVQ---MYLILLAVF

P10903 QLVAPLVVSLSIFAVFGSQGV------------KQPDGTELYLANASW---IWVPFLAIF

P37593 QLIAPLVIFLPIFTFLGVQGV------------PQPDGSLLALTNAAW---IWVPLLAVA

P37758 QLVAPLVIFVPVFAFLGVNGV------------PQADGSVMSLANAAW---IWVPLLAIA

Q9P3K8 GMWQS-----QLGSRVFYERF------------ADGTKGDLDVFHFFI---FLGVLLFVV

Q6FWD4 SLIGS-----QF-LRVKY--F------------HPVDYPYLDLGRVFK---AFAWIYTVI

Q6CPY8 SVIGL-----QV-IKLPW--F------------HSKEDGYLDLAVVFK---SFAVFYTFV

P22152 YFVMP-----AIFDSLIRDQG------------LPAHKAW-RVAYIVP---FILIVAAAL

Q02563 AAMAW-----AIIPHYGWSF-------------QMGSAYQFHSWRVFV---LVCAFPSVF

Q496J9 SAMAW-----AIIPHYGWSF-------------SMGSAYQFHSWRVFV---IVCALPCVS

Q63564 SAMAW-----SIIPHYGWGF-------------SMGTNYHFHSWRVFV---IVCALPATV

Q1JP63 VVLAV-----FVMPSLG--------------------------WRWLL---ILSAVPLLL

Q2XWK0 VLLAI-----FVMPTLG--------------------------WRWLL---ILSALPLML

Q1LVS8 IVLGM-----TVVPTMG--------------------------WRWMI---RFSVIPSLV

O08966 AGVAY-----AIP-----------------------------DWRWLQ---LAVSLPTFL

O15245 TGLAY-----ALP-----------------------------HWRWLQ---LAVSLPTFL

O77504 SGVAY-----AIP-----------------------------NWRWLQ---LTVSLPTFL

A7MBE0 SGLAY-----ILP-----------------------------HWRWLQ---LAVSLPIFL

Q9R0W2 AGVAY-----VIP-----------------------------NWRWLQ---FAVTLPNFC

O02713 AGVAY-----ALP-----------------------------HWRWLQ---FTVTLPNFC

Q8MJI6 AGVAY-----ALP-----------------------------RWRWLQ---LTVTLPYFC

O88446 PGIAY-----FTP-----------------------------SWQGIQ---LAISLPSFL

O75751 PGIAY-----FIP-----------------------------NWQGIQ---LAITLPSFL

Q9U539 GVVAM-----FIR-----------------------------RWRQLT---FFCNAPFAF

O76082 PLFAY-----FIR-----------------------------DWRMLL---VALTMPGVL

Q497L8 ALASY-----LLK-----------------------------TWWLYQ---IILCIVTTP

Q86VW1 ALTGY-----LVR-----------------------------TWWLYQ---MILSTVTVP

Q17QN9 ALTGY-----FVR-----------------------------TWWIYQ---IVLSSVTVP

Q95R48 AVFAY-----FVH-----------------------------DWRWLQ---IALTLPGLI

Q9Y267 TGIAY-----GLP-----------------------------HWQLLF---LVGGILVIP

Q6A4L0 AGLAY-----GVR-----------------------------NWRLLQ---ITGTAPVLL

Q9Y226 AGLAY-----GFR-----------------------------NWRLLQ---ITGTAPGLL

Q8IVM8 AGLAF-----AIR-----------------------------DWHILQ---LVVSVPYFV

Q66J52 VGLAF-----IIR-----------------------------DWQWLQ---LAASIPFFF

Q91WU2 TLVGY-----LIR-----------------------------SWRWLL---LAATLPCVP

Q8IZD6 ALLGY-----FIR-----------------------------SWRTLA---ILVNLQGTV

Q6DFR1 ALIGY-----FIR-----------------------------SWRLLA---LLVNVQGAA

Q6NUB3 ALIGY-----FIR-----------------------------SWRLLA---VLVNVQGAG

Q28ES4 ALLGF-----YIR-----------------------------NWRTLA---FVANSPGIF

A6NKX4 PGLAA-----LVQ-----------------------------DWRLLQGLGALMSGLLLL

P47185 YCSVY-----GTRKY-----------------------SNTAQWRIPV---GLCFLWALI

P13181 YCTNY-----GTKSY-----------------------SNSVQWRVPL---GLCFAWSLF

P23585 YCTNY-----GTKDY-----------------------SNSVQWRVPL---GLNFAFAIF

Q92339 ACINM-----GTHKL-----------------------RKTASWRTSF---GINMLWGIL

O74969 ACINM-----GTHKL-----------------------HKTAQWRVSI---GINLLWGII

P10870 SAVSQ-----GTHAR-----------------------NDASSYRIPI---GLQYVWSSF

Q12300 SAVSQ-----GTHSK-----------------------NGPSSYRIPI---GLQYVWSSI

P42833 NILNF-----ICERCYKDP-------------------TQNIAWQLPL---FLGYIWAII

P49374 FYIGY-----GCHFI-----------------------DGAAAFRITW---GLQMVPGLI

O74713 FYLSF-----GLGHI-----------------------NGVASFRIAW---GLQIVPGLC

Q9BE72 YISNY-----AFANV-------------------------FHGWKYMF---GLVIPLGIL

Q5J316 YISNY-----AFANI-------------------------SHGWKYMF---GLVIPLGVL

Q32NG5 YICNY-----LFASV-------------------------NNGWKYMF---GLITPLAAL

Q6NWF1 FGLSW-----AFAGV-------------------------PDGWRFTF---GGALLPALL

Q0P4G6 YAMNY-----FLSGV-------------------------NESWKYMF---GLAIVPAAF

Q6GN01 YAMNY-----FLSAV-------------------------NDGWKYMF---GLAIIPAAF

O95528 YALNY-----ALAGT-------------------------PWGWRHMF---GWATAPAVL

Q8VHD6 YGLNY-----VLAGS-------------------------PWGWRHMF---GWAAAPALL

Q3UHK1 SVVDG-----AFSYL------------------------QKDGWRYML---GLAAIPAVI

Q96QE2 SVVDG-----AFSYL------------------------QKDGWRYML---GLAAVPAVI

Q9C757 YLINL-----AFTDV-------------------------TGTWRWML---GIAGIPALL

Q8VZR6 YLVNS-----AFTQV-------------------------PGTWRWML---GVSGVPAVI

P30606 YGCGA-----GLNHV-------------------------KNGWRILV---GLSLIPTVL

P30605 YGCGA-----GLNYV-------------------------NNGWRILV---GLSLIPTAV

Q10286 YGIDT-----AFEHV-------------------------HNGWRWMV---GLAMVPAAF

Q04162 YVIAS-----LMKEI-------------------------DNSWRYLF---ALSAIPAIL

Q01440 AGFTA-----IMVVF---T-------------------SKNIGWRVAI---GIGALPAVV

P11166 QVFG------LDSIM-----------------------GNKDLWPLLL---SIIFIPALL

P46896 QVFG------LDLIM-----------------------GNDSLWPLLL---GFIFVPALL

P47843 QIFG------LKVIL-----------------------GTEDLWPLLL---GFTILPAII

P14672 QVLG------LESLL-----------------------GTASLWPLLL---GLTVLPALL

Q90592 QVLG------LDFLL-----------------------GNDELWPLLL---GLSGVAALL

P11168 QIIG------LEFIL-----------------------GNYDLWHILL---GLSGVRAIL

Q5RB09 QLLG------LPELL-----------------------GKESTWPYLF---GVIVVPAVV

A4ZYQ5 QIFS------LQAIL-----------------------GNPAGWPVLL---ALTGVPALL

P22732 QIFG------LRNLL-----------------------ANVDGWPILL---GLTGVPAAL

P58353 QIFG------LRSLL-----------------------ANEEGWPILL---GLTGIPAVL

Q863Y9 QIVG------LRSLL-----------------------ATEEGWPILL---GLTAIPAAL

P43427 QLFG------LRSVL-----------------------ASEEGWPILL---GLTGVPAGL

Q9WV38 QLFG------LRSLL-----------------------ANEDGWPVLL---GLTGVPAGL

P15686 GLVNY-----AVRDW-----------------------EN--GWRLSL---GPAAAPGAI

Q39525 GLVNY-----GVRNW-----------------------DN--GWRLSL---GLAAVPGLI

Q94AZ2 NLVNY-----GTAKI-----------------------KGGWGWRLSL---GLAGIPALL

Q10710 NMVNY-----GTHKL-----------------------E-SWGWRLSL---GLAAAPALL

Q41144 NVLNY-----FFAKI-----------------------KGGWGWRLSL---GGAMVPALI

P23586 EVLNY-----FFAKI-----------------------KGGWGWRLSL---GGAVVPALI

O65413 NVLNF-----FFSKI-------------------------SWGWRLSL---GGAVVPALI

Q9SX48 NLINY-----GTSQM-----------------------AKN-GWRVSL---GLAAVPAVI

Q9LT15 NLINY-----GTSKM-----------------------AQH-GWRVSL---GLAAVPAVV

Q9FMX3 NIVNY-----VTPKL-----------------------QNGIGWRLSL---GLAGVPAVM

Q9SBA7 NIVNY-----FTSSI-----------------------H-PYGWRIAL---GGAGIPALI

Q9SFG0 NIVNY-----FTATV-----------------------H-PYGWRIAL---GGAGIPAVI

Q8L7R8 NVINY-----ETQNI-----------------------KH--GWRISL---ATAAIPASI

Q93Y91 NLINY-----GTDSH-----------------------RN--GWRISL---GLAAVPAAI

P0AE24 FLSDT-----AFSYSG--------------------------NWRAML---GVLALPAVL

P0AEP1 YLSDT-----AFSYTG--------------------------AWRWML---GVIIIPAIL

P96710 YFINL-----AVQRSGTYE------------------WGVHTGWRWML---AYGMVPSVI

P54723 YTFNA-----IIGSTMGES---------------------ANVWRYML---VIATLPAVV

O34718 FVFNA-----ILGTTMGDN---------------------SHVWRFML---VIASLPALF

P46333 YIVNY-----LFTPF--------------------------EAWRWMV---GLAAVPAVL

O52733 YITNY-----SFSGFY-------------------------TGWRWML---GFAAIPAAL

P0AGF4 YCVNY-----FIARSGDAS-----------------WL-NTDGWRYMF---ASECIPALL

P21906 YIFTW-----LLAHFGSID-----------------WV-NASGWCWSP---ASEGLIGIA

P15729 LLSNW-----FIALMAGGSA-------------QNPWLFGAAAWRWMF---WTELIPALL

Q6AWX0 YGIGS-----LTVNV-------------------------HSGWRYMY---ATSVPLAVI

Q93YP9 YLLGL-----FVP------------------------------WRILA---VLGVLPCTL

Q94AF9 YFFGT-----VIN------------------------------WRVMA---VIGAIPCIL

O04036 FIIGN-----FIP------------------------------WRLLT---VVGLVPCVF

Q94KE0 YVIGN-----FVH------------------------------WRNLA---LIGLIPCAL

Q9SCW7 FFTGN-----FFH------------------------------WRTLA---LLSAIPCGI

Q4F7G0 FFTGN-----FFH------------------------------WRTLA---LLSAIPSAF

Q8LBI9 FLIGS-----LIS------------------------------WKTLA---LTGLAPCIV

P93051 FIIGT-----LVT------------------------------WRVLA---LIGIIPCAA

Q0WQ63 FLIGA-----VVN------------------------------WRTLA---LTGVAPCVV

Q3ECP7 YLLGS-----FIG------------------------------WRILA---LIGMIPCVV

Q9LTP6 YLLGS-----VIS------------------------------WQKLA---LISTVPCVF

Q8GXK5 YALGT-----AVA------------------------------WRSLA---ILGSIPSLV

Q9JJZ1 YVAGW-----VLE------------------------------WRWLA---VLGCVPPTL

P58354 YLAGW-----VLE------------------------------WRWLA---VLGCVPPSF

Q9NY64 YLAGW-----VLE------------------------------WRWLA---VLGCVPPSL

Q9UGQ3 YALGL-----LLP------------------------------WRWLA---VAGEAPVLI

P43562 HYLCI-----ALSLW-----------------------DSHFAFRIAW---CIGIIPGLL

Q9FYG3 --LFI-----GIPVH-----------------------NITGWWRVCF---WLSTIPAAL

P36035 I-IFY-----RAFGYFR---------------------DD--GWKILF---WFSIFLPIL

P47186 AGIMK-----NSQKKYA---------------------DSELGYKLPF---ALQWILPVP

A6QLI1 MPLAG-----ILVQ--------------------------YTGWSSVF---YVYGSFGMI

Q5W8I7 MPLAG-----ILVQ--------------------------YSGWSSVF---YIYGSFGIV

A4FV52 MPLAG-----VLVQ--------------------------YSGWSSVF---YVYGSFGIF

P34644 LPLSA-----FLVS--------------------------YVSWAAPF---YLYGVCGVI

Q66GI9 LMLSP-----ILMS--------------------------QGGIYGPF---VIFGLSGFL

Q9FKV1 MWLLP-----ALVE--------------------------LRGPESVF---LAEALAGVI

Q46916 APIMG-----WLTH--------------------------EVGWSHVF---FFMGGLGIV

Q91Y77 PLLLG-----NLTS--------------------------TVGLCYTL---RILCIFMFV

Q8TF71 PLLLR-----VLID--------------------------SVGLFYTL---RVLCIFMFV

A1L1W9 PYMLS-----GLLK--------------------------SVGLYHTL---RVLAIFMFI

P36021 PFLIR-----MLGD--------------------------KIKLAQTF---QVLSTFMFV

O35308 SPLGQ-----LLGE--------------------------RFGWRGGF---LLFGGLLLH

O95907 SPLGQ-----QLLE--------------------------RFGWRGGF---LLLGGLLLH

Q90632 SPLGQ-----VLLE--------------------------KFGWRGGF---LIMGGLLLN

O35910 SPLGQ-----LLQD--------------------------HYGWRGGF---LILGGLLLN

O15427 SPLGQ-----LLQD--------------------------RYGWRGGF---LILGGLLLN

P57788 SPLGQ-----ILQH--------------------------EYGWRGGF---LILGGMLLN

O15375 PLLSR-----YLLE--------------------------NLGWRGTF---LVFGGIFLH

Q6ZSM3 APVVQ-----LLIE--------------------------QFSWRGAL---LILGGFVLN

Q8BGC3 APVVQ-----LLIE--------------------------QFSWRGAL---LILGGFVLN

Q503M4 APVVQ-----LLIE--------------------------HYSWRGAL---LILGGFVLN

O15403 APAIM-----ALKE--------------------------RIGWRYSL---LFVGLLQLN

Q8NCK7 APALQ-----LLLD--------------------------TFGWRGAL---LLLGAITLH

Q7RTY0 APFFQ-----WLLS--------------------------HYAWRGSL---LLVSALSLH

Q5R5M4 AALQR-----MLVE--------------------------FYGLDGCL---LIVGALALN

Q7TM99 AALQR-----MLIE--------------------------FYGLDGCL---LIVGALALN

Q5ZJU0 AALQR-----ELIE--------------------------LYGLDGCL---LIVGALSLN

O15374 APFTK-----FLID--------------------------LYDWTGAL---ILFGAIALN

Q08777 PIMLR-----SFFSMKSDT-------------------DPTYGFVWGI---RTLGFLDLA

Q08268 PIMLR-----KLYK--------------------------EVGFQWAI---RILSFICLT

P39709 GGIQS-----AVYSSLNGV-------------------NGLEGWRWNF---IIDAIVSVV

P25621 GFMQT-----SIFTHLNGR-------------------NGLAGWRWLF---IIDFCITLP

O13880 SLVSA-----GFQKM-DGA-------------------RGLYGYQWMF---LIWGVVAIA

P40445 SLLAS-----GIIEM-RGI-------------------HNLAGWQYLF---IIEGFLSLS

P15365 NAIAY-----GVYIH-QDS-------------------YAIKGWRTLF---VITGVITIF

Q07904 GLISF-----G-FLH-YHG-------------------TAFTSWQIMF---LVVGLVTVA

P53322 GLIAY-----G--CS-KIS-------------------GSLKDWQYIY---IVEGCISLG

P32071 PLIGA-----AVINRS----------------------HDADGWRWSF---WFMAILSGV

P38124 PLLGA-----AMV--------------------------DAKNWRFIF---WLLMWLSAA

Q06451 PLVNG-----FISVST------------------------GR-MDLIF---WVNMAFAGV

P53283 PLVNG-----FISVST------------------------GR-MDLIF---WVNMAFAGV

Q9C0R8 PIFGG-----LLCDHA------------------------PT-WRWIY---WTFLIVAGV

Q9C0Q6 PIFGG-----LLCDYA------------------------PT-WRWVY---WTFLIVAGF

Q9HF77 PILGA-----FTVKNS------------------------ALGWRWTS---YFCGIIGSL

Q5A0E9 PIFGG-----FTVKNS------------------------SLGWRWTA---YFCGIVACL

Q07824 PVMGS-----YIAQRT------------------------T--WRWLE---YVVGCFASA

O59698 PIVGG-----FLTK-S------------------------YLGWRWTE---YITSFMGFL

O74829 PIVGG-----FIGK-S------------------------YLGWRWTS---YITAIMGFT

P38776 PIIGG-----ALYHQS---------------------------YKWTF---ITLLITSGC

Q9HDX4 PIASG-----FIAGSS-------------------------ISWRWEF---WILLMLSGV

P38227 SPIFG-----SLLV--NR----------------------WP-WRSTQ---WFMVILSGC

P38125 APIVA-----GLIL--MK----------------------GNYWRWLF---GFTSIMTGI

P40474 GALIG-----AGLS--SR-----------------------WGWRAIF---WFLAIGSGI

O94607 GPVLA-----QAFH--PEK---------------------NYGWRLGY---GIWAFILPT

Q9HE13 GPLIG-----GAI----SQ---------------------NTTWRWIF---FIN---LPT

Q04301 GAPLG-----GVF----IE---------------------TIGWRALF---GIQ---VPV

P13090 GCLFA-----GLIG--TED---------------------PKQWPWAF---YAY---SIA

Q08902 GAVFS-----SMLG----Q---------------------LAWWPWAY---WIM---GIA

P39886 GPIIG-----GLL----VQ---------------------HVGWEAVF---FIN---VPV

P76269 GPTIA-----AAI----LS---------------------IASWKWLF---LIN---VPL

Q8Y9K8 GPTIS-----GLI----LE---------------------NLTWNWIF---WIS---LPF

P28873 SFVMLCFT--------------LPETFGKTLL----------------YRKAKRLRAITG

A4WFG6 IFVLTFGC------------------EFPVLG----------------------KKA---

A1JSB0 IFVLTLCS------------------EFPVLG----------------------HKA---

A8GKP6 IFVLTLFS------------------EFPVLG----------------------NKG---

P57601 IFLLTINS------------------SFEKFK----------------------TNT---

Q8K942 IFILTVKS------------------HFPVSE----------------------EKT---

Q89A60 IFLITINT------------------KFPIIY----------------------TEI---

P76198 ITLMLLKS------------------KFPSQL----------------------VDA---

O05390 LALFFNKD------------------KFTPIQ----------------------KQD---

O34864 FALFINKD------------------RFEKKK----------------------RKR---

O52717 IALVSMRH------------------TETPRH----------------------MQNLTT

O52718 IALVSLRH------------------IQTPQH----------------------MQNLTT

A9MJT5 VCLLSLFI-------------------LRETR----------------------PARLA-

A6TG19 VAVLSVFI-------------------LRETR----------------------PT--A-

A0L190 VFGLCLFV-------------------LRETY----------------------SK--A-

P0AEY8 SFFGLQRA-------------------MPET-----------------------ATRIGE

Q7CP73 ALCGLLLA-------------------MPET-----------------------VQRGAV

P39386 SFVGLLLA-------------------MPET-----------------------VKRGAV

Q68WD6 LLALYYKI-------------------LPETN----------------------YYIDFS

Q4UMJ9 LLALYYKV-------------------LPETN----------------------SYIDFS

Q1RI77 LLTLYCKI-------------------LPETN----------------------PYINFS

P32482 ASAAAWRF-------------------WPETR----------------------VQRVA-

P37597 LILPIFWL-------------------KPTTK----------------------ARNNSQ

P45123 AAALVFFI-------------------IPETH----------------------KKENRI

P28246 ASAMIFFL-------------------IKETL----------------------PPERRQ

P31442 VTFSMARW-------------------MPETR----------------------P----V

P37482 ALLIWI----------------------PQLR----------------------HRDTAN

P76242 ALFLWL----------------------PQWR----------------------SQQHAN

P17583 ALFAWW----------------------WQ-S----------------------AREVAS

P0C105 LAIIFAIL------------------KPPDVQ----------------------EDEPA-

O25788 LALIMYLL------------------KLPDVE----------------------KEMPK-

P11551 VALLIMLT------------------KFPALQ----------------------SDNHS-

P44776 VFIIIGLK------------------KMPAVK----------------------IE----

A1A9E1 GILPLLFT------------------HVLNQQ----------------------AENHDS

A9MHY5 GILPLLFT------------------RIVNQQ----------------------TEARYS

A4W8S1 AILPLLFT------------------RIVNQG----------------------SEHHEA

A1JMG4 AMLPLLFA------------------HFSHQD----------------------SGDVPH

Q0TK80 TLLPL-LS------------------LPALPP----------------------PPQPRE

O06473 ALIAISFM------------------LPKLPK----------------------PAK-RV

Q2FI61 IAWILYY--------------------FVDVK----------------------LTNYN-

Q5HQE8 IAWVLYY--------------------FVNIN----------------------LTNYN-

Q49WE5 IAWILYY--------------------FVKVR----------------------LTNYN-

Q4L523 IAWVLYY--------------------FVDIK----------------------LTNYN-

P33026 MVWLFLP--------------------SMR-K----------------------ELPLA-

P31436 IVWLFLP--------------------SIQ-R----------------------NIPVV-

Q9S3K0 LVWLLLP--------------------SMP-K----------------------TRVKS-

P31675 LIAFMLP--------------------SVA-R----------------------VELPS-

P31126 FIQIWVK--------------------RSE-K----------------------IIATE-

Q58955 ISYMKLE-------------------DIVFNK----------------------NKEKI-

P02920 LFFAKTD-----------------APSSATVA----------------------NAVGA-

Q4UK37 VILCIKE---------------PKPKEKRHTT----------------------TNLIG-

Q1RKF6 IVLCIKE---------------PKPKQKSHRT----------------------KDLIN-

Q4ULW4 FIITVNE------------------KELVREK----------------------VNITS-

Q1RI01 FIITVKE------------------KELVREK----------------------IKFTS-

Q68WQ5 LLILVSRYTNSVDVIEENTSYFYVARCYTMEE----------------------MHLKNE

Q4UL88 LLIVAARYTNSFGLVEERICHSPSFLCHSRGS----------------------GNPNNE

Q92HQ3 LLIVGIKYCD----LNENI-HIQIIKNDIKNN----------------------QNKKN-

Q1RIL0 LLIVGVKYCR----FDQN--------NDIEQT----------------------TNNNDD

Q4UMU2 LIIIYPL--------------------KFKDK----------------------TIIND-

Q68W71 LIIFYPL--------------------KFKDK----------------------MIAND-

Q1RHK8 LIIFYPL--------------------KFKEK----------------------IAVND-

Q1LTM2 AAILNAWL-------------------LPAYR----------------------ISNAQ-

Q2NTK5 AAIFNAWL-------------------LPGYR----------------------ISTVR-

P55705 CAAVAWTS-------------------LPVPL----------------------GGAGL-

Q7Z3Q1 LIYILFFLGDPVK---------ECSSQNVTMS----------------------CS----

Q05B81 TLYAAFCFGETVK---------E---RTPTRL----------------------FT----

P76470 AGVFTFFWLDDTP---------EQARFLSKQE----------------------KTLLIN

P32135 VGIIIFFVLNDKE---------EAPSVEVKKE----------------------------

P38358 IILLLF---------------------ILHER----------------------KTSARA

A4WAE6 TLLCLIKL--------------LP---KLPS------------------------EHSG-

A6T8Y8 TLACLVKL--------------LP---TLPS------------------------EHSG-

Q6CZ44 IMVGLMKL--------------LP---VLPS------------------------SNSG-

Q888L8 VVFLLARA--------------LP---LLPS------------------------QNSG-

Q1IB51 TMLCLMKS--------------LP---LLPS------------------------QNSG-

A6UZY0 TLLYLVRS--------------LP---PLPS------------------------QNSG-

Q4QP52 IMFLIIRL--------------LP---NLPS------------------------KNAG-

Q9CM87 ILCLFYQL--------------LP---HLPS------------------------KNAG-

O25797 IALLMWKL--------------LP---HLPS------------------------RNAG-

Q17YP7 VALLMYRL--------------LP---SLPS------------------------RNAG-

P77389 SMVSLFFS--------------LP----KGG------------------------AGARP

O31577 GFLGLMAA--------------VP---NRKP------------------------KVIPM

O34367 AFITNGIL--------------VP---SKLR------------------------KGTKT

A1AHK2 CIFWIIKS--------------LP---SLPG------------------------EPSH-

A9MWE8 CVIWVVKS--------------LP---SLPG------------------------EPSH-

P23910 VMASVYFW--------------VP---DIRD------------------------EAKG-

P31141 AAFGVLKA--------------IPAGRATAA------------------------ATGGP

P43531 SALMFWKI--------------LPESRHFRP------------------------TSLRP

Q8K902 SSCLFLYF--------------LPSSKNFYP------------------------ISIDF

P57648 SSCFFLYF--------------LPPSKNFLS------------------------VSINF

Q89A23 SAVLFVYL--------------LPKSKNFCS------------------------SPLDL

P37498 ASVIFFIN--------------LPPSRHFTP------------------------RKLKL

P0A0J4 AFIMSIVL--------------IHDPKKSTT----------------------SGFQKLE

Q07282 AFLVSLFI--------------LHETHNANQVS--------------------DELKNET

P02982 NFLTGCFL--------------LPESHKGER----------------------RPLRREA

P02981 NLLLGCFL--------------MQESHKGER----------------------RPMPLRA

P70187 CFILVAVPES------------LPEKMRPASWG--------------------APISWEQ

Q5SR56 CFILVAVPES------------LPEKMRPVSWG--------------------AQISWKQ

P77726 GIALTIWV--------------VPNS--STH----------------------VL-NRES

Q89AA9 CLFFGMFY--------------IPASLLNKN----------------------VVCNFRS

Q8K999 SILIVFFL--------------IPSS--QNE----------------------ILKNYKK

P57538 CMIIVCFF--------------VPFS--KKN----------------------ILKQNKT

Q5HIA2 FGLYLRRK--------------LEESPVF-EN----------------------------

Q4L3Q4 FGLYLRRK--------------LEESPIY-EN----------------------------

Q5HRH0 FGLYLRRK--------------LEESPIY-EN-------------------------EQR

P0C0L7 IGLYLRHA--------------LEETPAF-QQH--------------------VDKLEQG

Q47421 IGLYLA-T--------------LEETPAF-RQH--------------------VEKLEQN

P0A2G3 LIFVLRRS--------------LQETEAF-LQ----------------------------

P16482 FIFILRRK--------------LEETQEF-TA----------------------------

P0AEX3 VALWLRRQ--------------LDETS---------------------------------

P76350 GALWVRNG--------------MEESAEF-EQ---------------------------Q

P41036 FALWLRKN--------------IPEAEDWKEKH--------------------AGKAPVR

Q9SYQ1 TFYWRM-L--------------MPETARYTALV--------------------ENNIVQA

Q9S735 TFYWRM-L--------------MPETARYTALV--------------------ENNVVQA

P76230 ATALAWRY--------------FPESPRW--LE--------------------SRGRYQE

P38055 AWFLSGKY--------------FIESPRW--LA--------------------GKGQIAG

Q46909 ITLLRW-G--------------TPESPRW--LL--------------------RQGRFAE

P31679 ILIGRF-E--------------LPESPRW--LL--------------------RKGRVKE

O24723 ILPFFVAL--------------VPEPAIISVRR--------------------GIPEARI

O30513 LLLPLMKV--------------LPESIDYL-VR--------------------KKKDETV

Q43975 LMLLVIFF--------------LPESYRFLIVK--------------------GKNTKKV

Q51955 LALVLMVW--------------LPESARFLVVR--------------------NRGTDKI

Q9I6Q3 LAVVLLFR--------------LPESARYLVVR--------------------NRGSERV

P77589 LVPLLMRW--------------LPESAVFAGEK--------------------QS-----

P94131 MAVLMHFF--------------VPEPAAWQQSR--------------------LAPSKQT

P71369 VAWFLRSH--------------LHEPEIFTQKQ--------------------TALSTQS

O34691 YALYLRTS--------------LPDSPKYESLS--------------------AKKRSMW

P0AGC0 VGFIGLRY--------------GSDSPESYGLGKA-------------------------

P27669 VGMVLCWR--------------LRDRPQAIGLPPV-------------------------

P96335 FAIPVYFV--------------MRDTPQSCGLPSI-------------------------

P08194 VALFAFAM--------------MRDTPQSCGLPPI-------------------------

P37948 ISFLIVLL--------------VRDTPQSCGLPPI-------------------------

P12681 FALIVLVL--------------GKGSPRKEGLPSL-------------------------

Q5M7K3 FGIFCFFF--------------LVEYPEDVGCTPP---------------QQPEDTKENL

Q9WU81 MGVITFLF--------------LIEYPEDVDCTPP---------------RHHDDP-EKE

Q58CV5 MGIITFFF--------------LIEYPEDVDCSPP---------------QHHGNP-EES

Q7SY29 TGVICFLF--------------LVERPEDVNCTAP---------------QHHERV-E--

P57057 MGIVCFLF--------------LIEHPNDVRCSSTLVTHSKGYENGTNRLRLQKQILKSE

Q17QZ3 GGIIIFFG--------------LLVSPEEIGIPGI-------------------ETED--

Q3TIT8 GGIIIFFG--------------LLVSPEEIGLPSI-------------------GAEE--

Q640L2 GGIIIFFG--------------LVTSPKELGLPDT-------------------GEGEMD

Q5F3N0 GGVIVFCG--------------LLTSPKEVGLPEL-------------------GADE--

Q09037 AMFLVGMF--------------LREST---------------------------------

Q06222 AMFLVGMF--------------LREST---------------------------------

P13865 LTVVLGIV--------------TRESR---------------------------------

P46499 PILPVLLW--------------LPESPKWYAT------------------------KNRF

P54219 DGALQLCI--------------LQPS----------------------------------

Q6NT16 MVPLNMYI--------------L-PN----------------------------------

Q8R0G7 AVLLLFLV--------------VQEPPR--------------------------------

Q5XGK0 AVLLLIFV--------------AEEPPR--------------------------------

Q7ZU13 AVFLLMLV--------------VQEPKR--------------------------------

A2CER7 GLLLLVFL--------------IPNPPR--------------------------------

Q9D232 ALALLILL--------------VPDVPR--------------------------------

Q6ZMD2 ALILLILL--------------VPDPPR--------------------------------

Q6GPQ3 GNIILIFA--------------IFREHR--------------------------------

A5IVG9 ALIMFIFG----------------------------------------------------

Q5HLK7 AILMFFLG----------------------------------------------------

P46907 ALLHVLFG----------------------------------------------------

P10903 TIA-AWFG----------------------------------------------------

P37593 TLA-AWFG----------------------------------------------------

P37758 TIA-AWSG----------------------------------------------------

Q9P3K8 GCLGTFGLKIVDEEDLIDEAVEELERSGYLDGSTFLQGSWTADRPGYGAIEQSPLDMESA

Q6FWD4 GVMIWIAT----------SKVAQIQHEADIME------------------EQDRMAEPSD

Q6CPY8 FLLTWIST----------STISIIK----LRG------------------SQSPSASHAS

P22152 GMLFTCDD---------TPTGKWSERHIWMKEDTQTASKGNIVDLSSGAQSSRPSGPPSI

Q02563 AIGAL-TT--------------QPESPRFFLEN---------GKHDEAWM---VLKQVHD

Q496J9 SVVAL-TF--------------MPESPRFLLEV---------GKHDEAWM---ILKLIHD

Q63564 SMVAL-KF--------------MPESPRFLLEM---------GKHDEAWM---ILKQVHD

Q1JP63 FAVLC-FW--------------LPESARYDVLS---------GNQEKAIA---TLKRIAT

Q2XWK0 FAILC-FW--------------LPESARYEVLS---------GNQEKALA---TLKRIAT

Q1LVS8 LIGLF-MF--------------IPESARFQVSA---------GNIQGAMS---TLKRIAK

O08966 FLLYY-WF--------------VPESPRWLLSQ---------KRTTQAVR---IMEQIAQ

O15245 FLLYY-WC--------------VPESPRWLLSQ---------KRNTEAIK---IMDHIAQ

O77504 CLFYY-WC--------------VPESPRWLLSQ---------KRNTDAVK---IMDNIAQ

A7MBE0 LLFRF-WF--------------VPESPRWLLSQ---------KRNTEAIK---IMDHIAQ

Q9R0W2 FLLYF-WC--------------IPESPRWLISQ---------NKIVKAMK---IIKHIAK

O02713 FLFYY-WC--------------VPESPRWLISQ---------NKNAKAMS---IIKHIAK

Q8MJI6 FLLYY-WC--------------IPESPRWLISQ---------NKNAKAMR---IMEHIAK

O88446 FLLYY-WV--------------VPESPRWLITR---------KQGEKALQ---ILRRVAK

O75751 FLLYY-WV--------------VPESPRWLITR---------KKGDKALQ---ILRRIAK

Q9U539 YIIYY-FF--------------LPESPRWSVSV---------GKWADAKK---QLKKIAK

O76082 CVALW-WF--------------IPESPRWLISQ---------GRFEEAEV---IIRKAAK

Q497L8 FILCC-WM--------------LPETPFWLLSE---------GRYKEAQG---TVDTMAV

Q86VW1 FILCC-WV--------------LPETPFWLLSE---------GRYEEAQK---IVDIMAK

Q17QN9 FVLCC-WM--------------LPETPFWLISG---------GKYEEAQK---VIDTMAK

Q95R48 FMFYY-WI--------------IPESARWLLLK---------GRKDCAIA---NMQKAAR

Q9Y267 FISYI-WI--------------LPESPRWLMMK---------GKVKEAKQ---VLCYAAS

Q6A4L0 LFFYF-WV--------------LPESPRWLLSQ---------GRTEEAKQ---LVQKAAL

Q9Y226 LFFYF-WA--------------LPESARWLLTR---------GRMDEAIQ---LIQKAAS

Q8IVM8 IFLTS-SW--------------LLESARWLIIN---------NKPEEGLK---ELRKAAH

Q66J52 YFLYS-WW--------------IPESGRWLVLS---------GKPEVACK---ALKKVAQ

Q91WU2 GIISI-WW--------------VPESARWLLTQ---------GRVEEAKK---YLSICAK

Q8IZD6 VFLLS-LF--------------IPESPRWLYSQ---------GRLSEAEE---ALYLIAK

Q6DFR1 VLALS-LC--------------IPESPRWLHAQ---------GRLREAQE---SLLSLGR

Q6NUB3 VLALS-LC--------------IPESPRWLYAQ---------GRLHEAQE---SLVSLGR

Q28ES4 FFLLS-FL--------------LPESPRWLYSH---------GYTTEAEG---VLQSMAV

A6NKX4 FWGFP-AL--------------FPESPCWLLAT---------GQVARARK---ILWRFAE

P47185 IIVGM-LL--------------VPESPRYLIE---------CERHEEACV---SIAKI-D

P13181 MIGAL-TL--------------VPESPRYLCE---------VNKVEDAKR---SIAKS-N

P23585 MIAGM-LM--------------VPESPRFLVE---------KGRYEDAKR---SLAKS-N

Q92339 LMVGV-LF--------------LPESPRYLIY---------KGRDEEALR---IMCNM-A

O74969 TMIGI-LF--------------LPESPRYLIQ---------VGKDEEAVR---VLSES-A

P10870 LAIGM-FF--------------LPESPRYYVL---------KDKLDEAAK---SLSFL-R

Q12300 LAVGM-IF--------------LPESPRYYVL---------KDELNKAAK---SLSFL-R

P42833 IGMSL-VY--------------VPESPQYLAKI--------KNDVPSAKY---SFARM-N

P49374 LMVGV-FF--------------IPESPRWLAN---------HDRWEETSL---IVANIVA

O74713 LFLGC-FF--------------IPESPRWLAK---------QGQWEAAEE---IVAKIQA

Q9BE72 QAIAM-YF--------------LPPSPRFLVM---------KGQEGAASK---VLGRLRA

Q5J316 QAIAM-YF--------------LPPSPRFLVM---------KGHEEAASK---VLGKLRA

Q32NG5 QAVAM-FF--------------LPRSPRFLIM---------KGYDDAAGK---VLQKLRA

Q6NWF1 QAGVM-PL--------------LPDSPRFLLA---------QQREKEAHA---TLLRLRA

Q0P4G6 QFISI-LF--------------LPSKPHKL-----------NFWEQDTDD---GFIELE-

Q6GN01 QFIVI-LF--------------LPSKPHTL-----------NFWEQDSDN---GFIELE-

O95528 QSLSL-LF--------------LPAGTD----------------ETATHK---DLIPLQG

Q8VHD6 QSLSL-FL--------------LPAGAE----------------GTAAPK---DLIPLQG

Q3UHK1 QFLGF-LF--------------LPESPRWLIQ---------KGQTQKARR---ILSQMRG

Q96QE2 QFFGF-LF--------------LPESPRWLIQ---------KGQTQKARR---ILSQMRG

Q9C757 QFVLM-FT--------------LPESPRWLYR---------KGREEEAKA---ILRRIYS

Q8VZR6 QFILM-LF--------------MPESPRWLFM---------KNRKAEAIQ---VLARTYD

P30606 QFSFF-CF--------------LPDTPRYYVM---------KGDLKRAKM---VLKRSYV

P30605 QFTCL-CF--------------LPDTPRYYVM---------KGDLARATE---VLKRSYT

Q10286 QLFIL-IW--------------LPESPRLLVK---------KERSQEAYN---TLARIYP

Q04162 FLSIL-DF--------------IPESPRWSIS---------KGDILYTRD---SLRMLYP

Q01440 QAFCLLFF--------------LPESPRWLLS---------KGHADRAKA---VADKFEV

P11166 QCIVL-PF--------------CPESPRFLLIN--------RNEENRAKS---VLKKLRG

P46896 QCIIL-PF--------------APESPRFLLIN--------RNEENKAKS---VLKKLRG

P47843 QCAAL-PF--------------CPESPRFLLIN--------RKEEEKAKE---ILQRLWG

P14672 QLVLL-PF--------------CPESPRYLYII--------QNLEGPARK---SLKRLTG

Q90592 QFFLL-LL--------------CPESPRYLYIK--------LGKVEEAKK---SLKRLRG

P11168 QSLLL-FF--------------CPESPRYLYIK--------LDEEVKAKQ---SLKRLRG

Q5RB09 QLLSL-PF--------------LPDSPRYLLLE--------KRNEARAVK---AFQTFLG

A4ZYQ5 QLLTL-PF--------------FPESPRYSLIQ--------KGDEATARQ---ALRRLRG

P22732 QLLLL-PF--------------FPESPRYLLIQ--------KKDEAAAKK---ALQTLRG

P58353 QLLFL-PF--------------FPESPRYLLIQ--------KKDEAAAKS---ALRRLRG

Q863Y9 QLLLL-PF--------------FPESPRYLLIQ--------KKDAAAAKN---ALKRLRG

P43427 QLLLL-PF--------------FPESPRYLLIQ--------KKNESAAEK---ALQTLRG

Q9WV38 QLLLL-PF--------------FPESPRYLLIQ--------KKDEAAAER---ALQTIRG

P15686 LFLGS-LV--------------LPESPNFL-VE--------KGKTEKGRE---VLQKLCG

Q39525 LLLGA-IV--------------LPESPNFL-VE--------KGRTDQGRR---ILEKLRG

Q94AZ2 LTVGA-LL--------------VTETPNSL-VE--------RGRLDEGKA---VLRRIRG

Q10710 MTIGG-LL--------------LPETPNSL-IE--------QGLHEKGRN---VLEKIRG

Q41144 ITVGS-LV--------------LPDTPNSM-IE--------RGQHEEARA---HLKRVRG

P23586 ITIGS-LV--------------LPDTPNSM-IE--------RGQHEEAKT---KLRRIRG

O65413 ITVGS-LI--------------LPDTPNSM-IE--------RGQFRLAEA---KLRKIRG

Q9SX48 MVIGS-FV--------------LPDTPNSM-LE--------RGKYEQARE---MLQKIRG

Q9LT15 MVIGS-FI--------------LPDTPNSM-LE--------RGKNEEAKQ---MLKKIRG

Q9FMX3 MLVGC-FF--------------LPDTPNSI-LE--------RGNKEKAKE---MLQKIRG

Q9SBA7 LLFGS-LL--------------ICETPTSL-IE--------RNKTKEGKE---TLKKIRG

Q9SFG0 LLFGS-LL--------------IIETPTSL-IE--------RNKNEEGKE---ALRKIRG

Q8L7R8 LTLGS-LF--------------LPETPNSI-IQ----------TTGDVHKTELMLRRVRG

Q93Y91 MTVGC-LF--------------ISDTPSSL-LA--------RGKHDEAHTSLLKLRGVEN

P0AE24 LIILV-VF--------------LPNSPRWLAEK---------GRHIEAEE---VLRMLRD

P0AEP1 LLIGV-FF--------------LPDSPRWFAAK---------RRFVDAER---VLLRLRD

P96710 FFLVL-LV--------------VPESPRWLAKA---------GKTNEALK---ILTRING

P54723 LWFGM-LI--------------VPESPRWLAAK---------GRMGDALR---VLRQIRE

O34718 LFFGM-IR--------------MPESPRWLVSK---------GRKEDALR---VLKKIRD

P46333 LLIGI-AF--------------MPESPRWLVKR---------GSEEEARR---IMNI--T

O52733 LFLGG-LI--------------LPESPRFLVKS---------GHLDEARH---VLDTMNK

P0AGF4 FLMLL-YT--------------VPESPRWLMSR---------GKQEQAEG---ILRKIMG

P21906 FLLLL-LT--------------APDTPHWLVMK---------GRHSEASK---ILARLEP

P15729 YGVCA-FL--------------IPESPRYLVAQ---------GQGEKAAA---ILWKVEG

Q6AWX0 MGIGM-WW--------------LPASPRWLLLRVIQGKGNVENQREAAIK---SLCCLRG

Q93YP9 LIPGL-FF--------------IPESPRWLAKM---------GLTDDFET---SLQVLRG

Q94AF9 QTIGI-FF--------------IPESPRWLAKI---------RLSKEVES---SLHRLRG

O04036 HVFCL-FF--------------IPESPRWLAKL---------GRDKECRS---SLQRLRG

Q94KE0 QVVTL-FF--------------IPESPRLLGKW---------GHEKECRA---SLQSLRG

Q9SCW7 QMICL-FF--------------IPESPRWLAMY---------GRERELEV---TLKRLRG

Q4F7G0 QVICL-FF--------------IPESPRWLAMY---------GQDQELEV---SLKKLRG

Q8LBI9 LLFGL-CF--------------IPESPRWLAKA---------GHEKEFRV---ALQKLRG

P93051 SFLGL-FF--------------IPESPRWLAKV---------GRDTEFEA---ALRKLRG

Q0WQ63 LFFGT-WF--------------IPESPRWLEMV---------GRHSDFEI---ALQKLRG

Q3ECP7 QMMGL-FV--------------IPESPRWLAKV---------GKWEEFEI---ALQRLRG

Q9LTP6 EFVGL-FF--------------IPESPRWLSRN---------GRVKESEV---SLQRLRG

Q8GXK5 VLPLL-FF--------------IPESPRWLAKV---------GREKEVEG---VLLSLRG

Q9JJZ1 MLLLM-CY--------------MPETPRFLLTQ---------HQYQEAMA---ALRFLWG

P58354 MLLLM-CF--------------MPETPRFLLSQ---------HKHQEAMA---AMQFLWG

Q9NY64 MLLLM-CF--------------MPETPRFLLTQ---------HRRQEAMA---ALRFLWG

Q9UGQ3 MILLL-SF--------------MPNSPRFLLSR---------GRDEEALR---ALAWLRG

P43562 FWMAS-YA--------------LPESYHWLVLH---------GKMSEAQEIQHNLAKKFN

Q9FYG3 LALGM-FL--------------CAESPQWLFKQ---------GKIAEAEA---EFERLLG

P36035 LIFWR-LL--------------WPETKYFTKVL--------KAR----KLILSDAVKANG

P47186 LALGI-FF--------------APESPWWL-VK--------KGRFDEARRSLRRTLSGKG

A6QLI1 WYMFWLLV--------------SYESP-AKHPTITDEE---RRYIEE--SIGESANL---

Q5W8I7 WYMFWILV--------------SYESP-ADHPTITDEE---RTYIEE--SIGESAKL---

A4FV52 WYLFWLLV--------------SYESP-ALHPSISEEE---RKYIED--AIGESAKL---

P34644 WAILWFCV--------------TFEKP-AFHPTISQEE---KIFIED--AIGHVSNT---

Q66GI9 WLLVWLSA--------------TSSAP-DRHPQITKSE---LEYIKQKKQISTMENK---

Q9FKV1 WSLLWIRY--------------ATDPPRSEHPKAAAAG---FGG-----ALLPTNVN---

Q46916 ISFIWLKV--------------IHEP--NQHPGVNKKE---LEYIAAGGALINMDQQ---

Q91Y77 LFLAGFTY--------------RPLVPSSKEKESEDSR---SS-----------------

Q8TF71 LFLAGFTY--------------RPLATSTKDKESGGSG---SS-----------------

A1L1W9 LMLAGLTY--------------KPLLP--KPVSSSKPG---S------------------

P36021 LMLLSLTY--------------RPLLPSSQDTPS-KRG---VR-----------------

O35308 CCACGAVM--------------RPPPGP-P-PRRDPS----PH---------GGPA----

O95907 CCACGAVM--------------RPPPGPGPRPRRDSAG---DR---------AGDAPGEA

Q90632 CCTCGAVM--------------RPLDA-GMKRKTEKAQ---DKYEAKEMLPIGGKSEEGI

O35910 CCVCAALM--------------RPLVAPQASGGAEPHG---PQ-----------------

O15427 CCVCAALM--------------RPLVVTAQPGS----G---PP-----------------

P57788 CCVCGALM--------------RPLEPPKKSEATKEPA---EK-----------------

O15375 CCICGAII--------------RPVATSVAPETKECPP---PPPETPALGCLAACG----

Q6ZSM3 LCVCGALM--------------RPITLKEDHT--TPEQ---NH--------VCRTQKEDI

Q8BGC3 LCVCGALM--------------RPITLKEDRS--VPEK---NH--------NRESQREDC

Q503M4 LCVCGALL--------------RPIILKEEEACPLPVD---SE--------CGYSVKPPT

O15403 IVIFGALL--------------RPIFIRGPASPKIVIQ---ENRKEAQYMLENEKTRTSI

Q8NCK7 LTPCGALL--------------LPLVLPGDPPAPPRSP---LA-----------------

Q7RTY0 LVACGALL--------------RPPSLAEDPAV--GGP---RA-----------------

Q5R5M4 ILACGSLM--------------RPLQSSDCPLPKKIAP---EDLPDKYSIYNEKGKNLEE

Q7TM99 ILACGSLM--------------RPLQTSDCPFPEKTAP---ENVPDRYSMYNEKEKNPEE

Q5ZJU0 ILACGSLM--------------RPLESSDSPSPEKACT---DKVPDQYFVYHEKEKTVEE

O15374 LVPSSMLL--------------RPIHIK---SENNSGI---KDKGSSLSAHGPEAHATET

Q08777 LLTLSIIL--------------VKERLPHVIE--NSKD---GES----------------

Q08268 CLICASVL--------------ARERTKPVVQPFKSKA---EVA----------------

P39709 VGLIGFYS--------------LPGDP--------YNC---YSIFLTDDEIRLARKRL-K

P25621 IAIYGFIF--------------FPGLPDQTSAVSKFSM---TRYIFNEQELHYARRRLPA

O13880 QALSIPWW--------------LPAVASKEHR-KSLSS---FIPL-PKWMKTLSPQRI-G

P40445 VGVASFYL--------------MRRGPTQTGE-SAFHK---GKSLFTEYEEKIMVNRI-L

P15365 IGILIFLW--------------IPDDPSKAR-------------FLSKREKLMVVQRI-R

Q07904 FGVLTFLY--------------LPDNVTNAW-------------FLNKEEKIQVVEHI-R

P53322 FVPFYAFG--------------LSKNLEDSW-------------FFNKEEKEYISERY-K

P32071 CFIVLSFS--------------LPETYGKTLL----------------RRKAERLRKLTG

P38124 TFILLAFF--------------FPETQHHNIL----------------YRRALKLRKETG

Q06451 MWIISSAI---------------PETYAPVIL----------------KRKAARLRKETG

P53283 MWIISSAI---------------PETYAPVIL----------------KRKAARLRKETG

Q9C0R8 FYAIFIAI--------------VPETHHGILL----------------KKRAKKLRKETG

Q9C0Q6 FYVVFIVV--------------VPETHHGILL----------------KKRAKKLRKDTG

Q9HF77 ALFMNTFL--------------LQETHHPLIL----------------TRRAEELRRRTG

Q5A0E9 GLVLNVFL--------------LDETHHPIIL----------------VKRAEELRRRTG

Q07824 VFVAIVLF--------------FEETHHPTIL----------------VNKAKQMRKQSN

O59698 SIILIYLF--------------CEETYLKTIT----------------ENKVQEYREITG

O74829 AFTSMIIF--------------HRETYTRTIT----------------EIRASKVRVLTG

P38776 CLVMIIFT--------------IPETYKPMLL----------------IRKAKRLRKEKN

Q9HDX4 SLLAGVVF--------------LKETYAPVLK----------------RKQAKKLLEKQE

P38227 NVILLTVL--------------LPETLRKQDSKGAIAQ---ILAERRIQVDNNERGEIQE

P38125 ALILVTAL--------------LPETLR------CI-------------VGNG-------

P40474 CFLASFLI--------------LPETKR--------------------------------

O94607 VSLPLLAS--------------L-FLNQRKAKAAGLYR---EHHNLINHSTPEKLRLFYL

Q9HE13 GGLSLALL--------------IFFLN-------------------LVPKPTVSFCVFLR

Q04301 IMLCSVLA--------------IKNINIK-----------------LFHVPPMKERYTLK

P13090 AFINFVLS--------------IYAIP-------------------ST-IPT-NIHHF-S

Q08902 CFVLAVAG--------------YFVIP-------------------HTPMPSRDASSF-K

P39886 GLAALVAG--------------LVILT---------------------------DARAER

P76269 GIIALLLA--------------MRFLP---------------------------PNGSRA

Q8Y9K8 LIIALLFG--------------MKFM----------------------------QNVSVV

P28873 NDR--------ITSEGEIENS--------------------------------------K

A4WFG6 EQSTQ------------------------------------------------------P

A1JSB0 TDQSK------------------------------------------------------P

A8GKP6 ADAGQ------------------------------------------------------P

P57601 KNSKE-------------------------------------------------------

Q8K942 KNNNE-------------------------------------------------------

Q89A60 SKKR--------------------------------------------------------

P76198 SVTNEL-----------------------------------------------------P

O05390 QPKWKE-----------------------------------------------------L

O34864 SETAEE-----------------------------------------------------L

O52717 REKFAE-----------------------------------------------------L

O52718 REKFSE-----------------------------------------------------L

A9MJT5 PRDLSR-----------------------------------------------------S

A6TG19 PPQAAL-----------------------------------------------------P

A0L190 SFHSQT-----------------------------------------------------L

P0AEY8 KLSLKE-----------------------------------------------------L

Q7CP73 PFSAVS-----------------------------------------------------V

P39386 PFSAKS-----------------------------------------------------V

Q68WD6 Q--SSK-----------------------------------------------------Y

Q4UMJ9 Q--SSK-----------------------------------------------------Y

Q1RI77 Q--TSK-----------------------------------------------------Y

P32482 ----GL-----------------------------------------------------Q

P37597 D---GL-----------------------------------------------------T

P45123 PLRLNI-----------------------------------------------------I

P28246 PFHIRT-----------------------------------------------------T

P31442 DAPRTR-----------------------------------------------------L

P37482 -QTMK------------------------------------------------------L

P76242 LSTSRA-----------------------------------------------------L

P17583 SHKTTT-----------------------------------------------------T

P0C105 LSDKKE-----------------------------------------------------G

O25788 ETTQK-------------------------------------------------------

P11551 DAKQGS-----------------------------------------------------F

P44776 EAGQIS-----------------------------------------------------F

A1A9E1 TSITSM-----------------------------------------------------L

A9MHY5 TSISAM-----------------------------------------------------L

A4W8S1 TQVWPM-----------------------------------------------------L

A1JMG4 IAVWPM-----------------------------------------------------L

Q0TK80 HPLKSL-----------------------------------------------------L

O06473 GVFDAM-----------------------------------------------------K

Q2FI61 ---TRP-----------------------------------------------------V

Q5HQE8 ---TKP-----------------------------------------------------V

Q49WE5 ---TRP-----------------------------------------------------V

Q4L523 ---TKP-----------------------------------------------------V

P33026 ---TGT-----------------------------------------------------I

P31436 ---TQP-----------------------------------------------------V

Q9S3K0 ---AAT-----------------------------------------------------L

P31675 ---ENA-----------------------------------------------------L

P31126 ---TGS-----------------------------------------------------V

Q58955 ---DVK-----------------------------------------------------K

P02920 ---NHS-----------------------------------------------------A

Q4UK37 --LQQY-----------------------------------------------------F

Q1RKF6 --LKQY-----------------------------------------------------F

Q4ULW4 --IISW-----------------------------------------------------I

Q1RI01 --IASW-----------------------------------------------------I

Q68WQ5 FFIKHY-----------------------------------------------------F

Q4UL88 FFIKRY-----------------------------------------------------Y

Q92HQ3 -IINFI-----------------------------------------------------Y

Q1RIL0 -----I-----------------------------------------------------F

Q4UMU2 -----------------------------------------------------------F

Q68W71 -----------------------------------------------------------F

Q1RHK8 -----------------------------------------------------------F

Q1LTM2 ---TSM-----------------------------------------------------L

Q2NTK5 ---APM-----------------------------------------------------L

P55705 ---TKT-----------------------------------------------------A

Q7Z3Q1 -EGFKN-----------------------------------------------------L

Q05B81 -LRHHR-----------------------------------------------------S

P76470 QLASEE-----------------------------------------------------Q

P32135 ----DG-----------------------------------------------------A

P38358 IIPMEL-----------------------------------------------------V

A4WAE6 S-----------------------------------------------------------

A6T8Y8 S-----------------------------------------------------------

Q6CZ44 S-----------------------------------------------------------

Q888L8 S-----------------------------------------------------------

Q1IB51 S-----------------------------------------------------------

A6UZY0 S-----------------------------------------------------------

Q4QP52 S-----------------------------------------------------------

Q9CM87 S-----------------------------------------------------------

O25797 T-----------------------------------------------------------

Q17YP7 T-----------------------------------------------------------

P77389 E-----------------------------------------------------------

O31577 L-----------------------------------------------------------

O34367 T-----------------------------------------------------------

A1AHK2 Q-----------------------------------------------------------

A9MWE8 Q-----------------------------------------------------------

P23910 N-----------------------------------------------------------

P31141 P-----------------------------------------------------------

P43531 KTL---------------------------------------------------------

Q8K902 NKF---------------------------------------------------------

P57648 HKC---------------------------------------------------------

Q89A23 RKI---------------------------------------------------------

P37498 GKL---------------------------------------------------------

P0A0J4 PQL---------------------------------------------------------

Q07282 INE---------------------------------------------------------

P02982 LNP---------------------------------------------------------

P02981 FNP---------------------------------------------------------

P70187 ADP---------------------------------------------------------

Q5SR56 ADP---------------------------------------------------------

P77726 GMV---------------------------------------------------------

Q89AA9 --E---------------------------------------------------------

Q8K999 -NI---------------------------------------------------------

P57538 LHS---------------------------------------------------------

Q5HIA2 DVATQPERDN--------------------------------------------------

Q4L3Q4 DVET-PARDN--------------------------------------------------

Q5HRH0 ELQKHPTREP--------------------------------------------------

P0C0L7 DREGLQDGPK--------------------------------------------------

Q47421 DRDGLKAGPG--------------------------------------------------

P0A2G3 -RKHRPDTRE--------------------------------------------------

P16482 -RRHHLAMRQ--------------------------------------------------

P0AEX3 -QQETRALKE--------------------------------------------------

P76350 QHYQAAAKKR--------------------------------------------------

P41036 TMVDILYRGE--------------------------------------------------

Q9SYQ1 AKDMQRVM---SRSHIS---D------------------------EATTDPPPPPPPPSY

Q9S735 AKDMQRVM---SVSMISQITE------------------------DSSSELEQPPSSSSY

P76230 AEKVMRSIEEGVIRQTGKPLP------------------------PVVIADDGKAPQAV-

P38055 AECQLREVEQQIEREKSIRLP------------------------PLTSYQSNSKVKVIK

Q46909 AHAI-------VHRYFG---P------------------------HVLLGDE--VVTATH

P31679 CEEM-------MIKLFG---E------------------------PVAFDEE--QPQQT-

O24723 RSALALVAPDRDIAGVDLTRA------------------------GLTLGAGEVRAKAL-

O30513 RFIMTKMVPSYQYQPDHVFVL------------------------N-SSNQNQA--QAP-

Q43975 RQILSRIAPQ-KVQGVTEFHV------------------------P-EEKVEAGTKKGV-

Q51955 RKTLSPIAPQ-VVAEAGSFSV------------------------P-EQKAVAA--RSV-

Q9I6Q3 RQVLAPIAPA-QVALARSFHV------------------------P-EQQTVQA--RNV-

P77589 -------AP-------------------------------------------------P-

P94131 E-TVKTSA----------------------------------------------------

P71369 SFTDKLRS----------------------------------------------------

O34691 ENVKSVWA----------------------------------------------------

P0AGC0 ----EELFG--------EEIS--------------------------------------E

P27669 ----GDWRH--------DALE--------------------------------------V

P96335 ----EKWRN--------DYPD--------------------------------------D

P08194 ----EEYKN--------DYPD--------------------------------------D

P37948 ----EEYRN--------DYP---------------------------------------K

P12681 ----EQMMP--------EEKV--------------------------------------V

Q5M7K3 EDMPVSYNSTD---TICKSTE--------------------------------------S

Q9WU81 QDNPEDPVNSP---YSSRESN--------------------------------------V

Q58CV5 QDQPEDPANGP---SCNKESS--------------------------------------L

Q7SY29 ----EEPLLRN---SSTNEEI--------------------------------------F

P57057 KNKPLDPEMQCLLLSDGKGSI--------------------------------------H

Q17QZ3 --NFEEDSHRPLINGAENEDE--------------------------------------A

Q3TIT8 --SSEEDSQRPLIDGAENEDD--------------------------------------Y

Q640L2 RAAQEEGANKPLI-GGNDEGD--------------------------------------D

Q5F3N0 EGSVEEDANRPLM-GDDDADD--------------------------------------D

Q09037 ---------------ATFAQD----------------------------DDGKAD--GGM

Q06222 ---------------ATFSQD----------------------------DDGKAD--GGM

P13865 ---------------AKFDGG----------------------------EEGRAE----L

P46499 QEMKEAEKKISWLSGISYVER----------------------------EDRRTEKIEEK

P54219 -----------------KVSP----------------------------ES---------

Q6NT16 -----------------Y-------------------------------ES---------

Q8R0G7 -------------GAVERH-S----------------------------GSPPLSPTSWW

Q5XGK0 -------------GALERK-T-----------------------------DRPLTNTSWS

Q7ZU13 -------------GAIEAH-P-----------------------------EHTLHRTSWL

A2CER7 -------------GASD---N----------------------------GGANMETTSYT

Q9D232 -------------GAAEKQGE----------------------------VAVRAPRSSWC

Q6ZMD2 -------------GAAETQGE----------------------------GAVGGFRSSWC

Q6GPQ3 ---------------VDDLEK----------------------------NVSSINSESEV

A5IVG9 ------DTQERKIKVPLMAQ----------------------------------------

Q5HLK7 ------DKNEPKVKIPLMAQ----------------------------------------

P46907 ------DRHEKKVKVSVKTQ----------------------------------------

P10903 ------MNDLATSKASIKEQ----------------------------------------

P37593 ------MNDIGSSKASVASQ----------------------------------------

P37758 ------MNDIASSRASIADQ----------------------------------------

Q9P3K8 ---GILDPSKPDNDSDSEEED----------------------------DNARIKKTWVL

Q6FWD4 ---NDVENHYDDNEQSRLLHA----------------------------THAQ-----QM

Q6CPY8 ---ARADAIVLEDETTLLLAS----------------------------GNKG-----EF

P22152 IAYAIPDVEKKGTETPLEPQS----------------------------QAIGQFDAFRA

Q02563 TNMRAKGHPE-RVFSVTHIK---------------------------------------T

Q496J9 TNMRARGQPE-KVFTVNKIK---------------------------------------T

Q63564 TNMRAKGTPE-KVFTVSHIK---------------------------------------T

Q1JP63 EN----GAP----MPLGKLI---------------------------------------I

Q2XWK0 EN----GAP----MPLGKLI---------------------------------------V

Q1LVS8 MN----NGV----LPEGELR---------------------------------------E

O08966 KN--RK---V-PPADLKMMC---------------------------------------L

O15245 KN--GK---L-PPADLKMLS---------------------------------------L

O77504 KN--GK---L-PPADLKMLS---------------------------------------L

A7MBE0 KN--GK---L-PPADLKMLS---------------------------------------L

Q9R0W2 KN--GK---S-VPVSLQNLT---------------------------------------P

O02713 KN--GK---S-LPASLQSLR---------------------------------------P

Q8MJI6 KN--GK---S-LPVSLQSLR---------------------------------------A

O88446 CN--GK---H-LSSNYSEIT---------------------------------------V

O75751 CN--GK---Y-LSSNYSEIT---------------------------------------V

Q9U539 MN--GKSNVD-VDELVDSMK---------------------------------------N

O76082 AN--G---IV-VPSTIFDPS---------------------------------------E

Q497L8 WN--KSSSCD--LVELLSLD---------------------------------------V

Q86VW1 WN--RASSCK--LSELLSLD---------------------------------------L

Q17QN9 WN--RTRPCK--LSEILSLD---------------------------------------H

Q95R48 FN--KVEISDEALSELLD-E---------------------------------------G

Q9Y267 VN--KKT------IPSNLLD---------------------------------------E

Q6A4L0 VN--GRP------LSPELLN---------------------------------------Q

Q9Y226 VN--RRK------LSPELMN---------------------------------------Q

Q8IVM8 RS--GMKNAR-DTLTLEILK---------------------------------------S

Q66J52 IN--GKKEAG-EKLTVEILK---------------------------------------S

Q91WU2 LN--GRPISE-DSLSQEALN---------------------------------------K

Q8IZD6 RN--RK------LKCTFSLT---------------------------------------H

Q6DFR1 RN--RR------KVTSFTLC----------------------------------------

Q6NUB3 RN--RK------KFTSFTLR----------------------------------------

Q28ES4 GN--GV------ERPVVKLK---------------------------------------S

A6NKX4 ASGVGPGDSSLEENSLATAT---------------------------------------E

P47185 K--VSPEDPWVLKQADE-INA--------------------------------------G

P13181 K--VSPEDPAVQAELDL-IMA--------------------------------------G

P23585 K--VTIEDPSIVAEMDT-IMA--------------------------------------N

Q92339 E--LSPESEIIQTNFNT-IKS--------------------------------------D

O74969 E--LFPDSEEVQNEYHR-LKS--------------------------------------S

P10870 G--VPVHDSGLLEELVE-IKA--------------------------------------T

Q12300 G--LPIEDPRLLEELVE-IKA--------------------------------------T

P42833 G--IPATDSMVIEFIDDLLEN--------------------------------------N

P49374 N--GDVNNEQVRFQLEE-IKE--------------------------------------Q

O74713 H--GDRENPDVLIEISE-IKD--------------------------------------Q

Q9BE72 ----LSDATEELTVIKSSLKD--------------------------------------E

Q5J316 ----VLDTTEELTVIKSSLKD--------------------------------------E

Q32NG5 ----TTDINEELTAIKSSIKA--------------------------------------E

Q6NWF1 GIKEVEPVEDELRAIRLAMGA--------------------------------------E

Q0P4G6 -------ETGEAGEFKPDTYD--------------------------------------R

Q6GN01 -------EAGESGEFKPDTYD--------------------------------------K

O95528 ------GEAPKLGPGRP-------------------------------------------

Q8VHD6 ------RETSKPGLVKP-------------------------------------------

Q3UHK1 ----NQTIDEEYDSIRNSIEE--------------------------------------E

Q96QE2 ----NQTIDEEYDSIKNNIEE--------------------------------------E

Q9C757 ----AEDVEQEIRALKDSVET--------------------------------------E

Q8VZR6 ----ISRLEDEIDHL-SAAEE--------------------------------------E

P30606 N-TEDEIIDQKVEELSS-LNQ------------------------SIPG----------K

P30605 D-TSEEIIERKVEELVT-LNQ------------------------SIPG----------K

Q10286 T-AHPYEIKTKLYLIQEGVRD------------------------PFSG----------S

Q04162 T-ASTYHVNSKIKQLIIELDKLRLYEDASEPLLVQSQSVIRYMDSSTSGTLSPPNIKRLS

Q01440 D-LCEFQEGDELPSVRIDYRP---------------------------------------

P11166 ----TADVTHDLQEMKEESRQ--------------------------------------M

P46896 ----TTDVSSDLQEMKEESRQ--------------------------------------M

P47843 ----TEDVAQDIQEMKDESMR--------------------------------------M

P14672 ----WADVSGVLAELKDEKRK--------------------------------------L

Q90592 ----NCDPMKEIAEMEKEKQE--------------------------------------A

P11168 ----YDDVTKDINEMRKEREE--------------------------------------A

Q5RB09 ----KADVSREVEEV-AESRV--------------------------------------Q

A4ZYQ5 ----HTDMEAELEDMRAEARA--------------------------------------E

P22732 ----WDSVDREVAEIRQEDEA--------------------------------------E

P58353 ----WHDVDAEIEEILEEDRA--------------------------------------E

Q863Y9 ----WDDVDAEMEEIQLEDEA--------------------------------------E

P43427 ----WKDVDMEMEEIRKEDEA--------------------------------------E

Q9WV38 ----WKDVHLEMEEIRKEDEA--------------------------------------E

P15686 ----TSEVDAEFADIVAAVEI--------------------------------------A

Q39525 ----TSHVEAEFADIVAAVEI--------------------------------------A

Q94AZ2 ----TDNVEPEFADLLEASRL--------------------------------------A

Q10710 ----TKHVDAEFQDMLDASEL--------------------------------------A

Q41144 ----VEDVDEEFTDLVHASED--------------------------------------S

P23586 ----VDDVSQEFDDLVAASKE--------------------------------------S

O65413 ----VDDIDDEINDLIIASEA--------------------------------------S

Q9SX48 ----ADNVDEEFQDLCDACEA--------------------------------------A

Q9LT15 ----ADNVDHEFQDLIDAVEA--------------------------------------A

Q9FMX3 ----TMEVEHEFNELCNACEA--------------------------------------A

Q9SBA7 ----VEDVDEEYESIVHACDI--------------------------------------A

Q9SFG0 ----VDDINDEYESIVHACDI--------------------------------------A

Q8L7R8 ----TNDVQDELTDLVEASS---------------------------------------G

Q93Y91 ----IADVETELAELVRSSQL--------------------------------------A

P0AE24 T----SEKARE--ELNEIRES--------------------------------------L

P0AEP1 T----SAEAKR--ELDEIRES--------------------------------------L

P96710 E----TV-AKE--ELKNIENS--------------------------------------L

P54723 D----SQAQQEIKEIKHAI----------------------------------------E

O34718 E----KRAAAELQEIEFAFKK--------------------------------------E

P46333 H----DPKDIE-MELAEMKQG--------------------------------------E

O52733 H----DQVAVN-KEINDI-QE--------------------------------------S

P0AGF4 N----TLATQAVQEIKH--SL--------------------------------------D

P21906 Q----ADPNLTIQKIKA--GF--------------------------------------D

P15729 G----DVPSR-IEEIQATVSL--------------------------------------D

Q6AWX0 P-AFVDSAAEQVNEILAELTF--------------------------------------V

Q93YP9 F-E--TDITVEVNEIKRSVAS--------------------------------------S

Q94AF9 K-D--TDVSGEAAEIQVMTKM--------------------------------------L

O04036 S-D--VDISREANTIRDTIDM--------------------------------------T

Q94KE0 D-D--ADISEEANTIKETMIL--------------------------------------F

Q9SCW7 E-N--GDILEEAAEIRETVET--------------------------------------S

Q4F7G0 E-N--SDILKEAAEIRETVEI--------------------------------------S

Q8LBI9 K-D--ADITNEADGIQVSIQA--------------------------------------L

P93051 K-K--ADISEEAAEIQDYIET--------------------------------------L

Q0WQ63 P-Q--ANITREAGEIQEYLAS--------------------------------------L

Q3ECP7 E-S--ADISYESNEIKDYTRR--------------------------------------L

Q9LTP6 N-N--TDITKEAAEIKKYMDN--------------------------------------L

Q8GXK5 A-K--SDVSDEAATILEYTKH--------------------------------------V

Q9JJZ1 S-E--EG--WEEPPV--GAEH--------------------------------------Q

P58354 Y-A--QG--WEEPPL--GAQH--------------------------------------Q

Q9NY64 S-E--QG--WEDPPI--GAE---------------------------------------Q

Q9UGQ3 T-D--VDVHWEFEQIQDNVRR--------------------------------------Q

P43562 E----SQPRDAVPEMSKIELA-------------------------------------GD

Q9FYG3 G----SHVKTAMAELYKLDL---------------------------------------D

P36035 G-E-----PLPKANFKQKMVS--------------------------------------M

P47186 P-EKEILVTLEVDKIKVTIDK--------------------------------------E

A6QLI1 ------------------------------------------------------------

Q5W8I7 ------------------------------------------------------------

A4FV52 ------------------------------------------------------------

P34644 ------------------------------------------------------------

Q66GI9 ------------------------------------------------------------

Q9FKV1 ------------------------------------------------------------

Q46916 ------------------------------------------------------------

Q91Y77 ------------------------------------------------------------

Q8TF71 ------------------------------------------------------------

A1L1W9 ------------------------------------------------------------

P36021 ------------------------------------------------------------

O35308 ------------------------------------------------------------

O95907 -------------------------EADGAGLQLREA-----------------------

Q90632 -------------------------STTDGTKKTKKA-----------------------

O35910 ------------------------------------------------------------

O15427 ------------------------------------------------------------

P57788 ------------------------------------------------------------

O15375 ------------------------------------------------------------

Q6ZSM3 -------------------------KRVSPYSSLTKE-----------------------

Q8BGC3 -------------------------KQASPYSPLTKE-----------------------

Q503M4 -------------------------LNGGPTRSAASD-----------------------

O15403 -------------------------DSIDSGVELTTSPKNVPTHTNLELEPKADMQQVLV

Q8NCK7 ------------------------------------------------------------

Q7RTY0 ------------------------------------------------------------

Q5R5M4 NINILEKSYSSEEKCRTTLANGDWKQDSLLHKNPTV-THTKEPETYKKKVAEQTYFCKQL

Q7TM99 TMNFQDKGYSSEDKC---LPNGDWGRETSLPKSLAIAAHTKEPETYKKKVVEQTNFCKQL

Q5ZJU0 NISILEKGY-IDEKCANNVP--DYKQDNILNKNVLSSINVDEKDTYKKKVVEQTNFCKQL

O15374 HCHETEES---------TIKDSTTQKAGLPSKNLTVSQNQSE-EFYNGPNRNRLLLKSDE

Q08777 ------------------------------------------------------------

Q08268 ------------------------------------------------------------

P39709 ----------------------------------------------ENQTGKSDFE----

P25621 ----------------------------------------------RDESTRLDWS----

O13880 ----------------------------------------------FLTPADKSLHSRYI

P40445 ----------------------------------------------RDDPSKGDMSNR--

P15365 ----------------------------------------------SNQQGFGN------

Q07904 ----------------------------------------------ANQTGLET------

P53322 ----------------------------------------------TMNTFDPD------

P32071 NNR--------IISEGELEDG--------------------------------------H

P38124 DDR--------YYTEQDKLDR--------------------------------------E

Q06451 NPK--------IMTEQEAQG----------------------------------------

P53283 NPK--------IMTEQEAQG----------------------------------------

Q9C0R8 DSR--------YRSFNELQI----------------------------------------

Q9C0Q6 DSR--------YRSFNELQI----------------------------------------

Q9HF77 NWG--------IYAPHEELK----------------------------------------

Q5A0E9 NWG--------IYAPHEELT----------------------------------------

Q07824 NWG--------IHAAHEDVE----------------------------------------

O59698 NQL--------VHARSEEES----------------------------------------

O74829 NYC--------LHAKSEEEP----------------------------------------

P38776 DQR--------YYAVLEVTRE--------------------------------------Q

Q9HDX4 NQKSVEVKISEITESSQQIDP--------------------------------------D

P38227 DYQRGEDETDRIENQVATLSTEKHNYVGEVRDQDSLDLESHSSPNTYDGRAGETQLQRIY

P38125 DPKWGDKKDER---------------------------ENNESP-FFEG-------NKI-

P40474 ---------------------------------------------------------NIS

O94607 FWQELDG------------------------------------------------LGIVL

Q9HE13 DFDFVGI------------------------------------------------VTITT

Q04301 NLSRIDI------------------------------------------------FGSLS

P13090 M----DW------------------------------------------------IGSVL

Q08902 LLERIDF------------------------------------------------AGSVT

P39886 APKSFDV------------------------------------------------SGIVL

P76269 SKPRFDL------------------------------------------------PSAVM

Q8Y9K8 TKPKIDI------------------------------------------------LSIIL

P28873 MTSHELII---DTLW----------------RPLEITVMEPV--------VLLINIY---

A4WFG6 VAKEK--------------------------------WGIGV--------LFLSVA----

A1JSB0 VAKEK--------------------------------WGMGV--------LFLAIA----

A8GKP6 VVKEK--------------------------------WGIGV--------LFLSIA----

P57601 -TKEK--------------------------------WNFNV--------FLLSIS----

Q8K942 -IK-N--------------------------------LNINI--------ILLSIS----

Q89A60 -IK-T--------------------------------WNFSI--------LCLSIS----

P76198 QMNSKPLV-----------------------------WLEGV--------SSVLFG----

O05390 SKAFTIMF-----------------------------ENPKV--------GIGGVV----

O34864 LKGVTILF-----------------------------TNPRV--------LTGGII----

O52717 GRAATLLY-----------------------------TNRSI--------LFSSIV----

O52718 GRAATLLY-----------------------------TNRNI--------LLSSMV----

A9MJT5 SH-AAESL-----------------------------INRFF--------LSRLAI----

A6TG19 QHDAGESL-----------------------------LNRFF--------LSRLLI----

A0L190 PRVQTESF-----------------------------KQGFF--------ISRVVI----

P0AEY8 GRDYKLVL-----------------------------KNGRF--------VAGALA----

Q7CP73 LRDFRNVF-----------------------------RNPIF--------LTGAAT----

P39386 LRDFRNVF-----------------------------CNRLF--------LFGAAT----

Q68WD6 FEVFNIII-----------------------------KDKIL--------WLYAFI----

Q4UMJ9 FEVLQVII-----------------------------KDKIL--------WLYAFI----

Q1RI77 FEVLKVVI-----------------------------RDKSL--------WLYAFI----

P32482 WSQLLLPV-----------------------------KCLNF--------WLYTLC----

P37597 FTDLL--------------------------------RSKTY--------RGNVLI----

P45123 ARNFLLLW-----------------------------KQKEV--------LGYMFA----

P28246 IGNFAALF-----------------------------RHKRV--------LSYMLA----

P31442 LTSYKTLF-----------------------------GNSGF--------NCYLLM----

P37482 --QSSSIW-----------------------------ASKMA--------WYVTIF----

P76242 --HTRGIW-----------------------------RSPLA--------WQVTLF----

P17583 --PVRVVF------------------------------TPRA--------WTLGVY----

P0C105 S-----AW-----------------------------QYRHL--------VLGAIG----

O25788 S-----LF-----------------------------SHKHF--------VFGALG----

P11551 SASLSRLA-----------------------------RIRHW--------RWAVLA----

P44776 KTAVSRLA-----------------------------QKAKY--------REGVIA----

A1A9E1 KLRQARLG-----------------------------VNGCI--------ISGIVL----

A9MHY5 KLRQARLG-----------------------------VNGCI--------ISGIVL----

A4W8S1 RLRHARLG-----------------------------VNGCI--------ISGIVL----

A1JMG4 KRRSARLG-----------------------------INGCI--------ISGVLL----

Q0TK80 A-GFRFLL-----------------------------ASPLV--------GGIALL----

O06473 ALKYKGLL-----------------------------TMAVS--------AFLYNF----

Q2FI61 KAQLRQIV----------------------------DVTKRH--------LLLFPG----

Q5HQE8 KAQLKQIV----------------------------DVTQRH--------LILFPG----

Q49WE5 KQQLGQIV----------------------------DVTKRH--------MILFPG----

Q4L523 SEQLGQIV----------------------------DVMKRH--------LVLFPG----

P33026 E-APRRNR------------------------------RDTL--------LLFVIC----

P31436 EILPSTHR----------------------------K-RDTR--------LLFVVC----

Q9S3K0 E-SPRQNR------------------------------RDTL--------LLFTAC----

P31675 S-MQGGWQ----------------------------D-SNVR--------MLFVAS----

P31126 W-SPKVLL----------------------------Q-DKAL--------LWFTCS----

Q58955 ISTLFSFE----------------------------FLKNRN----------FSSS----

P02920 FSLKLALE----------------------------LFRQPK--------LWFLSL----

Q4UK37 EVIKKSIISL---------------------------KNEQQ--------YLLLIM----

Q1RKF6 EVIKSSIISL---------------------------KNEQQ--------HLLLIM----

Q4ULW4 YAVINPFKDF----------------------------FKRE--------FAVTIL----

Q1RI01 HTVISPFKDF----------------------------FKRE--------FAITIL----

Q68WQ5 NFFKNCISAYLLKIF------------SGSHVYR-NDISLAY--------FIVLIL----

Q4UL88 S---NFLKIFLDSRF------------RGNDIKSGNDISLAY--------FIILIL----

Q92HQ3 ----NALK----------------------------PIGSVY--------FIILIL----

Q1RIL0 AFIKNILK----------------------------PIGSIS--------FIILIL----

Q4UMU2 DRFWYAFY----------------------------DFIKKP--------KWLIIV----

Q68W71 DKFFSAFY----------------------------DFIKKP--------KWIVII----

Q1RHK8 DRFWHAFY----------------------------DFIKKP--------KWLIIV----

Q1LTM2 EGIRRVLY-----------------------------DQRFV--------T-YVFT----

Q2NTK5 EGMRRVLR-----------------------------DQRFV--------T-YVLT----

P55705 KATTVSCA-----------------------------PSTGV--------SVFGLL----

Q7Z3Q1 FYRTYMLFKN--------------------------ASGKRR--------FLLCLL----

Q05B81 VIQLYV--TQ--------------------------APEKSR--------KHLALY----

P76470 QKVTSRLSDA--------------------------LRNGRV--------WQLAII----

P32135 SKNTS-MTSV--------------------------LKDKTI--------WLIAFN----

P38358 N-------------------------------------SSYS--------VVVLSI----

A4WAE6 --LKSLPL---------------------------LFRRPAL--------MSIYLL----

A6T8Y8 --LKSLPV---------------------------LFRRPAL--------VSVYIL----

Q6CZ44 --LKSLPL---------------------------LLKRPAL--------LCVYGL----

Q888L8 --LRSLPI---------------------------LFKRPRL--------MAIYLL----

Q1IB51 --LRSLPI---------------------------LFKRPAL--------VITYLL----

A6UZY0 --LRSLPM---------------------------LFRRPAL--------VCLYIL----

Q4QP52 --IASLPL---------------------------LAKRPLL--------LWLYVT----

Q9CM87 --LNSLPT---------------------------LFKRPLL--------LGLYAL----

O25797 --LASVPV---------------------------LMKRPLL--------MGIYLL----

Q17YP7 --LSSLPV---------------------------LMKRPLL--------VGIYLL----

P77389 --VKKELA---------------------------VLMRPQV--------LSALLT----

O31577 --M-NEWG---------------------------VFKHKQV--------LFSFAI----

O34367 --MRDQLK---------------------------LVTNSRL--------LLLFVI----

A1AHK2 --KQNTFR---------------------------LLQRPGV--------MAGMIA----

A9MWE8 --KQNMFS---------------------------LLQRPGV--------MAGMIA----

P23910 --LREQFH---------------------------FLRSPAP--------WLIFAA----

P31141 --LRVELA---------------------------ALKTPRL--------LLAMLL----

P43531 --FINFRL---------------------------HWRDRGL--------PLLFAE----

Q8K902 --LKNFYL---------------------------HLKNPTL--------LILFAI----

P57648 --LHRFYL---------------------------QLKNRVL--------FFLFII----

Q89A23 --LLYFIF---------------------------QWRDPVL--------SKLFFM----

P37498 --GMSLIG---------------------------HLRDRKL--------FSLFLI----

P0A0J4 --LTKINW---------------------------KVFITPV--------ILTLVL----

Q07282 --TTS-SI---------------------------REMISPL--------SGLLVV----

P02982 --LASFRW---------------------------ARGMTVV--------AALMAV----

P02981 --VSSFRW---------------------------ARGMTIV--------AALMTV----

P70187 --FASLKK---------------------------VGQDSIV--------LLICIT----

Q5SR56 --FASLKK---------------------------VGKDSTV--------LLICIT----

P77726 --KGSFSK---------------------------VLAEPRL--------LKLNFG----

Q89AA9 --ISNFFK---------------------------ILSNRVL--------CQINLS----

Q8K999 --YQKKIK---------------------------FIFNKIF--------FRFYLG----

P57538 --YKKVLN---------------------------FVLNKVF--------FRFYLG----

Q5HIA2 --INFLQI---------------------------IRFYYKD--------IFVCFVA---

Q4L3Q4 --IGFFTI---------------------------IRYYFKD--------ILVCFVA---

Q5HRH0 --IGVFTL---------------------------IRFYYKD--------IIVCFVA---

P0C0L7 --VSFKEI---------------------------ATKYWRS--------LLTCIGL---

Q47421 --VSFREI---------------------------ATHHWKS--------LLVCIGL---

P0A2G3 --I-FATI---------------------------AK-NWRI--------ITAGTLL---

P16482 --V-FATL---------------------------LA-NWQV--------VIAGMMM---

P0AEX3 --AGSLKG---------------------------LWRNRRA--------FIMVLGF---

P76350 --IPVIEA---------------------------LLRHPGA--------FLKIIAL---

P41036 --HRIANI---------------------------VMTLAAA--------TALWFCFAGN

Q9SYQ1 KLFSRCFF---------------------------RLHGRDL--------FAASFNW---

Q9S735 KLFSRRFL---------------------------SLHGRDL--------FAASANW---

P76230 -PYSALLT---------------------------GVLLKRV--------ILGSCVL---

P38055 GTFWLLFK---------------------------GEMLRRT--------LVAITVL---

Q46909 KHIKTLFS---------------------------SRYWRRT--------AFNSVFF---

P31679 -RFRDLFN---------------------------RRHFPFV--------LFVAAIW---

O24723 --FAEILC---------------------------RPLLGVT--------LLIWGVF---

O30513 --VKMIFQ---------------------------EQRAFST--------MMFWCSI---

Q43975 --FGMLFS---------------------------AKYVKGT--------VLLWVTY---

Q51955 --FAVIFS---------------------------GTYGLGT--------MLLWLTY---

Q9I6Q3 --FAVIFS---------------------------GTYSAGT--------LLLWLTY---

P77589 --LRALFA---------------------------PETATAT--------LLLWLCY---

P94131 --FKLIFQ---------------------------DKRNRNM--------FILWALT---

P71369 --FQLLIK---------------------------DKATSKI--------SLGIVVL---

O34691 --RQYI------------------------------RPTVML--------SIVWFCV---

P0AGC0 EDKETEST---DMT-------------------KWQIFVEYV--------LKNKVI----

P27669 AQQQEGA----GLS-------------------RKEILAKYV--------LLNPYI----

P96335 YNEKTYEN---DLT-------------------AKEIFVTYV--------LKNKLL----

P08194 YNEKA-EQ---ELT-------------------AKQIFMQYV--------LPNKLL----

P37948 HAFKNQEK---ELT-------------------TKEILFQYV--------LNNKFL----

P12681 LKTKNTAKAPENMS-------------------AWQIFCTYV--------LRNKNA----

Q5M7K3 NEDDSGNAHTETEH-------------------PQAISFFGA--------LRIPGV----

Q9WU81 DIAASSSKE-QGPE-------------------PEAISFLGA--------LRIPGV----

Q58CV5 ESAVTCSKE-ASAQ-------------------PSAISFFGA--------LRIPGV----

Q7SY29 NSHTSTAVEPVEDH-------------------SEAISFCGA--------LRIPGV----

P57057 PNHVVILPGDGGSG-------------------TAAISFTGA--------LKIPGV----

Q17QZ3 EPNYSIQEGNTVTQ-------------------VKAISFYQA--------CCLPGV----

Q3TIT8 EPNYSIQEDRAVVQ-------------------VKAISFHQA--------CCLPGV----

Q640L2 ESNYSIQSDDVVIT-------------------PKAIGFMQA--------CCLPGV----

Q5F3N0 EGNYSIQAADTDSQ-------------------PKAIGFFQA--------CCLPGV----

Q09037 DPNEYGWG--------------------------QML--WPL--------FMGAVTA---

Q06222 DPNEYGWG--------------------------QML--WPL--------FMGAVTA---

P13865 NPSEYGYV--------------------------EMI--PRL--------LMGCVMA---

P46499 DTKVYTIR--------------------------DLFSSWPI--------AYSTIVV---

P54219 AKGTPLF---------------------------MLLKDPYI--------LVAAGSI---

Q6NT16 DPGEHSFW--------------------------KLIALPKV--------GLIAFVI---

Q8R0G7 ADLKALAR--------------------------NP----SF--------VLSSLGF---

Q5XGK0 SDMKALLK--------------------------NP----SF--------ILSTFGF---

Q7ZU13 ADMKALCR--------------------------NP----SF--------ILSTFGF---

A2CER7 EDIKYLLK--------------------------NR----SF--------VWSSLGV---

Q9D232 EDVRYLGR--------------------------NW----SF--------VFSTLGV---

Q6ZMD2 EDVRYLGK--------------------------NW----SF--------VWSTLGV---

Q6GPQ3 TDVEKANE--------------------------GPIDQIAV--------ISSNILF---

A5IVG9 ---MKTLS--------------------------KNYKLYYL--------SYWYFI----

Q5HLK7 ---VKDLS--------------------------KNYKLYYL--------SLWYFI----

P46907 ---IKAVY--------------------------RNHVLWFL--------SLFYFI----

P10903 ---LPVLK--------------------------RGH-LWIM--------SLLYLA----

P37593 ---LPVLK--------------------------RLH-LWLL--------SLLYLA----

P37758 ---LPVLQ--------------------------RLH-LWLL--------SLLYLA----

Q9P3K8 NAETRRFL--------------------------TDHTMWCF--------ALGFFL----

Q6FWD4 --TLMKVF--------------------------RDPVLYIF--------GATIFC----

Q6CPY8 YKRIRNFF--------------------------QDYIAYAF--------LIVMLL----

P22152 NAVASPSR--------------------------KEAFNVIF--------SLATMA----

Q02563 IHQEDELIEIQSDTGTWYQRWGVRALSLGGQVWGNFLSCFSP--------EYRRIT----

Q496J9 PKQIDELIEIESDTGTWYRRCFVRIRTELYGIWLTFMRCFNY--------PVRDNT----

Q63564 PKQMDEFIEIQSSTGTWYQRWLVRFMTIFKQVWDNALYCVMG--------PYRMNT----

Q1JP63 SRQEDR---------------------------GKMRDLFTP--------HFRWTT----

Q2XWK0 SRQEDR---------------------------GKIRDLFSP--------QFRCTT----

Q1LVS8 PEVTER---------------------------GNAVTLISS--------AFRRTS----

O08966 EEDASER----RSPS----------FA--------DLFRTPS--------L-RKHT----

O15245 EEDVTEK----LSPS----------FA--------DLFRTPR--------L-RKRT----

O77504 DEDVTEK----LSPS----------LA--------DLFRTPN--------L-RKHT----

A7MBE0 EEDVTEK----LSPS----------FI--------DLFRTPN--------L-RKYT----

Q9R0W2 DEDAGKK----LNPS----------FL--------DLVRTPQ--------I-RKHT----

O02713 DEEVGEK----LKPS----------FL--------DLVRTPQ--------I-RKHT----

Q8MJI6 AEDVGEK----LNPS----------FL--------DLVRTPQ--------I-RKHT----

O88446 TDE--EV----SNPS----------CL--------DLVRTPQ--------M-RKCT----

O75751 TDE--EV----SNPS----------FL--------DLVRTPQ--------M-RKCT----

Q9U539 HQNAAEEKETKRSHN----------VT--------DLFKTPN--------L-RRKT----

O76082 LQDLSSKKQ--QSHN----------IL--------DLLRTWN--------I-RMVT----

Q497L8 TRSHNKSPHSIRKHR----------LA--------DLFHNLD--------V-AKMT----

Q86VW1 QGPVSNSPTEVQKHN----------LS--------YLFYNWS--------I-TKRT----

Q17QN9 DGSAGNKPSQVEKHT----------LS--------ELFYDWS--------I-GTRT----

Q95R48 ENSEEKAKQKLEDQE----------LDEGPPPSVWDLFCYPN--------L-RRKT----

Q9Y267 LQLPRKKVTRA---S----------VL--------DFCKNRQ--------L-CKVT----

Q6A4L0 L-VPEKTGPSG---N----------AL--------DLFRHPH--------L-RKVT----

Q9Y226 L-VPEKTGPSG---N----------AL--------DLFRHPQ--------L-RKVT----

Q8IVM8 TMKKELEAAQKKKPS----------LC--------EMLHMPN--------ICKRIS----

Q66J52 SMQREINASHNSTYS----------AL--------DLVRTP---------VVRRIS----

Q91WU2 VITME-RVSQR--PS----------YL--------DLFRTSQ--------L-RHVS----

Q8IZD6 PANRSCR----ETGS----------FL--------DLFRY-R--------VLLGHT----

Q6DFR1 PRQKDSA----HSAN----------IL--------TIYSN-A--------ILRQRT----

Q6NUB3 PRQKDST----HSAN----------II--------TIYSN-S--------ILRHRT----

Q28ES4 CPGTSSK----SAHS----------VF--------DLVKY-G--------VLRWRT----

A6NKX4 LTMLSARSPQPRYHS----------PL--------GLLRT-R--------VTWRNG----

P47185 VLAQRELGEASWKE-------------L-----F---SVKTK--------VLQRLI----

P13181 IEAEKLAGNASWGE-------------L-----F---STKTK--------VFQRLL----

P23585 VETERLAGNASWGE-------------L-----F---SNKGA--------ILPRVI----

Q92339 IEIEMAGGKARWIE-------------I-----F---GKD----------IRYRTC----

O74969 IDEEFAGGPCSWAS-------------I-----F---GKD----------IRYRTF----

P10870 YDYEASFGSSNFID-------------C-----FISSKSRPK--------QTLRMF----

Q12300 YDYEASFGPSTLLD-------------C-----FKTSENRPK--------QILRIF----

P42833 YNNEETNNESKKQS-------------LVKRNTFEFIMGKPK--------LWLRLI----

P49374 VIIDSAAKNFGYKD-------------L-----F-----RKK--------TLPKTI----

O74713 LLLEESSKQIGYAT-------------L-----F-----TKK--------YIQRTF----

Q9BE72 YQYS-------F---------------------WDLFRSKDN--------MRTRIM----

Q5J316 YQYS-------F---------------------WDLFRSKDN--------MRTRIM----

Q32NG5 YQYK-------F---------------------LDLFCSRDN--------MRARLL----

Q6NWF1 RLHG-------F---------------------LDLFQSRDN--------MLQRLL----

Q0P4G6 -QYT-------F---------------------LDLFRSKDN--------MRTRTL----

Q6GN01 -QYT-------F---------------------LDLFRSKDN--------MRTRTL----

O95528 -RYS-------F---------------------LDLFRARDN--------MRGRTT----

Q8VHD6 -QYS-------F---------------------LDLFRAQDG--------MWSRTV----

Q3UHK1 EKEATAAGPI-I---------------------CRMLSYPP---------TRRALV----

Q96QE2 EKEVGSAGPV-I---------------------CRMLSYPP---------TRRALI----

Q9C757 ILEEGSSEKINM---------------------IKLCKAKT---------VRRGLI----

Q8VZR6 --EKQRKRTVGY---------------------LDVFRSKE---------LRLAFL----

P30606 NPITKFWNMV-----------------------KELHTV-PS--------NFRALI----

P30605 NVPEKVWNTI-----------------------KELHTV-PS--------NLRALI----

Q10286 R-WQKIVKTF-----------------------KELYFN-PS--------NFRALI----

Q04162 SNTERTSNTMSSSSAYLSALRGPAPNGALASNKKKRHRMEPR--------TIRALI----

Q01440 -------------------------------------LMARD--------MRFRVV----

P11166 MREK-KVT-------------------------ILELFRSPA--------YRQPIL----

P46896 MREK-KVT-------------------------IMELFRSPM--------YRQPIL----

P47843 SQEK-QVT-------------------------VLELFRAPN--------YRQPII----

P14672 ERER-PLS-------------------------LLQLLGSRT--------HRQPLI----

Q90592 ASEK-RVS-------------------------IGQLFSSSK--------YRQAVI----

P11168 SSEQ-KVS-------------------------IIQLFTNSS--------YRQPIL----

Q5RB09 RSIR-LVS-------------------------VLELLRAPY--------VRWQVV----

A4ZYQ5 RAEG-HLS-------------------------VLHLCALRS--------LRWQLL----

P22732 KAAG-FIS-------------------------VLKLFRMRS--------LRWQLL----

P58353 KAVG-FIS-------------------------VLKLFKMRS--------LRWQVI----

Q863Y9 KAAG-IIS-------------------------VLTMFRMRS--------LRWQVI----

P43427 KAAG-FIS-------------------------VWKLFRMQS--------LRWQLI----

Q9WV38 KAAG-FIS-------------------------VWKLFTMQS--------LRWQLI----

P15686 RPITMRQS-------------------------WASL-FTRR--------YMPQLL----

Q39525 RPITMRQS-------------------------WRSL-FTRR--------YMPQLL----

Q94AZ2 KEV--KHP-------------------------FRNL-LQRR--------NRPQLV----

Q10710 NSI--KHP-------------------------FRNI-LEKR--------NRPQLV----

Q41144 KKV--EHP-------------------------WRNL-LQRK--------YRPHLS----

P23586 QSI--EHP-------------------------WRNL-LRRK--------YRPHLT----

O65413 KLV--EHP-------------------------WRNL-LQRK--------YRPHLT----

Q9SX48 KKV--DNP-------------------------WKNIFQQAK--------YRPALV----

Q9LT15 KKV--ENP-------------------------WKNI-MESK--------YRPALI----

Q9FMX3 KKV--KHP-------------------------WTNI-MQAR--------YRPQLT----

Q9SBA7 RQV--KDP-------------------------YTKL-MKPA--------SRPPFV----

Q9SFG0 SQV--KDP-------------------------YRKL-LKPA--------SRPPFI----

Q8L7R8 SDTD-SNA-------------------------FLK-LLQRK--------YRPELV----

Q93Y91 IEAR-AEL-------------------------FMKTILQRR--------YRPHLV----

P0AE24 KL--KQGG-------------------------WALFKINRN--------VRRAVF----

P0AEP1 QV--KQSG-------------------------WALFKENSN--------FRRAVF----

P96710 KI--EQMG-------------------------SLSQLFKPG--------LRKALV----

P54723 GT--AKKA-------------------------GFHDFQEPW--------IRRILF----

O34718 DQ--LEKA-------------------------TFKDLSVPW--------VRRIVF----

P46333 AE--KKET-------------------------TLGVLKAKW--------IRPMLL----

O52733 AK--IVSG-------------------------GWSELFGKM--------VRPSLI----

P0AGF4 HG--R-KT-------------------------GGRLLMFG----------VGVIV----

P21906 KA--MDKS-------------------------SAGLFAFG----------ITVVF----

P15729 H---KPRF-------------------------SDLLSRRGG--------LLPIVW----

Q6AWX0 GE--DKEV-------------------------TFGELFQGK--------CLKALI----

Q93YP9 SK--RSAV-------------------------RFVDLKRRR--------YYFPLM----

Q94AF9 EE--DSKS-------------------------SFSDMFQKK--------YRRTLV----

O04036 EN--GGET-------------------------KMSELFQRR--------YAYPLI----

Q94KE0 DE--GPKS-------------------------RVMDLFQRR--------YAPSVV----

Q9SCW7 RR--ESRS-------------------------GLKDLFNMK--------NAHPLI----

Q4F7G0 RK--ESQS-------------------------GIRDLFHIG--------NAHSLI----

Q8LBI9 EI--LPKA-------------------------RIQDLVSKK--------YGRSVI----

P93051 ER--LPKA-------------------------KMLDLFQRR--------YIRSVL----

Q0WQ63 AH--LPKA-------------------------TLMDLIDKK--------NIRFVI----

Q3ECP7 TD--LSEG-------------------------SIVDLFQPQ--------YAKSLV----

Q9LTP6 QE--FKED-------------------------GFFDLFNPR--------YSRVVT----

Q8GXK5 EQQDIDSR-------------------------GFFKLFQRK--------YALPLT----

Q9JJZ1 G----FQL---------------------------AMLRRPG--------VHKPLI----

P58354 D----FHV---------------------------AQLRRPG--------VYKPFI----

Q9NY64 S----FHL---------------------------ALLRQPG--------IYKPFI----

Q9UGQ3 S----SRV-------------------------SWAEARAPH--------VCRPIT----

P43562 FWIGVNDL-------------------------DFSKKLPRG--------SFKPLI----

Q9FYG3 KTDEPDVV-------------------------SLSELLYGR--------HSRVVF----

P36035 KRTVQK---------------------------Y--------------------------

P47186 KRLTSKEGS------------------------YSDCFEDKI--------NRRRTR----

A6QLI1 LGAME-KFKTPW----------------------RKFFTSMP--------VYAIIV----

Q5W8I7 LGAME-KYKTPW----------------------RKFFTSMP--------VYAIIV----

A4FV52 MNPVT-KFNTPW----------------------RRFFTSMP--------VYAIIV----

P34644 HPTIR-SI--PW----------------------KAIVTSKP--------VWAIIV----

Q66GI9 RISTS-GI-PPF----------------------GRLLSKMP--------TWAVIV----

Q9FKV1 HHKVT-HI--PW----------------------KKIMLSLP--------VWAIVV----

Q46916 NTKVKVPFSVKWGQI-------------------KQLLGSRM--------MIGVYI----

Q91Y77 FFSRRKLSPPKKIFN-------------------FALFK-ET--------AYAVWA----

Q8TF71 LFSRKKFSPPKKIFN-------------------FAIFK-VT--------AYAVWA----

A1L1W9 -----RCPPLSRIFN-------------------VNIWK-SL--------GYRIWA----

P36021 TLHQRFLAQLRKYFN-------------------MRVFR-QR--------TYRIWA----

O35308 -----RRRR---LLD-------------------VAVCT-DR--------AFVVYV----

O95907 SPRVRPRRR---LLD-------------------LAVCT-DR--------AFAVYA----

Q90632 KKKPKKGKK---LLD-------------------FSIFS-NR--------GFIIYT----

O35910 ----RPSPR---LLD-------------------LSVFR-DR--------GFLIYA----

O15427 ----RPSRR---LLD-------------------LSVFR-DR--------GFVLYA----

P57788 ----KAKKK---LLD-------------------FSVFK-DG--------GFVIYT----

O15375 ----RTIQR---HLA-------------------FDILRHNT--------GYCVYI----

Q6ZSM3 WAQTCLCCC---LQQ-------------------EYSFLLMS--------DFVVLA----

Q8BGC3 CTETRLCCS---LQQ-------------------EYGFLLMS--------DFVVLA----

Q503M4 AKQR--CFQ---SMQ-------------------EYHFLLMP--------DFLVLA----

O15403 KTSPRPSEKKAPLLD-------------------FSILK-EK--------SFICYA----

Q8NCK7 ------------ALG-------------------LSLFT-RR--------AFSIFA----

Q7RTY0 ------------QL--------------------TSLLH-HG--------PFLRYT----

Q5R5M4 AKRKWQLYKNYCGET-------------------VALFK-NK--------VFSALF----

Q7TM99 AKRKWQLYRNYCGET-------------------ASLFK-NK--------VFSALF----

Q5ZJU0 AKRKWQLYLNYWEET-------------------VVLFK-NR--------VFSALF----

O15374 ESDKVISWSCKQLFD-------------------ISLFR-NP--------FFYIFT----

Q08777 RWRYILRVYILQCFD-------------------AKAFL-DM--------KYLFCV----

Q08268 KW------YISSVFN-------------------WRYFL-EG--------KFLFVA----

P39709 ------TKVFDIKLW-------------------KTIFSDWK--------IYILTL----

P25621 ------TIPRVLKRW------------------------HWW--------MFSL-V----

O13880 AEMNVGKRWQWSDLL-------------------KS-CLDLR--------VWPFIL----

P40445 ------QPVTFKEIL-------------------YT-LTEFD--------LWPLFI----

P15365 ------HEIKKYQII-------------------EA-LKDVR--------TWLYFL----

Q07904 ------KKFKKQQVK-------------------ELFLHDKF--------TWPMLL----

P53322 ------EKFEWFQVW-------------------QA-VKDVK--------TWASAV----

P32071 KTTSQVVS---SLLW----------------RPLEITMLEPV--------VFLIDIY---

P38124 VDARTFLI---NTLY----------------RPLKMIIKEPA--------ILAFDLY---

Q06451 VSMGEMMR---ACLL----------------RPLYFSVTEPV--------LVATCFY---

P53283 VSMSEMMR---ACLL----------------RPLYFAVTEPV--------LVATCFY---

Q9C0R8 RSFGEVAK---TSLL----------------RP-FVLLSELI--------VFLMTIY---

Q9C0Q6 RTFAQVAK---TSLL----------------RP-FVLLSELI--------VFLVTMY---

Q9HF77 LSMKEIVE---NNIA----------------RPLKMLFTEPI--------LFLVSLY---

Q5A0E9 LSLKEIVE---NNIA----------------RPLKMLFTESI--------LFLVSIY---

Q07824 LSIKDIVQ---KTVT----------------RPIIMLFVEPL--------LLFVTIY---

O59698 LSARDIIM---NYLL----------------IPLKMLATEPI--------VFLVSLY---

O74829 LEFSYFFH---KYFT----------------FPLRLLIFEPI--------LLVVSTY---

P38776 TSLLSAIF---LSTK----------------RPFGLLLRDRM--------MGVLCFY---

Q9HDX4 KSFAEVVHILVTTIR----------------RPLHLLCTQPI--------MILISLI---

P38227 TEASRSLYEYQLDDSGIDATTAQVTRIRSTDPKLARSIRENSLRKLQTNLEEQVKKVLSS

P38125 --SHRRLF----PDIGI-----------------RKPVNNDAF------FQENFPK----

P40474 GNGSVT----------------------------PKSYLNRAPILVLPTVRKSLH-----

O94607 FVSGFTLL--------------------------LLPFSHSSQVVSPDSTILTLFT----

Q9HE13 GVVLFLLG--------------------------LNIGSTTGHW----AHANVLCY----

Q04301 LVATISGV--------------------------LFLCS---------SQLNKLYL----

P13090 GVIGLILL--------------------------NFVWNQAP--ISGWNQAYIIVI----

Q08902 GVVGLILF--------------------------NFAWNQGP--VVGWQTPYTYAL----

P39886 LSGAMFCL--------------------------VWGLIKAP--AWGWGDLRTLGF----

P76269 -NALTFGL--------------------------LITALSGF--AQGQSLTLIAAE----

Q8Y9K8 STLGFGGV--------------------------VFAFSSAG--ESGWGSATVLVS----

P28873 -----IAMVYSILYLFFEVFPIYFVGVKHFTLVELGTTY---------------------

A4WFG6 -----ALCYILGQLGFISWVPEYA--------KGLGM-----S-----------------

A1JSB0 -----ALCYILGQLGFIQWVPEYAT-------KTFNM-----D-----------------

A8GKP6 -----ALCYILGQLGFIQWVPEYAT-------KSFGM-----D-----------------

P57601 -----ALLYILGQLGFISWVPQYAT-------EIMNI-----D-----------------

Q8K942 -----ALLYILGQLSFISWVPQYTT-------EIINI-----N-----------------

Q89A60 -----ALLYILGQLSFISWMPEYTM-------KYIHI-----S-----------------

P76198 -----VAAFSTFYV-IVVWMPKYAM-------AFAGM-----S-----------------

O05390 -----KTINAIGQFGFAIFLP-TYL-------ARYGY-----S-----------------

O34864 -----RIINSIGTYGFPVFLP-MHM-------AQHGI-----S-----------------

O52717 -----RIINTLSLFGFAVIMPMMFV-------DELGF-----T-----------------

O52718 -----RIINTLSLFGFAVIMPMMFV-------DELGF-----S-----------------

A9MJT5 -----TTLSVSVILTFVNASPVLLM-------EVMEF-----S-----------------

A6TG19 -----TTLSVTVILTYVNVSPVLMM-------EEMGF-----D-----------------

A0L190 -----TTLGVTTILSYVNVSPMLIM-------GQMGF-----D-----------------

P0AEY8 -----LGFVSLPLLAWIAQSPIIII-------TGEQL-----S-----------------

Q7CP73 -----LSLSYIPMMSWVAVSPVILI-------DAGGM-----S-----------------

P39386 -----ISLSYIPMMSWVAVSPVILI-------DAGSL-----T-----------------

Q68WD6 -----IGAFNGIYYGFFIEAPFILI-------DKMKV-----L-----------------

Q4UMJ9 -----IGAFNGIYYGFFIEAPFIFI-------DKMKV-----S-----------------

Q1RI77 -----IGAFNGIYYGFYIEAPFIFI-------DKMKV-----A-----------------

P32482 -----YAAGMGSFFVFFSIAPGLMM-------GRQGV-----S-----------------

P37597 -----YAACSASFFAWLTGSP-FIL-------SEMGY-----S-----------------

P45123 -----ASFSFGGLFAFVTAGSIVYI-------GIYGV-----P-----------------

P28246 -----SGFSFAGMFSFLSAGPFVYI-------EINHV-----A-----------------

P31442 -----LIGGLAGIAAFEACSGVLMG-------AVLGL-----S-----------------

P37482 -----MGLQSFLFYSSIAWFPEILR-------S-HGI-----D-----------------

P76242 -----LGINSLVYYVIIGWLPAILI-------S-HGY-----S-----------------

P17583 -----FGLINGGYASLIAWLPAFYI-------E-IGA-----S-----------------

P0C105 -----IFVYVGAEVSVGSFLVNFLSD-----PTVAGL-----S-----------------

O25788 -----IFFYVGGEVAIGSFLV--LSF-----EKLLNL-----D-----------------

P11551 -----QFCYVGAQTACWSYLIRY-AV-----EEIPGM-----T-----------------

P44776 -----QAFYVGVQIMCWTFIVQY-A------ERL-GF-----T-----------------

A1A9E1 -----GSLYGLMPLY-------L---------NHKGV-----S-----------------

A9MHY5 -----GSLYGLMPLY-------L---------KHQGM-----A-----------------

A4W8S1 -----GSLYGLMPLY-------L---------NHQGV-----S-----------------

A1JMG4 -----GSLYGLLPLY-------L---------SHQGM-----S-----------------

Q0TK80 -----GGLLTMASAVRVLYPALA---------DNWQM-----S-----------------

O06473 -----GFFILLAYSPFVLD---L---------DEHGL-----G-----------------

Q2FI61 -----ILLQGAAIAALVPILPTYATK-----VINVST-----I-----------------

Q5HQE8 -----ILLQGAAIAALVPILPKYATQ-----VVKVST-----V-----------------

Q49WE5 -----ILLQGASITALVPILPTYATK-----VVGVST-----L-----------------

Q4L523 -----ILLQGASISALLPILPTYATK-----VVGVST-----I-----------------

P33026 -----TLMWGS-NSLYIINMPLF--I-----INELHL-----P-----------------

P31436 -----SMMWAA-NNLYMINMPLF--I-----IDELHL-----T-----------------

Q9S3K0 -----TLMWTC-NGIYLINMPLY--L-----VNELRL-----P-----------------

P31675 -----TLMWTC-NTMYIIDMPLW--I-----SSELGL-----P-----------------

P31126 -----GFLASFVSGAFASCISQYVMV-----IADGDF-----A-----------------

Q58955 -----FIINVS---NVMINAGIYAYL-----ALYAIN-----Y-----------------

P02920 -----YVIGVSCTYDVFDQQFANFFT-----SFFATG-----------------------

Q4UK37 -----LFVFLYKAADSIPMAMSSPLF-----LDLSFT-----T-----------------

Q1RKF6 -----LFVFLYKAADSIPMAMSSPLL-----LDLSFT-----T-----------------

Q4ULW4 -----LAVIFFKLGDAMLGAVASPFY-----IELGYT-----K-----------------

Q1RI01 -----FAVIFFKLGDSMLLSIASPFY-----IELGYT-----K-----------------

Q68WQ5 -----IFLVLYRLPDNLINVMINPFL-----LHLGYN-----A-----------------

Q4UL88 -----IFLVLYRLPDNLINVMINPFL-----LHLEYD-----A-----------------

Q92HQ3 -----IFLVLYRLPDNLINVMINPFL-----LHLEYD-----A-----------------

Q1RIL0 -----IFLILYRLPDNFINVMINPFL-----LHLNYD-----A-----------------

Q4UMU2 -----SFMLLYRLQDNFLSIMPNMFY-----LDIGYT-----K-----------------

Q68W71 -----SFMLLYRLQDSFLSIMPNMFY-----LDIGYT-----K-----------------

Q1RHK8 -----GFMLLYRLQDNFLAVMPNMFY-----LDIGYT-----K-----------------

Q1LTM2 -----LTGYYILSVQVMLILP--IRV-----NEVAG------Q-----------------

Q2NTK5 -----LTGYYMLSVQVMLMLP--IRI-----NEVAG------Q-----------------

P55705 -----ALMGLLLASRMVLQVPFSLYM-----SEVYGS-----Q-----------------

Q7Z3Q1 -----LFTVITYFFVVIGIAPIFILY-----ELDSPLCWNEVF-----------------

Q05B81 -----SLAIFVMITVHLGAQDILTLY-----ELSAPLCWDSRL-----------------

P76470 -----YLTIQVAVYGLIFFLPTQVAA-----LLGTK------V-----------------

P32135 -----VFFVYAVYCGLTFFIPFLKNI-----YL-LP------V-----------------

P38358 -----SILVGFASYAYLFTLPLFFQI-----VLGD-------S-----------------

A4WAE6 -----TVVVVTAHYTAYSYIEPFV-----QVVAGF-SANF--------------------

A6T8Y8 -----TVVVVTAHYTAYSYIEPFV-----QTVAGL-SGNF--------------------

Q6CZ44 -----TVMIVTAHFTAYSYIEPFI-----LKVALL-SENF--------------------

Q888L8 -----TAIVVTAHFTAYSYIEPFT-----QTVSRL-SGEM--------------------

Q1IB51 -----VTLVITAQFTAYSYIEPFA-----LHVAQI-GGER--------------------

A6UZY0 -----TVVVISAQFTAYSYIEPFA-----RQVAQM-GGEA--------------------

Q4QP52 -----TAIVISAHFTAYTYIEPFM-----IDVGHL-DPNF--------------------

Q9CM87 -----TMIIISAHFTAYSYIEPFM-----LNISTM-SHSM--------------------

O25797 -----VIMVISGHFTTYSYIEPFI-----IQISQF-SPDI--------------------

Q17YP7 -----VILAISGHFTTYSYIEPFI-----IQISQF-SPEV--------------------

P77389 -----TVLGAGAMFTLYTYISPVL-----QSITHA-TPVF--------------------

O31577 -----TILGYSGVFIAYTFIEPIL-----RHSAGF-STVG--------------------

O34367 -----TALGYGGTFVVFTYLSPLL-----QEVTGF-KAGT--------------------

A1AHK2 -----IFMSFAGQFAFFTYIRPVY-----MNLAGF-GVDG--------------------

A9MWE8 -----IFMSFAGQFAFFTYIRPVY-----MNLAGF-DVDG--------------------

P23910 -----TMFGNAGVFAWFSYVKPYM-----MFISGF-SETA--------------------

P31141 -----GALVNAATFASFTFLAPVV-----TDTAGL-GDLW--------------------

P43531 -----GFLLMGSFVTLFNYIGYRL-----MLSPWH-VSQA--------------------

Q8K902 -----GFMLMGSFITIFNYISYRL-----MLSPFF-LSSS--------------------

P57648 -----GFILMGSFVTIFNYIGYRL-----MLEPFF-LCQS--------------------

Q89A23 -----GCILMGSFITLFNYVGYRL-----ISQPFF-LGQT--------------------

P37498 -----GFLLLGSNVALFNYIVYVL-----LGPPYS-LNKA--------------------

P0A0J4 -----SFGLSAFE----TLYSLYT-----ADKVNY-SPKD--------------------

Q07282 -----FFIIQLIGQIPATLWVLFG-----EERFAW-DGVM--------------------

P02982 -----FFIMQLVGQVPAALWVIFG-----EDRFHW-DATT--------------------

P02981 -----FFIMQLVGQVPAALWVIFG-----EDRFRW-SATM--------------------

P70187 -----VFLSYLPEAGQYSSFFLYL-----KQIMKF-SPES--------------------

Q5SR56 -----VFLSYLPEAGQYSSFFLYL-----RQVIGF-GSVK--------------------

P77726 -----IMCLHILLMSTFVALPGQL-----AD-AGFPAAEH--------------------

Q89AA9 -----VFLIHFFLMCNFIIIPVEL-----KKIFEFFEYVP--------------------

Q8K999 -----VFLLHFLLTMNFLIIPYEF------ELSGLALHYH--------------------

P57538 -----VFFLHFLLMIKFTMIPNQF------EISGFSLDNH--------------------

Q5HIA2 -----VVFFNVTNYMVTAYLPTYL-----EQVIKLDATT---------------------

Q4L3Q4 -----VVFFNVTNYTVTAYLPTYL-----GQIVKIDETT---------------------

Q5HRH0 -----VAFFNVTNYMVTAYLPSYL-----EGVIKLNGTT---------------------

P0C0L7 -----VIATNVTYYMLLTYMPSYL-----SHNLHYSEDH---------------------

Q47421 -----VIATNVTYYMLLTYMPSYL-----SHSLHYSENH---------------------

P0A2G3 -----VAMTTTTFYFITVYTPTYG-----RTVLNLSARD---------------------

P16482 -----VAMTTTAFYLITVYAPTFG-----KKVLMLSASD---------------------

P0AEX3 -----TAAGSLCFYTFTTYMQKYL-----VNTAGMHANV---------------------

P76350 -----RLCELLTMYIVTAFALNYS-----TQNMGLPREL---------------------

P41036 L--QNAAIVAVLGLLCAAIFISFM-----VQSAGKRWPT---------------------

Q9SYQ1 -----FLVD-IVFYTSNLLLSHIFSHYSKKPSTAENVYDAA-------------------

Q9S735 -----FLVD-VVFYTSNLLLSQIF-NFSNKPLNSTNVYDSA-------------------

P76230 -----IAMN-VVQYTLINWLPTIF------MTQGINL-KD--------------------

P38055 -----IAMN-ISLYTITVWIPTIF------VNSGIDV-DK--------------------

Q46909 -----VCLV-IPWFVIYTWLPTIA------QTIGL---ED--------------------

P31679 -----TCQV-IPMFAIYTFGPQIV------GLLGLGVGKN--------------------

O24723 -----FVVQ-GSGLLVLQYMPMLL----QAPAPGLSTVE---------------------

O30513 -----FMTL-IMVYALGNWLPKLM----I--EAGYNLSK---------------------

Q43975 -----FMGL-VMIYLLTSWLPTLM----R--ETGASLER---------------------

Q51955 -----FMGL-VIVYLLTSWLPTLM----R--DSGASMEQ---------------------

Q9I6Q3 -----FMGL-VIVYLLTSWLPTLM----R--DSGASLEQ---------------------

P77589 -----FFTL-LVVYMLINWLPLLL----V--EQGFQPSQ---------------------

P94131 -----AGFLQFGYYGVNNWMPSYL----ES-ELGMKFKE---------------------

P71369 -----TSVQNFGYYGIMIWLPNFL----SK-QLGFSLTK---------------------

O34691 -----V----FSYYGMFLWLPSVM----LL-K-GFSMIQ---------------------

P0AGC0 -----WLLCFANIFLYVVRIGIDQWS-----TVYAFQELKLSK-----------------

P27669 -----WLLSLCYVLVYVVRAAINDWG-----NLYMSETLGVDL-----------------

P96335 -----WYIAIANVFVYLIRYGVLKWS-----PVYLSEVKHFNI-----------------

P08194 -----WYIAIANVFVYLLRYGILDWS-----PTYLKEVKHFAL-----------------

P37948 -----WYIAFANVFVYFVRYGVVDWA-----PTYLTEAKGFSP-----------------

P12681 -----WYISLVDVFVYMVRFGMISWL-----PIYLLTVKHFSK-----------------

Q5M7K3 -----VEFSLCLLFAKLVSYTFLYWL-----PLYIANDAQFDP-----------------

Q9WU81 -----IEFSLCLLFAKLVSYTFLYWL-----PLYIFNVAHFSA-----------------

Q58CV5 -----VEFSLCLLFAKLVSYTFLYWL-----PLYISNVVHFTA-----------------

Q7SY29 -----VEFSLCLLFAKLVSYTFLYWL-----PLYIANVAHFDP-----------------

P57057 -----IEFSLCLLFAKLVSYTFLFWL-----PLYITNVDHLDA-----------------

Q17QZ3 -----IAYSLAYACLKLVNYSFFFWL-----PFYLSNNFGWKE-----------------

Q3TIT8 -----IPYSLAYACLKLVNYSFFFWL-----PFYLSNNFGWKE-----------------

Q640L2 -----LLYSLAYACLKLVNYSFFFWL-----PYYLSNNFKWKE-----------------

Q5F3N0 -----VLYSLAYACLKLVNYSFFFWL-----PFYLSNNFGWKE-----------------

Q09037 -----GTLQLTGINAVMNYAPKI------TENLGM-------------------------

Q06222 -----GTLQLTGINAVMNYAPKI------TENLGM-------------------------

P13865 -----GTLQLTGINAVMNYAPTI------MGSLGL-------------------------

P46499 -----GSLWFS--TSVSSFGADLN-----SGNLAG-------------------------

P54219 -----CFAN-MGVAILEPTLPIWM-----MQTMCS-------------------------

Q6NT16 -----NSLS-SCFGFLDPTLSLFV-----LEKFNL-------------------------

Q8R0G7 -----TSVAFVTGSLAL-WAPAFL-----LRSRVVLGETPPCL-----------------

Q5XGK0 -----TTVAFVTGALAL-WGPTYL-----MRSRMVIYKSKPC------------------

Q7ZU13 -----TAVAFVTGSLAL-WAPAFL-----FRAGVFTGVKQPCF-----------------

A2CER7 -----TAMAFVTGALAF-WTPTFL-----SRAQVTQGLKQPCK-----------------

Q9D232 -----TAIAFVTGALGF-WAPKFL-----FEARVVHGLQLPCF-----------------

Q6ZMD2 -----TAMAFVTGALGF-WAPKFL-----LEARVVHRLQPPCF-----------------

Q6GPQ3 -----FVVLFVFAIFET-ISTPLT-----MDMYAWTRTQA--------------------

A5IVG9 -----TFG---AFVAFGIFLPNYLVNHFGIDKVDAGIRS---------------------

Q5HLK7 -----TFG---AFVAFGIFLPNFLVDHFSIDKVDAGIRS---------------------

P46907 -----TFG---AFVAFTIYLPNFLVEHFGLNPADAGLRT---------------------

P10903 -----TFG---SFIGFSAGFAMLSKTQF----PDVQILQ---------------------

P37593 -----TFG---SFIGFSAGFAMLAKTQF----PDVNILQ---------------------

P37758 -----TFG---SFIGFSAGFAMLAKTQF----PDVNILR---------------------

Q9P3K8 -----MIGPGEAFINNLGTVIKTLYPPHLKFVGEPTSAA---------------------

Q6FWD4 -----ALGPLEMFIANMGSLTNVLAGGH-----EPAMSS---------------------

Q6CPY8 -----SMGVMEMFNTNMVNLTSLILG--------PGSSF---------------------

P22152 -----VAVPYACSFGSELAINSILGDYYDKNFPYMGQTQ---------------------

Q02563 -----LMMMGVWFTMSFSYYGLTVWF-----PDMIRHLQAVDYAARTKVFPGERVEHVTF

Q496J9 -----IKLTIVWFTLSFGYYGLSVWF-----PDVIKPLQSDEYALLTRNVERDKYANFTI

Q63564 -----LILAVVWFTMALSYYGLTVWF-----PDMIRYFQDEEYKSKMKVFFGEHVHGATI

Q1JP63 -----LLLWFIWFSNAFSYYGLVLLT-----TEL---FQAGDV--------------C--

Q2XWK0 -----LLLWFIWFSNAFSYYGLVLLT-----TEL---FQAGDV--------------C--

Q1LVS8 -----LLLWYSWFVASFSYYGSVLSS-----SEL---LEKNLL--------------CVT

O08966 -----LILMYLWFSCAVLYQGLIMHV-----GATG-------------------------

O15245 -----FILMYLWFTDSVLYQGLILHM-----GATS-------------------------

O77504 -----FILMFLWFTCSVLYQGLILHM-----GATG-------------------------

A7MBE0 -----FILMYLWFTSSVVYQGLIMHV-----GATG-------------------------

Q9R0W2 -----LILMYNWFTSSVLYQGLIMHM-----GLAG-------------------------

O02713 -----LILMYNWFTSAVLYQGLVMHM-----GLAG-------------------------

Q8MJI6 -----CILMYNWFTSSVLYQGLIMHL-----GLAG-------------------------

O88446 -----LILMFAWFTSAVVYQGLVMRL-----GLIG-------------------------

O75751 -----LILMFAWFTSAVVYQGLVMRL-----GIIG-------------------------

Q9U539 -----LIVTYIWVMNAIIYNGLTLNV-----SNLP-------------------------

O76082 -----IMSIMLWMTISVGYFGLSLDT-----PNLH-------------------------

Q497L8 -----LIVWLDWFTANLGYYMFGKEV-----IRRK-------------------------

Q86VW1 -----LTVWLIWFTGSLGFYSFSLNS-----VNLG-------------------------

Q17QN9 -----LILWLIWFTGCLGFYTFSLNS-----VHLG-------------------------

Q95R48 -----LLIFLDWLVTSGVYYGLSWNT-----SNLG-------------------------

Q9Y267 -----LVMSCVWFTVSYTYFTLSLRM-----RELG-------------------------

Q6A4L0 -----LILIAVWFVDSLVYYSLSFQV-----GDFG-------------------------

Q9Y226 -----LIIFCVWFVDSLGYYGLSLQV-----GDFG-------------------------

Q8IVM8 -----L-LSFTRFANFMAYFGLNLHV-----QHLG-------------------------

Q66J52 -----FCISCTWFSTSFAYYGLALDL-----QSFG-------------------------

Q91WU2 -----LCCMMMWFGVNFSYYGLTLDA-----SGLG-------------------------

Q8IZD6 -----LILMFIWFVCSLVYYGLTLSA-----GDLG-------------------------

Q6DFR1 -----LIMMWVWFVCSLVYYGLTLSS-----GDLG-------------------------

Q6NUB3 -----LVMMWVWFVCSLVYYGLTLSS-----GDLG-------------------------

Q28ES4 -----ILLMYIWYVCSLVYYGLTLNA-----GELK-------------------------

A6NKX4 -----LILGFSSLVGGGIRASFRRSL-----APQV-------------------------

P47185 -----TGILVQTFLQLTGENYFFFYG-----TTIFKSVGL--T-----------------

P13181 -----MGVFVQMFQQLTGNNYFFYYG-----TVIFKSVGL--D-----------------

P23585 -----MGIMIQSLQQLTGNNYFFYYG-----TTIFNAVGM--K-----------------

Q92339 -----LGFLVMLFRELIGNNYYFYYA-----TQVFKGTGM--T-----------------

O74969 -----LGMFVMSLQQLTGNNYFFYYG-----FSVMQGAGI--N-----------------

P10870 -----TGIALQAFQQFSGINFIFYYG-----VNFFNKTGV--S-----------------

Q12300 -----TGIAIQAFQQASGINFIFYYG-----VNFFNNTGV--D-----------------

P42833 -----IGMMIMAFQQLSGINYFFYYG-----TSVFKGVGI--K-----------------

P49374 -----VGVSAQMWQQLCGMNVMMYYI-----VYIFNMAGYTGN-----------------

O74713 -----TAIFAQIWQQLTGMNVMMYYI-----VYIFQMAGYSGN-----------------

Q9BE72 -----IGLTLVFFVQITGQPNILFYA-----STVLKSVGFQSN-----------------

Q5J316 -----IGLTLVFFVQITGQPNILFYA-----STVLKSVGFQSN-----------------

Q32NG5 -----IGLTLSFFVQITGQPNILFYA-----STVLKSVGFQST-----------------

Q6NWF1 -----VGAALVFLQQATGQPNILAYA-----STVLSSVGFHGN-----------------

Q0P4G6 -----LGLGLVLFQQFTGQPNVLYYA-----STIFQSVGFQSN-----------------

Q6GN01 -----LGLGLVLFQQFTGQPNVLYYA-----STIFRSVGFQSN-----------------

O95528 -----VGLGLVLFQQLTGQPNVLCYA-----STIFSSVGFHGG-----------------

Q8VHD6 -----VGLGLVLFQQLTGQPNVLYYA-----STIFRSVGFHGG-----------------

Q3UHK1 -----VGCGLQMFQQLSGINTIMYYS-----ATILQMSGVEDD-----------------

Q96QE2 -----VGCGLQMFQQLSGINTIMYYS-----ATILQMSGVEDD-----------------

Q9C757 -----AGVGLQVFQQFVGINTVMYYS-----PTIVQLAGFASN-----------------

Q8VZR6 -----AGAGLQAFQQFTGINTVMYYS-----PTIVQMAGFHSN-----------------

P30606 -----IGCGLQAIQQFTGWNSLMYFS-----GTIFETVGFKN------------------

P30605 -----IGCGLQAIQQFTGWNSLMYFS-----GTIFETVGFKN------------------

Q10286 -----LACGLQAMQQLSGFNSLMYFS-----STIFEVVGFNN------------------

Q04162 -----VGCMLMFFQQITGFNAFMYYA-----AIIFSKFNIKN------------------

Q01440 -----LSSGLQIIQQFSGINTIMYYS-----SVILYDAGFRDA-----------------

P11166 -----IAVVLQLSQQLSGINAVFYYS-----TSIFEKAGV--Q-----------------

P46896 -----IAIVLQLSQQLSGINAVFYYS-----TSIFEKSGV--E-----------------

P47843 -----ISIMLQLSQQLSGINAVFYYS-----TGIFKDAGV--Q-----------------

P14672 -----IAVVLQLSQQLSGINAVFYYS-----TSIFETAGV--G-----------------

Q90592 -----VALMVQISQQFSGINAIFYYS-----TNIFQRAGV--G-----------------

P11168 -----VALMLHVAQQFSGINGIFYYS-----TSIFQTAGI--S-----------------

Q5RB09 -----TVIVTMACYQLCGLNAIWFYT-----NSIFGKAGIPPA-----------------

A4ZYQ5 -----SIIVLMAGQQLSGINAVNYYA-----DTIYTSAGVEAA-----------------

P22732 -----SIIVLMGGQQLSGVNAIYYYA-----DQIYLSAGVPEE-----------------

P58353 -----SIIVLMAGQQLSGVNAIYYYA-----DQIYLSAGVNED-----------------

Q863Y9 -----SIIILMGGQQLSGVNAIYYYA-----DQIYLSAGVKDQ-----------------

P43427 -----STIVLMAGQQLSGVNAIYYYA-----DQIYLSAGVKSN-----------------

Q9WV38 -----SMIVLMAGQQLSGVNAIYYYA-----DQIYLSAGVKSD-----------------

P15686 -----TSFVIQFFQQFTGINAIIFYV-----PVLFSSLGSANS-----------------

Q39525 -----TSFVIQFFQQFTGINAIIFYV-----PVLFSSLGSASS-----------------

Q94AZ2 -----IAVALQIFQQCTGINAIMFYA-----PVLFSTLGFGSD-----------------

Q10710 -----MAIFMPTFQILTGINIILFYA-----PPLFQSMGFGGN-----------------

Q41144 -----MAIAIPFFQQLTGINVIMFYA-----PVLFDTIGFGSD-----------------

P23586 -----MAVMIPFFQQLTGINVIMFYA-----PVLFNTIGFTTD-----------------

O65413 -----MAILIPAFQQLTGINVIMFYA-----PVLFQTIGFGSD-----------------

Q9SX48 -----FCSAIPFFQQITGINVIMFYA-----PVLFKTLGFADD-----------------

Q9LT15 -----FCSAIPFFQQITGINVIMFYA-----PVLFKTLGFGDD-----------------

Q9FMX3 -----FCTFIPFFQQLTGINVIMFYA-----PVLFKTIGFGND-----------------

Q9SBA7 -----IGMLLQFFQQFTGINAIMFYA-----PVLFQTVGFGND-----------------

Q9SFG0 -----IGMLLQLFQQFTGINAIMFYA-----PVLFQTVGFGSD-----------------

Q8L7R8 -----MALVIPFFQQVTGINVVAFYA-----PVLYRTVGFGES-----------------

Q93Y91 -----VAVVIPCFQQLTGITVNAFYA-----PVLFRSVGFGSG-----------------

P0AE24 -----LGMLLQAMQQFTGMNIIMYYA-----PRIFKMAGFTTT-----------------

P0AEP1 -----LGVLLQVMQQFTGMNVIMYYA-----PKIFELAGYTNT-----------------

P96710 -----IGILLALFNQVIGMNAITYYG-----PEIFKMMGF-GQ-----------------

P54723 -----IGIGIAIVQQITGVNSIMYYG-----TEILREAGF-QT-----------------

O34718 -----IGLGIAIVQQITGVNSIMYYG-----TEILRNSGF-QT-----------------

P46333 -----IGVGLAIFQQAVGINTVIYYA-----PTIFTKAGL-GT-----------------

O52733 -----IGIGLAIFQQVMGCNTVLYYA-----PTIFTDVGF-GV-----------------

P0AGF4 -----IGVMLSIFQQFVGINVVLYYA-----PEVFKTLGA-ST-----------------

P21906 -----AGVSVAAFQQLVGINAVLYYA-----PQMFQNLGF-GA-----------------

P15729 -----IGMGLSALQQFVGINVIFYYS-----SVLWRSVGF-TE-----------------

Q6AWX0 -----IGGGLVLFQQITGQPSVLYYA-----PSILQTAGFSAA-----------------

Q93YP9 -----VGIGLLALQQLGGINGVLFYS-----STIFES-A--GV-----------------

Q94AF9 -----VGIGLMLIQQLSGASGITYYS-----NAIFRK-A--GF-----------------

O04036 -----IGVGLMFLQQLCGSSGVTYYA-----SSLFNK-G--GF-----------------

Q94KE0 -----IGVGLMLLQQLSGSSGLMYYV-----GSVFDK-G--GF-----------------

Q9SCW7 -----IGLGLMLLQQFCGSSAISAYA-----ARIFDT-A--GF-----------------

Q4F7G0 -----IGLGLMLLQQFCGSAAISAYA-----ARIFDK-A--GF-----------------

Q8LBI9 -----IGVSLMVFQQFVGINGIGFYA-----SETFVK-A--GF-----------------

P93051 -----IAFGLMVFQQFGGINGICFYT-----SSIFEQ-A--GF-----------------

Q0WQ63 -----VGVGLMFFQQFVGINGVIFYA-----QQIFVS-A--G------------------

Q3ECP7 -----VGVGLMVLQQFGGVNGIAFYA-----SSIFES-A--GV-----------------

Q9LTP6 -----VGIGLLVLQQLGGLSGYTFYL-----SSIFKK-S--GF-----------------

Q8GXK5 -----IGVVLISMPQLGGLNGYTFYT-----DTIFTS-T--GV-----------------

Q9JJZ1 -----IGICLMVFQQLSGVNAIMFYA-----NTIFEE-A--KF-----------------

P58354 -----IGISLMAFQQLSGVNAVMFYA-----ETIFEE-A--KF-----------------

Q9NY64 -----IGVSLMAFQQLSGVNAVMFYA-----ETIFEE-A--KF-----------------

Q9UGQ3 -----VALLMRLLQQLTGITPILVYL-----QSIFDSTA--VL-----------------

P43562 -----LGMTLQLLVQFSGINIILGYI-----TYICEIVGLEGN-----------------

Q9FYG3 -----IGSTLFALQQLSGINAVFYFS-----STVFKSAGVPSD-----------------

P36035 -----WLLFAYLVVLLVGPNYLTHASQDLLPTMLRAQLGL-SK-----------------

P47186 -----ITCLCWAGQATCGSILIGYS------TYFYEKAGV-ST-----------------

A6QLI1 -----ANFCRSWTFYLLLISQPAYFEEVFGFEISK-VGML--------------------

Q5W8I7 -----ANFCRSWTFYLLLISQPAYFEEVFGFEISK-VGMV--------------------

A4FV52 -----ANFCRSWTFYLLLISQPAYFEEVFGFEISK-VGLV--------------------

P34644 -----ANFARSWTFYLLLQNQLTYMKEALGMKIAD-SGLL--------------------

Q66GI9 -----ANSMHSWGFFVILSWMPIYFNSVYHVNLKQ-AAWF--------------------

Q9FKV1 -----NNFTFHYALYVLMNWLPTYFE--LGLQISL-QGMD--------------------

Q46916 -----GQYCINALTYFFITWFPVYLVQARGMSILK-AGFV--------------------

Q91Y77 -----AGIPLALFGYFVPYVHLMNHVKERFKDVNN-KEVL--------------------

Q8TF71 -----VGIPLALFGYFVPYVHLMKHVNERFQDEKN-KEVV--------------------

A1L1W9 -----FGIPAALYGYFVPYVHLMTHVEERFGPEAN-KEVL--------------------

P36021 -----FGIAAAALGYFVPYVHLMKYVEEEFSEIKE-TWVL--------------------

O35308 -----VTKFLMALGLFVPAILLVNYAKDAGVPDAE-AAFL--------------------

O95907 -----VTKFLMALGLFVPAILLVNYAKDAGVPDTD-AAFL--------------------

Q90632 -----ISKFILVLGLFVPPILLVNYPKDTGVPDTE-AAFL--------------------

O35910 -----VAASIMVLGLFVPPVFVVSYAKDMGVPDTK-AAFL--------------------

O15427 -----VAASVMVLGLFVPPVFVVSYAKDLGVPDTK-AAFL--------------------

P57788 -----LAASIMVLGLFVPPVFVVSYAKDLGYQDTK-AAFL--------------------

O15375 -----LGVMWSVLGFPLPQVFLVPYAMWHSVDEQQ-AALL--------------------

Q6ZSM3 -----VSVLFMAYGCSPLFVYLVPYALSVGVSHQQ-AAFL--------------------

Q8BGC3 -----VSVLFMAYGCSPLFVYLVPYALSVGVSHHQ-AAFL--------------------

Q503M4 -----GSFLLLASGCSLPFVYLVPYALDVGVGHQH-AAFL--------------------

O15403 -----LFGLFATLGFFAPSLYIIPLGISLGIDQDR-AAFL--------------------

Q8NCK7 -----LGTALVGGGYFVPYVHLAPHALDRGLGGYG-AALV--------------------

Q7RTY0 -----VALTLINTGYFIPYLHLVAHLQDLDWDPLP-AAFL--------------------

Q5R5M4 -----IAILLFDIGGFPPSLLMEDVARSSNVKEEEFIMPL--------------------

Q7TM99 -----IAILLFDIGGFPPSLLMEDVARSYHVREEDLTMPL--------------------

Q5ZJU0 -----FAILLFDIGGFPPSLLMEDIARSANINEEDYHMPL--------------------

O15374 -----WSFLLSQLAYFIPTFHLVARAKTLGIDIMD-ASYL--------------------

Q08777 -----LGTVFSELSINSALTYYGSYATSHGISAND-AYTL--------------------

Q08268 -----IGASFAESSLTSCATYLASYSMTRGNTENV-AYTM--------------------

P39709 -----WNIFCWNDSNVSSGAYLLWLKSLK-RYSIPKLNQL--------------------

P25621 -----WVLGGENLGFASNSTFALWLQN-Q-KYTLAQRNNY--------------------

O13880 -----MYFGIVGVGNGIFNYCTLIIEEINPSFSGIDISLL--------------------

P40445 -----QGITAFISLQTVGSYLSLILKSLN--YSTFLSNIL--------------------

P15365 -----FTVSSNIPNGGISSFMSILLNS-DFGYSSKETLLM--------------------

Q07904 -----LTACSQISTGAIGTF-SVTITG-TFGFDKYETALL--------------------

P53322 -----ALFGIDLTTFGLTVFLPIIITS--MGFTNVRAQLM--------------------

P32071 -----IALVYSIMYLIFESVPIVYAGIHHFTLVEMGATY---------------------

P38124 -----IAVAYGCFYLFFEAFPIVFVGIYHFSLVEVGLAY---------------------

Q06451 -----VCLIYSLLYAFFFAFPVIFGELYGYKDNLVGLMF---------------------

P53283 -----VCLIYSLLYAFFFAFPVIFGELYGYKDNLVGLMF---------------------

Q9C0R8 -----MAICYGLLYMFFFAYPVVYQQGKGWSASLTGVMF---------------------

Q9C0Q6 -----MSVLYGLLYMFFFAYPIVYQEGKGWSASKTGVMF---------------------

Q9HF77 -----NAFIYGMLYLFLTAIPLIFLGEYHFVQGVAELPY---------------------

Q5A0E9 -----NGFIYAMLYCLLTAMPLIFQEGYGFRRGLAELPY---------------------

Q07824 -----NSFVYGILYLLLEAYPLVFVEGYGFTEN-GELPY---------------------

O59698 -----CSFVYAIIYLLLEAYPVIFQEGRHFPLGVSALPY---------------------

O74829 -----TAFVYGILYGLLEAYPVIFGESRKWRLGVESLPY---------------------

P38776 -----TGLELAIIYLYFVAFPYVFKKLYNFGPMEIACSY---------------------

Q9HDX4 -----VGTVYGILYLLFTAFAEVWISQYHFTSGLSGLTY---------------------

P38227 NGGEIAPKQVSAVRKVWDTFFVYFIKPLKSLHFLEYPPVA--------------------

P38125 -----PPK--AGLTLYW--------KMIKC------PPII--------------------

P40474 -----LDNPDYETLELPTQ--LNLLAPFKI---LKAYEIC--------------------

O94607 -----ITLSIALLVTLCFYDVKYARYPVFALKSLK-DRTI--------------------

Q9HE13 -----LIFGILCIAGFVVNELYTTRTRIIAPSAFQ-TLSL--------------------

Q04301 -----ALFTIGSFIVFILVERYYATEKIL-PFELL-TRSF--------------------

P13090 -----LIISVIFLVVFIIYEIRFAKTPLLPRAVIK-DRHM--------------------

Q08902 -----LIVGTFFLVIFAYIESR-AAFPLLPFAALS-SDTA--------------------

P39886 -----LAAAVLAFAGFTLRESR-ATEPLMPLAMFRSVPLS--------------------

P76269 -----LVVMVVVGIFFIRRQLS-LPVPLLPVDLLR-IPLF--------------------

Q8Y9K8 -----IIVGGIALGLFVWRQLT-MEKPLMDLKVFK-YPMF--------------------

P28873 ------------------------------------------------------------

A4WFG6 ------------------------------------------------------------

A1JSB0 ------------------------------------------------------------

A8GKP6 ------------------------------------------------------------

P57601 ------------------------------------------------------------

Q8K942 ------------------------------------------------------------

Q89A60 ------------------------------------------------------------

P76198 ------------------------------------------------------------

O05390 ------------------------------------------------------------

O34864 ------------------------------------------------------------

O52717 ------------------------------------------------------------

O52718 ------------------------------------------------------------

A9MJT5 ------------------------------------------------------------

A6TG19 ------------------------------------------------------------

A0L190 ------------------------------------------------------------

P0AEY8 ------------------------------------------------------------

Q7CP73 ------------------------------------------------------------

P39386 ------------------------------------------------------------

Q68WD6 ------------------------------------------------------------

Q4UMJ9 ------------------------------------------------------------

Q1RI77 ------------------------------------------------------------

P32482 ------------------------------------------------------------

P37597 ------------------------------------------------------------

P45123 ------------------------------------------------------------

P28246 ------------------------------------------------------------

P31442 ------------------------------------------------------------

P37482 ------------------------------------------------------------

P76242 ------------------------------------------------------------

P17583 ------------------------------------------------------------

P0C105 ------------------------------------------------------------

O25788 ------------------------------------------------------------

P11551 ------------------------------------------------------------

P44776 ------------------------------------------------------------

A1A9E1 ------------------------------------------------------------

A9MHY5 ------------------------------------------------------------

A4W8S1 ------------------------------------------------------------

A1JMG4 ------------------------------------------------------------

Q0TK80 ------------------------------------------------------------

O06473 ------------------------------------------------------------

Q2FI61 ------------------------------------------------------------

Q5HQE8 ------------------------------------------------------------

Q49WE5 ------------------------------------------------------------

Q4L523 ------------------------------------------------------------

P33026 ------------------------------------------------------------

P31436 ------------------------------------------------------------

Q9S3K0 ------------------------------------------------------------

P31675 ------------------------------------------------------------

P31126 ------------------------------------------------------------

Q58955 ------------------------------------------------------------

P02920 ------------------------------------------------------------

Q4UK37 ------------------------------------------------------------

Q1RKF6 ------------------------------------------------------------

Q4ULW4 ------------------------------------------------------------

Q1RI01 ------------------------------------------------------------

Q68WQ5 ------------------------------------------------------------

Q4UL88 ------------------------------------------------------------

Q92HQ3 ------------------------------------------------------------

Q1RIL0 ------------------------------------------------------------

Q4UMU2 ------------------------------------------------------------

Q68W71 ------------------------------------------------------------

Q1RHK8 ------------------------------------------------------------

Q1LTM2 ------------------------------------------------------------

Q2NTK5 ------------------------------------------------------------

P55705 ------------------------------------------------------------

Q7Z3Q1 ------------------------------------------------------------

Q05B81 ------------------------------------------------------------

P76470 ------------------------------------------------------------

P32135 ------------------------------------------------------------

P38358 ------------------------------------------------------------

A4WAE6 ------------------------------------------------------------

A6T8Y8 ------------------------------------------------------------

Q6CZ44 ------------------------------------------------------------

Q888L8 ------------------------------------------------------------

Q1IB51 ------------------------------------------------------------

A6UZY0 ------------------------------------------------------------

Q4QP52 ------------------------------------------------------------

Q9CM87 ------------------------------------------------------------

O25797 ------------------------------------------------------------

Q17YP7 ------------------------------------------------------------

P77389 ------------------------------------------------------------

O31577 ------------------------------------------------------------

O34367 ------------------------------------------------------------

A1AHK2 ------------------------------------------------------------

A9MWE8 ------------------------------------------------------------

P23910 ------------------------------------------------------------

P31141 ------------------------------------------------------------

P43531 ------------------------------------------------------------

Q8K902 ------------------------------------------------------------

P57648 ------------------------------------------------------------

Q89A23 ------------------------------------------------------------

P37498 ------------------------------------------------------------

P0A0J4 ------------------------------------------------------------

Q07282 ------------------------------------------------------------

P02982 ------------------------------------------------------------

P02981 ------------------------------------------------------------

P70187 ------------------------------------------------------------

Q5SR56 ------------------------------------------------------------

P77726 ------------------------------------------------------------

Q89AA9 ------------------------------------------------------------

Q8K999 ------------------------------------------------------------

P57538 ------------------------------------------------------------

Q5HIA2 ------------------------------------------------------------

Q4L3Q4 ------------------------------------------------------------

Q5HRH0 ------------------------------------------------------------

P0C0L7 ------------------------------------------------------------

Q47421 ------------------------------------------------------------

P0A2G3 ------------------------------------------------------------

P16482 ------------------------------------------------------------

P0AEX3 ------------------------------------------------------------

P76350 ------------------------------------------------------------

P41036 ------------------------------------------------------------

Q9SYQ1 ------------------------------------------------------------

Q9S735 ------------------------------------------------------------

P76230 ------------------------------------------------------------

P38055 ------------------------------------------------------------

Q46909 ------------------------------------------------------------

P31679 ------------------------------------------------------------

O24723 ------------------------------------------------------------

O30513 ------------------------------------------------------------

Q43975 ------------------------------------------------------------

Q51955 ------------------------------------------------------------

Q9I6Q3 ------------------------------------------------------------

P77589 ------------------------------------------------------------

P94131 ------------------------------------------------------------

P71369 ------------------------------------------------------------

O34691 ------------------------------------------------------------

P0AGC0 ------------------------------------------------------------

P27669 ------------------------------------------------------------

P96335 ------------------------------------------------------------

P08194 ------------------------------------------------------------

P37948 ------------------------------------------------------------

P12681 ------------------------------------------------------------

Q5M7K3 ------------------------------------------------------------

Q9WU81 ------------------------------------------------------------

Q58CV5 ------------------------------------------------------------

Q7SY29 ------------------------------------------------------------

P57057 ------------------------------------------------------------

Q17QZ3 ------------------------------------------------------------

Q3TIT8 ------------------------------------------------------------

Q640L2 ------------------------------------------------------------

Q5F3N0 ------------------------------------------------------------

Q09037 ------------------------------------------------------------

Q06222 ------------------------------------------------------------

P13865 ------------------------------------------------------------

P46499 ------------------------------------------------------------

P54219 ------------------------------------------------------------

Q6NT16 ------------------------------------------------------------

Q8R0G7 ------------------------------------------------------------

Q5XGK0 ------------------------------------------------------------

Q7ZU13 ------------------------------------------------------------

A2CER7 ------------------------------------------------------------

Q9D232 ------------------------------------------------------------

Q6ZMD2 ------------------------------------------------------------

Q6GPQ3 ------------------------------------------------------------

A5IVG9 ------------------------------------------------------------

Q5HLK7 ------------------------------------------------------------

P46907 ------------------------------------------------------------

P10903 ------------------------------------------------------------

P37593 ------------------------------------------------------------

P37758 ------------------------------------------------------------

Q9P3K8 ------------------------------------------------------------

Q6FWD4 ------------------------------------------------------------

Q6CPY8 ------------------------------------------------------------

P22152 ------------------------------------------------------------

Q02563 NFTLENQIHRGGQYFNDKFIGLRLKSVSFEDSLFEECYFEDVTSSNTFFRNCTFINTVFY

Q496J9 NFTMENQIHTGMEYDNGRFIGVKFKSVTFKDSVFKSCTFEDVTSVNTYFKNCTFIDTVFD

Q63564 NFTMENQIHQHGKLVNDKFIKMYFKHVLFEDTFFDKCYFEDVTSTDTYFKNCTIESTTFY

Q1JP63 --SISSRK----------------KAVEAKCSLA--------------------------

Q2XWK0 --SISNQR----------------KAVKPKCSLA--------------------------

Q1LVS8 DPDLEHQI----------------KHIQEETLCY--------------------------

O08966 ------------------------------------------------------------

O15245 ------------------------------------------------------------

O77504 ------------------------------------------------------------

A7MBE0 ------------------------------------------------------------

Q9R0W2 ------------------------------------------------------------

O02713 ------------------------------------------------------------

Q8MJI6 ------------------------------------------------------------

O88446 ------------------------------------------------------------

O75751 ------------------------------------------------------------

Q9U539 ------------------------------------------------------------

O76082 ------------------------------------------------------------

Q497L8 ------------------------------------------------------------

Q86VW1 ------------------------------------------------------------

Q17QN9 ------------------------------------------------------------

Q95R48 ------------------------------------------------------------

Q9Y267 ------------------------------------------------------------

Q6A4L0 ------------------------------------------------------------

Q9Y226 ------------------------------------------------------------

Q8IVM8 ------------------------------------------------------------

Q66J52 ------------------------------------------------------------

Q91WU2 ------------------------------------------------------------

Q8IZD6 ------------------------------------------------------------

Q6DFR1 ------------------------------------------------------------

Q6NUB3 ------------------------------------------------------------

Q28ES4 ------------------------------------------------------------

A6NKX4 ------------------------------------------------------------

P47185 ------------------------------------------------------------

P13181 ------------------------------------------------------------

P23585 ------------------------------------------------------------

Q92339 ------------------------------------------------------------

O74969 ------------------------------------------------------------

P10870 ------------------------------------------------------------

Q12300 ------------------------------------------------------------

P42833 ------------------------------------------------------------

P49374 ------------------------------------------------------------

O74713 ------------------------------------------------------------

Q9BE72 ------------------------------------------------------------

Q5J316 ------------------------------------------------------------

Q32NG5 ------------------------------------------------------------

Q6NWF1 ------------------------------------------------------------

Q0P4G6 ------------------------------------------------------------

Q6GN01 ------------------------------------------------------------

O95528 ------------------------------------------------------------

Q8VHD6 ------------------------------------------------------------

Q3UHK1 ------------------------------------------------------------

Q96QE2 ------------------------------------------------------------

Q9C757 ------------------------------------------------------------

Q8VZR6 ------------------------------------------------------------

P30606 ------------------------------------------------------------

P30605 ------------------------------------------------------------

Q10286 ------------------------------------------------------------

Q04162 ------------------------------------------------------------

Q01440 ------------------------------------------------------------

P11166 ------------------------------------------------------------

P46896 ------------------------------------------------------------

P47843 ------------------------------------------------------------

P14672 ------------------------------------------------------------

Q90592 ------------------------------------------------------------

P11168 ------------------------------------------------------------

Q5RB09 ------------------------------------------------------------

A4ZYQ5 ------------------------------------------------------------

P22732 ------------------------------------------------------------

P58353 ------------------------------------------------------------

Q863Y9 ------------------------------------------------------------

P43427 ------------------------------------------------------------

Q9WV38 ------------------------------------------------------------

P15686 ------------------------------------------------------------

Q39525 ------------------------------------------------------------

Q94AZ2 ------------------------------------------------------------

Q10710 ------------------------------------------------------------

Q41144 ------------------------------------------------------------

P23586 ------------------------------------------------------------

O65413 ------------------------------------------------------------

Q9SX48 ------------------------------------------------------------

Q9LT15 ------------------------------------------------------------

Q9FMX3 ------------------------------------------------------------

Q9SBA7 ------------------------------------------------------------

Q9SFG0 ------------------------------------------------------------

Q8L7R8 ------------------------------------------------------------

Q93Y91 ------------------------------------------------------------

P0AE24 ------------------------------------------------------------

P0AEP1 ------------------------------------------------------------

P96710 ------------------------------------------------------------

P54723 ------------------------------------------------------------

O34718 ------------------------------------------------------------

P46333 ------------------------------------------------------------

O52733 ------------------------------------------------------------

P0AGF4 ------------------------------------------------------------

P21906 ------------------------------------------------------------

P15729 ------------------------------------------------------------

Q6AWX0 ------------------------------------------------------------

Q93YP9 ------------------------------------------------------------

Q94AF9 ------------------------------------------------------------

O04036 ------------------------------------------------------------

Q94KE0 ------------------------------------------------------------

Q9SCW7 ------------------------------------------------------------

Q4F7G0 ------------------------------------------------------------

Q8LBI9 ------------------------------------------------------------

P93051 ------------------------------------------------------------

Q0WQ63 ------------------------------------------------------------

Q3ECP7 ------------------------------------------------------------

Q9LTP6 ------------------------------------------------------------

Q8GXK5 ------------------------------------------------------------

Q9JJZ1 ------------------------------------------------------------

P58354 ------------------------------------------------------------

Q9NY64 ------------------------------------------------------------

Q9UGQ3 ------------------------------------------------------------

P43562 ------------------------------------------------------------

Q9FYG3 ------------------------------------------------------------

P36035 ------------------------------------------------------------

P47186 ------------------------------------------------------------

A6QLI1 ------------------------------------------------------------

Q5W8I7 ------------------------------------------------------------

A4FV52 ------------------------------------------------------------

P34644 ------------------------------------------------------------

Q66GI9 ------------------------------------------------------------

Q9FKV1 ------------------------------------------------------------

Q46916 ------------------------------------------------------------

Q91Y77 ------------------------------------------------------------

Q8TF71 ------------------------------------------------------------

A1L1W9 ------------------------------------------------------------

P36021 ------------------------------------------------------------

O35308 ------------------------------------------------------------

O95907 ------------------------------------------------------------

Q90632 ------------------------------------------------------------

O35910 ------------------------------------------------------------

O15427 ------------------------------------------------------------

P57788 ------------------------------------------------------------

O15375 ------------------------------------------------------------

Q6ZSM3 ------------------------------------------------------------

Q8BGC3 ------------------------------------------------------------

Q503M4 ------------------------------------------------------------

O15403 ------------------------------------------------------------

Q8NCK7 ------------------------------------------------------------

Q7RTY0 ------------------------------------------------------------

Q5R5M4 ------------------------------------------------------------

Q7TM99 ------------------------------------------------------------

Q5ZJU0 ------------------------------------------------------------

O15374 ------------------------------------------------------------

Q08777 ------------------------------------------------------------

Q08268 ------------------------------------------------------------

P39709 ------------------------------------------------------------

P25621 ------------------------------------------------------------

O13880 ------------------------------------------------------------

P40445 ------------------------------------------------------------

P15365 ------------------------------------------------------------

Q07904 ------------------------------------------------------------

P53322 ------------------------------------------------------------

P32071 ------------------------------------------------------------

P38124 ------------------------------------------------------------

Q06451 ------------------------------------------------------------

P53283 ------------------------------------------------------------

Q9C0R8 ------------------------------------------------------------

Q9C0Q6 ------------------------------------------------------------

Q9HF77 ------------------------------------------------------------

Q5A0E9 ------------------------------------------------------------

Q07824 ------------------------------------------------------------

O59698 ------------------------------------------------------------

O74829 ------------------------------------------------------------

P38776 ------------------------------------------------------------

Q9HDX4 ------------------------------------------------------------

P38227 ------------------------------------------------------------

P38125 ------------------------------------------------------------

P40474 ------------------------------------------------------------

O94607 ------------------------------------------------------------

Q9HE13 ------------------------------------------------------------

Q04301 ------------------------------------------------------------

P13090 ------------------------------------------------------------

Q08902 ------------------------------------------------------------

P39886 ------------------------------------------------------------

P76269 ------------------------------------------------------------

Q8Y9K8 ------------------------------------------------------------

P28873 -------------------------------------MSIVIGIVIAAFIYIPVIR---Q

A4WFG6 ------------------------------------LNDAGKLVSDFWMSYMFGMWAFSF

A1JSB0 ------------------------------------ISQAGQLVSNFWISYMIGMWVFSF

A8GKP6 ------------------------------------ISQAGKLVSDFWTSYMIGMWVFSF

P57601 ------------------------------------IKKTGSLVSGFWMSYMLGMWFFSF

Q8K942 ------------------------------------IKKTGVLVSNFWMAYMIGMWCFSF

Q89A60 ------------------------------------INQSSKLVSAFWMAYMVGMWIFSF

P76198 ------------------------------------EAEALKTISYYSMGSLVCVFIFAA

O05390 ------------------------------------VSEWLQIWGTLFFVNIVFNIIFGA

O34864 ------------------------------------TNVWLQIWGTIFLGNIVFNLIFGI

O52717 ------------------------------------TSEWLQVWAAFFFTTIFSNVFWGI

O52718 ------------------------------------TSEWLQVWAVFFFTTIFSNVLWGI

A9MJT5 ------------------------------------RGDYAITMALTAGVSMVVSFSTPF

A6TG19 ------------------------------------RGTYSMAMALMAMISMAVSFSTPF

A0L190 ------------------------------------RGQYSNTMAMTALVSMLASFSTPF

P0AEY8 ------------------------------------SYEYGLLQVPIFGALIAGNLLLAR

Q7CP73 ------------------------------------TSQFAWAQVPVFGAVIVANMIVVR

P39386 ------------------------------------TSQFAWTQVPVFGAVIVANAIVAR

Q68WD6 ------------------------------------PSFYGKLAFLLSFSAIFGGFLGGY

Q4UMJ9 ------------------------------------SSFYGKLAFLLSFAAIFGGFLGGY

Q1RI77 ------------------------------------PSFYGKLAFLLSFAGIFGGFLGGY

P32482 ------------------------------------QLGFSLLFATVAIAMVFTARFMGR

P37597 ------------------------------------PAVIGLSYVPQTIAFLIGGYGCRA

P45123 ------------------------------------VDQFGYFFMMNIVTMIFASFLNSR

P28246 ------------------------------------PENFGYYFALNIVFLFVMTIFNSR

P31442 ------------------------------------SMTVSILFILPIPAAFFGAWFAGR

P37482 ------------------------------------TATAGWMVSLMQFASLPSTFLTPV

P76242 ------------------------------------EAQAGSLHGLLQLATAAPGLLIPL

P17583 ------------------------------------AQYSGSLLALMTLGQAAGALLMPA

P0C105 ------------------------------------ETDAAHHVAYFWGGAMVGRFIGSA

O25788 ------------------------------------SQSSAHYLVYYWGGAMVGRFLGSV

P11551 ------------------------------------AGFAANYLTGTMVCFFIGRFTGTW

P44776 ------------------------------------KAEGQNFNIIAMAIFISSRFISTA

A1A9E1 ------------------------------------NASIGFWMAVLVSAGILGQWPIGR

A9MHY5 ------------------------------------NASIGFWMAVLVSAGILGQWPVGR

A4W8S1 ------------------------------------DSGIGFWMAVMVSAGIVGQWPIGK

A1JMG4 ------------------------------------DASVGWWMALLVSSGIIGQWPIGK

Q0TK80 ------------------------------------AAQIGFLYAAIPLGAAIGALTSGK

O06473 ------------------------------------YVFFGWG-LLLAITSVFTAPLVHK

Q2FI61 ------------------------------------EYTVAIIIG-GIGCAVSMLFLSKL

Q5HQE8 ------------------------------------EYTVAIIIG-GIGCAFSMLFLSKI

Q49WE5 ------------------------------------EYTMAIVFG-GIGCAISMLFLSKI

Q4L523 ------------------------------------EYTIAIAIG-GAGCAFSMLFLSKI

P33026 ------------------------------------EKLAGVMMGTAAGLEIPTMLIAGY

P31436 ------------------------------------DKLTGEMIGIAAGLEIPMMLIAGY

Q9S3K0 ------------------------------------EKLAGVMMGTAAGLEIPVMLLAGY

P31675 ------------------------------------DKLAGFLMGTAAGLEIPAMILAGY

P31126 ------------------------------------EKVVAVVLPVNAAMVVTLQYSVGR

Q58955 ------------------------------------NITISQVGFMIALTNILMALLQRS

P02920 ------------------------------------EQGTRVFGYVTTMGELLNASIMFF

Q4UK37 ------------------------------------HEIAVIYKAYGLLIMIVGGALGGV

Q1RKF6 ------------------------------------HEIAFIYKAYGLFIMIVGGALGGI

Q4ULW4 ------------------------------------GEIAIIAKLYGLIATLVGGFAGGI

Q1RI01 ------------------------------------SEIAVIVKLYGLIATLVGGFVGGI

Q68WQ5 ------------------------------------FEIASVCKFCGVIGAIIGGLIGGI

Q4UL88 ------------------------------------FEIASVGKFWGVVGAIIGGLVGGF

Q92HQ3 ------------------------------------FEIASVGKFCGVVGAIIGGLVGGV

Q1RIL0 ------------------------------------FEIASVGKFWGVMGAIVGGLLGGF

Q4UMU2 ------------------------------------KDLALGYKAFGMCAAILGGFIGGF

Q68W71 ------------------------------------QDLAIGYKAFGMCAAIFGGVIGGF

Q1RHK8 ------------------------------------KDLALGYKAFGMCATIAGGFIGGF

Q1LTM2 ------------------------------------LAAVRWTYAIEAALSLSLLYPIAR

Q2NTK5 ------------------------------------PAAVKWMYAIEAALSLSLLYPIAR

P55705 ------------------------------------HWITGLSYGL-LALGFVVAAPV--

Q7Z3Q1 ------------------------------------IGYGSALGSASFLTSFLGIWLF--

Q05B81 ------------------------------------ISYGSAAQQLPYLTSLLGLRLL--

P76470 ------------------------------------GFTASVVTAIPWVAALFGTWLIPR

P32135 ------------------------------------ALVGAYGIINQYCLKMIGGPIGGM

P38358 ------------------------------------TAKAGLRLTIPSLFTPVGSLITGF

A4WAE6 ---------------------------------------ATVLLLILGGAGIIGSVLFGK

A6T8Y8 ---------------------------------------ATVLLLILGGAGIIGSILFGK

Q6CZ44 ---------------------------------------TTILLLIFGGAGIIGSMLFSR

Q888L8 ---------------------------------------TTILLLVFGGAGIMGSIVFSL

Q1IB51 ---------------------------------------TTLLLLLFGGAGVFGSLLFSR

A6UZY0 ---------------------------------------TTLLLLLFGGAGIFGSLLFSR

Q4QP52 ---------------------------------------ATAVLLVFGFSGIAASLLFNR

Q9CM87 ---------------------------------------ATFVLFVFGLSGITASLLFNR

O25797 ---------------------------------------TTLMLFVFGLAGVVGSFLFGR

Q17YP7 ---------------------------------------ATLMLFVFGLAGVMGSFLFGR

P77389 ---------------------------------------VTAMLVLIGVGFSIGNYLGGK

O31577 ---------------------------------------ITGALFAYGLGGVAGNFFAGK

O34367 ---------------------------------------VAVILLGYGIAIAIGNMIGGK

A1AHK2 ---------------------------------------LTLVLLSFGIASFVGTSLSSF

A9MWE8 ---------------------------------------LTLVLLSFGIASFVGTSFSSY

P23910 ---------------------------------------MTFIMMLVGLGMVLGNMLSGR

P31141 ---------------------------------------ISVALVLFGAGSFAGVTVAGR

P43531 ---------------------------------------VVGLLSLAYLTGTWSSPKAGT

Q8K902 ---------------------------------------NIGFLSIIYLTGVYSSPKAGI

P57648 ---------------------------------------SIGLLSTIYLTGVYSSPKAGV

Q89A23 ---------------------------------------TIGLLSIIYLIGVYSSPQAGV

P37498 ---------------------------------------FSSWIFIVMIVGIFSSSFIGR

P0A0J4 ---------------------------------------ISIAITGGGIFGALFQIYFFD

Q07282 ---------------------------------------VGVSLAVFGLTHALFQGLAAG

P02982 ---------------------------------------IGISLAAFGILHSLAQAMITG

P02981 ---------------------------------------IGLSLAVFGILHALAQAFVTG

P70187 ---------------------------------------VAAFIAVLGILSIIAQTIVLS

Q5SR56 ---------------------------------------IAAFIAMVGILSIVAQTAFLS

P77726 ---------------------------------------WKVYLATMLIAFGSVVPFIIY

Q89AA9 ---------------------------------------EIIYIVILLVSFLIVLFCICF

Q8K999 ---------------------------------------WIVYFATIVFSFFFLFLIVFY

P57538 ---------------------------------------WKVYLGTILISFFVLFLFIFY

Q5HIA2 ---------------------------------------TSVLITCVMAIMIPLALMFGK

Q4L3Q4 ---------------------------------------TSVLITCVMAVMIPLALFFGK

Q5HRH0 ---------------------------------------TSIMITCIMVIMIPLALMFGR

P0C0L7 ---------------------------------------GVLIIIAIMIGMLFVQPVMGL

Q47421 ---------------------------------------GVLIIIAIMIGMLFVQPVMGL

P0A2G3 ---------------------------------------SLIVTMLVGVSNFIWLPIGGA

P16482 ---------------------------------------SLLVTLLVAISNFFWLPVGGA

P0AEX3 ---------------------------------------ASGIMTAALFVFMLIQPLIGA

P76350 ---------------------------------------FLNIGLLVGGLSCLTIPCFAW

P41036 ---------------------------------------GVMLMVVVLFAFLYSWPIQAL

Q9SYQ1 ------------------------------------FEVAELGAIIAACSTIPGYWFTVY

Q9S735 ------------------------------------FEVAKLAAIVAACSTIPGYWFTVY

P76230 ---------------------------------------SIVLNTMSMFGAPFGIFIAML

P38055 ---------------------------------------SILMTAVIMIGAPVGIFIAAL

Q46909 ---------------------------------------ALTASLMLNALLIVGALLGLV

P31679 ---------------------------------------AALGNVVISLFFMLGCIPPML

O24723 ---------------------------------------SGLIVAMYGWGALIGQLTIAF

O30513 ---------------------------------------SLIFLFSLNVGGMIGSILGGY

Q43975 ---------------------------------------AAFLGGLFQFGGVLSALFIGW

Q51955 ---------------------------------------AAFIGALFQFGGVLSAVGVGW

Q9I6Q3 ---------------------------------------AAFIGALFQFGGVLSAVAVGW

P77589 ---------------------------------------AAGVMFALQMGAASGTLMLGA

P94131 ---------------------------------------MTAYMVGTYTAMILGKILAGF

P71369 ---------------------------------------SGLWTAVTVCGMMAGIWIFGQ

O34691 ---------------------------------------SFEYVLLMTLAQLPGYFSAAW

P0AGC0 -------------------------------------AVAIQGFTLFEAGALVGTLLWGW

P27669 -------------------------------------VTANTAVSMFELGGFIGALVAGW

P96335 -------------------------------------KGTAWAYTIYELAAVPGTLLCGW

P08194 -------------------------------------DKSSWAYFLYEYAGIPGTLLCGW

P37948 -------------------------------------EDSRWSYFLYEYAGIPGTILCGW

P12681 -------------------------------------EQMSVAFLFFEWAAIPSTLLAGW

Q5M7K3 -------------------------------------KAAGDLSTLFDVGGIIGGILAGG

Q9WU81 -------------------------------------KEAGDLSTLFDVGGIIGGIMAGL

Q58CV5 -------------------------------------KEAGDLSTLFDVGGIIGGILAGL

Q7SY29 -------------------------------------KKAGDLSTLFDVGGILGGIVAGL

P57057 -------------------------------------KKAGELSTLFDVGGIFGGILAGV

Q17QZ3 -------------------------------------AEADQLSIWYDVGGIIGGTLQGF

Q3TIT8 -------------------------------------AEADKLSIWYDVGGIIGGTLLGF

Q640L2 -------------------------------------AEADQLSIWYDIGGIVGGTVQGL

Q5F3N0 -------------------------------------AEADQLSIWYDVGGIIGGTIQGL

Q09037 -----------------------------------D---PSLGNFLVMAWNFVTSLVAIP

Q06222 -----------------------------------D---PSLGNFLVMAWNFVTSLVAIP

P13865 -----------------------------------A---PLVGNFVVMLWNFVTTLASIP

P46499 -----------------------------------NFYLSQFVQAAAIALSKLSIFLLDL

P54219 -----------------------------------PKWQLGLAFLPASVSYLIGTNLFGV

Q6NT16 -----------------------------------PAGYVGLVFLGMALSYAISSPLFGL

Q8R0G7 ------------------------------PGDSCSSSDSLIFGLITCLTGVLGVGLGVE

Q5XGK0 ------------------------------EGGICNYDDSMIFGGITCITGILGVLTGVE

Q7ZU13 ------------------------------KAP-CDDSDSLIFGAITVVTGILGVASGVQ

A2CER7 ------------------------------EEP-CDSVDSYIFGAITVVTGVVGVFLGTC

Q9D232 ------------------------------QEQ-CHSQDSLIFGALTVATGIIGVMLGAE

Q6ZMD2 ------------------------------QEP-CSNPDSLIFGALTIMTGVIGVILGAE

Q6GPQ3 ---------------------------------------VFYNGIILAAVGVESVIVFLT

A5IVG9 ---------------------------------------G---VFIA--LATFLRPIGGI

Q5HLK7 ---------------------------------------G---IFIA--LATFLRPVGGV

P46907 ---------------------------------------A---GFIA--VSTLLRPAGGF

P10903 ---------------------------------------Y---AFFGPFIGALARSAGGA

P37593 ---------------------------------------L---AFFGPFIGALARSAGGV

P37758 ---------------------------------------L---AFFGPFIGAIARSVGGA

Q9P3K8 ---------------------------------------T--HVSIVGITSTLVRLLTGS

Q6FWD4 ---------------------------------------A--LLSIYALTSTLTRLGTGL

Q6CPY8 ---------------------------------------N--VLTQFALFSTSSRLLSGL

P22152 ---------------------------------------TGKWAAMFGFLNIVCRPAGGF

Q02563 NTDLFEYKFVNSRLVNSTFLHNKEGCPLDVTGTGEGAYMVYFVSFLGTLAVLPGNIVSA-

Q496J9 NTDFEPYKFIDSEFKNCSFFHNKTGCQI-TFDDDYSAYWIYFVNFLGTLAVLPGNIVSA-

Q63564 NTDLYKHKFIDCRFINSTFLEQKEGCHMDFEEDND--FLIYLVSFLGSLSVLPGNIISA-

Q1JP63 -------------------------CEYLSEED-------YMDLLWTTLSEFPGVLVTL-

Q2XWK0 -------------------------CEYLTVED-------YTDLLWTTLSEFPGLLVTL-

Q1LVS8 -------------------------CIPFNSDD-------YQTLLISCLGEVALIPLNI-

O08966 -------------------------------------ANLYLDFFYSSLVEFPAAFIIL-

O15245 -------------------------------------GNLYLDFLYSALVEIPGAFIAL-

O77504 -------------------------------------GNVYLDFFYSSLVEFPAAFVIL-

A7MBE0 -------------------------------------GNLYLDFLYSALVEFPAGFIIL-

Q9R0W2 -------------------------------------DNIYLDFFYSALVEFPAAFIII-

O02713 -------------------------------------SNLYLDFFYSALVEFPAALLIL-

Q8MJI6 -------------------------------------GDIYLDFFYSALVEFPAAFLII-

O88446 -------------------------------------GNLYMDFFISGLVELPGALLIL-

O75751 -------------------------------------GNLYIDFFISGVVELPGALLIL-

Q9U539 -------------------------------------VDDYWSFIINGAVELPGYFVVW-

O76082 -------------------------------------GDIFVNCFLSAMVEVPAYVLAW-

Q497L8 -------------------------------------ENEPLYLLLVGAMEIPAYICLC-

Q86VW1 -------------------------------------GNEYLNLFLLGVVEIPAYTFVC-

Q17QN9 -------------------------------------GSEYLNLFLMGVVEIPAYVLVC-

Q95R48 -------------------------------------GNVLLNFVISGAVEIPAYIFLL-

Q9Y267 -------------------------------------VSVHFRHVVPSIMEVPARLCCI-

Q6A4L0 -------------------------------------LDIYVTQLIFGAVEMPGRFLSV-

Q9Y226 -------------------------------------LDVYLTQLIFGAVEVPARCSSI-

Q8IVM8 -------------------------------------NNVFLLQTLFGAVILLANCVAP-

Q66J52 -------------------------------------VSIYIIQIIFGTVDIPAKFISY-

Q91WU2 -------------------------------------LTVYQTQLLFGAVEVPSKITVF-

Q8IZD6 -------------------------------------GSIYANLALSGLIEIPSYPLCI-

Q6DFR1 -------------------------------------GDIYLNLALSGLAELPAYPLCM-

Q6NUB3 -------------------------------------GDIYLNLALSGLAELPAYPLCM-

Q28ES4 -------------------------------------GNLYLNVALYGLVEVPAFPLCL-

A6NKX4 -------------------------------------PTFYLPYFLEAGLEAAALVFLL-

P47185 -------------------------------------D-GFETSIVLGTVNFFSTIIAV-

P13181 -------------------------------------D-SFETSIVIGVVNFASTFFSL-

P23585 -------------------------------------D-SFQTSIVLGIVNFASTFVAL-

Q92339 -------------------------------------D-IFLPAVILGAINFGTTFGAL-

O74969 -------------------------------------S-PYLSAMILDAVNFGCTFGGM-

P10870 -------------------------------------N-SYLVSFITYAVNVVFNVPGL-

Q12300 -------------------------------------N-SYLVSFISYAVNVAFSIPGM-

P42833 -------------------------------------D-PYITSIILSSVNFLSTILGI-

P49374 -------------------------------------T-NLVASSIQYVLNVVMTIPAL-

O74713 -------------------------------------S-NLVASSIQYVINTCVTVPAL-

Q9BE72 -------------------------------------EAASLASTGVGVVKVISTIPAT-

Q5J316 -------------------------------------EAASLASTGVGVVKVISTIPAT-

Q32NG5 -------------------------------------EAASLASTGIGVVKVVSTIPAI-

Q6NWF1 -------------------------------------EAATLASTGFGVVKVGGTIPAI-

Q0P4G6 -------------------------------------SSAVLASVGLGVVKVASTLIAI-

Q6GN01 -------------------------------------SSAVLASVGLGVVKVASTLIAI-

O95528 -------------------------------------SSAVLASVGLGAVKVAATLTAM-

Q8VHD6 -------------------------------------SSAVLASVGLGTVKVAATLVAT-

Q3UHK1 -------------------------------------RLAIWLASITAFTNFIFTLVGV-

Q96QE2 -------------------------------------RLAIWLASVTAFTNFIFTLVGV-

Q9C757 -------------------------------------RTALLLSLVTAGLNAFGSIISI-

Q8VZR6 -------------------------------------QLALFLSLIVAAMNAAGTVVGI-

P30606 ---------------------------------------SSAVSIIVSGTNFVFTLIAF-

P30605 ---------------------------------------SSAVSIIVSGTNFIFTLVAF-

Q10286 ---------------------------------------PTATGLIIAATNFVFTIVAF-

Q04162 ---------------------------------------PLLPPILIASTNFIFTFFAM-

Q01440 -------------------------------------IMPVVLSIPLAFMNALFTAVAI-

P11166 -------------------------------------Q-PVYATIGSGIVNTAFTVVSL-

P46896 -------------------------------------Q-PVYATIGSGVVNTAFTVVSL-

P47843 -------------------------------------E-PVYATIGAGVVNTIFTVVSV-

P14672 -------------------------------------Q-PAYATIGAGVVNTVFTLVSV-

Q90592 -------------------------------------Q-PVYATIGVGVVNTVFTVISV-

P11168 -------------------------------------K-PVYATIGVGAVNMVFTAVSV-

Q5RB09 -------------------------------------K-IPYVTLSTGGIETLAAIFSG-

A4ZYQ5 -------------------------------------H-SQYVTVGSGVVNIVMTITSA-

P22732 -------------------------------------H-VQYVTAGTGAVNVVMTFCAV-

P58353 -------------------------------------D-VQYVTAGTGAVNVLITVCAI-

Q863Y9 -------------------------------------D-VQYVTVGTGAVNVLMTICAV-

P43427 -------------------------------------D-VQYVTAGTGAVNVFMTMVTV-

Q9WV38 -------------------------------------D-VQYVTAGTGAVNVFMTILTI-

P15686 -------------------------------------A-ALLNTVVVGAVNVGSTLIAV-

Q39525 -------------------------------------A-ALLNTVVVGAVNVGSTMIAV-

Q94AZ2 -------------------------------------A-SLYSAVVTGAVNVLSTLVSI-

Q10710 -------------------------------------A-ALYSSAVTGAVLCSSTFISI-

Q41144 -------------------------------------A-ALMSAVITGLVNVFATMVSI-

P23586 -------------------------------------A-SLMSAVVTGSVNVAATLVSI-

O65413 -------------------------------------A-ALISAVVTGLVNVGATVVSI-

Q9SX48 -------------------------------------A-SLISAVITGAVNVVSTLVSI-

Q9LT15 -------------------------------------A-ALMSAVITGVVNMLSTFVSI-

Q9FMX3 -------------------------------------A-SLISAVITGLVNVLSTIVSI-

Q9SBA7 -------------------------------------A-ALLSAVVTGTINVLSTFVGI-

Q9SFG0 -------------------------------------A-ALLSAVITGSINVLATFVGI-

Q8L7R8 -------------------------------------G-SLMSTLVTGIVGTSSTLLSM-

Q93Y91 -------------------------------------P-ALIATFILGFVNLGSLLLST-

P0AE24 -------------------------------------EQQMIATLVVGLTFMFATFIAV-

P0AEP1 -------------------------------------TEQMWGTVIVGLTNVLATFIAI-

P96710 -------------------------------------NAGFVTTCIVGVVEVIFTVIAV-

P54723 -------------------------------------EAALIGNIANGVISVIAVIFGI-

O34718 -------------------------------------EAALIGNIANGVISVLATFVGI-

P46333 -------------------------------------SASALGTMGIGILNVIMCITAM-

O52733 -------------------------------------SAALLAHIGIGIFNVIVTAIAV-

P0AGF4 -------------------------------------DIALLQTIIVGVINLTFTVLAI-

P21906 -------------------------------------DTALLQTISIGVVNFIFTMIAS-

P15729 -------------------------------------EKSLLITVITGFINILTTLVAI-

Q6AWX0 -------------------------------------GDATRVSILLGLLKLIMTGVAV-

Q93YP9 -------------------------------------TSSNVATFGVGVVQVVATGIAT-

Q94AF9 -------------------------------------SE-RLGSMIFGVFVIPKALVGL-

O04036 -------------------------------------PS-AIGTSVIATIMVPKAMLAT-

Q94KE0 -------------------------------------PS-SIGSMILAVIMIPKALLGL-

Q9SCW7 -------------------------------------PS-DIGTSILAVILVPQSIIVM-

Q4F7G0 -------------------------------------PS-DIGTTILAVILIPQSIVVM-

Q8LBI9 -------------------------------------TSGKLGTIAIACVQVPITVLGT-

P93051 -------------------------------------PT-RLGMIIYAVLQVVITALNA-

Q0WQ63 -------------------------------------ASPTLGSILYSIEQVVLTALGAT

Q3ECP7 -------------------------------------SS-KIGMIAMVVVQIPMTTLGV-

Q9LTP6 -------------------------------------PN-NVGVMMASVVQSVTSVLGI-

Q8GXK5 -------------------------------------SS-DIGFILTSIVQMTGGVLGV-

Q9JJZ1 -------------------------------------KDSSLASVTVGIIQVLFTAVAA-

P58354 -------------------------------------KDSSLASVVVGVIQVLFTATAA-

Q9NY64 -------------------------------------KDSSLASVVVGVIQVLFTAVAA-

Q9UGQ3 -------------------------------------LPPKDDAAIVGAVRLLSVLIAA-

P43562 -------------------------------------V-KLFTSSIPYFINMVLSLLPI-

Q9FYG3 -------------------------------------L----GNIFVGVSNLLGSVIAM-

P36035 -------------------------------------DAVTVIVVVTNIGAICGGMIFG-

P47186 -------------------------------------EMSFTFSIIQYCLGICATFLSW-

A6QLI1 -------------------------------------S--AVPHLVMTIIVPIGGQIADF

Q5W8I7 -------------------------------------S--ALPHLVMTIIVPIGGQLADY

A4FV52 -------------------------------------S--ALPHLVMTIIVPIGGQIADF

P34644 -------------------------------------A--AIPHLVMGCVVLMGGQLADY

Q66GI9 -------------------------------------S--AVPWSMMAFTGYIAGFWSDL

Q9FKV1 -------------------------------------SSKMVPYLNMFVFSIVGGFIADY

Q46916 -------------------------------------A--SVPAVCGFIGGVLGGIISDW

Q91Y77 -------------------------------------F--MCIGVTSGVGRLLFGRIAD-

Q8TF71 -------------------------------------L--MCIGVTSGVGRLLFGRIAD-

A1L1W9 -------------------------------------L--ACIGITSGVGRLIFGRVAD-

P36021 -------------------------------------L--VCIGATSGLGRLVSGHISD-

O35308 -------------------------------------L--SIVGFVDIVARPACGALAGL

O95907 -------------------------------------L--SIVGFVDIVARPACGALAGL

Q90632 -------------------------------------L--SIIGFIDIFARPACGMVAGL

O35910 -------------------------------------L--TILGFIDIFARPTAGFITGL

O15427 -------------------------------------L--TILGFIDIFARPAAGFVAGL

P57788 -------------------------------------L--TILGFIDIFARPICGMVAGL

O15375 -------------------------------------I--SIIGFSNIFLRPLAGLMAGR

Q6ZSM3 -------------------------------------M--SILGVIDIIGNITFGWLTDR

Q8BGC3 -------------------------------------M--SILGVIDIVGNITFGWLTDR

Q503M4 -------------------------------------M--SILGVIDIVGNITFGWLTDR

O15403 -------------------------------------L--STMAIAEVFGRIGAGFVLNR

Q8NCK7 -------------------------------------V--AVAAMGDAGARLVCGWLADQ

Q7RTY0 -------------------------------------L--SVVAISDLVGRVVSGWLGDA

Q5R5M4 -------------------------------------I--SIIGIMTAVGKLLLGILADF

Q7TM99 -------------------------------------I--SIFGIMTAVGKLLLGILADF

Q5ZJU0 -------------------------------------V--SIIGIMTAIGKLILGILADF

O15374 -------------------------------------V--SVAGILETVSQIISGWVADQ

Q08777 -------------------------------------I--MIINVCGIPGRWVPGYLSDK

Q08268 -------------------------------------I--TASNAVGILGRYIPGYFADK

P39709 -------------------------------------S--MITPGL----GMVYLMLTGI

P25621 -------------------------------------P--SGIFAV----GIVSTLCSAV

O13880 -------------------------------------N--APIWLA----DALGIVTVMP

P40445 -------------------------------------A--IPGQAL----LLINLPLAAL

P15365 -------------------------------------G--LPTGAVELVGCPLFGILAVY

Q07904 -------------------------------------Q--LPIGAI----TAMIILITTQ

P53322 -------------------------------------T--VPIYFL----TAIVFFICAV

P32071 -------------------------------------VSTIIGIIIGGAIYLPTVY---Y

P38124 -------------------------------------MGFCVGCVLAYGLFG-ILN---M

Q06451 -------------------------------------IPIVIGALWA-LATTFYCE--NK

P53283 -------------------------------------IPIVIGALWA-LATTFYCE--NK

Q9C0R8 -------------------------------------IPIGVGVIIATIAAPFFNKDYNR

Q9C0Q6 -------------------------------------IPIGVGVIASSLAAPFFNKDYNR

Q9HF77 -------------------------------------LAMLIGILIGGGMIMLFEK---R

Q5A0E9 -------------------------------------LAMLLGVIIGIAALILFEQ---R

Q07824 -------------------------------------IALIIGMMVCAAFIWYMDN---D

O59698 -------------------------------------IGILVGVFIGCGINCLFEP---W

O74829 -------------------------------------LAIFVGVCIGCSSVALFQP---Y

P38776 -------------------------------------IGIMVGMILSAPTCLLFQK---T

Q9HDX4 -------------------------------------ISLSIGQVFAVFVLLPLNQ--KY

P38227 -------------------------------------LAITFSAISFSTVYFVNMTVEYK

P38125 -------------------------------------ITSVSTALLFSSYYAFSVTFSY-

P40474 -------------------------------------ILMLVAGLQFAMYTTHLTALSTA

O94607 -------------------------------------LGSCVLIFTYFMSYYIFSNFLTS

Q9HE13 -------------------------------------SSVMVTSFLHYYIMSTVTYYIPI

Q04301 -------------------------------------CLSSAVTVISSFVVFGEIFRSPI

P13090 -------------------------------------IQIMLALFFGWGSFGIFTFYYFQ

Q08902 -------------------------------------F-VLSCIAAGWASFGIWIFYTWQ

P39886 -------------------------------------AGTVLMVLMAFSF-IGGLFFVTF

P76269 -------------------------------------SLSICTSVCSFCAQMLAMVSLPF

Q8Y9K8 -------------------------------------TLGLILVFISFMMILSTMILLPL

P28873 KFTKP-------------ILRQEQVFPEVFIPIAIVGGILLTSG----LFIFG-------

A4WFG6 ILR-F-------------------------FDLQRILTVLAGLA----TVLMY-------

A1JSB0 ILR-F-------------------------FDLQRIVTVLAALA----TLSMY-------

A8GKP6 ILR-F-------------------------FDLQRIVTILAALA----TGAMY-------

P57601 IIK-F-------------------------FNLYRMFIFLTSMS----TILMY-------

Q8K942 IIK-F-------------------------FNLQRMFIFLTGSS----SVLMY-------

Q89A60 ILK-F-------------------------FDLKKTIITLSGIS----LFLMS-------

P76198 LLKKM-------------------------VRPIWANVFNSALA----TITAA-------

O05390 VGDKL----------------------GWRNTVMWFGGVGCGIF----TLALY-------

O34864 VGDKF----------------------GWKNTVIWFGGVGCGIF----TVLLY-------

O52717 VAEKM----------------------GWMKVIRWFGCIGMALS----SLAFY-------

O52718 LGEKL----------------------GWMKVVRWFGCIGMALS----SLAFY-------

A9MJT5 ALGL------------------------FKPRTLMLVSQGLFLT----AGVTL-------

A6TG19 VLSL------------------------FNPRTLMLTSQVLFLA----AGVTL-------

A0L190 LLNQ------------------------FKEKSLILFSQTLFAA----AALVF-------

P0AEY8 LTSRR----------------------TVRSLIIMG-GWPI-MI----GLLVA-------

Q7CP73 LVKDP----------------------T-RPRFIWR-AVPIQLS----GLATL-------

P39386 FVKDP----------------------T-EPRFIWR-AVPIQLV----GLSLL-------

Q68WD6 LIKKR----------------------QVHDKKVMSIGFIFSLC----GCILF-------

Q4UMJ9 LIKKR----------------------HVHDKKVMGLGFIFSLC----GCILF-------

Q1RI77 LIKKR----------------------HIHDQKVMILGLVFSVI----GCSLL-------

P32482 VIPK------------------------WGSPSVLRMGMGCLIA----GAVLL-------

P37597 ALQK------------------------WQGKQLLPWLLVLFAV----SVIAT-------

P45123 FVTK------------------------VGAETMLRIALAIQFL----SGMWL-------

P28246 FVRR------------------------IGALNMFRSGLWIQFI----MAAWM-------

P31442 PNKR--------------------------FSTLMWQSVICCLL----AGLLM-------

P37482 LADRV----------------------KQQRGIVAALASVYLIG----LCGLL-------

P76242 FLHHV----------------------KDQRGIAAFVALMCAVG----AVGLC-------

P17583 MARH-----------------------QDRRKLLMLALVLQLVG----FCGFI-------

P0C105 AMRYI----------------------DDGKALAFNAFVAIILL----FITVA-------

O25788 LMNKI----------------------APNKYLAFNALSSIVLI----ALAII-------

P11551 LISRF----------------------APHKVLAAYALIAMALC----LISAF-------

P44776 LMKYL----------------------KAEFMLMLFAIGGFLSI----LGVIF-------

A1A9E1 LADKF-------------------------GRLLVLRVQVFVVI----LGSIA-------

A9MHY5 LADKF-------------------------GRLLVLRVQVFVVI----LGSIV-------

A4W8S1 LADRY-------------------------GRLLVLRVQVFVVI----LGCLA-------

A1JMG4 MADRY-------------------------GRLLVLRIQVFVVI----LGSIA-------

Q0TK80 LAHSV--------------------------RPGLLMLLSTLGA----FLAIG-------

O06473 ALGTV--------------------------RSLVVLFIAFAAI----LVIMG-------

Q2FI61 IDNR------------------------SRNFMYGVILSGFILY----MILIF-------

Q5HQE8 IDNN------------------------SKGFMYGVIFSGFILY----TILIF-------

Q49WE5 IDKH------------------------GTLFMYWVIFGGFVLY----TLMIF-------

Q4L523 IDKN------------------------SKGFMYAVIFTGFILF----TAFIF-------

P33026 FAKRL----------------------GKRFLMRVAAVGGVCFY----AGMLM-------

P31436 YMKRI----------------------GKRLLMLIAIVSGMCFY----ASVLM-------

Q9S3K0 LTSRL----------------------GKRLLMRLAVIAGLIFY----TGLTL-------

P31675 YVKRY----------------------GKRRMMVIAVAAGVLFY----TGLIF-------

P31126 RLNPA----------------------NIRALMTAGTLCFVIGL----VGFIF-------

Q58955 FGKLY----------------------DKLG--NIMIIIGIFII----SFGMY-------

P02920 APLII----------------------NRIGGKNALLLAGT-IM----SVRII-------

Q4UK37 LAAKM----------------------GIFHSVLIGGVIQLLSP----LMFMI-------

Q1RKF6 LSTKI----------------------GILRSVLTGGIIQLLSP----LMFMI-------

Q4ULW4 VMYKV----------------------GNFKGLIITGIAQSLTH----FAFIW-------

Q1RI01 VMYRF----------------------NNFKGLIITGILQSITH----FAFIW-------

Q68WQ5 IMKYK----------------------NMLYSILLFGIIHALSH----ILFIL-------

Q4UL88 IMKHK----------------------NILNSIFLFGIIHALGH----ILFIF-------

Q92HQ3 IMKHK----------------------NILNSIFLFGIIHALGH----ILFIF-------

Q1RIL0 IMKKK----------------------NILDSILLFGIIHALAH----ILFII-------

Q4UMU2 LCRKY----------------------EYFYLLKRALIYHALSS----LSFLF-------

Q68W71 LCRKY----------------------EYSYLLKRVLIYHALSS----LSFIY-------

Q1RHK8 LCRKY----------------------EYTYIFKRVLVYHALSS----ITFLL-------

Q1LTM2 WSEKR----------------------FSLENRFMAGLTIMLLS------IIP-------

Q2NTK5 WSEKR----------------------FRLETRLMAGLTVMLLS------LFP-------

P55705 WARTF----------------------EGREPSFVLGCNVLIAG----GCFVA-------

Q7Z3Q1 ---SY----------------------CMEDIHMAFIGIFTTMT----GMAMT-------

Q05B81 ---QY----------------------CLADTWVAEIGLVFNIL----GMMVF-------

P76470 YSDK-----------------------TGERRNVAALTLLAAGI----GIGLS-------

P32135 ISDKI----------------------LKSPSKYLCYTFIISTA----ALVLL-------

P38358 SMSKY----------------------NCLRLLLYIGISLMFLG----NFLFL-------

A4WAE6 LGNKH-----------------------ASLLVSSAIGLLLACL----LLLMP-------

A6T8Y8 LGNQH-----------------------ASGLISLAIALLLACL----LLLLP-------

Q6CZ44 YSSKY-----------------------PAGFLIVSFAFLAVCL----LLLLP-------

Q888L8 FSDRF-----------------------PNGLLITAIGTLAVCL----LMLLP-------

Q1IB51 YSERF-----------------------PHGFLVMAIAALAACL----LLLLP-------

A6UZY0 YSEAF-----------------------PRGFLLAAILALGTSL----ALLLP-------

Q4QP52 LYRFA-----------------------PTKFIVVSMSLLMFSL----LLLLF-------

Q9CM87 YYNAG-----------------------PIRFILFSMGLLTATL----LLLFI-------

O25797 LYAKN-----------------------SRKFIAFAMVLVICPQ----LLLFV-------

Q17YP7 FYEKN-----------------------PKKFIACAIILVLCPQ----LLLFS-------

P77389 LADRS-----------------------VNGTLKGFLLLLMVIM----LAIPF-------

O31577 VPLPL-----------------------LTRTMIGVMIGLIGVL----AVFPY-------

O34367 LSNRN-----------------------PIAALFYMFIVQAIVL----FVLTF-------

A1AHK2 ILKRS-----------------------VKLALAGAPFVLALSA----LVLTL-------

A9MWE8 VLKRS-----------------------VKLALAGAPLLLALSA----LTLIV-------

P23910 ISGRY------------S----------PLRIAAVTDFIIVLAL----LMLFF-------

P31141 LSDRR-----------------------PAQVLAVAGPLLLVGW----PALAM-------

P43531 MTTRY------------------------GRGPVMLFSTGVMLF----GLLMT-------

Q8K902 LINQY------------------------NRSSILRIALLLMIL----GLLMT-------

P57648 LINKY------------------------NRNNILIVSLMLMII----GLFIT-------

Q89A23 LIERY------------------------RKGVILTLALTMMIF----GVLIT-------

P37498 MVDRY------------------------GYPKILVMNIFIVIA----GALFT-------

P0A0J4 KFMKY------------F----------SELTFIAWSLLYSVVV----LILLV-------

Q07282 FIAKH------------L----------GERKAIAVGILADGCG----LFLLA-------

P02982 PVAAR------------L----------GERRALMLGMIADGTG----YILLA-------

P02981 PATKR------------F----------GEKQAIIAGMAADALG----YVLLA-------

P70187 LLMRS------------I----------GNKNTILLGLGFQILQ----LAWYG-------

Q5SR56 ILMRS------------L----------GNKNTVLLGLGFQMLQ----LAWYG-------

P77726 AEVKR------------K----------MKQV-FVFCVGLIVVA----EIVLW-------

Q89AA9 IQSKV------------L----------YSNITITTSAFLFVLC----YGIFL-------

Q8K999 FKFHF------------F----------LKNI-IEICIFFIFLS----LLLFL-------

P57538 CKYKY------------I----------LENI-IEICILFILFS----EIIFL-------

Q5HIA2 LADKI------------G----------EKKVFLIGTGGLTLFS----IIAFM-------

Q4L3Q4 LADKI------------G----------EKKVFLIGTGGLTLLS----IVAFS-------

Q5HRH0 IADRI------------G----------EKKVFLIGLGGLILLS----VVAFS-------

P0C0L7 LSDRF------------G----------RRPFVLLGSVALFVLA----IPAFI-------

Q47421 LSDRF------------G----------RKPFVVIGSVAMFFLA----VPSFM-------

P0A2G3 ISDRI------------G----------RRAVLMGITLLALITT----WPVMQW------

P16482 LSDRF------------G----------RRSVLIAMTLLALATA----WPALTM------

P0AEX3 LSDKI------------G----------RRTSMLCFGSLAAIFT----VPILSA------

P76350 LADRF------------G----------RRRVYITGTLIGTLSA----FPFFM-------

P41036 LPTYL------------KTDLAYNPHTVANVLFFSGFGAAVGCC----VGGFLGDWLGTR

Q9SYQ1 FIDKI------------G----------RVKIQIMGFFFMAVIY----LVAGI-------

Q9S735 FIDKI------------G----------RVKIQMMGFFLMAVVY----LVAGI-------

P76230 VMDKI------------P-----------RKTMGVGLLILIAVL----GYIYS-------

P38055 IIDHF------------P-----------RRLFGSTLLIIIAVL----GYIYS-------

Q46909 LTHLL------------A----------HRKFLLGSFLLLAATL----VVMAC-------

P31679 WLNTA------------G----------RRPLLIGSFAMMTLAL----AVLGL-------

O24723 ILKRF------------D-----------RFIALAAFIFWSVVG----LLIVA-------

O30513 LADRY------------N----------V-KFVTMGLLLLGAIS----LSLLS-------

Q43975 AMDRF------------N----------PNRIIAGFYLAAGIFA----VIVGQ-------

Q51955 AMDRY------------N----------PHKVIGIFYLLAGVFA----YAVGQ-------

Q9I6Q3 AMDRF------------N----------PHKVIGLFYLLAGVFA----WCVGQ-------

P77589 LMDKL------------R----------P--VTMSLLIYSGMLA----SLLAL-------

P94131 MADKL------------G-----------RRFTYAFGAIGTAIF----LPLIV-------

P71369 LADRI------------G-----------RKPSFLLFQLGAVIS----IVVYS-------

O34691 LIEKA------------G-----------RKWILVVYLIGTAGS----AYFFG-------

P0AGC0 LSD-L---------------------ANGRRGLVACIALALIIA----TLGVY-------

P27669 GSDKL---------------------FNGNRGPMNLIFAAGILL----SVGSL-------

P96335 VSDKV---------------------FKGKRGLTGFIFMILTTA----AVVAY-------

P08194 MSDKV---------------------FRGNRGATGVFFMTLVTI----ATIVY-------

P37948 ISDRF---------------------FKSRRAPAGVLFMAGVFI----AVLVY-------

P12681 LSDKL---------------------FKGRRMPLAMICMALIFV----CLIGY-------

Q5M7K3 ISDYT-----------------------GKSAITCTIMLILTAP----MLFIY-------

Q9WU81 ISDYT-----------------------NSRATTCCIMLILAAP----MMFLY-------

Q58CV5 VSDYI-----------------------NGRATTCCVMLILAAP----MMFLY-------

Q7SY29 VSDYT-----------------------GGRASTCCAMLIIAAP----MLFLY-------

P57057 ISDRL-----------------------EKRASTCGLMLLLAAP----TLYIF-------

Q17QZ3 ISDML-----------------------QKRAPVLALSLLLAIG----SLVGY-------

Q3TIT8 ISDVL-----------------------QKRAPVLALSLFLAVW----SLVGY-------

Q640L2 ISDLM-----------------------KMRSPVLTVSLLLAVG----ALFGY-------

Q5F3N0 ISDVL-----------------------QKRAPVLAISLLFAVG----SLFGY-------

Q09037 LASRF------------TM---------RQMFITCSFVASCMCL----FLCGI-------

Q06222 LASRF------------TM---------RQMFITCSFVASCMCL----FLCGI-------

P13865 LSYVF------------TM---------RHVFLFGSIFTSCMCL----FMCGI-------

P46499 FIPSF------------NR---------QRLHQVPQIIMIACYT----TIMAL-------

P54219 LANK------------------------MGRWLCSLIGMLVVGT----SLLCV-------

Q6NT16 LSDKR------------PP---------LRKWLLVFGNLITAGC----YMLLG-------

Q8R0G7 ISRRL------------RR------FNPRADPLVCAAGLLGSAP----FLFLA-------

Q5XGK0 ISKRY------------RK------TNPRADPLVCAVGMISSAP----FLFLS-------

Q7ZU13 ASKLL------------RT------RTPRADPLVCAAGLLLAAP----FLYLS-------

A2CER7 ISKKL------------RD------RVPNADPLICAVGMLSSSP----CFFIA-------

Q9D232 ASRRY------------KK------VNPRAEPLICASSLFATAP----CLYLA-------

Q6ZMD2 AARRY------------KK------VIPGAEPLICASSLLATAP----CLYLA-------

Q6GPQ3 VKILC------------KK------T---GERVLLLGGLAVIWI----GFFIL-------

A5IVG9 LGD----------------------KFNAVKVLMIDFVIMIIGA----VILGISD-----

Q5HLK7 IGD----------------------KFNAVQALIIDFVIMIIGA----LILSLSS-----

P46907 LAD----------------------KMSPLRILMFVFTGLTLSG----IILSFSP-----

P10903 LSD----------------------RLGGTRVTLVNFILMAIFS----GLLFLTLPTDGQ

P37593 ISD----------------------KFGGVRVTLINFIFMALFT----ALLFLTLPGSG-

P37758 ISD----------------------KFGGVRVTLINFIFMAIFS----ALLFLTLPGTG-

Q9P3K8 LTDLLAPSPQARHVQITSSGTLERKRFSLSRVSFLLFFAVTLSV----GLATLAS-----

Q6FWD4 TVDY-----------------FNKRQLSVKWILLLFLVVGLVTQ----GKIYMLSMSSLD

Q6CPY8 FIDL-----------------FTKWNWPRIPLIILMLLSAILAQ----V-IIIHAMNVVN

P22152 LADFL----------------YRKTNTPWAKKLLLSFLGVVMGA----FMIAMGFSDPKS

Q02563 LLMDK----------------------IGRLRMLAGSSVLSCVS----CFFLS-------

Q496J9 LLMDR----------------------IGRLTMLGGSMVLSGIS----CFFLW-------

Q63564 LLMDR----------------------IGRLKMIGGSMLISAVC----CFFLF-------

Q1JP63 WIIDR----------------------LGRKKTMALCFVVFSFC----SLLLF-------

Q2XWK0 WIIDR----------------------VGRKKTMAICFIIFSFS----ALLLF-------

Q1LVS8 ILLNI----------------------VGRKYSMVILLLLSAFF----FMLVN-------

O08966 VTIDR----------------------IGRIYPIAASNLVAGAA----CLLMI-------

O15245 ITIDR----------------------VGRIYPMAMSNLLAGAA----CLVMI-------

O77504 VTIDR----------------------VGRIYPMAASNLAAGVA----SVILI-------

A7MBE0 VTIDR----------------------FGRRYPLATSNLAAGLA----CFLMI-------

Q9R0W2 LTIDR----------------------VGRRYPWAVSNMVAGAA----CLASV-------

O02713 LTIDR----------------------LGRRHPWAASNVVAGAA----CLASV-------

Q8MJI6 ATIDR----------------------VGRRYPWAVSNMVAGAA----CLASV-------

O88446 LTIER----------------------LGRRLPFAASNIVAGVS----CLVTA-------

O75751 LTIER----------------------LGRRLPFAASNIVAGVA----CLVTA-------

Q9U539 PLLQC----------------------AGRRWTLAATMIVCGIG----CVSAM-------

O76082 LLLQY----------------------LPRRYSMATALFLGGSV----LLFMQ-------

Q497L8 IWLKR----------------------VGRRKTMLLFLLVSSLT----CMLHV-------

Q86VW1 IAMDK----------------------VGRRTVLAYSLFCSALA----CGVVM-------

Q17QN9 LGMDR----------------------VGRRNILIFSLLSSAVT----SGVIM-------

Q95R48 LTLNR----------------------WGRRSILCGCLVMAGLS----LLATV-------

Q9Y267 FLLQQ----------------------IGRKWSLAVTLLQAIIW----CLLLL-------

Q6A4L0 LMMEK----------------------LGRKWSQLCTLTLAGIM----YIIII-------

Q9Y226 FMMQR----------------------FGRKWSQLGTLVLGGLM----CIIII-------

Q8IVM8 WALKY----------------------MNRRASQMLLMFLLAIC----LLAII-------

Q66J52 FITTY----------------------VGRRVSQAITLILAGIA----ILVNI-------

Q91WU2 FLVRL----------------------VGRRLTEAGMLLATALT----FGISL-------

Q8IZD6 YLINQ--------------------KWFGRKRTLSAFLCLGGLA----CLIVM-------

Q6DFR1 YLINH--------------------KRVGRRGSLAGFLCVGGGA----CLLIM-------

Q6NUB3 YLINH--------------------KRVGRRRSLAGFLFLGGGS----CLLIM-------

Q28ES4 YFIEK--------------------SWSGRRKATAGFLGFAGFA----CIFTI-------

A6NKX4 LTADC----------------------CGRRPVLLLGTMVTGLA----SLLLL-------

P47185 MVVDK----------------------IGRRKCLLFGAASMMAC----MVIFA-------

P13181 WTVEN----------------------LGHRKCLLLGAATMMAC----MVIYA-------

P23585 YTVDK----------------------FGRRKCLLGGSASMAIC----FVIFS-------

Q92339 YTIDN----------------------LGRRNPLIFGAAFQSIC----FFIYA-------

O74969 YVLER----------------------FGRRNPLIIGGIWQSIC----FFIYS-------

P10870 FFVEF----------------------FGRRKVLVVGGVIMTIA----NFIVA-------

Q12300 YLVDR----------------------IGRRPVLLAGGVIMAIA----NLVIA-------

P42833 YYVEK----------------------WGHKTCLLYGSTNLLFY----MMTYA-------

P49374 FLIDK----------------------FGRRPVLIIGGIFMFTW----LFSVA-------

O74713 YFIDK----------------------VGRRPLLIGGATMMMAF----QFGLA-------

Q9BE72 LLVDH----------------------VGSKTFLCIGSSVMAAS----LVTMG-------

Q5J316 LLVDQ----------------------VGSKTFLCIGSSVMAAS----LVTMG-------

Q32NG5 FLVDK----------------------IGSKTFLCIGSAVMAVS----LVSVG-------

Q6NWF1 FLVDK----------------------VGPKALLCVGVVVMMLS----TATLG-------

Q0P4G6 CFADK----------------------AGRRILLLAGCIVMTIA----ITGIG-------

Q6GN01 CFADK----------------------AGRRILLLAGCIVMTIA----ISGIG-------

O95528 GLVDR----------------------AGRRALLLAGCALMALS----VSGIG-------

Q8VHD6 GLVDR----------------------AGRRVLLLFGCALMALS----VSGIG-------

Q3UHK1 WLVEK----------------------VGRRKLTFGSLAGTTVA----LIILA-------

Q96QE2 WLVEK----------------------VGRRKLTFGSLAGTTVA----LIILA-------

Q9C757 YFIDR----------------------IGRKKLLIISLFGVIIS----LGILT-------

Q8VZR6 YFIDH----------------------CGRKKLALSSLFGVIIS----LLILS-------

P30606 FCIDK----------------------IGRRYILLIGLPGMTVA----LVICA-------

P30605 FSIDK----------------------IGRRTILLIGLPGMTMA----LVVCS-------

Q10286 GVIDF----------------------FGRRILLLLTVWGMIAA----LIVCA-------

Q04162 YTMDS----------------------LGRRAILLRTILIMTVG----LLLCS-------

Q01440 FTVDR----------------------FGRRRMLLISVFGCLVL----LVVIA-------

P11166 FVVER----------------------AGRRTLHLIGLAGMAGC----AILMT-------

P46896 FVVER----------------------AGRRTLHLIGLAGMAGC----AILMT-------

P47843 FLVER----------------------AGRRTLHLIGLGGMAFC----SILMT-------

P14672 LLVER----------------------AGRRTLHLLGLAGMCGC----AILMT-------

Q90592 FLVEK----------------------AGRRSLFLAGLMGMLIS----AVAMT-------

P11168 FLVEK----------------------AGRRSLFLIGMSGMFVC----AIFMS-------

Q5RB09 LVIEH----------------------LGRRPLLIGGFGLMALF----FGTLT-------

A4ZYQ5 VLVER----------------------LGRRHLLLAGYGICGSA----CLVLT-------

P22732 FVVEL----------------------LGRRLLLLLGFSICLIA----CCVLT-------

P58353 FVVEL----------------------MGRRFLLLLGFSVCFTA----CCVLT-------

Q863Y9 FVVEY----------------------LGRRALLLLGFSVCFIA----CCVLT-------

P43427 FVVEL----------------------WGRRNLLLIGFSTCLTA----CIVLT-------

Q9WV38 FVVEL----------------------WGRRFLLLVGFSTCLIA----CLVLT-------

P15686 MFSDK----------------------FGRRFLLIEGGIQCCLA----MLTTG-------

Q39525 LLSDK----------------------FGRRFLLIEGGITCCLA----MLAAG-------

Q94AZ2 YSVDK----------------------VGRRVLLLEAGVQMFFS----QVVIA-------

Q10710 ATVDR----------------------LGRRFLLISGGIQMITC----QVIVA-------

Q41144 YGVDK----------------------WGRRFLFLEGGVQMLIC----QAIVA-------

P23586 YGVDR----------------------WGRRFLFLEGGTQMLIC----QAVVA-------

O65413 YGVDK----------------------WGRRFLFLEGGFQMLIS----QVAVA-------

Q9SX48 YAVDR----------------------YGRRILFLEGGIQMIVS----QIVVG-------

Q9LT15 YAVDR----------------------YGRRLLFLEGGIQMFIC----QLLVG-------

Q9FMX3 YSVDK----------------------FGRRALFLQGGFQMIVT----QIAVG-------

Q9SBA7 FLVDK----------------------TGRRFLLLQSSVHMLIC----QLVIG-------

Q9SFG0 YLVDR----------------------TGRRFLLLQSSVHMLIC----QLIIG-------

Q8L7R8 LVVDR----------------------IGRKTLFLIGGLQMLVS----QVTIG-------

Q93Y91 MVIDR----------------------FGRRFLFIAGGILMLLC----QIAVA-------

P0AE24 FTVDK----------------------AGRKPALKIGFSVMALG----TLVLG-------

P0AEP1 GLVDR----------------------WGRKPTLTLGFLVMAAG----MGVLG-------

P96710 LLIDK----------------------VGRKKLMSIGSAFMAIF----MILIG-------

P54723 WLLGK----------------------VRRRPMLIIGQIGTMTA----LLLIG-------

O34718 WLLGR----------------------VGRRPMLMTGLIGTTTA----LLLIG-------

P46333 ILIDR----------------------VGRKKLLIWGSVGITLS----LAALS-------

O52733 AIMDK----------------------IDRKKIVNIGAVGMGIS----LFVMS-------

P0AGF4 MTVDK----------------------FGRKPLQIIGALGMAIG----MFSLG-------

P21906 RVVDR----------------------FGRKPLLIWGALGMAAM----MAVLG-------

P15729 AFVDK----------------------FGRKPLLLMGSIGMTIT----LGILS-------

Q6AWX0 VVIDR----------------------LGRRPLLLGGVGGMVVS----LFLLG-------

Q93YP9 WLVDK----------------------AGRRLLLMISSIGMTIS----LVIVA-------

Q94AF9 ILVDR----------------------WGRRPLLLASAVGMSIG----SLLIG-------

O04036 VLVDK----------------------MGRRTLLMASCSAMGLS----ALLLS-------

Q94KE0 ILVEK----------------------MGRRPLLLASTGGMCFF----SLLLS-------

Q9SCW7 FAVDR----------------------CGRRPLLMSSSIGLCIC----SFLIG-------

Q4F7G0 LTVDR----------------------WGRRPLLMISSIGMCIC----SFFIG-------

Q8LBI9 ILIDK----------------------SGRRPLIMISAGGIFLG----CILTG-------

P93051 PIVDR----------------------AGRKPLLLVSATGLVIG----CLIAA-------

Q0WQ63 LLIDR----------------------LGRRPLLMASAVGMLIG----CLLIG-------

Q3ECP7 LLMDK----------------------SGRRPLLLISATGTCIG----CFLVG-------

Q9LTP6 VIVDK----------------------YGRRSLLTVATIMMCLG----SLITG-------

Q8GXK5 LLVDI----------------------SGRRSLLLFSQAGMFLG----CLATA-------

Q9JJZ1 LIMDR----------------------AGRKLLLALSGVIMVFS----MSAFG-------

P58354 LIMDR----------------------AGRRLLLTLSGVVMVFS----TSAFG-------

Q9NY64 LIMDR----------------------AGRRLLLVLSGVVMVFS----TSAFG-------

Q9UGQ3 LTMDL----------------------AGRKVLLFVSAAIMFAA----NLTLG-------

P43562 TFIDY----------------------TSRKLITLLGGFPISGL----LITIG-------

Q9FYG3 VLMDK----------------------VGRKLLLLWSFIGMAAA----MALQV-------

P36035 QFMEV----------------------TGR--RLGLLIACTMGG----CFTYP-------

P47186 WASKY----------------------FGRYDLYAFGLAFQTIV----FFIIG-------

A6QLI1 LRSKQ--------------------ILSTTTVRKIMNCGGFGME----ATLLL-------

Q5W8I7 LRSKN--------------------ILTTTTVRKIMNCGGFGME----ATLLL-------

A4FV52 LRSRR--------------------IMSTTNVRKLMNCGGFGME----ATLLL-------

P34644 LRSNK--------------------ILSTTAVRKIFNCGGFGGE----AAFML-------

Q66GI9 L-IRR--------------------GTSITLTRKIMQSIGFIGP----GIALI-------

Q9FKV1 LITKR--------------------ILSVTRTRKFLNTVGFLIA----SAALM-------

Q46916 L-MRR--------------------TGSLNIARKTPIVMGMLL-----SMVMV-------

Q91Y77 -YLP-------------------------GVKKVYLQVLSFFFI----GLTSM-------

Q8TF71 -YVP-------------------------GVKKVYLQVLSFFFI----GLMSM-------

A1L1W9 -YVP-------------------------GVNKVFLQVSSFMVI----GVMSM-------

P36021 -SIP-------------------------GLKKIYLQVLSFLLL----GLMSM-------

O35308 GRLR--------------------------PHVPYLFSLALLAN----GLTDL-------

O95907 ARLR--------------------------PHVPYLFSLALLAN----GLTDL-------

Q90632 KWVR--------------------------PHVAYLFSFAMLFN----GSTDI-------

O35910 KKVR--------------------------PYSVYLFSFAMFFN----GFTDL-------

O15427 GKVR--------------------------PYSVYLFSFSMFFN----GLADL-------

P57788 KWVR--------------------------PRCVYLFSFAMIFN----GFTDL-------

O15375 PAFA--------------------------SHRKYLFSLALLLN----GLTNL-------

Q6ZSM3 RCLK--------------------------NYQYVCYLFAVGMD----GLCYL-------

Q8BGC3 RCLK--------------------------NYRYVCYLFAVALD----GLCYL-------

Q503M4 RCLK--------------------------KYRNICYMFAVGME----GLCCL-------

O15403 EPIR--------------------------K--IYIELICVILL----TVSLF-------

Q8NCK7 GWVP-------------------------LPRLLAVFGALTGLGLWVVGLVPV-------

Q7RTY0 VPGP-------------------------VTRLLMLWTTLTGVSL---ALFPV-------

Q5R5M4 KWI----------------------------NTLYLYVATLIIM----GLALC-------

Q7TM99 KWI----------------------------NTLYLYVATLIIT----GLALC-------

Q5ZJU0 KWV----------------------------NTLYLYVLTLLMM----GAALL-------

O15374 NWI----------------------------KKYHYHKSYLILC----GITNL-------

Q08777 F-GR---------------------------FNVAIATLLTLFI----VMFVG-------

Q08268 FIGR---------------------------FNVEIITISMAAL----FNFVM-------

P39709 IADK------------------------L-HSR-WFAIIFTQVF----NIIGN-------

P25621 YMSK------------------------IPRARHWHVSVFISLV----MVIVA-------

O13880 LYDR------------------------FHKKFSFFTGSCLII-------IAG-------

P40445 LSRK------------------------LKEKSLCVGIANVWVL----PFIVS-------

P15365 AANKK--------------------IPFWKYKLSWAIFAAVLAL----IASCM-------

Q07904 MLSR------------------------WGH-ITLITTSMYIPA----IIGCI-------

P53322 WSDR------------------------IKLRSPFILGACLTTS----IGIAI-------

P32071 KFTKK-------------LLAGQNVTPEVFLPPAIFGAICMPIG----VFIFG-------

P38124 RIIVP-------------RFRNGTFTPEAFLIVAMCVCWCLPLS----LFLFG-------

Q06451 YLQIV-------------KQR--KPTPEDRLLGAKIGAPFAAIA----LWILG-------

P53283 YLQIV-------------KQR--KPTPEDRLLGAKIGAPFAAIA----LWILG-------

Q9C0R8 RAQVY-------------RDRGELPPPELRLIPMMIACWFVPVG----LFAFA-------

Q9C0Q6 RAQEY-------------RDRGELPPAELRLIPMMIGCWFVPAG----LFAFA-------

Q9HF77 YIKAM-------------EDNGGKIIPEKRLEPMMVGGFTFVIG----IFWLG-------

Q5A0E9 YLKAM-------------EINNVNPIPEERLPPVFIGGIFFIIG----IVILT-------

Q07824 YLKRC-------------RAKG-KLVPEARLYAMVIAGTVFPIG----ILWFC-------

O59698 YFRQV-------------IKAGNKPAPEARLPPMMIGSFLFPAG----IFWLA-------

O74829 YFKKM-------------DENKGRPVPEARLPSMMIGCIVFPIG----IFWLA-------

P38776 FEWRV-------------KRNNGVKTPEMRFEPLFYGAFLTPVG----LFIFA-------

Q9HDX4 WLSAV-------------QKNNGVPEPEFRLPMAFLGCFAIMTG----MFIFG-------

P38227 YSRPP--------------------YNFKPLYIGLLYIPNSVTY----FFASI-------

P38125 YLEHD--------------------YRFTMLEIGAAYVCPGVAM----LLGSQ-------

P40474 LSK-Q--------------------YHLTVAKVGLCYLPSGICT----LCSIV-------

O94607 FLQVS--------------------YGLSIDMSSLTLNVFVFSM----TTTAI-------

Q9HE13 YFQSI--------------------KGDGPLMSGVHTLSLAVVS----SVVSA-------

Q04301 YLQLL--------------------QNISVTKTGLFLIFPSISV----AVGSL-------

P13090 FQLNI--------------------RQYTALWAGGTYFMFLIWG----IIAAL-------

Q08902 FMEDS--------------------RGQTPLLSSAQFSPVAISG----FCAAV-------

P39886 YLQNV--------------------HGMSPVESGVHLLPLTGMM----IVGAP-------

P76269 YLQTV--------------------LGRSEVETGLLLTPWPLAT----MVMAP-------

Q8Y9K8 YLQNS--------------------LALAAFSAGLVLLPGGVLN----GLMSP-------

P28873 ------WSAN--RTT---------------------------------------------

A4WFG6 ------LFIN-GA-----------------------------------------------

A1JSB0 ------MFVSTNN-----------------------------------------------

A8GKP6 ------LFVSTDN-----------------------------------------------

P57601 ------CFIKSEN-----------------------------------------------

Q8K942 ------CFIYSKS-----------------------------------------------

Q89A60 ------LFNIFYD-----------------------------------------------

P76198 ------IIYLYPS-----------------------------------------------

O05390 ------YTPQLIG-----------------------------------------------

O34864 ------YAPVFSG-----------------------------------------------

O52717 ------YLPQHFG-----------------------------------------------

O52718 ------YIPQHFG-----------------------------------------------

A9MJT5 ------SLAHT-------------------------------------------------

A6TG19 ------SLATR-------------------------------------------------

A0L190 ------ILTQLGW-----------------------------------------------

P0AEY8 ------AAATVIS-----------------------------------------------

Q7CP73 ------LLGNLLL-----------------------------------------------

P39386 ------IVGNLLS-----------------------------------------------

Q68WD6 ------VVNAFILEFILVSN----------------------------------------

Q4UMJ9 ------AVNAFILEVVSASH----------------------------------------

Q1RI77 ------AIDALILQDKEVGQ----------------------------------------

P32482 ------AITEIWA-----------------------------------------------

P37597 ------WAAGFIS-----------------------------------------------

P45123 ------ILTALLD-----------------------------------------------

P28246 ------VISALLG-----------------------------------------------

P31442 ------WIPDWFG-----------------------------------------------

P37482 ------AGGSHT------------------------------------------------

P76242 ------FMPAHA------------------------------------------------

P17583 ------WLPMQ-------------------------------------------------

P0C105 ------TTGHI-------------------------------------------------

O25788 ------IGGKI-------------------------------------------------

P11551 ------AGGHV-------------------------------------------------

P44776 ------IDGVW-------------------------------------------------

A1A9E1 ------MLS---------------------------------------------------

A9MHY5 ------MLT---------------------------------------------------

A4W8S1 ------MLG---------------------------------------------------

A1JMG4 ------ILG---------------------------------------------------

Q0TK80 ------LFGLMP------------------------------------------------

O06473 ------IW--TD------------------------------------------------

Q2FI61 ------TLSMIV------------------------------------------------

Q5HQE8 ------GLSTIT------------------------------------------------

Q49WE5 ------ALSLIT------------------------------------------------

Q4L523 ------GLSLVT------------------------------------------------

P33026 ------AHSPVI------------------------------------------------

P31436 ------ATTPAV------------------------------------------------

Q9S3K0 ------LNGSWA------------------------------------------------

P31675 ------FNSRMA------------------------------------------------

P31126 ------SGNSLL------------------------------------------------

Q58955 ------LLSTST------------------------------------------------

P02920 ------GSSFAT------------------------------------------------

Q4UK37 ------LATIGY------------------------------------------------

Q1RKF6 ------LAIVGY------------------------------------------------

Q4ULW4 ------LNHQPP------------------------------------------------

Q1RI01 ------LNGQPP------------------------------------------------

Q68WQ5 ------LEVNGK------------------------------------------------

Q4UL88 ------LEINGK------------------------------------------------

Q92HQ3 ------LEINGK------------------------------------------------

Q1RIL0 ------LKIHGK------------------------------------------------

Q4UMU2 ------LYFYNR------------------------------------------------

Q68W71 ------LYFLNQ------------------------------------------------

Q1RHK8 ------LYSYSQ------------------------------------------------

Q1LTM2 ------IGMIHNL-----------------------------------------------

Q2NTK5 ------IGLIEDL-----------------------------------------------

P55705 ------TGLAGST-----------------------------------------------

Q7Z3Q1 ------AFASTTLM----------------------------------------------

Q05B81 ------AFATITPL----------------------------------------------

P76470 ------GLLS----P---------------------------------------------

P32135 ------IMLPHESMP---------------------------------------------

P38358 ------FIEKTSP-----------------------------------------------

A4WAE6 ------AAQSETHLA---------------------------------------------

A6T8Y8 ------ASHNPQHLM---------------------------------------------

Q6CZ44 ------LSFSGWSLS---------------------------------------------

Q888L8 ------LSGDATMLG---------------------------------------------

Q1IB51 ------LSGNFYLFA---------------------------------------------

A6UZY0 ------FSAQPTWLM---------------------------------------------

Q4QP52 ------STEAIIAMF---------------------------------------------

Q9CM87 ------ASQQTWTMF---------------------------------------------

O25797 ------FKNLEWVVF---------------------------------------------

Q17YP7 ------FKHLEWVIF---------------------------------------------

P77389 ------LARNEFGAA---------------------------------------------

O31577 ------IAIYPAAAI---------------------------------------------

O34367 ------TAPYQAAGL---------------------------------------------

A1AHK2 ------WGSDKIVAT---------------------------------------------

A9MWE8 ------WGSDKTVAA---------------------------------------------

P23910 ------CGGMKTTSL---------------------------------------------

P31141 ------LADRPVALL---------------------------------------------

P43531 ------LFSSLWLIF---------------------------------------------

Q8K902 ------QYNEIFIII---------------------------------------------

P57648 ------QYNQLFIII---------------------------------------------

Q89A23 ------QCNIVLLII---------------------------------------------

P37498 ------INNMLAVKI---------------------------------------------

P0A0J4 ------FANGYWSIM---------------------------------------------

Q07282 ------VITQSWMVW---------------------------------------------

P02982 ------FATRGWMAF---------------------------------------------

P02981 ------FATRGWMAF---------------------------------------------

P70187 ------FGSEPWMMW---------------------------------------------

Q5SR56 ------FGSQAWMMW---------------------------------------------

P77726 ------NAQTQFWQL---------------------------------------------

Q89AA9 ------LFGHNNISL---------------------------------------------

Q8K999 ------LSQHNLICL---------------------------------------------

P57538 ------SAQKNLLFL---------------------------------------------

Q5HIA2 ------LLHSQSFVV---------------------------------------------

Q4L3Q4 ------LLNTKSLPF---------------------------------------------

Q5HRH0 ------LMQLQSLFF---------------------------------------------

P0C0L7 ------LINSNVIGL---------------------------------------------

Q47421 ------LINSDIIGL---------------------------------------------

P0A2G3 ------LTAAPDFTR---------------------------------------------

P16482 ------LANAPSFLM---------------------------------------------

P0AEX3 ------LQNVSS-PY---------------------------------------------

P76350 ------ALEAQSIFW---------------------------------------------

P41036 KAYVCSLLASQLLII---------------------------------------------

Q9SYQ1 ------PYSWYWSKHEHNNKG---------------------------------------

Q9S735 ------PYSWYWSKHEKTNKG---------------------------------------

P76230 ------LQTSMLL-----------------------------------------------

P38055 ------IQTTEWA-----------------------------------------------

Q46909 ------LPS-GSS-----------------------------------------------

P31679 ------IPDMGIW-----------------------------------------------

O24723 ------AFGTGFGFF---------------------------------------------

O30513 ------FQF---SSV---------------------------------------------

Q43975 ------SLS---NPT---------------------------------------------

Q51955 ------SLG---NIT---------------------------------------------

Q9I6Q3 ------SLG---QVT---------------------------------------------

P77589 ------GTVSSFNGM---------------------------------------------

P94131 ------FYN---SPD---------------------------------------------

P71369 ------QLT---DPD---------------------------------------------

O34691 ------TAD---SLS---------------------------------------------

P0AGC0 ------QHASNE------------------------------------------------

P27669 ------WLMPFASY----------------------------------------------

P96335 ------WMNPATPEAELANYSAWYENPY--------------------------------

P08194 ------WMNPAG-------------NP---------------------------------

P37948 ------WLNPAG-------------NP---------------------------------

P12681 ------WKSESL------------------------------------------------

Q5M7K3 ------NYLGQTGV----------------------------------------------

Q9WU81 ------NYIGQNGI----------------------------------------------

Q58CV5 ------NHVGQRGI----------------------------------------------

Q7SY29 ------NKIGQNGL----------------------------------------------

P57057 ------STISKMGL----------------------------------------------

Q17QZ3 ------SR-SPNDK----------------------------------------------

Q3TIT8 ------SR-SPNNK----------------------------------------------

Q640L2 ------SH-SPNDK----------------------------------------------

Q5F3N0 ------SR-SPNSK----------------------------------------------

Q09037 ------PVFPGVAEE---------KVKNGV------------------------------

Q06222 ------PVFPGVAEE---------KVKNGV------------------------------

P13865 ------PVYPGVSKKL--------EAKNGV------------------------------

P46499 ------MISPDSDCSS--------QGSRNL------------------------------

P54219 ------PL-AHNIFGL--------IG----------------------------------

Q6NT16 ------PVPILHIKSQ--------LW----------------------------------

Q8R0G7 ------LACARGSI----------VATYIF------------------------------

Q5XGK0 ------LAFADTSL----------VATYVF------------------------------

Q7ZU13 ------IMFAQAST----------VATYVF------------------------------

A2CER7 ------IVLASTSI----------PATYTF------------------------------

Q9D232 ------LILASRTL----------LASYVF------------------------------

Q6ZMD2 ------LVLAPTTL----------LASYVF------------------------------

Q6GPQ3 ------LPWGNQMP----------KIQWTD------------------------------

A5IVG9 -------HIALFTVG--------------C------------------------------

Q5HLK7 -------HIVLFTIG--------------C------------------------------

P46907 -------TIGLYTFG--------------S------------------------------

P10903 -----GGSFMAFFAV--------------F------------------------------

P37593 -----AGSFSAFYLV--------------F------------------------------

P37758 -----SGNFIAFYAV--------------F------------------------------

Q9P3K8 ------GWIQNHGER--------------F------------------------------

Q6FWD4 -----HSHMVTINRK--------------L------------------------------

Q6CPY8 -----ISYIA--------------------------------------------------

P22152 -----EATMFGLTA----------------------------------------------

Q02563 ------FGN---------------------------------------------------

Q496J9 ------FGT---------------------------------------------------

Q63564 ------FGN---------------------------------------------------

Q1JP63 ------ICV---------------------------------------------------

Q2XWK0 ------LCV---------------------------------------------------

Q1LVS8 ------ICT---------------------------------------------------

O08966 ------FIP---------------------------------------------------

O15245 ------FIS---------------------------------------------------

O77504 ------FVP---------------------------------------------------

A7MBE0 ------FIP---------------------------------------------------

Q9R0W2 ------FIP---------------------------------------------------

O02713 ------FIP---------------------------------------------------

Q8MJI6 ------FVP---------------------------------------------------

O88446 ------FLP---------------------------------------------------

O75751 ------FLP---------------------------------------------------

Q9U539 ------FMP---------------------------------------------------

O76082 ------LVP---------------------------------------------------

Q497L8 ------VMP---------------------------------------------------

Q86VW1 ------VIP---------------------------------------------------

Q17QN9 ------VIP---------------------------------------------------

Q95R48 ------IIP---------------------------------------------------

Q9Y267 ------FLPEGEDGLRLKWPRCPA------------------------------------

Q6A4L0 ------FIP---------------------------------------------------

Q9Y226 ------FIP---------------------------------------------------

Q8IVM8 ------FVP---------------------------------------------------

Q66J52 ------SVP---------------------------------------------------

Q91WU2 ------LVS---------------------------------------------------

Q8IZD6 ------FLPEKKDT---------G------------------------------------

Q6DFR1 ------LVPGKTGS---------G------------------------------------

Q6NUB3 ------LVPVKEGS---------G------------------------------------

Q28ES4 ------FLPETNGD----------------------------------------------

A6NKX4 ------AGA---------------------------------------------------

P47185 ------SIGVKCLYP-----------HGQDGPSS--------------------------

P13181 ------SVGVTRLYP-----------HGKSQPSS--------------------------

P23585 ------TVGVTSLYP-----------NGKDQPSS--------------------------

Q92339 ------AVGDRKLI-------------YKNGTSD--------------------------

O74969 ------AVGSRALY-------------HKNGTSN--------------------------

P10870 ------IVGCSL--------------------KT--------------------------

Q12300 ------IVGVSE-------------------GKT--------------------------

P42833 ------TVGTF-------------------GRET--------------------------

P49374 ------GILATYSVPAPGGVNGDDTVTIQIPSEN--------------------------

O74713 ------GILGQYSIPWPD--SGNDSVNIRIPEDN--------------------------

Q9BE72 ------IVNLNIHMNFTNICRS-HNSINQSLDESVIYGPGNLSASNNTLRDHFKGIASHS

Q5J316 ------IVNLNIHMNFTSICRN-HSPINQSLDESVFYGPGNLSASNDTLRESFKGMTFHS

Q32NG5 ------LVSLQLDVNYNNICKV-HTVQNHSLQDSFVYGPVALAKHNESLFEETGTWLEST

Q6NWF1 ------AITMQSRTHVSSLCRGPGNTANFTLFETGDETDIQTNTPLGLYQPQNKLKTNTF

Q0P4G6 ------IVSFTVKMDSHRDC----GSVTGRNMSSGESNVSQLLGIVHAETSTINTLDNSV

Q6GN01 ------IVSFMVELDSHRDC----GSIRSKNTSYGDSNASQLLGIIHAGTPTINTKDNLA

O95528 ------LVSFAVPMDSGPSC----LAVPNATGQTGLPGDSGLL-QDSSLPPIPRTNEDQR

Q8VHD6 ------LVSFAVSLDSGPSC----LATSNASQQVDLPGSSGLL-VRSSLPPVLHTNGDQG

Q3UHK1 ------LGFLLSAQVSPRVTFRPTTPSDQNTTCTGYSYCNECMLDPDCGFCYKINGSAVI

Q96QE2 ------LGFVLSAQVSPRITFKPIAPSGQNATCTRYSYCNECMLDPDCGFCYKMNKSTVI

Q9C757 ------GVFYEAATHAPAISSLETQRFN-NISCPDYKSAMNTNA-WDCMTCLKASSPSCG

Q8VZR6 ------VSFFKQS---------ETS-----------------------------------

P30606 ------IAFHFLGIKFNGADAVVASDGFSS------------------------------

P30605 ------IAFHFLGIKFDGAVAVVVSSGFSS------------------------------

Q10286 ------VAFHFLPKDENGN---YTSGQSNA------------------------------

Q04162 ------VGFGHDQVN---------------------------------------------

Q01440 ------IIGFFIGTRISY------------------------------------------

P11166 ------IALALL--------EQL-------------------------------------

P46896 ------IALTLL--------DQM-------------------------------------

P47843 ------ISLLLK--------DNY-------------------------------------

P14672 ------VALLLL--------ERV-------------------------------------

Q90592 ------VGLVLL--------SQF-------------------------------------

P11168 ------VGLVLL--------NKF-------------------------------------

Q5RB09 ------VTLTLQ--------DRA-------------------------------------

A4ZYQ5 ------VVLLFQ--------NRV-------------------------------------

P22732 ------AALALQ--------DTV-------------------------------------

P58353 ------GALALQ--------DVI-------------------------------------

Q863Y9 ------VALALQ--------DRV-------------------------------------

P43427 ------VALALQ--------NTI-------------------------------------

Q9WV38 ------AALALQ--------NTI-------------------------------------

P15686 ------VVLAIEFAKYGT-DPLP-------------------------------------

Q39525 ------ITLGVEFGQYGT-EDLP-------------------------------------

Q94AZ2 ------IILGVKVTDTST--NLS-------------------------------------

Q10710 ------IILGVKFGD-NQ--QLS-------------------------------------

Q41144 ------ACIGAKFGVDGAPGDLP-------------------------------------

P23586 ------ACIGAKFGVDGTPGELP-------------------------------------

O65413 ------AAIGAKFGVDGTPGVLP-------------------------------------

Q9SX48 ------TLIGMKFGTTGS-GTLT-------------------------------------

Q9LT15 ------SFIGARFGTSGT-GTLT-------------------------------------

Q9FMX3 ------SMIGWKFGFNGE-GNLS-------------------------------------

Q9SBA7 ------IILAKDLDVTGT---LA-------------------------------------

Q9SFG0 ------IILAKDLGVTGT---LG-------------------------------------

Q8L7R8 ------VIVMVA--DVHD-GVIK-------------------------------------

Q93Y91 ------VLLAVTVGATGD-GEMK-------------------------------------

P0AE24 ------YCLMQF----------------------DNGTAS--------------------

P0AEP1 -------TMMHI------------------------GIHS--------------------

P96710 -------TSFYF----------------------E---LT--------------------

P54723 ------ILSIVL----------------------E---GT--------------------

O34718 ------IFSLVL----------------------E---GS--------------------

P46333 ------GVLLTL----------------------G---LS--------------------

O52733 ------IGMKFS----------------------G---GS--------------------

P0AGF4 ------TAF-----------------------------YT--------------------

P21906 ------CCF-----------------------------WF--------------------

P15729 ------VVFGGAT------------------VVNGQPTLT--------------------

Q6AWX0 ------SYYLFF------------------------------------------------

Q93YP9 ------VAFYLKE------------------FVSPDSNMY--------------------

Q94AF9 ------VSFTLQQ------------------M----NVLP--------------------

O04036 ------VSYGFQS------------------F----GILP--------------------

Q94KE0 ------FSFCFRS------------------Y----GMLD--------------------

Q9SCW7 ------LSYYLQN------------------H----GDFQ--------------------

Q4F7G0 ------LSYYLQK------------------N----GEFQ--------------------

Q8LBI9 ------TSFLLKG------------------Q----SLLL--------------------

P93051 ------VSFYLKV------------------H----DMAH--------------------

Q0WQ63 ------NSFLLKA------------------H----GLAL--------------------

Q3ECP7 ------LSFSLQF------------------V----KQLS--------------------

Q9LTP6 ------LSFLFQS------------------Y----GLLE--------------------

Q8GXK5 ------ISFFLQK------------------N----NCWE--------------------

Q9JJZ1 ------TYFKLTQSGPSNSSHVGLLVPI---SAEPADVHL--------------------

P58354 ------TYFKLTEGGPSNSSHVDLPALV---SMEAADTNV--------------------

Q9NY64 ------AYFKLTQGGPGNSSHVAISAPV---SAQPVDASV--------------------

Q9UGQ3 ------LYIHFGPRPLSPNSTAGLESESWGDLAQPLAAPA--------------------

P43562 ------ALFVKYGQDTKPIDGNRSLVWSIGEN----------------------------

Q9FYG3 ------GATSSY------LPHFSALCLS--------------------------------

P36035 ------AFMLRSE-----------------------------------------------

P47186 ------GLGCSST-----------------------------------------------

A6QLI1 ------VVGYSHTRGV--------------------------------------------

Q5W8I7 ------VVGFSHSKGV--------------------------------------------

A4FV52 ------VVGYSHSKGV--------------------------------------------

P34644 ------IVAYTTSDTT--------------------------------------------

Q66GI9 ------GLTTAKQPLV--------------------------------------------

Q9FKV1 ------VLPMFRTENG--------------------------------------------

Q46916 ------FCNYVNVEWM--------------------------------------------

Q91Y77 ------MIPLCSVFGA--------------------------------------------

Q8TF71 ------MIPLCSIFGA--------------------------------------------

A1L1W9 ------MIPLCHVFGG--------------------------------------------

P36021 ------MIPLCRDFGG--------------------------------------------

O35308 ------ISARARSYGT--------------------------------------------

O95907 ------SSARARSYGA--------------------------------------------

Q90632 ------CSARASNYTG--------------------------------------------

O35910 ------TGSTASDYGG--------------------------------------------

O15427 ------AGSTAGDYGG--------------------------------------------

P57788 ------MGSMSVDYGG--------------------------------------------

O15375 ------VCAASGDFWV--------------------------------------------

Q6ZSM3 ------CLPMLQSLPL--------------------------------------------

Q8BGC3 ------CLPMLQTFPL--------------------------------------------

Q503M4 ------FIPLLRTFVW--------------------------------------------

O15403 ------AFTFATEFWG--------------------------------------------

Q8NCK7 ------VGGEESWGGP--------------------------------------------

Q7RTY0 ------AQAPTA------------------------------------------------

Q5R5M4 ------AIPFAKSY-V--------------------------------------------

Q7TM99 ------AIPFAKSY-V--------------------------------------------

Q5ZJU0 ------AIPFARSY-F--------------------------------------------

O15374 ------LAPLATTFPL--------------------------------------------

Q08777 ------WLPFGTNLTN--------------------------------------------

Q08268 ------WLPFGGNTKV--------------------------------------------

P39709 ------SILAAWDVAE--------------------------------------------

P25621 ------VLIRADPLNP--------------------------------------------

O13880 ------LAVANYAPRA--------------------------------------------

P40445 ------LVALPTDTNP--------------------------------------------

P15365 ------LGFAT--NSK--------------------------------------------

Q07904 ------VLISLPLSHK--------------------------------------------

P53322 ------VLGS---QVH--------------------------------------------

P32071 ------WTSS--PDI---------------------------------------------

P38124 ------WTAR----V---------------------------------------------

Q06451 ------ATAY--KHI---------------------------------------------

P53283 ------ATAY--KHI---------------------------------------------

Q9C0R8 ------WSSY--TWV---------------------------------------------

Q9C0Q6 ------WSSY--QRL---------------------------------------------

Q9HF77 ------WTGNYPQHV---------------------------------------------

Q5A0E9 ------VAGDFPKKV---------------------------------------------

Q07824 ------WTGYYPHKI---------------------------------------------

O59698 ------WSGYYTY-V---------------------------------------------

O74829 ------WTGNYPW-I---------------------------------------------

P38776 ------FTCY--KHV---------------------------------------------

Q9HDX4 ------WTVQY--KV---------------------------------------------

P38227 ------YGGRWVDMLLKRYKEKY-------G-ILAPE-----------------------

P38125 ------SGGHLSDYLRSRWIKSHPK-----K-KFPAE-----------------------

P40474 ------IAGRYLNWNYRRRLKYYQNWLGKKRSKLLEEHDNDLNLVQRIIENDPKYTFNIF

O94607 ------LSGFLMKRFG--------------------------------------------

Q9HE13 ------ISGMGIGKLK----NY--------------------------------------

Q04301 ------VTGWVLRNTKINLAHC--------------------------------------

P13090 ------LVGFTIKNVS--------------------------------------------

Q08902 ------TTGFLLSHTP--------------------------------------------

P39886 ------VSGIVISRFG--------------------------------------------

P76269 ------LAGYLIERVH--------------------------------------------

Q8Y9K8 ------FTGRLFDAYG--------------------------------------------

P28873 ----------------------------------------------HWVGPLFGAATTAS

A4WFG6 ----------------------------------------------PEHMAWFILTLGF-

A1JSB0 ----------------------------------------------PEHLSYYILALGF-

A8GKP6 ----------------------------------------------PEHLSYYIMALGF-

P57601 ----------------------------------------------FLNQQYIIISLGF-

Q8K942 ----------------------------------------------YLALKYTIISLGF-

Q89A60 ----------------------------------------------YALLYIIILSLGF-

P76198 ----------------------------------------------PLVCNAGAFVIGFS

O05390 ----------------------------------------------HQYWVLMIIACCYG

O34864 ----------------------------------------------GSLAVVSVIGFIWG

O52717 ----------------------------------------------HNFAMALVPAIALG

O52718 ----------------------------------------------HSFAMALIPAIALG

A9MJT5 -------------------------------------------------NTVTLFGLMLI

A6TG19 -------------------------------------------------QAVTLIGLGMI

A0L190 -----------------------------------------------LGQLFNLLGFGLV

P0AEY8 ----------------------------------------------SHAYLWMTAGLSIY

Q7CP73 ----------------------------------------------PHVWLWSVLGTSLY

P39386 ----------------------------------------------PHVWLWSVLGTSLY

Q68WD6 ----------------------------------------------GLAISMIFVPMMIH

Q4UMJ9 ----------------------------------------------SLAIAMIFVPMMIH

Q1RI77 ----------------------------------------------NIAVIMMFAPMMLH

P32482 ----------------------------------------------LQSVLGFIAPMWLV

P37597 ----------------------------------------------HVSLVEILIPFCVM

P45123 ----------------------------------------------L-GFWPMAIGVAFF

P28246 ----------------------------------------------L-GFWSLVVGVAAF

P31442 ----------------------------------------------VMNVWTLLVPAALF

P37482 ----------------------------------------------LLAIWMIIIGIG--

P76242 ----------------------------------------------I--TWTLLFGFG--

P17583 ----------------------------------------------LPVLWAMVCGLG--

P0C105 ------------------------------------------------AMWSVLAIGLFN

O25788 ------------------------------------------------ALFALTFVGFFN

P11551 ------------------------------------------------GLIALTLCSAFM

P44776 ------------------------------------------------GLYCLILTSGFM

A1A9E1 -----------------------------------------------QAAMAPALFILGA

A9MHY5 -----------------------------------------------QAAMAPALFILGA

A4W8S1 -----------------------------------------------NAAMAPALFILGA

A1JMG4 -----------------------------------------------NYALAPALFILGC

Q0TK80 ----------------------------------------------MWILGVVCLALFGW

O06473 ----------------------------------------------HQTLIITCIVVAGA

Q2FI61 ----------------------------------------------NIHILWIIALAIGL

Q5HQE8 ----------------------------------------------NIYIVWAIGLFIGL

Q49WE5 ----------------------------------------------NITIVWVLAVFIGL

Q4L523 ----------------------------------------------NILIVWIVAIFIGL

P33026 ----------------------------------------------LLGLQLLNAIFIGI

P31436 ----------------------------------------------ELELQILNAIFLGI

Q9S3K0 ----------------------------------------------LLALQLLNAIFIGI

P31675 ----------------------------------------------LMTLQLFNAVFIGI

P31126 ----------------------------------------------LWGMSAAVFTVGEI

Q58955 ----------------------------------------------TFLTILASLTIIAV

P02920 ----------------------------------------------SALEVVILKTLHMF

Q4UK37 ----------------------------------------------DIRIFIITVTVQNF

Q1RKF6 ----------------------------------------------NVKIFIITITTQNF

Q4ULW4 ----------------------------------------------SFEALLIAITIENF

Q1RI01 ----------------------------------------------SFGALLITITIENF

Q68WQ5 ----------------------------------------------NSLILFITIGIESI

Q4UL88 ----------------------------------------------NSLLLFITIGIESI

Q92HQ3 ----------------------------------------------NSLLLFITIGIASI

Q1RIL0 ----------------------------------------------NSTLLFITIGAESI

Q4UMU2 ----------------------------------------------DITSLYIAVFFQEF

Q68W71 ----------------------------------------------DIISLYIAVFCQEF

Q1RHK8 ----------------------------------------------TITTLYIAVFLQEF

Q1LTM2 ----------------------------------------------QVLFLLIGIFYT-G

Q2NTK5 ----------------------------------------------RALFMLIGLFYI-G

P55705 ----------------------------------------------RAIGLFAALYFTWG

Q7Z3Q1 ----------------------------------------------MFLARVPFLFTIVP

Q05B81 ----------------------------------------------MFTGYGLLFLSLVV

P76470 ----------------------------------------------VMAIVALCVAAIGF

P32135 ----------------------------------------------VYLGMACTLGFGAI

P38358 ----------------------------------------------NWLIGLFLIPANLG

A4WAE6 ----------------------------------------------ILSI-FWGVAIMII

A6T8Y8 ----------------------------------------------LLSI-FWGVAIMII

Q6CZ44 ----------------------------------------------TLCI-VWGIAIMAL

Q888L8 ----------------------------------------------TLTV-VWGMAIMCF

Q1IB51 ----------------------------------------------GLSM-VWGMAILSF

A6UZY0 ----------------------------------------------ALSV-LWGMSIMCF

Q4QP52 ----------------------------------------------SLVF-IWGIGISCI

Q9CM87 ----------------------------------------------LLTF-FWGIGIAGI

O25797 ----------------------------------------------LQIF-LWGIGITSL

Q17YP7 ----------------------------------------------LQIF-LWGIGITSL

P77389 ----------------------------------------------ISMV-VWGAATFAV

O31577 ----------------------------------------------VATF-LFGACAFGT

O34367 ----------------------------------------------ITIL-CMGLLAFMN

A1AHK2 ----------------------------------------------GVAI-IWGLTFALI

A9MWE8 ----------------------------------------------VIAI-IWGLAFALV

P23910 ----------------------------------------------IFAF-ICCAGLFAL

P31141 ----------------------------------------------TLVF-VQGALSFAL

P43531 ----------------------------------------------AGML-LFSAGFFAA

Q8K902 ----------------------------------------------LGLV-IFSSGFFAS

P57648 ----------------------------------------------LGLI-IFSGGFFAS

Q89A23 ----------------------------------------------VGLT-LFAAGFFAA

P37498 ----------------------------------------------LGIA-LFTFGFFGG

P0A0J4 ----------------------------------------------LISF-VVFIGFDMI

Q07282 ----------------------------------------------PVLL-LLACGGITL

P02982 ----------------------------------------------PIMV-LLASGGIGM

P02981 ----------------------------------------------PIMI-LLASGGIGM

P70187 ----------------------------------------------AAGA-VAAMSSITF

Q5SR56 ----------------------------------------------AAGT-VAAMSSITF

P77726 ----------------------------------------------VVGVQLFFVAFNLM

Q89AA9 ----------------------------------------------ILGLQIFFIAFIFL

Q8K999 ----------------------------------------------TFALQIFFIAFNIL

P57538 ----------------------------------------------IISLQIFFISFNFL

Q5HIA2 ----------------------------------------------IVIGIFILGFFLST

Q4L3Q4 ----------------------------------------------IILGVFILGFFLST

Q5HRH0 ----------------------------------------------VSIGVLILGFFLST

P0C0L7 ----------------------------------------------IFAGLLMLAVILNC

Q47421 ----------------------------------------------IFLGLLMLAVILNA

P0A2G3 ----------------------------------------------MTLVLLWFSFFFGM

P16482 ----------------------------------------------MLSVLLWLSFIYGM

P0AEX3 ----------------------------------------------AAFGLVMCALLIVS

P76350 ----------------------------------------------IVFFSIMLANIAHD

P41036 ----------------------------------------------PVFAIGGANVWVLG

Q9SYQ1 ----------------------------------------------FMVLYGLVFFFCNF

Q9S735 ----------------------------------------------FMVLYGLIFFFSNF

P76230 ----------------------------------------------ITLIGFFLITFVYM

P38055 ----------------------------------------------ILIYGLVMIFFLYM

Q46909 ----------------------------------------------LTLLLFVLFSTTIS

P31679 ----------------------------------------------LVVMAFAVYAFFSG

O24723 ----------------------------------------------GYFTLLFAIGLSLP

O30513 ----------------------------------------------ILYILIACAGAASI

Q43975 ----------------------------------------------LLALFILCAGIAVN

Q51955 ----------------------------------------------VLATLVLIAGMCVN

Q9I6Q3 ----------------------------------------------LLATLVLLAGMCIN

P77589 ----------------------------------------------LLAGFV--AGLFAT

P94131 ----------------------------------------------NILYLLVIFGFLYG

P71369 ----------------------------------------------IMLLAGAFLGMFVN

O34691 ----------------------------------------------LLLTAGVLLSFFNL

P0AGC0 -----------------------------------------------YIYLASLFALGFL

P27669 -----------------------------------------------VMQAACFFTTGFF

P96335 ----------------------------------------------QLTDFVLMTLIGFL

P08194 -----------------------------------------------TVDMICMIVIGFL

P37948 -----------------------------------------------LVDNIALISIGFL

P12681 -----------------------------------------------LMVTIFAAIVGCL

Q5M7K3 -----------------------------------------------STTVAMLIVCGIL

Q9WU81 -----------------------------------------------TSSIVMLIICGVL

Q58CV5 -----------------------------------------------GISIVMLLICGAL

Q7SY29 -----------------------------------------------PTTVGMLLWCGAL

P57057 -----------------------------------------------EATIAMLLLSGAL

Q17QZ3 -----------------------------------------------SINALLMAVTGFF

Q3TIT8 -----------------------------------------------SINALLMTITGFF

Q640L2 -----------------------------------------------VMNAFIMSITGFF

Q5F3N0 -----------------------------------------------PINAVIMAITGFF

Q09037 ----------------------------------------------ATTGIALFIAAF-E

Q06222 ----------------------------------------------ATTGIALFIAAF-E

P13865 ----------------------------------------------AITGILLFILGF-E

P46499 ----------------------------------------------AIIIINIIGTSFIE

P54219 ----------------------------------------------PNAGLGLAIGM-VD

Q6NT16 ----------------------------------------------LLVLILVVSGLSAG

Q8R0G7 ----------------------------------------------IFIGETLLSMNW-A

Q5XGK0 ----------------------------------------------IFIGETLLSLNW-A

Q7ZU13 ----------------------------------------------IFLGETFLSMNW-A

A2CER7 ----------------------------------------------IAIGETLLSLNW-A

Q9D232 ----------------------------------------------LALGELLLSCNW-A

Q6ZMD2 ----------------------------------------------LGLGELLLSCNW-A

Q6GPQ3 ----------------------------------------------LQNATIHNTTQWTS

A5IVG9 ----------------------------------------------LTISICAGIGNGLI

Q5HLK7 ----------------------------------------------LAISICAGIGNGLI

P46907 ----------------------------------------------LTVAVCSGIGNGTV

P10903 ----------------------------------------------LALFLTAGLGSGST

P37593 ----------------------------------------------MGLFLTAGLGSGST

P37758 ----------------------------------------------MGLFLTAGLGSGST

Q9P3K8 ----------------------------------------------WVASGLVGAGYGAV

Q6FWD4 ----------------------------------------------FYIGIMQGIAYGGL

Q6CPY8 -----------------------------------------------IASAISGFTYGGL

P22152 -----------------------------------------------GLAFFLESCNGAI

Q02563 -------------------------------------------------SESAMIALLCL

Q496J9 -------------------------------------------------SESMMIGMLCL

Q63564 -------------------------------------------------SESAMIGWQCL

Q1JP63 -------------------------------------------------GRNMLTLLLFI

Q2XWK0 -------------------------------------------------GRNVLTVFLFI

Q1LVS8 -------------------------------------------------TMLGFTILLFL

O08966 ----------------------------------------------HELH-WLNVTLACL

O15245 ----------------------------------------------PDLH-WLNIIIMCV

O77504 ----------------------------------------------QDLH-WLTIVLSCV

A7MBE0 ----------------------------------------------HDLP-WLNIMVACV

Q9R0W2 ----------------------------------------------DDLQ-WLKITIACL

O02713 ----------------------------------------------EDPH-WLRITVLCL

Q8MJI6 ----------------------------------------------DDLQ-GLRITVACL

O88446 ----------------------------------------------EGIP-WLRTTVATL

O75751 ----------------------------------------------EGIA-WLRTTVATL

Q9U539 ----------------------------------------------DGYP-WLVASASFI

O76082 ----------------------------------------------PDLY-YLATVLVMV

Q497L8 ----------------------------------------------SDYK-TAKRMVALL

Q86VW1 ----------------------------------------------QKHY-ILGVVTAMV

Q17QN9 ----------------------------------------------KDYH-VWLVVASMA

Q95R48 ----------------------------------------------QRMH-TLIVACAML

Q9Y267 ----------------------------------------------TELK-SMTILVLML

Q6A4L0 ----------------------------------------------GDLP-TVVTVLAVV

Q9Y226 ----------------------------------------------ADLP-VVVTMLAVV

Q8IVM8 ----------------------------------------------QEMQ-TLREVLATL

Q66J52 ----------------------------------------------QDFQ-TVRTAMAVF

Q91WU2 ----------------------------------------------SDTK-SWITALVVI

Q8IZD6 ----------------------------------------------VFAV-VNSHSLSLL

Q6DFR1 ----------------------------------------------PLSV-LNSQTLSLL

Q6NUB3 ----------------------------------------------PLSV-LNSQTLSLL

Q28ES4 -------------------------------------------------L-LNPTVLALF

A6NKX4 ----------------------------------------------QYLPGWTVLFLSVL

P47185 ----------------------------------------------KGAGNAMIVFTCFY

P13181 ----------------------------------------------KGAGNCMIVFTCFY

P23585 ----------------------------------------------KAAGNVMIVFTCLF

Q92339 ----------------------------------------------HRAGSVMIVFSCLF

O74969 ----------------------------------------------TRAGAVMIVMACLF

P10870 ----------------------------------------------VAAAKVMIAFICLF

Q12300 ----------------------------------------------VVASKIMIAFICLF

P42833 ----------------------------------------------DFSNIVLIIVTCCF

P49374 ----------------------------------------------TSAANGVIASSYLF

O74713 ----------------------------------------------KSASKGAIACCYLF

Q9BE72 RSSLMPLRND--VDKRGETTSASLLNAVLSHTE-YQIVTDP-GDVPAFLKWLSLASLLVY

Q5J316 RSSLRPTRND--INGRGETTLASLPNAGLSQTE-YQIVTDS-ADVPTFLKWLSLASLLVY

Q32NG5 KASYHSTSQN--GTKLLHVSAPEDSSFGFTVKE-PKVKSQS-DEIPEYMKWLCLSSLLAF

Q6NWF1 LTSINDTREHWILNHTYNHRTALMETAELSKKDSAKIALQSLHEVSPSLKWISLVSLLVY

Q0P4G6 HQLAMAIRSPSLANSASSNHKDLISQNSTVLPASPELPSNY-----TILNWITLLSMMAF

Q6GN01 HQLAMVIQSPSLSNSAGSKHTASMFPNSTVPPAGPD--SNY-----AILNWITLLSMMAF

O95528 EPILSTAKKTKPHPRSGDPSAPPRLALSSALPGPPLPARGH-----ALLRWTALLCLMVF

Q8VHD6 QLVLSVTER-PIHPVITASLGP---VLNTASPVPTSPILEH-----TLLCWSALVCMMVY

Q3UHK1 DSSCVPVNK-------ASTTEAAWGRCDNETKFKAEGAHWAYSFCPTPYSWTALVGLVLY

Q96QE2 DSSCVPVNK-------ASTNEAAWGRCENETKFKTEDIFWAYNFCPTPYSWTALLGLILY

Q9C757 YCSS-PIGK-------EHPGACWISDDSVKDLCHNENRLWYTRGCPSNFGWFALLGLGLY

Q8VZR6 ------------------------SDGGL-------------------YGWLAVLGLALY

P30606 ------------------------------------------------WGIVIIVFIIVY

P30605 ------------------------------------------------WGIVIIVFIIVF

Q10286 ------------------------------------------------WAIVVLISMIVY

Q04162 ---------------------------------------------------LLLISVVIY

Q01440 ----------------------------------------------SVGGGLFLALLAVF

P11166 ----------------------------------------------PWMSYLSIVAIFGF

P46896 ----------------------------------------------PWMSYLSIVAIFGF

P47843 ----------------------------------------------SWMSFICIGAILVF

P14672 ----------------------------------------------PAMSYVSIVAIFGF

Q90592 ----------------------------------------------AWMSYVSMVAIFLF

P11168 ----------------------------------------------SWMSYVSMIAIFLF

Q5RB09 ----------------------------------------------PWVPYLSIVGILAI

A4ZYQ5 ----------------------------------------------PELSYLGIICVFAY

P22732 ----------------------------------------------SWMPYISIVCVISY

P58353 ----------------------------------------------SWMPYVSIACVISY

Q863Y9 ----------------------------------------------SWMPYISIVCVISY

P43427 ----------------------------------------------SWMPYVSIVCVIVY

Q9WV38 ----------------------------------------------SWMPYISIVCVIVY

P15686 ----------------------------------------------KAVASGILAVICIF

Q39525 ----------------------------------------------HPVSAGVLAVICIF

Q94AZ2 ----------------------------------------------KGFAILVVVMICTY

Q10710 ----------------------------------------------KSFSVLVVIMICLF

Q41144 ----------------------------------------------QWYAVVVVLFICIY

P23586 ----------------------------------------------KWYAIVVVTFICIY

O65413 ----------------------------------------------KWYAIVVVLFICIY

Q9SX48 ----------------------------------------------PATADWILAFICLY

Q9LT15 ----------------------------------------------PATADWILAFICVY

Q9FMX3 ----------------------------------------------GVDADIILALICLY

Q9SBA7 ----------------------------------------------RPQALVVVIFVCVY

Q9SFG0 ----------------------------------------------RPQALVVVIFVCVY

Q8L7R8 ----------------------------------------------EGYGYAVVVLVCVY

Q93Y91 ----------------------------------------------KGYAVTVVVLLCIY

P0AE24 ----------------------------------------------SGLSWLSVGMTMMC

P0AEP1 ----------------------------------------------PSAQYFAIAMLLMF

P96710 ----------------------------------------------SGI--MMIVLILGF

P54723 ----------------------------------------------PALPYVVLSLTILF

O34718 ----------------------------------------------PALPYVVLSLTVTF

P46333 ----------------------------------------------ASTAWMTVVFLGVY

O52733 ----------------------------------------------QTAAIISVIALTVY

P0AGF4 ----------------------------------------------QAPGIVALLSMLFY

P21906 ----------------------------------------------KVGGVLPLASVLLY

P15729 ----------------------------------------------GAAGIIALVTANLY

Q6AWX0 ----------------------------------------------SASPVVAVVALLLY

Q93YP9 ----------------------------------------------NILSMVSVVGVVAM

Q94AF9 ----------------------------------------------ELIPIFVFVNILVY

O04036 ----------------------------------------------ELTPIFTCIGVLGH

Q94KE0 ----------------------------------------------ELTPIFTCIGVVGF

Q9SCW7 ----------------------------------------------EFCSPILIVGLVGY

Q4F7G0 ----------------------------------------------KLCSVMLIVGLVGY

Q8LBI9 ----------------------------------------------EWVPSLAVGGVLIY

P93051 ----------------------------------------------EAVPVLAVVGIMVY

Q0WQ63 ----------------------------------------------DIIPALAVSGVLVY

Q3ECP7 ----------------------------------------------GDASYLALTGVLVY

Q9LTP6 ----------------------------------------------HYTPISTFMGVLVF

Q8GXK5 ----------------------------------------------TGTPIMALISVMVY

Q9JJZ1 ----------------------------------------------G-LAWLAVGSMCLF

P58354 ----------------------------------------------G-LAWLAVGSMCLF

Q9NY64 ----------------------------------------------G-LAWLAVGSMCLF

Q9UGQ3 ----------------------------------------------GYLTLVPLLATMLF

P43562 ----------------------------------------------PFVGGWILTLCFLI

Q9FYG3 ------------------------------------------------VGGTLVFVLTFA

P36035 ----------------------------------------------K-AILGAG-FMLYF

P47186 ----------------------------------------------HGSKMGSGSLLMAV

A6QLI1 -A--------------------------------------------ISFLVLAVGFSGFA

Q5W8I7 -A--------------------------------------------ISFLVLAVGFSGFA

A4FV52 -A--------------------------------------------ISFLVLAVGFSGFA

P34644 -A--------------------------------------------IMALIAAVGMSGFA

Q66GI9 -A--------------------------------------------SAWLSLAVGLKSFS

Q9FKV1 -V--------------------------------------------ILCSSVALGFLALG

Q46916 -I--------------------------------------------IGFMALAFFGKGIG

Q91Y77 -L--------------------------------------------IALCLIMGLFDGCF

Q8TF71 -L--------------------------------------------IAVCLIMGLFDGCF

A1L1W9 -L--------------------------------------------IAVCLLMGLFDGCF

P36021 -L--------------------------------------------IVVCLFLGLCDGFF

O35308 -L--------------------------------------------VAFCIAFGLSYGMV

O95907 -L--------------------------------------------VAFCVAFGLSYGMV

Q90632 -L--------------------------------------------VIFCVFFGISYGMV

O35910 -L--------------------------------------------VVFCIFFGISYGMV

O15427 -L--------------------------------------------VVFCIFFGISYGMV

P57788 -L--------------------------------------------VVFCIFFGISYGMV

O15375 -L--------------------------------------------VGYCLAYSVSMSGI

Q6ZSM3 -L--------------------------------------------VPFSCTFGYFDGAY

Q8BGC3 -L--------------------------------------------VPFSCTFGYFDGAY

Q503M4 -L--------------------------------------------VPFSVLYGYFDGAY

O15403 -L--------------------------------------------MSCSIFFGFMVGTI

Q8NCK7 -L--------------------------------------------LAAAVAYGLSAGSY

Q7RTY0 -L--------------------------------------------VALAVAYGFTSGAL

Q5R5M4 -T--------------------------------------------LALLSGILGFLTGN

Q7TM99 -T--------------------------------------------LAILSGILGFLTGN

Q5ZJU0 -T--------------------------------------------LAVLSGILGFLTGN

O15374 -L--------------------------------------------MTYTICFAIFAGGY

Q08777 -M--------------------------------------------YVISALYGFCSGSV

Q08268 -L--------------------------------------------WAYVCLWGFSTGSI

P39709 -G--------------------------------------------AKWFAFMLQCFGW-

P25621 -K--------------------------------------------VVFSAQYLGGVAY-

O13880 -W--------------------------------------------SRYGGLLMIGFGLG

P40445 -W--------------------------------------------IKYILLTGI-LGLP

P15365 -K--------------------------------------------ARLAGAYLWYISP-

Q07904 -I--------------------------------------------GNLFSLYLLYSGS-

P53322 -G--------------------------------------------VRYFGVYILCMGIY

P32071 ----------------------------------------------NWFVPLIGMALFAV

P38124 ----------------------------------------------HWILPVISEVFFVL

Q06451 ----------------------------------------------IWVGPASAGLAFGF

P53283 ----------------------------------------------IWVGPASAGLAFGF

Q9C0R8 ----------------------------------------------SWAGPCFSGLAAGF
[truncated: 183,596 more chars]
