# Supplementary material for: Rational Mutational Analysis of a Multidrug MFS Transporter CaMdr1p of Candida albicans by Employing a Membrane Environment Based Computational Approach
Source: PLoS Comput Biol. 2009 Dec 24;5(12):e1000624. doi: 10.1371/journal.pcbi.1000624 (PMC2789324; doi:10.1371/journal.pcbi.1000624)
Supplement: Table S1 — REM, RE and conservation scores for all the positions of the MSA of 342 MFS sequences. (2.01 MB DOC) [file pcbi.1000624.s002.doc]

| **Alignment position** | **MFA** | **RE_M_** | **CaMdr1p**  **Position** | **CaMdr1p**  **Residue** | **RE** | **Conservation** |
| --- | --- | --- | --- | --- | --- | --- |
| 1 | gap | 0.0527 | - | - | 0.0547 | 0.0 |
| 2 | gap | 0.0259 | - | - | 0.0263 | 0.0 |
| 3 | gap | 0.0626 | - | - | 0.0642 | 0.0 |
| 4 | gap | 0.0368 | - | - | 0.0355 | 0.0 |
| 5 | gap | 0.0498 | - | - | 0.0488 | 0.0 |
| 6 | gap | 0.0597 | 1 | M | 0.0627 | 0.0 |
| 7 | gap | 0.0463 | 2 | H | 0.0492 | 0.0 |
| 8 | gap | 0.1108 | 3 | Y | 0.1147 | 0.0 |
| 9 | gap | 0.0672 | 4 | R | 0.0672 | 0.0 |
| 10 | gap | 0.0759 | 5 | F | 0.0826 | 0.0 |
| 11 | gap | 0.0295 | 6 | L | 0.0293 | 0.0 |
| 12 | gap | 0.0341 | 7 | R | 0.0336 | 0.0 |
| 13 | gap | 0.0658 | 8 | D | 0.0666 | 0.0 |
| 14 | gap | 0.0341 | 9 | S | 0.0343 | 0.0 |
| 15 | gap | 0.0513 | 10 | F | 0.0512 | 0.0 |
| 16 | gap | 0.0351 | 11 | V | 0.0316 | 0.0 |
| 17 | gap | 0.0262 | 12 | G | 0.0253 | 0.0 |
| 18 | gap | 0.0537 | 13 | R | 0.0485 | 0.0 |
| 19 | gap | 0.0590 | 14 | V | 0.0589 | 0.0 |
| 20 | gap | 0.0435 | 15 | T | 0.0429 | 0.0 |
| 21 | gap | 0.0353 | 16 | Y | 0.0365 | 0.0 |
| 22 | gap | 0.0408 | 17 | H | 0.0427 | 0.0 |
| 23 | gap | 0.0678 | 18 | L | 0.0708 | 0.0 |
| 24 | gap | 0.0448 | 19 | S | 0.0435 | 0.0 |
| 25 | gap | 0.0781 | 20 | K | 0.0735 | 0.0 |
| 26 | gap | 0.0169 | 21 | H | 0.0198 | 0.0 |
| 27 | gap | 0.0519 | 22 | K | 0.0515 | 0.0 |
| 28 | gap | 0.0305 | 23 | Y | 0.0267 | 0.0 |
| 29 | gap | 0.0538 | 24 | F | 0.0575 | 0.0 |
| 30 | gap | 0.0569 | 25 | A | 0.0573 | 0.0 |
| 31 | gap | 0.0586 | 26 | H | 0.0562 | 0.0 |
| 32 | gap | 0.0427 | - | - | 0.0454 | 0.0 |
| 33 | gap | 0.0391 | - | - | 0.0390 | 0.0 |
| 34 | gap | 0.0433 | - | - | 0.0392 | 0.0 |
| 35 | gap | 0.0494 | - | - | 0.0477 | 0.0 |
| 36 | gap | 0.0489 | - | - | 0.0503 | 0.0 |
| 37 | gap | 0.0289 | - | - | 0.0275 | 0.0 |
| 38 | gap | 0.0457 | - | - | 0.0417 | 0.0 |
| 39 | gap | 0.0598 | - | - | 0.0554 | 0.0 |
| 40 | gap | 0.0668 | - | - | 0.0691 | 0.0 |
| 41 | gap | 0.0359 | - | - | 0.0309 | 0.0 |
| 42 | gap | 0.0460 | - | - | 0.0510 | 0.0 |
| 43 | gap | 0.0705 | - | - | 0.0644 | 0.0 |
| 44 | gap | 0.0703 | - | - | 0.0695 | 0.0 |
| 45 | gap | 0.0621 | - | - | 0.0579 | 0.0 |
| 46 | gap | 0.0485 | - | - | 0.0528 | 0.0 |
| 47 | gap | 0.0633 | - | - | 0.0723 | 0.0 |
| 48 | gap | 0.0748 | - | - | 0.0705 | 0.0 |
| 49 | gap | 0.0884 | - | - | 0.0945 | 0.0 |
| 50 | gap | 0.0996 | - | - | 0.0998 | 0.0 |
| 51 | gap | 0.0647 | - | - | 0.0666 | 0.0 |
| 52 | gap | 0.0881 | - | - | 0.0914 | 0.0 |
| 53 | gap | 0.1085 | - | - | 0.0978 | 0.0 |
| 54 | gap | 0.0823 | - | - | 0.0771 | 0.0 |
| 55 | gap | 0.0894 | - | - | 0.0882 | 0.0 |
| 56 | gap | 0.0613 | - | - | 0.0617 | 0.0 |
| 57 | gap | 0.0730 | - | - | 0.0659 | 0.0 |
| 58 | gap | 0.0881 | - | - | 0.0826 | 0.0 |
| 59 | gap | 0.0748 | - | - | 0.0716 | 0.0 |
| 60 | gap | 0.1214 | - | - | 0.1185 | 0.0 |
| 61 | gap | 0.1102 | - | - | 0.1028 | 0.0 |
| 62 | gap | 0.0991 | - | - | 0.1121 | 0.0 |
| 63 | gap | 0.0344 | - | - | 0.0344 | 0.0 |
| 64 | gap | 0.0260 | - | - | 0.0250 | 0.0 |
| 65 | gap | 0.0194 | - | - | 0.0189 | 0.0 |
| 66 | gap | 0.0235 | - | - | 0.0234 | 0.0 |
| 67 | gap | 0.0197 | - | - | 0.0194 | 0.0 |
| 68 | gap | 0.0224 | - | - | 0.0224 | 0.0 |
| 69 | gap | 0.0301 | - | - | 0.0284 | 0.0 |
| 70 | gap | 0.0832 | - | - | 0.0836 | 0.0 |
| 71 | gap | 0.0720 | - | - | 0.0742 | 0.0 |
| 72 | gap | 0.0863 | - | - | 0.0996 | 0.0 |
| 73 | gap | 0.1786 | 27 | P | 0.1767 | 0.0 |
| 74 | gap | 0.0600 | 28 | E | 0.0554 | 0.0 |
| 75 | gap | 0.0619 | 29 | E | 0.0559 | 0.0 |
| 76 | gap | 0.0657 | 30 | A | 0.0664 | 0.0 |
| 77 | gap | 0.0633 | 31 | K | 0.0652 | 0.0 |
| 78 | gap | 0.0302 | 32 | D | 0.0424 | 0.0 |
| 79 | gap | 0.0672 | 33 | Y | 0.0708 | 0.0 |
| 80 | gap | 0.0818 | 34 | I | 0.0790 | 0.0 |
| 81 | gap | 0.0824 | 35 | V | 0.0718 | 0.0 |
| 82 | gap | 0.0671 | 36 | P | 0.0639 | 0.0 |
| 83 | gap | 0.0723 | 37 | E | 0.0662 | 0.0 |
| 84 | gap | 0.0554 | 38 | K | 0.0560 | 0.0 |
| 85 | gap | 0.1991 | 39 | Y | 0.2130 | 0.0 |
| 86 | gap | 0.0654 | 40 | L | 0.0596 | 0.0 |
| 87 | gap | 0.0572 | 41 | A | 0.0602 | 0.0 |
| 88 | gap | 0.0622 | 42 | D | 0.0657 | 0.0 |
| 89 | gap | 0.0765 | 43 | Y | 0.0673 | 0.0 |
| 90 | gap | 0.1286 | 44 | K | 0.1396 | 0.0 |
| 91 | gap | 0.1214 | 45 | P | 0.1174 | 0.0 |
| 92 | gap | 0.0717 | 46 | T | 0.0722 | 0.0 |
| 93 | gap | 0.0565 | 47 | L | 0.0589 | 0.0 |
| 94 | gap | 0.0940 | 48 | A | 0.0975 | 0.0 |
| 95 | gap | 0.0720 | 49 | D | 0.0715 | 0.0 |
| 96 | gap | 0.1080 | 50 | D | 0.1002 | 0.0 |
| 97 | gap | 0.1220 | 51 | T | 0.1361 | 0.0 |
| 98 | gap | 0.1007 | 52 | S | 0.1033 | 0.0 |
| 99 | gap | 0.0876 | 53 | I | 0.0883 | 0.0 |
| 100 | gap | 0.0819 | 54 | N | 0.0833 | 0.0 |
| 101 | gap | 0.1109 | 55 | F | 0.1020 | 0.0 |
| 102 | gap | 0.1516 | 56 | E | 0.1574 | 0.0 |
| 103 | gap | 0.0878 | 57 | K | 0.0765 | 0.0 |
| 104 | gap | 0.1693 | 58 | E | 0.1629 | 0.0 |
| 105 | gap | 0.1382 | 59 | E | 0.1304 | 0.0 |
| 106 | gap | 0.0540 | 60 | I | 0.0639 | 0.0 |
| 107 | gap | 0.1670 | 61 | D | 0.1445 | 0.0 |
| 108 | gap | 0.1243 | 62 | N | 0.1167 | 0.0 |
| 109 | gap | 0.0866 | 63 | Q | 0.0828 | 0.0 |
| 110 | gap | 0.1025 | 64 | G | 0.1084 | 0.0 |
| 111 | gap | 0.1246 | 65 | E | 0.1305 | 0.0 |
| 112 | gap | 0.0796 | 66 | P | 0.0734 | 0.0 |
| 113 | gap | 0.0711 | 67 | N | 0.0748 | 0.0 |
| 114 | gap | 0.0566 | 68 | S | 0.0507 | 0.0 |
| 115 | gap | 0.0847 | 69 | S | 0.0886 | 0.0 |
| 116 | gap | 0.1737 | 70 | Q | 0.1886 | 0.0 |
| 117 | gap | 0.0581 | 71 | S | 0.0625 | 0.0 |
| 118 | gap | 0.1378 | 72 | S | 0.1465 | 0.0 |
| 119 | gap | 0.1523 | 73 | S | 0.1547 | 0.0 |
| 120 | gap | 0.1201 | 74 | S | 0.1324 | 0.0 |
| 121 | gap | 0.1079 | 75 | N | 0.1347 | 0.0 |
| 122 | S | 0.2049 | 76 | N | 0.2114 | 0.0 |
| 123 | gap | 0.1520 | 77 | T | 0.1503 | 0.0 |
| 124 | L | 0.0977 | 78 | I | 0.1291 | 0.0 |
| 125 | V | 0.1065 | 79 | V | 0.1079 | 0.0 |
| 126 | gap | 0.2314 | 80 | D | 0.2277 | 0.0 |
| 127 | gap | 0.0713 | 81 | N | 0.0818 | 0.0 |
| 128 | gap | 0.1080 | 82 | N | 0.1058 | 0.0 |
| 129 | gap | 0.0456 | 83 | N | 0.0428 | 0.0 |
| 130 | gap | 0.0599 | 84 | N | 0.0590 | 0.0 |
| 131 | gap | 0.0728 | 85 | N | 0.0699 | 0.0 |
| 132 | gap | 0.1888 | 86 | N | 0.1939 | 0.0 |
| 133 | gap | 0.1256 | 87 | D | 0.1246 | 0.0 |
| 134 | gap | 0.1100 | 88 | N | 0.1088 | 0.0 |
| 135 | gap | 0.1942 | 89 | D | 0.1898 | 0.0 |
| 136 | gap | 0.0885 | 90 | V | 0.0898 | 0.0 |
| 137 | gap | 0.0580 | 91 | D | 0.0547 | 0.0 |
| 138 | gap | 0.0756 | 92 | G | 0.0698 | 0.0 |
| 139 | gap | 0.0722 | - | - | 0.0735 | 0.0 |
| 140 | gap | 0.0745 | - | - | 0.0706 | 0.0 |
| 141 | gap | 0.0602 | - | - | 0.0608 | 0.0 |
| 142 | gap | 0.0455 | - | - | 0.0473 | 0.0 |
| 143 | gap | 0.0424 | - | - | 0.0440 | 0.0 |
| 144 | gap | 0.0674 | - | - | 0.0678 | 0.0 |
| 145 | gap | 0.0759 | - | - | 0.0752 | 0.0 |
| 146 | gap | 0.2038 | - | - | 0.1878 | 0.0 |
| 147 | gap | 0.0530 | - | - | 0.0548 | 0.0 |
| 148 | gap | 0.1022 | - | - | 0.0969 | 0.0 |
| 149 | gap | 0.1130 | - | - | 0.1188 | 0.0 |
| 150 | gap | 0.0573 | - | - | 0.0563 | 0.0 |
| 151 | gap | 0.0784 | - | - | 0.0768 | 0.0 |
| 152 | gap | 0.0788 | - | - | 0.0831 | 0.0 |
| 153 | gap | 0.0863 | - | - | 0.0828 | 0.0 |
| 154 | gap | 0.0741 | - | - | 0.0855 | 0.0 |
| 155 | gap | 0.0862 | - | - | 0.0871 | 0.0 |
| 156 | gap | 0.0929 | 93 | D | 0.0855 | 0.0 |
| 157 | gap | 0.0610 | 94 | K | 0.0594 | 0.0 |
| 158 | gap | 0.0506 | 95 | I | 0.0494 | 0.0 |
| 159 | gap | 0.0372 | 96 | V | 0.0362 | 0.0 |
| 160 | gap | 0.0927 | 97 | V | 0.0901 | 0.0 |
| 161 | gap | 0.0735 | 98 | T | 0.0710 | 0.0 |
| 162 | gap | 0.1227 | 99 | W | 0.1424 | 0.0 |
| 163 | gap | 0.0773 | 100 | D | 0.0714 | 0.0 |
| 164 | gap | 0.0345 | 101 | G | 0.0384 | 0.0 |
| 165 | gap | 0.0750 | 102 | D | 0.0746 | 0.0 |
| 166 | gap | 0.1142 | - | - | 0.1045 | 0.0 |
| 167 | gap | 0.0915 | 103 | D | 0.0848 | 0.0 |
| 168 | gap | 0.1940 | 104 | D | 0.1804 | 0.0 |
| 169 | gap | 0.1447 | 105 | P | 0.1449 | 0.0 |
| 170 | gap | 0.0749 | 106 | E | 0.0750 | 0.0 |
| 171 | gap | 0.0659 | 107 | N | 0.0634 | 0.0 |
| 172 | gap | 0.0424 | - | - | 0.0458 | 0.0 |
| 173 | gap | 0.1026 | 108 | P | 0.1026 | 0.0 |
| 174 | gap | 0.0528 | 109 | Q | 0.0576 | 0.0 |
| 175 | gap | 0.1450 | 110 | N | 0.1462 | 0.0 |
| 176 | gap | 0.1668 | 111 | W | 0.1801 | 0.0 |
| 177 | gap | 0.1833 | 112 | P | 0.2293 | 0.0 |
| 178 | gap | 0.0830 | 113 | T | 0.0913 | 0.0 |
| 179 | gap | 0.1675 | 114 | L | 0.1680 | 0.0 |
| 180 | gap | 0.2226 | 115 | Q | 0.2923 | 0.0 |
| 181 | A | 0.3067 | 116 | K | 0.3146 | 0.0 |
| 182 | L | 0.4313 | 117 | A | 0.4638 | 0.0 |
| 183 | G | 0.3742 | 118 | F | 0.4030 | 0.0 |
| 184 | G | 0.1643 | 119 | F | 0.4658 | 0.0 |
| 185 | gap | 0.0731 | 120 | I | 0.1753 | 0.0 |
| 186 | L | 0.3637 | 121 | F | 0.7398 | 4.0 |
| 187 | L | 0.2384 | 122 | Q | 0.4586 | 4.0 |
| 188 | F | 0.3293 | 123 | I | 0.4437 | 2.0 |
| 189 | G | 0.4936 | 124 | S | 0.7810 | 4.0 |
| 190 | Y | 0.6568 | 125 | F | 0.9086 | 5.0 |
| 191 | D | 0.5750 | 126 | L | 0.3393 | 2.0 |
| 192 | L | 0.1732 | 127 | T | 0.4163 | 4.0 |
| 193 | G | 0.2155 | 128 | T | 0.3604 | 2.0 |
| 194 | V | 0.1845 | 129 | S | 0.2806 | 4.0 |
| 195 | I | 0.1376 | 130 | V | 0.2823 | 0.0 |
| 196 | S | 0.2909 | 131 | Y | 0.3080 | 0.0 |
| 197 | G | 0.2188 | 132 | M | 0.3265 | 0.0 |
| 198 | gap | 0.2039 | 133 | G | 0.1540 | 0.0 |
| 199 | gap | 0.2108 | 134 | S | 0.2399 | 0.0 |
| 200 | V | 0.2271 | 135 | A | 0.4339 | 0.0 |
| 201 | I | 0.1728 | 136 | V | 0.2657 | 0.0 |
| 202 | L | 0.1326 | 137 | Y | 0.2988 | 0.0 |
| 203 | L | 0.1901 | 138 | T | 0.3386 | 0.0 |
| 204 | I | 0.3175 | 139 | P | 0.1125 | 0.0 |
| 205 | V | 0.3713 | 140 | G | 0.0919 | 0.0 |
| 206 | gap | 0.3162 | 141 | I | 0.1898 | 0.0 |
| 207 | gap | 0.1939 | 142 | E | 0.2090 | 0.0 |
| 208 | gap | 0.1450 | 143 | E | 0.1280 | 0.0 |
| 209 | gap | 0.2111 | 144 | L | 0.1761 | 0.0 |
| 210 | gap | 0.1671 | 145 | M | 0.1890 | 0.0 |
| 211 | gap | 0.2797 | - | - | 0.3143 | 0.0 |
| 212 | gap | 0.1177 | - | - | 0.1137 | 0.0 |
| 213 | gap | 0.0723 | - | - | 0.0679 | 0.0 |
| 214 | gap | 0.0455 | - | - | 0.0486 | 0.0 |
| 215 | gap | 0.0201 | - | - | 0.0178 | 0.0 |
| 216 | gap | 0.0368 | - | - | 0.0379 | 0.0 |
| 217 | gap | 0.0550 | - | - | 0.0562 | 0.0 |
| 218 | gap | 0.0264 | - | - | 0.0265 | 0.0 |
| 219 | gap | 0.0546 | - | - | 0.0560 | 0.0 |
| 220 | gap | 0.0541 | - | - | 0.0504 | 0.0 |
| 221 | gap | 0.0325 | - | - | 0.0341 | 0.0 |
| 222 | gap | 0.0427 | - | - | 0.0403 | 0.0 |
| 223 | gap | 0.0454 | - | - | 0.0524 | 0.0 |
| 224 | gap | 0.0326 | - | - | 0.0305 | 0.0 |
| 225 | gap | 0.0693 | - | - | 0.0683 | 0.0 |
| 226 | gap | 0.0211 | - | - | 0.0204 | 0.0 |
| 227 | gap | 0.0233 | - | - | 0.0229 | 0.0 |
| 228 | gap | 0.0230 | - | - | 0.0218 | 0.0 |
| 229 | gap | 0.0213 | - | - | 0.0223 | 0.0 |
| 230 | gap | 0.0215 | - | - | 0.0204 | 0.0 |
| 231 | gap | 0.0179 | - | - | 0.0174 | 0.0 |
| 232 | gap | 0.0241 | - | - | 0.0245 | 0.0 |
| 233 | gap | 0.0183 | - | - | 0.0183 | 0.0 |
| 234 | gap | 0.0171 | - | - | 0.0174 | 0.0 |
| 235 | gap | 0.0137 | - | - | 0.0139 | 0.0 |
| 236 | gap | 0.0233 | - | - | 0.0259 | 0.0 |
| 237 | gap | 0.0203 | - | - | 0.0200 | 0.0 |
| 238 | gap | 0.0174 | - | - | 0.0170 | 0.0 |
| 239 | gap | 0.0160 | - | - | 0.0154 | 0.0 |
| 240 | gap | 0.0243 | - | - | 0.0250 | 0.0 |
| 241 | gap | 0.0167 | - | - | 0.0164 | 0.0 |
| 242 | gap | 0.1474 | - | - | 0.1441 | 0.0 |
| 243 | gap | 0.1224 | - | - | 0.1383 | 0.0 |
| 244 | gap | 0.0507 | - | - | 0.0500 | 0.0 |
| 245 | gap | 0.0586 | - | - | 0.0580 | 0.0 |
| 246 | gap | 0.1026 | - | - | 0.0986 | 0.0 |
| 247 | gap | 0.0677 | - | - | 0.0634 | 0.0 |
| 248 | gap | 0.0767 | - | - | 0.0742 | 0.0 |
| 249 | gap | 0.0315 | - | - | 0.0321 | 0.0 |
| 250 | gap | 0.0464 | - | - | 0.0424 | 0.0 |
| 251 | gap | 0.0320 | - | - | 0.0340 | 0.0 |
| 252 | gap | 0.0276 | - | - | 0.0264 | 0.0 |
| 253 | gap | 0.1033 | - | - | 0.0996 | 0.0 |
| 254 | gap | 0.1024 | - | - | 0.0980 | 0.0 |
| 255 | gap | 0.0974 | - | - | 0.1004 | 0.0 |
| 256 | gap | 0.0665 | - | - | 0.0647 | 0.0 |
| 257 | gap | 0.0949 | - | - | 0.1110 | 0.0 |
| 258 | gap | 0.0952 | - | - | 0.0903 | 0.0 |
| 259 | gap | 0.0545 | - | - | 0.0533 | 0.0 |
| 260 | gap | 0.0646 | - | - | 0.0729 | 0.0 |
| 261 | gap | 0.0487 | - | - | 0.0469 | 0.0 |
| 262 | gap | 0.0987 | - | - | 0.1140 | 0.0 |
| 263 | gap | 0.1134 | 146 | H | 0.1107 | 0.0 |
| 264 | gap | 0.1463 | 147 | D | 0.1296 | 0.0 |
| 265 | gap | 0.2420 | 148 | F | 0.2171 | 0.0 |
| 266 | gap | 0.1800 | 149 | G | 0.2126 | 0.0 |
| 267 | gap | 0.2817 | 150 | I | 0.2619 | 0.0 |
| 268 | L | 0.1826 | 151 | G | 0.2723 | 0.0 |
| 269 | S | 0.6291 | 152 | R | 0.3601 | 0.0 |
| 270 | T | 0.3352 | 153 | V | 0.1744 | 0.0 |
| 271 | S | 0.2458 | 154 | V | 0.3176 | 0.0 |
| 272 | L | 0.5458 | 155 | A | 0.3088 | 0.0 |
| 273 | T | 0.3513 | 156 | T | 0.4245 | 2.0 |
| 274 | G | 0.5540 | 157 | L | 0.5801 | 3.0 |
| 275 | L | 0.4197 | - | - | 0.7376 | 0.0 |
| 276 | I | 0.2776 | 158 | P | 0.5780 | 4.0 |
| 277 | V | 0.2944 | 159 | L | 0.3969 | 2.0 |
| 278 | S | 1.0227 | 160 | T | 1.1577 | 7.0 |
| 279 | L | 0.3437 | 161 | L | 0.6901 | 4.0 |
| 280 | F | 0.5013 | 162 | F | 0.7408 | 3.0 |
| 281 | L | 0.2137 | 163 | V | 0.4364 | 0.0 |
| 282 | L | 0.3486 | 164 | I | 0.7399 | 5.0 |
| 283 | G | 0.7101 | 165 | G | 1.0571 | 5.0 |
| 284 | A | 0.2283 | 166 | Y | 0.4160 | 2.0 |
| 285 | L | 0.2799 | 167 | G | 0.5997 | 3.0 |
| 286 | L | 0.4595 | 168 | V | 0.8502 | 4.0 |
| 287 | G | 0.6386 | 169 | G | 0.9724 | 4.0 |
| 288 | S | 0.4861 | 170 | P | 0.4694 | 4.0 |
| 289 | L | 0.4689 | 171 | L | 0.7406 | 4.0 |
| 290 | L | 0.3595 | 172 | V | 0.6876 | 3.0 |
| 291 | gap | 0.0796 | - | - | 0.0701 | 0.0 |
| 292 | F | 0.2032 | 173 | F | 0.5323 | 4.0 |
| 293 | G | 1.4191 | 174 | S | 1.4295 | 6.0 |
| 294 | P | 0.3367 | 175 | P | 0.4038 | 1.0 |
| 295 | L | 0.9306 | 176 | M | 0.9554 | 7.0 |
| 296 | S | 0.5248 | 177 | S | 0.7134 | 4.0 |
| 297 | D | 1.2780 | 178 | E | 1.1745 | 4.0 |
| 298 | gap | 0.4896 | 179 | N | 0.4673 | 0.0 |
| 299 | gap | 0.1142 | 180 | A | 0.1694 | 0.0 |
| 300 | gap | 0.5603 | 181 | I | 0.3886 | 0.0 |
| 301 | gap | 0.0148 | - | - | 0.0110 | 0.0 |
| 302 | gap | 0.0057 | - | - | 0.0068 | 0.0 |
| 303 | gap | 0.0066 | - | - | 0.0078 | 0.0 |
| 304 | F | 0.4986 | 182 | F | 0.7779 | 1.0 |
| 305 | G | 1.7539 | 183 | G | 1.7373 | 3.0 |
| 306 | R | 1.4174 | 184 | R | 1.3519 | 2.0 |
| 307 | R | 2.3729 | 185 | T | 1.5099 | 3.0 |
| 308 | gap | 0.0126 | - | - | 0.0079 | 0.0 |
| 309 | gap | 0.0298 | - | - | 0.0266 | 0.0 |
| 310 | P | 0.6645 | 186 | S | 0.3767 | 1.0 |
| 311 | V | 0.2229 | 187 | I | 0.4210 | 5.0 |
| 312 | L | 0.5864 | 188 | Y | 0.9650 | 6.0 |
| 313 | L | 0.2373 | 189 | I | 0.5254 | 0.0 |
| 314 | L | 0.2103 | 190 | I | 0.5657 | 4.0 |
| 315 | G | 0.4953 | 191 | T | 0.7305 | 5.0 |
| 316 | L | 0.1616 | 192 | L | 0.4039 | 3.0 |
| 317 | L | 0.3513 | 193 | F | 0.7296 | 4.0 |
| 318 | L | 0.3173 | 194 | L | 0.6945 | 4.0 |
| 319 | F | 0.3995 | 195 | F | 0.7284 | 4.0 |
| 320 | A | 0.2915 | 196 | V | 0.6138 | 4.0 |
| 321 | L | 0.3543 | 197 | I | 0.7226 | 4.0 |
| 322 | G | 0.4019 | 198 | L | 0.6928 | 0.0 |
| 323 | S | 0.3374 | 199 | Q | 0.4665 | 2.0 |
| 324 | L | 0.3451 | 200 | I | 0.7124 | 6.0 |
| 325 | L | 0.2677 | 201 | P | 0.6115 | 4.0 |
| 326 | S | 0.3064 | 202 | T | 0.5490 | 4.0 |
| 327 | A | 0.5580 | 203 | A | 0.5629 | 3.0 |
| 328 | F | 0.6172 | 204 | L | 0.6904 | 4.0 |
| 329 | A | 0.7562 | 205 | V | 0.7690 | 4.0 |
| 330 | gap | 0.1528 | 206 | N | 0.1645 | 0.0 |
| 331 | gap | 0.1686 | 207 | N | 0.1907 | 0.0 |
| 332 | gap | 0.2075 | 208 | I | 0.2561 | 0.0 |
| 333 | gap | 0.2403 | - | - | 0.2385 | 0.0 |
| 334 | gap | 0.3157 | - | - | 0.3064 | 0.0 |
| 335 | gap | 0.2842 | - | - | 0.3369 | 0.0 |
| 336 | gap | 0.1817 | - | - | 0.2196 | 0.0 |
| 337 | gap | 0.2869 | - | - | 0.3381 | 0.0 |
| 338 | gap | 0.0564 | - | - | 0.0776 | 0.0 |
| 339 | gap | 0.0137 | - | - | 0.0139 | 0.0 |
| 340 | gap | 0.0153 | - | - | 0.0160 | 0.0 |
| 341 | gap | 0.0208 | - | - | 0.0208 | 0.0 |
| 342 | gap | 0.0237 | - | - | 0.0228 | 0.0 |
| 343 | gap | 0.0166 | - | - | 0.0167 | 0.0 |
| 344 | gap | 0.0230 | - | - | 0.0237 | 0.0 |
| 345 | gap | 0.0283 | - | - | 0.0265 | 0.0 |
| 346 | gap | 0.0274 | - | - | 0.0274 | 0.0 |
| 347 | gap | 0.0150 | - | - | 0.0144 | 0.0 |
| 348 | gap | 0.0149 | - | - | 0.0147 | 0.0 |
| 349 | gap | 0.0112 | - | - | 0.0113 | 0.0 |
| 350 | gap | 0.0114 | - | - | 0.0112 | 0.0 |
| 351 | gap | 0.0126 | - | - | 0.0123 | 0.0 |
| 352 | gap | 0.0068 | - | - | 0.0068 | 0.0 |
| 353 | gap | 0.0679 | - | - | 0.0703 | 0.0 |
| 354 | gap | 0.0847 | - | - | 0.0890 | 0.0 |
| 355 | gap | 0.2471 | 209 | A | 0.3178 | 0.0 |
| 356 | gap | 0.2198 | 210 | G | 0.2314 | 0.0 |
| 357 | L | 0.7231 | 211 | L | 1.1398 | 6.0 |
| 358 | L | 0.4165 | 212 | C | 0.7470 | 6.0 |
| 359 | V | 0.3173 | 213 | I | 0.6547 | 4.0 |
| 360 | G | 0.2651 | 214 | L | 0.5923 | 4.0 |
| 361 | R | 1.9726 | 215 | R | 1.5762 | 3.0 |
| 362 | F | 0.4417 | 216 | F | 0.8928 | 6.0 |
| 363 | L | 0.5539 | 217 | L | 0.9865 | 6.0 |
| 364 | L | 0.3806 | 218 | G | 0.4527 | 4.0 |
| 365 | G | 1.4760 | 219 | G | 1.8514 | 10.0 |
| 366 | L | 0.3474 | 220 | F | 0.7528 | 4.0 |
| 367 | G | 0.5378 | 221 | F | 0.8838 | 4.0 |
| 368 | V | 0.2556 | 222 | A | 0.2991 | 0.0 |
| 369 | G | 1.0453 | 223 | S | 1.2925 | 8.0 |
| 370 | G | 0.1789 | 224 | P | 0.5037 | 4.0 |
| 371 | A | 0.1513 | 225 | C | 0.3348 | 2.0 |
| 372 | S | 0.5780 | 226 | L | 0.7691 | 0.0 |
| 373 | P | 0.4079 | 227 | A | 0.3667 | 0.0 |
| 374 | V | 0.2771 | 228 | T | 0.4500 | 3.0 |
| 375 | V | 0.4293 | 229 | G | 0.6560 | 5.0 |
| 376 | P | 0.4102 | 230 | G | 0.5150 | 3.0 |
| 377 | V | 0.2540 | 231 | A | 0.4172 | 5.0 |
| 378 | Y | 0.5992 | 232 | S | 0.8077 | 4.0 |
| 379 | L | 0.2891 | 233 | V | 0.6154 | 4.0 |
| 380 | A | 0.4181 | 234 | A | 0.5637 | 3.0 |
| 381 | E | 1.7278 | 235 | D | 0.7461 | 3.0 |
| 382 | I | 0.4462 | 236 | V | 0.5302 | 1.0 |
| 383 | A | 0.5098 | 237 | V | 0.5465 | 2.0 |
| 384 | P | 0.7424 | 238 | K | 0.7581 | 1.0 |
| 385 | K | 0.3088 | 239 | F | 0.3106 | 1.0 |
| 386 | K | 0.4140 | 240 | W | 0.3817 | 1.0 |
| 387 | K | 0.6062 | 241 | N | 0.2271 | 0.0 |
| 388 | R | 2.0881 | 242 | L | 1.4744 | 0.0 |
| 389 | gap | 0.3997 | 243 | P | 0.3538 | 0.0 |
| 390 | G | 0.4376 | 244 | V | 0.4021 | 2.0 |
| 391 | A | 0.2509 | 245 | G | 0.3242 | 0.0 |
| 392 | L | 0.2440 | 246 | L | 0.4864 | 0.0 |
| 393 | gap | 0.0576 | - | - | 0.0935 | 0.0 |
| 394 | gap | 0.0069 | - | - | 0.0083 | 0.0 |
| 395 | gap | 0.0075 | - | - | 0.0079 | 0.0 |
| 396 | gap | 0.0068 | - | - | 0.0074 | 0.0 |
| 397 | gap | 0.0073 | - | - | 0.0094 | 0.0 |
| 398 | gap | 0.0114 | - | - | 0.0094 | 0.0 |
| 399 | G | 0.5161 | 247 | A | 0.7077 | 4.0 |
| 400 | I | 0.1731 | 248 | A | 0.3979 | 2.0 |
| 401 | L | 0.1983 | 249 | W | 0.5168 | 4.0 |
| 402 | gap | 0.0174 | - | - | 0.0161 | 0.0 |
| 403 | gap | 0.0120 | - | - | 0.0131 | 0.0 |
| 404 | gap | 0.0071 | - | - | 0.0079 | 0.0 |
| 405 | gap | 0.0105 | - | - | 0.0085 | 0.0 |
| 406 | gap | 0.0100 | - | - | 0.0102 | 0.0 |
| 407 | gap | 0.0110 | - | - | 0.0093 | 0.0 |
| 408 | gap | 0.0123 | - | - | 0.0083 | 0.0 |
| 409 | gap | 0.2949 | - | - | 0.3745 | 0.0 |
| 410 | Q | 0.5964 | 250 | S | 0.4436 | 2.0 |
| 411 | L | 0.1786 | 251 | L | 0.5103 | 4.0 |
| 412 | G | 0.2568 | 252 | G | 0.5080 | 4.0 |
| 413 | I | 0.2133 | 253 | A | 0.4296 | 3.0 |
| 414 | T | 0.3110 | 254 | V | 0.4297 | 1.0 |
| 415 | V | 0.2758 | 255 | C | 0.6428 | 4.0 |
| 416 | G | 0.9142 | 256 | G | 1.2452 | 5.0 |
| 417 | I | 0.2493 | 257 | P | 0.4583 | 0.0 |
| 418 | L | 0.2772 | 258 | S | 0.6371 | 4.0 |
| 419 | L | 0.3147 | 259 | F | 0.6678 | 4.0 |
| 420 | A | 0.3970 | 260 | G | 0.6623 | 3.0 |
| 421 | A | 0.3518 | 261 | P | 0.4329 | 2.0 |
| 422 | L | 0.3277 | 262 | F | 0.5992 | 4.0 |
| 423 | L | 0.3202 | 263 | F | 0.6984 | 4.0 |
| 424 | G | 0.5667 | 264 | G | 0.6772 | 3.0 |
| 425 | Y | 0.3619 | 265 | S | 0.4216 | 0.0 |
| 426 | gap | 0.2053 | - | - | 0.2138 | 0.0 |
| 427 | gap | 0.1568 | - | - | 0.1619 | 0.0 |
| 428 | gap | 0.1501 | - | - | 0.1549 | 0.0 |
| 429 | gap | 0.0622 | - | - | 0.0737 | 0.0 |
| 430 | gap | 0.1638 | - | - | 0.1733 | 0.0 |
| 431 | L | 0.3537 | 266 | I | 0.3900 | 4.0 |
| 432 | L | 0.4158 | 267 | L | 0.4375 | 0.0 |
| 433 | gap | 0.1061 | 268 | T | 0.0996 | 0.0 |
| 434 | gap | 0.0638 | 269 | V | 0.0538 | 0.0 |
| 435 | gap | 0.1710 | 270 | K | 0.2017 | 0.0 |
| 436 | gap | 0.0639 | 271 | A | 0.0752 | 0.0 |
| 437 | gap | 0.0789 | - | - | 0.0790 | 0.0 |
| 438 | gap | 0.0761 | - | - | 0.0878 | 0.0 |
| 439 | gap | 0.0807 | - | - | 0.0762 | 0.0 |
| 440 | gap | 0.0488 | - | - | 0.0487 | 0.0 |
| 441 | gap | 0.0421 | - | - | 0.0415 | 0.0 |
| 442 | gap | 0.0209 | - | - | 0.0212 | 0.0 |
| 443 | gap | 0.0129 | - | - | 0.0133 | 0.0 |
| 444 | gap | 0.0145 | - | - | 0.0156 | 0.0 |
| 445 | gap | 0.0153 | - | - | 0.0149 | 0.0 |
| 446 | gap | 0.0129 | - | - | 0.0127 | 0.0 |
| 447 | gap | 0.0112 | - | - | 0.0113 | 0.0 |
| 448 | gap | 0.0128 | - | - | 0.0131 | 0.0 |
| 449 | gap | 0.0140 | - | - | 0.0140 | 0.0 |
| 450 | gap | 0.0183 | - | - | 0.0181 | 0.0 |
| 451 | gap | 0.0184 | - | - | 0.0180 | 0.0 |
| 452 | gap | 0.0169 | - | - | 0.0162 | 0.0 |
| 453 | gap | 0.0182 | - | - | 0.0180 | 0.0 |
| 454 | gap | 0.0416 | - | - | 0.0423 | 0.0 |
| 455 | gap | 0.0444 | - | - | 0.0446 | 0.0 |
| 456 | gap | 0.0447 | - | - | 0.0433 | 0.0 |
| 457 | gap | 0.0540 | - | - | 0.0585 | 0.0 |
| 458 | gap | 0.1409 | - | - | 0.1310 | 0.0 |
| 459 | gap | 0.0617 | - | - | 0.0639 | 0.0 |
| 460 | gap | 0.1022 | - | - | 0.1064 | 0.0 |
| 461 | gap | 0.0574 | - | - | 0.0599 | 0.0 |
| 462 | gap | 0.1451 | - | - | 0.1790 | 0.0 |
| 463 | G | 0.6778 | 272 | S | 0.6886 | 0.0 |
| 464 | W | 2.2042 | 273 | W | 2.6908 | 1.0 |
| 465 | R | 1.0494 | 274 | R | 1.0186 | 0.0 |
| 466 | W | 0.5551 | 275 | W | 0.7001 | 4.0 |
| 467 | L | 0.4651 | 276 | T | 0.4926 | 4.0 |
| 468 | F | 0.9457 | 277 | F | 1.0517 | 4.0 |
| 469 | gap | 0.0079 | - | - | 0.0078 | 0.0 |
| 470 | gap | 0.0068 | - | - | 0.0068 | 0.0 |
| 471 | gap | 0.0079 | - | - | 0.0078 | 0.0 |
| 472 | L | 0.2574 | 278 | W | 0.5716 | 2.0 |
| 473 | L | 0.3501 | 279 | F | 0.7228 | 4.0 |
| 474 | A | 0.0897 | 280 | M | 0.2869 | 0.0 |
| 475 | G | 0.3436 | 281 | C | 0.6087 | 4.0 |
| 476 | I | 0.3333 | 282 | I | 0.6829 | 4.0 |
| 477 | P | 0.7047 | 283 | I | 0.8648 | 5.0 |
| 478 | A | 0.3421 | 284 | S | 0.5367 | 3.0 |
| 479 | L | 0.3066 | 285 | G | 0.6465 | 4.0 |
| 480 | L | 0.3865 | 286 | F | 0.7049 | 3.0 |
| 481 | L | 0.2474 | 287 | S | 0.4961 | 4.0 |
| 482 | L | 0.2327 | 288 | F | 0.6259 | 4.0 |
| 483 | L | 0.3919 | 289 | V | 0.7774 | 6.0 |
| 484 | L | 0.2400 | 290 | M | 0.5651 | 4.0 |
| 485 | L | 0.1793 | 291 | L | 0.4857 | 4.0 |
| 486 | gap | 0.1150 | 292 | C | 0.2991 | 0.0 |
| 487 | F | 0.4489 | 293 | F | 0.5813 | 1.0 |
| 488 | F | 0.2197 | 294 | T | 0.5262 | 0.0 |
| 489 | gap | 0.0450 | - | - | 0.0381 | 0.0 |
| 490 | gap | 0.0642 | - | - | 0.0402 | 0.0 |
| 491 | gap | 0.0355 | - | - | 0.0264 | 0.0 |
| 492 | gap | 0.0325 | - | - | 0.0283 | 0.0 |
| 493 | gap | 0.0479 | - | - | 0.0258 | 0.0 |
| 494 | gap | 0.0158 | - | - | 0.0130 | 0.0 |
| 495 | gap | 0.0167 | - | - | 0.0175 | 0.0 |
| 496 | gap | 0.0357 | - | - | 0.0211 | 0.0 |
| 497 | gap | 0.0423 | - | - | 0.0276 | 0.0 |
| 498 | gap | 0.0435 | - | - | 0.0318 | 0.0 |
| 499 | gap | 0.0283 | - | - | 0.0258 | 0.0 |
| 500 | gap | 0.0276 | - | - | 0.0255 | 0.0 |
| 501 | gap | 0.0356 | - | - | 0.0324 | 0.0 |
| 502 | gap | 0.0251 | - | - | 0.0212 | 0.0 |
| 503 | L | 0.3922 | 295 | L | 0.5963 | 0.0 |
| 504 | P | 1.3077 | 296 | P | 1.3348 | 0.0 |
| 505 | E | 0.9447 | 297 | E | 0.9144 | 0.0 |
| 506 | S | 0.7244 | 298 | T | 0.7813 | 1.0 |
| 507 | P | 1.2043 | 299 | F | 0.9039 | 0.0 |
| 508 | R | 0.9295 | 300 | G | 0.5194 | 0.0 |
| 509 | W | 0.5028 | 301 | K | 0.6720 | 0.0 |
| 510 | L | 0.3162 | 302 | T | 0.3092 | 0.0 |
| 511 | gap | 0.0916 | 303 | L | 0.0853 | 0.0 |
| 512 | gap | 0.1532 | 304 | L | 0.1521 | 0.0 |
| 513 | gap | 0.1668 | - | - | 0.1538 | 0.0 |
| 514 | gap | 0.0668 | - | - | 0.0694 | 0.0 |
| 515 | gap | 0.0461 | - | - | 0.0439 | 0.0 |
| 516 | gap | 0.0271 | - | - | 0.0265 | 0.0 |
| 517 | gap | 0.0571 | - | - | 0.0554 | 0.0 |
| 518 | gap | 0.0656 | - | - | 0.0620 | 0.0 |
| 519 | gap | 0.0188 | - | - | 0.0187 | 0.0 |
| 520 | gap | 0.0196 | - | - | 0.0219 | 0.0 |
| 521 | gap | 0.0184 | - | - | 0.0178 | 0.0 |
| 522 | gap | 0.1654 | - | - | 0.1538 | 0.0 |
| 523 | gap | 0.4266 | - | - | 0.4092 | 0.0 |
| 524 | gap | 0.3189 | - | - | 0.2999 | 0.0 |
| 525 | gap | 0.1132 | - | - | 0.1154 | 0.0 |
| 526 | gap | 0.2190 | - | - | 0.1999 | 0.0 |
| 527 | gap | 0.3371 | - | - | 0.3145 | 0.0 |
| 528 | gap | 0.5039 | - | - | 0.4893 | 0.0 |
| 529 | gap | 0.1821 | 305 | Y | 0.1759 | 0.0 |
| 530 | gap | 0.2219 | 306 | R | 0.2041 | 0.0 |
| 531 | gap | 0.0918 | 307 | K | 0.0881 | 0.0 |
| 532 | gap | 0.0738 | 308 | A | 0.0674 | 0.0 |
| 533 | gap | 0.0494 | 309 | K | 0.0446 | 0.0 |
| 534 | gap | 0.1907 | 310 | R | 0.1786 | 0.0 |
| 535 | L | 0.2966 | 311 | L | 0.3031 | 0.0 |
| 536 | K | 0.3020 | 312 | R | 0.2622 | 0.0 |
| 537 | gap | 0.3070 | 313 | A | 0.2820 | 0.0 |
| 538 | gap | 0.1779 | 314 | I | 0.1838 | 0.0 |
| 539 | gap | 0.2839 | 315 | T | 0.2634 | 0.0 |
| 540 | gap | 0.2354 | 316 | G | 0.2166 | 0.0 |
| 541 | gap | 0.1040 | 317 | N | 0.0940 | 0.0 |
| 542 | gap | 0.1824 | 318 | D | 0.1823 | 0.0 |
| 543 | gap | 0.0519 | 319 | R | 0.0393 | 0.0 |
| 544 | gap | 0.0384 | - | - | 0.0446 | 0.0 |
| 545 | gap | 0.0845 | - | - | 0.0838 | 0.0 |
| 546 | gap | 0.1545 | - | - | 0.1433 | 0.0 |
| 547 | gap | 0.2149 | - | - | 0.1938 | 0.0 |
| 548 | gap | 0.1772 | - | - | 0.1590 | 0.0 |
| 549 | gap | 0.1655 | - | - | 0.1546 | 0.0 |
| 550 | gap | 0.1077 | - | - | 0.1016 | 0.0 |
| 551 | gap | 0.2920 | - | - | 0.2771 | 0.0 |
| 552 | gap | 0.1316 | 320 | I | 0.1347 | 0.0 |
| 553 | gap | 0.0804 | 321 | T | 0.0688 | 0.0 |
| 554 | gap | 0.1973 | 322 | S | 0.1870 | 0.0 |
| 555 | gap | 0.1674 | 323 | E | 0.1703 | 0.0 |
| 556 | gap | 0.1196 | 324 | G | 0.1022 | 0.0 |
| 557 | gap | 0.1632 | 325 | E | 0.1464 | 0.0 |
| 558 | gap | 0.1381 | 326 | I | 0.1341 | 0.0 |
| 559 | gap | 0.1383 | 327 | E | 0.1345 | 0.0 |
| 560 | gap | 0.1809 | 328 | N | 0.1599 | 0.0 |
| 561 | gap | 0.0952 | 329 | S | 0.0841 | 0.0 |
| 562 | gap | 0.0185 | - | - | 0.0187 | 0.0 |
| 563 | gap | 0.0312 | - | - | 0.0292 | 0.0 |
| 564 | gap | 0.0313 | - | - | 0.0355 | 0.0 |
| 565 | gap | 0.0264 | - | - | 0.0259 | 0.0 |
| 566 | gap | 0.0333 | - | - | 0.0320 | 0.0 |
| 567 | gap | 0.0291 | - | - | 0.0265 | 0.0 |
| 568 | gap | 0.0288 | - | - | 0.0278 | 0.0 |
| 569 | gap | 0.0346 | - | - | 0.0352 | 0.0 |
| 570 | gap | 0.0328 | - | - | 0.0335 | 0.0 |
| 571 | gap | 0.0359 | - | - | 0.0366 | 0.0 |
| 572 | gap | 0.0410 | - | - | 0.0401 | 0.0 |
| 573 | gap | 0.0381 | - | - | 0.0384 | 0.0 |
| 574 | gap | 0.0391 | - | - | 0.0385 | 0.0 |
| 575 | gap | 0.0391 | - | - | 0.0389 | 0.0 |
| 576 | gap | 0.0317 | - | - | 0.0318 | 0.0 |
| 577 | gap | 0.0392 | - | - | 0.0385 | 0.0 |
| 578 | gap | 0.0172 | - | - | 0.0174 | 0.0 |
| 579 | gap | 0.0379 | - | - | 0.0362 | 0.0 |
| 580 | gap | 0.0291 | - | - | 0.0286 | 0.0 |
| 581 | gap | 0.0266 | - | - | 0.0251 | 0.0 |
| 582 | gap | 0.0411 | - | - | 0.0414 | 0.0 |
| 583 | gap | 0.0279 | - | - | 0.0294 | 0.0 |
| 584 | gap | 0.0401 | - | - | 0.0387 | 0.0 |
| 585 | gap | 0.0359 | - | - | 0.0371 | 0.0 |
| 586 | gap | 0.0660 | - | - | 0.0693 | 0.0 |
| 587 | gap | 0.0285 | - | - | 0.0295 | 0.0 |
| 588 | gap | 0.0422 | - | - | 0.0413 | 0.0 |
| 589 | gap | 0.0474 | - | - | 0.0467 | 0.0 |
| 590 | gap | 0.0638 | - | - | 0.0579 | 0.0 |
| 591 | gap | 0.0480 | - | - | 0.0433 | 0.0 |
| 592 | gap | 0.0433 | - | - | 0.0390 | 0.0 |
| 593 | gap | 0.0750 | - | - | 0.0716 | 0.0 |
| 594 | gap | 0.0518 | - | - | 0.0493 | 0.0 |
| 595 | gap | 0.0298 | - | - | 0.0323 | 0.0 |
| 596 | gap | 0.0326 | - | - | 0.0348 | 0.0 |
| 597 | gap | 0.0846 | - | - | 0.0811 | 0.0 |
| 598 | gap | 0.0700 | - | - | 0.0744 | 0.0 |
| 599 | gap | 0.0754 | - | - | 0.0817 | 0.0 |
| 600 | gap | 0.1048 | 330 | K | 0.1072 | 0.0 |
| 601 | gap | 0.0673 | 331 | M | 0.0623 | 0.0 |
| 602 | gap | 0.1024 | 332 | T | 0.0895 | 0.0 |
| 603 | gap | 0.0889 | 333 | S | 0.0841 | 0.0 |
| 604 | gap | 0.1073 | 334 | H | 0.1023 | 0.0 |
| 605 | gap | 0.1429 | 335 | E | 0.1517 | 0.0 |
| 606 | L | 0.1147 | 336 | L | 0.1154 | 0.0 |
| 607 | L | 0.1194 | 337 | I | 0.1344 | 0.0 |
| 608 | gap | 0.0995 | 338 | I | 0.1249 | 0.0 |
| 609 | gap | 0.0584 | - | - | 0.0580 | 0.0 |
| 610 | gap | 0.0427 | - | - | 0.0413 | 0.0 |
| 611 | gap | 0.0412 | - | - | 0.0438 | 0.0 |
| 612 | gap | 0.0712 | 339 | D | 0.0990 | 0.0 |
| 613 | gap | 0.0781 | 340 | T | 0.0770 | 0.0 |
| 614 | gap | 0.1166 | 341 | L | 0.1183 | 0.0 |
| 615 | gap | 0.1116 | 342 | W | 0.1224 | 0.0 |
| 616 | gap | 0.0334 | - | - | 0.0337 | 0.0 |
| 617 | gap | 0.0390 | - | - | 0.0450 | 0.0 |
| 618 | gap | 0.0323 | - | - | 0.0332 | 0.0 |
| 619 | gap | 0.0280 | - | - | 0.0269 | 0.0 |
| 620 | gap | 0.0277 | - | - | 0.0271 | 0.0 |
| 621 | gap | 0.0332 | - | - | 0.0361 | 0.0 |
| 622 | gap | 0.0194 | - | - | 0.0198 | 0.0 |
| 623 | gap | 0.0292 | - | - | 0.0281 | 0.0 |
| 624 | gap | 0.0283 | - | - | 0.0270 | 0.0 |
| 625 | gap | 0.0183 | - | - | 0.0190 | 0.0 |
| 626 | gap | 0.0692 | - | - | 0.0730 | 0.0 |
| 627 | gap | 0.0785 | - | - | 0.0794 | 0.0 |
| 628 | gap | 0.0569 | - | - | 0.0549 | 0.0 |
| 629 | gap | 0.0318 | - | - | 0.0325 | 0.0 |
| 630 | gap | 0.0225 | - | - | 0.0227 | 0.0 |
| 631 | gap | 0.0320 | - | - | 0.0304 | 0.0 |
| 632 | gap | 0.1039 | 343 | R | 0.1004 | 0.0 |
| 633 | gap | 0.1225 | 344 | P | 0.1313 | 0.0 |
| 634 | gap | 0.1282 | 345 | L | 0.1602 | 0.0 |
| 635 | gap | 0.1340 | 346 | E | 0.1429 | 0.0 |
| 636 | gap | 0.1028 | 347 | I | 0.1013 | 0.0 |
| 637 | L | 0.3177 | 348 | T | 0.3271 | 0.0 |
| 638 | F | 0.3398 | 349 | V | 0.3877 | 0.0 |
| 639 | R | 0.2845 | 350 | M | 0.2886 | 0.0 |
| 640 | K | 0.2025 | 351 | E | 0.2198 | 0.0 |
| 641 | P | 0.2239 | 352 | P | 0.2215 | 1.0 |
| 642 | L | 0.2073 | 353 | V | 0.1266 | 0.0 |
| 643 | gap | 0.0255 | - | - | 0.0221 | 0.0 |
| 644 | gap | 0.0203 | - | - | 0.0216 | 0.0 |
| 645 | gap | 0.0247 | - | - | 0.0214 | 0.0 |
| 646 | gap | 0.0310 | - | - | 0.0338 | 0.0 |
| 647 | gap | 0.0349 | - | - | 0.0390 | 0.0 |
| 648 | gap | 0.0587 | - | - | 0.0554 | 0.0 |
| 649 | gap | 0.0349 | - | - | 0.0336 | 0.0 |
| 650 | gap | 0.0362 | - | - | 0.0295 | 0.0 |
| 651 | L | 0.1583 | 354 | V | 0.3603 | 4.0 |
| 652 | L | 0.3609 | 355 | L | 0.4748 | 0.0 |
| 653 | L | 0.2429 | 356 | L | 0.2702 | 1.0 |
| 654 | A | 0.2081 | 357 | I | 0.2051 | 1.0 |
| 655 | L | 0.2024 | 358 | N | 0.4671 | 4.0 |
| 656 | I | 0.2591 | 359 | I | 0.5438 | 4.0 |
| 657 | gap | 0.1351 | 360 | Y | 0.1836 | 0.0 |
| 658 | gap | 0.0084 | - | - | 0.0102 | 0.0 |
| 659 | gap | 0.0101 | - | - | 0.0116 | 0.0 |
| 660 | gap | 0.0145 | - | - | 0.0131 | 0.0 |
| 661 | gap | 0.0126 | - | - | 0.0121 | 0.0 |
| 662 | gap | 0.0066 | - | - | 0.0078 | 0.0 |
| 663 | gap | 0.0066 | - | - | 0.0078 | 0.0 |
| 664 | gap | 0.0200 | - | - | 0.0133 | 0.0 |
| 665 | gap | 0.0138 | - | - | 0.0135 | 0.0 |
| 666 | I | 0.2105 | 361 | I | 0.5128 | 4.0 |
| 667 | G | 0.1874 | 362 | A | 0.4844 | 4.0 |
| 668 | L | 0.3255 | 363 | M | 0.6971 | 6.0 |
| 669 | G | 0.1246 | 364 | V | 0.3511 | 4.0 |
| 670 | L | 0.2656 | 365 | Y | 0.5220 | 0.0 |
| 671 | A | 0.1654 | 366 | S | 0.3133 | 4.0 |
| 672 | A | 0.1507 | 367 | I | 0.3614 | 4.0 |
| 673 | F | 0.3241 | 368 | L | 0.6206 | 4.0 |
| 674 | Q | 0.6414 | 369 | Y | 0.6876 | 4.0 |
| 675 | Q | 0.5766 | 370 | L | 0.5034 | 2.0 |
| 676 | F | 0.3010 | 371 | F | 0.5935 | 3.0 |
| 677 | T | 0.2397 | 372 | F | 0.5067 | 4.0 |
| 678 | G | 0.3136 | 373 | E | 0.4689 | 0.0 |
| 679 | Y | 0.5175 | 374 | V | 0.6462 | 3.0 |
| 680 | N | 0.4833 | 375 | F | 0.5305 | 3.0 |
| 681 | P | 0.4511 | 376 | P | 0.4721 | 1.0 |
| 682 | I | 0.3825 | 377 | I | 0.4017 | 0.0 |
| 683 | L | 0.5328 | 378 | Y | 0.5920 | 3.0 |
| 684 | Y | 0.5928 | 379 | F | 0.6697 | 0.0 |
| 685 | Y | 0.6024 | 380 | V | 0.6748 | 1.0 |
| 686 | gap | 0.2352 | 381 | G | 0.2352 | 0.0 |
| 687 | gap | 0.0759 | 382 | V | 0.0756 | 0.0 |
| 688 | gap | 0.0930 | 383 | K | 0.0978 | 0.0 |
| 689 | gap | 0.0702 | 384 | H | 0.0668 | 0.0 |
| 690 | gap | 0.0830 | 385 | F | 0.1003 | 0.0 |
| 691 | gap | 0.0733 | 386 | T | 0.0656 | 0.0 |
| 692 | P | 0.1950 | 387 | L | 0.1977 | 0.0 |
| 693 | S | 0.1277 | 388 | V | 0.1142 | 2.0 |
| 694 | I | 0.2783 | 389 | E | 0.2723 | 1.0 |
| 695 | F | 0.2495 | 390 | L | 0.4007 | 3.0 |
| 696 | gap | 0.4954 | 391 | G | 0.2241 | 0.0 |
| 697 | S | 0.2209 | 392 | T | 0.1569 | 0.0 |
| 698 | gap | 0.1709 | 393 | T | 0.1570 | 0.0 |
| 699 | gap | 0.2808 | 394 | Y | 0.2609 | 0.0 |
| 700 | gap | 0.1729 | - | - | 0.2653 | 0.0 |
| 701 | gap | 0.1452 | - | - | 0.0916 | 0.0 |
| 702 | gap | 0.2909 | - | - | 0.2005 | 0.0 |
| 703 | gap | 0.3093 | - | - | 0.1590 | 0.0 |
| 704 | gap | 0.0202 | - | - | 0.0175 | 0.0 |
| 705 | gap | 0.0103 | - | - | 0.0125 | 0.0 |
| 706 | gap | 0.0188 | - | - | 0.0140 | 0.0 |
| 707 | gap | 0.0211 | - | - | 0.0224 | 0.0 |
| 708 | gap | 0.0294 | - | - | 0.0195 | 0.0 |
| 709 | gap | 0.0197 | - | - | 0.0195 | 0.0 |
| 710 | gap | 0.0161 | - | - | 0.0212 | 0.0 |
| 711 | gap | 0.0202 | - | - | 0.0166 | 0.0 |
| 712 | gap | 0.0181 | - | - | 0.0185 | 0.0 |
| 713 | gap | 0.0330 | - | - | 0.0188 | 0.0 |
| 714 | gap | 0.0279 | - | - | 0.0182 | 0.0 |
| 715 | gap | 0.0186 | - | - | 0.0204 | 0.0 |
| 716 | gap | 0.0246 | - | - | 0.0167 | 0.0 |
| 717 | gap | 0.0228 | - | - | 0.0185 | 0.0 |
| 718 | gap | 0.0334 | - | - | 0.0397 | 0.0 |
| 719 | gap | 0.0261 | - | - | 0.0269 | 0.0 |
| 720 | gap | 0.0174 | - | - | 0.0224 | 0.0 |
| 721 | gap | 0.0397 | - | - | 0.0299 | 0.0 |
| 722 | gap | 0.0252 | - | - | 0.0306 | 0.0 |
| 723 | gap | 0.0362 | - | - | 0.0321 | 0.0 |
| 724 | gap | 0.0251 | - | - | 0.0328 | 0.0 |
| 725 | gap | 0.0545 | - | - | 0.0362 | 0.0 |
| 726 | gap | 0.0511 | - | - | 0.0409 | 0.0 |
| 727 | gap | 0.0596 | - | - | 0.0479 | 0.0 |
| 728 | gap | 0.0351 | - | - | 0.0347 | 0.0 |
| 729 | gap | 0.0444 | - | - | 0.0331 | 0.0 |
| 730 | gap | 0.0208 | - | - | 0.0169 | 0.0 |
| 731 | gap | 0.0224 | - | - | 0.0211 | 0.0 |
| 732 | gap | 0.0172 | - | - | 0.0210 | 0.0 |
| 733 | gap | 0.0267 | - | - | 0.0160 | 0.0 |
| 734 | gap | 0.0200 | - | - | 0.0217 | 0.0 |
| 735 | gap | 0.0180 | - | - | 0.0162 | 0.0 |
| 736 | gap | 0.0330 | - | - | 0.0279 | 0.0 |
| 737 | gap | 0.0276 | - | - | 0.0193 | 0.0 |
| 738 | gap | 0.0294 | - | - | 0.0195 | 0.0 |
| 739 | gap | 0.0218 | - | - | 0.0283 | 0.0 |
| 740 | gap | 0.0207 | - | - | 0.0248 | 0.0 |
| 741 | gap | 0.0199 | - | - | 0.0183 | 0.0 |
| 742 | gap | 0.0128 | - | - | 0.0160 | 0.0 |
| 743 | gap | 0.0231 | - | - | 0.0174 | 0.0 |
| 744 | gap | 0.0146 | - | - | 0.0201 | 0.0 |
| 745 | gap | 0.0735 | - | - | 0.0495 | 0.0 |
| 746 | gap | 0.0390 | - | - | 0.0334 | 0.0 |
| 747 | gap | 0.0346 | - | - | 0.0398 | 0.0 |
| 748 | gap | 0.0266 | - | - | 0.0174 | 0.0 |
| 749 | gap | 0.0294 | - | - | 0.0307 | 0.0 |
| 750 | gap | 0.0625 | - | - | 0.0363 | 0.0 |
| 751 | gap | 0.0533 | - | - | 0.0410 | 0.0 |
| 752 | gap | 0.0292 | - | - | 0.0316 | 0.0 |
| 753 | gap | 0.0209 | - | - | 0.0283 | 0.0 |
| 754 | gap | 0.0276 | - | - | 0.0356 | 0.0 |
| 755 | gap | 0.0285 | - | - | 0.0151 | 0.0 |
| 756 | gap | 0.0228 | - | - | 0.0144 | 0.0 |
| 757 | gap | 0.0341 | - | - | 0.0368 | 0.0 |
| 758 | gap | 0.0229 | - | - | 0.0234 | 0.0 |
| 759 | gap | 0.0218 | - | - | 0.0283 | 0.0 |
| 760 | gap | 0.0379 | - | - | 0.0237 | 0.0 |
| 761 | gap | 0.0398 | - | - | 0.0257 | 0.0 |
| 762 | gap | 0.0214 | - | - | 0.0237 | 0.0 |
| 763 | gap | 0.0256 | - | - | 0.0256 | 0.0 |
| 764 | gap | 0.0226 | - | - | 0.0236 | 0.0 |
| 765 | gap | 0.0136 | - | - | 0.0146 | 0.0 |
| 766 | gap | 0.0297 | - | - | 0.0216 | 0.0 |
| 767 | gap | 0.0256 | - | - | 0.0256 | 0.0 |
| 768 | gap | 0.0216 | - | - | 0.0243 | 0.0 |
| 769 | gap | 0.0218 | - | - | 0.0283 | 0.0 |
| 770 | gap | 0.0294 | - | - | 0.0195 | 0.0 |
| 771 | gap | 0.0330 | - | - | 0.0279 | 0.0 |
| 772 | gap | 0.0341 | - | - | 0.0368 | 0.0 |
| 773 | gap | 0.0256 | - | - | 0.0256 | 0.0 |
| 774 | gap | 0.0159 | - | - | 0.0216 | 0.0 |
| 775 | gap | 0.0209 | - | - | 0.0189 | 0.0 |
| 776 | gap | 0.0222 | - | - | 0.0161 | 0.0 |
| 777 | gap | 0.0256 | - | - | 0.0256 | 0.0 |
| 778 | gap | 0.0172 | - | - | 0.0187 | 0.0 |
| 779 | gap | 0.0218 | - | - | 0.0283 | 0.0 |
| 780 | gap | 0.0276 | - | - | 0.0234 | 0.0 |
| 781 | gap | 0.0330 | - | - | 0.0279 | 0.0 |
| 782 | gap | 0.0256 | - | - | 0.0256 | 0.0 |
| 783 | gap | 0.0398 | - | - | 0.0257 | 0.0 |
| 784 | gap | 0.0130 | - | - | 0.0175 | 0.0 |
| 785 | gap | 0.0202 | - | - | 0.0179 | 0.0 |
| 786 | gap | 0.0252 | - | - | 0.0154 | 0.0 |
| 787 | gap | 0.0292 | - | - | 0.0259 | 0.0 |
| 788 | gap | 0.0368 | - | - | 0.0248 | 0.0 |
| 789 | gap | 0.0218 | - | - | 0.0283 | 0.0 |
| 790 | gap | 0.0153 | - | - | 0.0189 | 0.0 |
| 791 | gap | 0.0320 | - | - | 0.0209 | 0.0 |
| 792 | gap | 0.0209 | - | - | 0.0224 | 0.0 |
| 793 | gap | 0.0281 | - | - | 0.0194 | 0.0 |
| 794 | gap | 0.0146 | - | - | 0.0201 | 0.0 |
| 795 | gap | 0.0166 | - | - | 0.0148 | 0.0 |
| 796 | gap | 0.0330 | - | - | 0.0279 | 0.0 |
| 797 | gap | 0.0209 | - | - | 0.0224 | 0.0 |
| 798 | gap | 0.0190 | - | - | 0.0193 | 0.0 |
| 799 | gap | 0.0218 | - | - | 0.0283 | 0.0 |
| 800 | gap | 0.0130 | - | - | 0.0175 | 0.0 |
| 801 | gap | 0.0367 | - | - | 0.0244 | 0.0 |
| 802 | gap | 0.0278 | - | - | 0.0225 | 0.0 |
| 803 | gap | 0.0368 | - | - | 0.0248 | 0.0 |
| 804 | gap | 0.0282 | - | - | 0.0188 | 0.0 |
| 805 | gap | 0.0198 | - | - | 0.0234 | 0.0 |
| 806 | gap | 0.0681 | - | - | 0.0736 | 0.0 |
| 807 | gap | 0.0409 | - | - | 0.0261 | 0.0 |
| 808 | gap | 0.0247 | - | - | 0.0280 | 0.0 |
| 809 | gap | 0.0297 | - | - | 0.0248 | 0.0 |
| 810 | gap | 0.0226 | - | - | 0.0242 | 0.0 |
| 811 | gap | 0.0591 | - | - | 0.0345 | 0.0 |
| 812 | gap | 0.0869 | - | - | 0.0534 | 0.0 |
| 813 | gap | 0.0898 | - | - | 0.0576 | 0.0 |
| 814 | gap | 0.0218 | - | - | 0.0183 | 0.0 |
| 815 | gap | 0.0776 | - | - | 0.0739 | 0.0 |
| 816 | gap | 0.0717 | - | - | 0.0464 | 0.0 |
| 817 | gap | 0.1355 | - | - | 0.0494 | 0.0 |
| 818 | gap | 0.2766 | 395 | M | 0.0879 | 0.0 |
| 819 | gap | 0.2777 | 396 | S | 0.1144 | 0.0 |
| 820 | A | 0.2507 | 397 | I | 0.4004 | 0.0 |
| 821 | L | 0.1660 | 398 | V | 0.3020 | 4.0 |
| 822 | L | 0.2505 | 399 | I | 0.4330 | 4.0 |
| 823 | L | 0.0970 | 400 | G | 0.2747 | 2.0 |
| 824 | S | 0.1492 | 401 | I | 0.2969 | 2.0 |
| 825 | A | 0.2844 | 402 | V | 0.6532 | 4.0 |
| 826 | I | 0.2399 | 403 | I | 0.5522 | 4.0 |
| 827 | F | 0.1595 | 404 | A | 0.3353 | 2.0 |
| 828 | G | 0.3276 | 405 | A | 0.5270 | 0.0 |
| 829 | V | 0.2451 | 406 | F | 0.6009 | 4.0 |
| 830 | V | 0.3287 | 407 | I | 0.6158 | 5.0 |
| 831 | N | 0.5986 | 408 | Y | 0.3736 | 1.0 |
| 832 | I | 0.4232 | 409 | I | 0.7796 | 3.0 |
| 833 | L | 0.3783 | 410 | P | 0.6306 | 3.0 |
| 834 | G | 0.3735 | 411 | V | 0.6870 | 4.0 |
| 835 | T | 0.4266 | 412 | I | 0.4797 | 2.0 |
| 836 | L | 0.4608 | 413 | R | 0.5502 | 3.0 |
| 837 | L | 0.6722 | - | - | 0.6753 | 0.0 |
| 838 | A | 0.5551 | - | - | 0.5710 | 0.0 |
| 839 | G | 0.4277 | - | - | 0.4205 | 0.0 |
| 840 | gap | 0.2497 | 414 | Q | 0.2890 | 0.0 |
| 841 | L | 0.6015 | 415 | K | 0.6716 | 5.0 |
| 842 | L | 0.3003 | 416 | F | 0.3418 | 4.0 |
| 843 | D | 0.4326 | 417 | T | 0.3828 | 2.0 |
| 844 | D | 0.7392 | 418 | K | 0.6659 | 2.0 |
| 845 | R | 0.4916 | 419 | P | 0.4939 | 0.0 |
| 846 | gap | 0.0076 | - | - | 0.0074 | 0.0 |
| 847 | gap | 0.0087 | - | - | 0.0089 | 0.0 |
| 848 | gap | 0.0074 | - | - | 0.0079 | 0.0 |
| 849 | gap | 0.0087 | - | - | 0.0089 | 0.0 |
| 850 | gap | 0.0097 | - | - | 0.0094 | 0.0 |
| 851 | gap | 0.0076 | - | - | 0.0074 | 0.0 |
| 852 | gap | 0.0088 | - | - | 0.0085 | 0.0 |
| 853 | gap | 0.0113 | - | - | 0.0110 | 0.0 |
| 854 | gap | 0.0082 | - | - | 0.0079 | 0.0 |
| 855 | gap | 0.0097 | - | - | 0.0094 | 0.0 |
| 856 | gap | 0.0083 | - | - | 0.0083 | 0.0 |
| 857 | gap | 0.0085 | - | - | 0.0085 | 0.0 |
| 858 | gap | 0.0894 | - | - | 0.0886 | 0.0 |
| 859 | gap | 0.0681 | 420 | I | 0.0657 | 0.0 |
| 860 | gap | 0.0342 | 421 | L | 0.0314 | 0.0 |
| 861 | gap | 0.0682 | 422 | R | 0.0656 | 0.0 |
| 862 | gap | 0.0558 | 423 | Q | 0.0560 | 0.0 |
| 863 | gap | 0.0497 | 424 | E | 0.0480 | 0.0 |
| 864 | gap | 0.0684 | 425 | Q | 0.0654 | 0.0 |
| 865 | gap | 0.0661 | 426 | V | 0.0692 | 0.0 |
| 866 | gap | 0.0748 | 427 | F | 0.0721 | 0.0 |
| 867 | gap | 0.1119 | 428 | P | 0.1254 | 0.0 |
| 868 | gap | 0.0875 | 429 | E | 0.1086 | 0.0 |
| 869 | G | 0.5405 | 430 | V | 0.5449 | 0.0 |
| 870 | R | 1.2703 | 431 | F | 0.8199 | 0.0 |
| 871 | R | 1.1746 | 432 | I | 0.6557 | 0.0 |
| 872 | P | 0.2871 | 433 | P | 0.1763 | 1.0 |
| 873 | L | 0.3267 | 434 | I | 0.4733 | 1.0 |
| 874 | L | 0.2568 | 435 | A | 0.4791 | 2.0 |
| 875 | L | 0.2367 | 436 | I | 0.5224 | 5.0 |
| 876 | L | 0.0901 | 437 | V | 0.3272 | 4.0 |
| 877 | G | 0.2260 | 438 | G | 0.5140 | 4.0 |
| 878 | L | 0.1754 | 439 | G | 0.4783 | 3.0 |
| 879 | L | 0.1458 | 440 | I | 0.4738 | 4.0 |
| 880 | L | 0.1784 | 441 | L | 0.4269 | 4.0 |
| 881 | M | 0.4168 | 442 | L | 0.7457 | 4.0 |
| 882 | A | 0.1801 | 443 | T | 0.4919 | 4.0 |
| 883 | I | 0.2310 | 444 | S | 0.5921 | 4.0 |
| 884 | A | 0.2387 | 445 | G | 0.3951 | 3.0 |
| 885 | gap | 0.0113 | - | - | 0.0137 | 0.0 |
| 886 | gap | 0.0120 | - | - | 0.0131 | 0.0 |
| 887 | gap | 0.0071 | - | - | 0.0079 | 0.0 |
| 888 | gap | 0.0071 | - | - | 0.0079 | 0.0 |
| 889 | L | 0.2234 | 446 | L | 0.4990 | 4.0 |
| 890 | L | 0.2574 | 447 | F | 0.6233 | 4.0 |
| 891 | L | 0.2351 | 448 | I | 0.6028 | 4.0 |
| 892 | L | 0.2115 | 449 | F | 0.5382 | 4.0 |
| 893 | G | 0.1384 | 450 | G | 0.4219 | 3.0 |
| 894 | gap | 0.0364 | - | - | 0.0409 | 0.0 |
| 895 | gap | 0.0363 | - | - | 0.0331 | 0.0 |
| 896 | gap | 0.0424 | - | - | 0.0433 | 0.0 |
| 897 | gap | 0.0234 | - | - | 0.0209 | 0.0 |
| 898 | gap | 0.0248 | - | - | 0.0216 | 0.0 |
| 899 | gap | 0.0232 | - | - | 0.0247 | 0.0 |
| 900 | gap | 0.0302 | - | - | 0.0202 | 0.0 |
| 901 | gap | 0.0123 | - | - | 0.0083 | 0.0 |
| 902 | gap | 0.0068 | - | - | 0.0074 | 0.0 |
| 903 | gap | 0.0100 | - | - | 0.0102 | 0.0 |
| 904 | gap | 0.0071 | - | - | 0.0079 | 0.0 |
| 905 | gap | 0.0114 | - | - | 0.0123 | 0.0 |
| 906 | gap | 0.0270 | - | - | 0.0224 | 0.0 |
| 907 | L | 0.1601 | 451 | W | 0.4757 | 4.0 |
| 908 | L | 0.0763 | 452 | S | 0.2589 | 4.0 |
| 909 | G | 0.2394 | 453 | A | 0.3468 | 2.0 |
| 910 | gap | 0.0814 | 454 | N | 0.1000 | 0.0 |
| 911 | gap | 0.0817 | - | - | 0.0975 | 0.0 |
| 912 | gap | 0.0926 | - | - | 0.1269 | 0.0 |
| 913 | gap | 0.0862 | 455 | R | 0.1067 | 0.0 |
| 914 | gap | 0.0598 | 456 | T | 0.0705 | 0.0 |
| 915 | gap | 0.0999 | 457 | T | 0.1096 | 0.0 |
| 916 | gap | 0.0323 | - | - | 0.0375 | 0.0 |
| 917 | gap | 0.0998 | - | - | 0.1002 | 0.0 |
| 918 | gap | 0.0831 | - | - | 0.0793 | 0.0 |
| 919 | gap | 0.0601 | - | - | 0.0652 | 0.0 |
| 920 | gap | 0.0873 | - | - | 0.0783 | 0.0 |
| 921 | gap | 0.0835 | - | - | 0.0764 | 0.0 |
| 922 | gap | 0.0640 | - | - | 0.0600 | 0.0 |
| 923 | gap | 0.0392 | - | - | 0.0426 | 0.0 |
| 924 | gap | 0.0431 | - | - | 0.0410 | 0.0 |
| 925 | gap | 0.0483 | - | - | 0.0492 | 0.0 |
| 926 | gap | 0.0819 | - | - | 0.0822 | 0.0 |
| 927 | gap | 0.0422 | - | - | 0.0450 | 0.0 |
| 928 | gap | 0.0911 | - | - | 0.0958 | 0.0 |
| 929 | gap | 0.0693 | - | - | 0.0680 | 0.0 |
| 930 | gap | 0.0746 | - | - | 0.0784 | 0.0 |
| 931 | gap | 0.0297 | - | - | 0.0284 | 0.0 |
| 932 | gap | 0.0512 | - | - | 0.0538 | 0.0 |
| 933 | gap | 0.0737 | - | - | 0.0703 | 0.0 |
| 934 | gap | 0.0687 | - | - | 0.0717 | 0.0 |
| 935 | gap | 0.0656 | - | - | 0.0634 | 0.0 |
| 936 | gap | 0.0371 | - | - | 0.0358 | 0.0 |
| 937 | gap | 0.0775 | - | - | 0.0777 | 0.0 |
| 938 | gap | 0.0380 | - | - | 0.0362 | 0.0 |
| 939 | gap | 0.0591 | - | - | 0.0620 | 0.0 |
| 940 | gap | 0.0399 | - | - | 0.0432 | 0.0 |
| 941 | gap | 0.0463 | - | - | 0.0439 | 0.0 |
| 942 | gap | 0.0671 | - | - | 0.0659 | 0.0 |
| 943 | gap | 0.0457 | - | - | 0.0471 | 0.0 |
| 944 | gap | 0.0317 | - | - | 0.0312 | 0.0 |
| 945 | gap | 0.0349 | - | - | 0.0336 | 0.0 |
| 946 | gap | 0.0343 | - | - | 0.0348 | 0.0 |
| 947 | gap | 0.0451 | - | - | 0.0427 | 0.0 |
| 948 | gap | 0.0493 | - | - | 0.0476 | 0.0 |
| 949 | gap | 0.0402 | - | - | 0.0409 | 0.0 |
| 950 | gap | 0.0446 | - | - | 0.0463 | 0.0 |
| 951 | gap | 0.0592 | - | - | 0.0565 | 0.0 |
| 952 | gap | 0.0266 | - | - | 0.0262 | 0.0 |
| 953 | gap | 0.0361 | - | - | 0.0372 | 0.0 |
| 954 | gap | 0.0402 | - | - | 0.0387 | 0.0 |
| 955 | gap | 0.0456 | - | - | 0.0462 | 0.0 |
| 956 | gap | 0.0312 | - | - | 0.0332 | 0.0 |
| 957 | gap | 0.0226 | - | - | 0.0231 | 0.0 |
| 958 | gap | 0.0408 | - | - | 0.0405 | 0.0 |
| 959 | gap | 0.0392 | - | - | 0.0374 | 0.0 |
| 960 | gap | 0.0268 | - | - | 0.0281 | 0.0 |
| 961 | gap | 0.0387 | - | - | 0.0362 | 0.0 |
| 962 | gap | 0.0900 | - | - | 0.0952 | 0.0 |
| 963 | gap | 0.0486 | - | - | 0.0516 | 0.0 |
| 964 | gap | 0.0415 | - | - | 0.0403 | 0.0 |
| 965 | gap | 0.0429 | - | - | 0.0436 | 0.0 |
| 966 | gap | 0.0429 | - | - | 0.0429 | 0.0 |
| 967 | gap | 0.0425 | - | - | 0.0418 | 0.0 |
| 968 | gap | 0.0387 | - | - | 0.0372 | 0.0 |
| 969 | gap | 0.0412 | - | - | 0.0404 | 0.0 |
| 970 | gap | 0.0331 | - | - | 0.0320 | 0.0 |
| 971 | gap | 0.0235 | - | - | 0.0265 | 0.0 |
| 972 | gap | 0.0235 | - | - | 0.0237 | 0.0 |
| 973 | gap | 0.0243 | - | - | 0.0236 | 0.0 |
| 974 | gap | 0.0444 | - | - | 0.0437 | 0.0 |
| 975 | gap | 0.0273 | - | - | 0.0269 | 0.0 |
| 976 | gap | 0.0232 | - | - | 0.0229 | 0.0 |
| 977 | gap | 0.0304 | - | - | 0.0305 | 0.0 |
| 978 | gap | 0.0417 | - | - | 0.0418 | 0.0 |
| 979 | gap | 0.0393 | - | - | 0.0394 | 0.0 |
| 980 | gap | 0.0365 | - | - | 0.0365 | 0.0 |
| 981 | gap | 0.0269 | - | - | 0.0265 | 0.0 |
| 982 | gap | 0.0579 | - | - | 0.0559 | 0.0 |
| 983 | gap | 0.0292 | - | - | 0.0324 | 0.0 |
| 984 | gap | 0.0402 | - | - | 0.0443 | 0.0 |
| 985 | gap | 0.0315 | - | - | 0.0341 | 0.0 |
| 986 | gap | 0.0298 | - | - | 0.0278 | 0.0 |
| 987 | gap | 0.0420 | - | - | 0.0395 | 0.0 |
| 988 | gap | 0.0388 | - | - | 0.0391 | 0.0 |
| 989 | gap | 0.0385 | - | - | 0.0395 | 0.0 |
| 990 | gap | 0.0315 | - | - | 0.0315 | 0.0 |
| 991 | gap | 0.0244 | - | - | 0.0237 | 0.0 |
| 992 | gap | 0.0516 | - | - | 0.0518 | 0.0 |
| 993 | gap | 0.0358 | - | - | 0.0346 | 0.0 |
| 994 | gap | 0.0318 | - | - | 0.0323 | 0.0 |
| 995 | gap | 0.0474 | - | - | 0.0484 | 0.0 |
| 996 | gap | 0.0389 | - | - | 0.0375 | 0.0 |
| 997 | gap | 0.0302 | - | - | 0.0293 | 0.0 |
| 998 | gap | 0.0322 | - | - | 0.0307 | 0.0 |
| 999 | gap | 0.0345 | - | - | 0.0364 | 0.0 |
| 1000 | gap | 0.0504 | - | - | 0.0543 | 0.0 |
| 1001 | gap | 0.0487 | - | - | 0.0503 | 0.0 |
| 1002 | gap | 0.0229 | - | - | 0.0236 | 0.0 |
| 1003 | gap | 0.0217 | - | - | 0.0207 | 0.0 |
| 1004 | gap | 0.0319 | - | - | 0.0319 | 0.0 |
| 1005 | gap | 0.0521 | - | - | 0.0482 | 0.0 |
| 1006 | gap | 0.0512 | - | - | 0.0527 | 0.0 |
| 1007 | gap | 0.1190 | 458 | H | 0.1155 | 0.0 |
| 1008 | L | 0.1607 | 459 | W | 0.2260 | 0.0 |
| 1009 | L | 0.3375 | 460 | V | 0.3699 | 0.0 |
| 1010 | G | 0.2073 | 461 | G | 0.2168 | 3.0 |
| 1011 | gap | 0.2382 | 462 | P | 0.2914 | 0.0 |
| 1012 | L | 0.2335 | 463 | L | 0.5685 | 4.0 |
| 1013 | L | 0.2174 | 464 | F | 0.5365 | 4.0 |
| 1014 | L | 0.1884 | 465 | G | 0.4979 | 4.0 |
| 1015 | I | 0.1872 | 466 | A | 0.4932 | 4.0 |
| 1016 | G | 0.2481 | 467 | A | 0.5835 | 4.0 |
| 1017 | I | 0.2371 | 468 | T | 0.5357 | 4.0 |
| 1018 | G | 0.2112 | 469 | T | 0.5117 | 4.0 |
| 1019 | L | 0.1433 | 470 | A | 0.4400 | 0.0 |
| 1020 | F | 0.3330 | 471 | S | 0.6062 | 0.0 |
| 1021 | V | 0.2610 | 472 | G | 0.5340 | 6.0 |
| 1022 | A | 0.1704 | 473 | A | 0.2532 | 1.0 |
| 1023 | G | 0.2501 | 474 | F | 0.5846 | 4.0 |
| 1024 | F | 0.2688 | 475 | L | 0.5165 | 3.0 |
| 1025 | A | 0.2706 | 476 | I | 0.3682 | 3.0 |
| 1026 | L | 0.1750 | 477 | F | 0.3640 | 3.0 |
| 1027 | G | 0.1134 | 478 | Q | 0.2646 | 3.0 |
| 1028 | I | 0.3039 | 479 | T | 0.5311 | 3.0 |
| 1029 | G | 0.1882 | 480 | L | 0.4749 | 4.0 |
| 1030 | P | 0.4160 | 481 | F | 0.2335 | 0.0 |
| 1031 | gap | 0.2298 | 482 | N | 0.2051 | 0.0 |
| 1032 | gap | 0.1606 | 483 | F | 0.1838 | 0.0 |
| 1033 | gap | 0.0934 | - | - | 0.0548 | 0.0 |
| 1034 | gap | 0.0620 | - | - | 0.0537 | 0.0 |
| 1035 | gap | 0.0285 | - | - | 0.0251 | 0.0 |
| 1036 | gap | 0.0355 | - | - | 0.0358 | 0.0 |
| 1037 | gap | 0.0385 | - | - | 0.0279 | 0.0 |
| 1038 | gap | 0.0157 | - | - | 0.0179 | 0.0 |
| 1039 | gap | 0.0355 | - | - | 0.0208 | 0.0 |
| 1040 | gap | 0.0189 | - | - | 0.0237 | 0.0 |
| 1041 | gap | 0.0201 | - | - | 0.0251 | 0.0 |
| 1042 | gap | 0.0227 | - | - | 0.0248 | 0.0 |
| 1043 | gap | 0.0398 | - | - | 0.0269 | 0.0 |
| 1044 | gap | 0.0892 | - | - | 0.0484 | 0.0 |
| 1045 | I | 0.1866 | 484 | M | 0.3413 | 0.0 |
| 1046 | P | 0.3791 | 485 | G | 0.4169 | 3.0 |
| 1047 | W | 0.5142 | 486 | A | 0.6379 | 0.0 |
| 1048 | L | 0.2471 | 487 | S | 0.2216 | 0.0 |
| 1049 | V | 0.3655 | 488 | F | 0.3491 | 0.0 |
| 1050 | gap | 0.1595 | 489 | K | 0.1540 | 0.0 |
| 1051 | S | 0.3537 | 490 | P | 0.3869 | 3.0 |
| 1052 | E | 0.6230 | 491 | H | 0.6084 | 1.0 |
| 1053 | I | 0.2488 | 492 | Y | 0.2951 | 2.0 |
| 1054 | F | 0.3632 | 493 | I | 0.6021 | 0.0 |
| 1055 | gap | 0.4764 | - | - | 0.3631 | 0.0 |
| 1056 | gap | 0.3127 | - | - | 0.2744 | 0.0 |
| 1057 | gap | 0.0110 | - | - | 0.0093 | 0.0 |
| 1058 | gap | 0.0075 | - | - | 0.0079 | 0.0 |
| 1059 | gap | 0.0069 | - | - | 0.0083 | 0.0 |
| 1060 | gap | 0.0120 | - | - | 0.0131 | 0.0 |
| 1061 | gap | 0.0120 | - | - | 0.0131 | 0.0 |
| 1062 | gap | 0.0346 | - | - | 0.0202 | 0.0 |
| 1063 | G | 0.3793 | 494 | A | 0.5007 | 0.0 |
| 1064 | gap | 0.2868 | 495 | S | 0.1760 | 0.0 |
| 1065 | A | 0.2432 | 496 | V | 0.4529 | 0.0 |
| 1066 | R | 0.6374 | 497 | F | 0.4403 | 2.0 |
| 1067 | G | 0.4383 | 498 | A | 0.5447 | 3.0 |
| 1068 | L | 0.2521 | 499 | S | 0.5023 | 2.0 |
| 1069 | G | 0.3511 | 500 | N | 0.5663 | 4.0 |
| 1070 | L | 0.2341 | 501 | D | 0.3434 | 2.0 |
| 1071 | G | 0.2641 | 502 | L | 0.5656 | 0.0 |
| 1072 | L | 0.2095 | 503 | F | 0.5515 | 4.0 |
| 1073 | G | 0.3621 | 504 | R | 0.4820 | 0.0 |
| 1074 | A | 0.3289 | 505 | S | 0.4775 | 0.0 |
| 1075 | L | 0.1407 | 506 | V | 0.4395 | 4.0 |
| 1076 | V | 0.1621 | 507 | I | 0.4359 | 4.0 |
| 1077 | G | 0.6414 | 508 | A | 0.6930 | 4.0 |
| 1078 | W | 0.5203 | 509 | S | 0.6459 | 1.0 |
| 1079 | L | 0.1661 | 510 | V | 0.4851 | 4.0 |
| 1080 | G | 0.2700 | 511 | F | 0.6385 | 4.0 |
| 1081 | P | 0.5273 | 512 | P | 0.5551 | 2.0 |
| 1082 | F | 0.1957 | 513 | L | 0.4047 | 3.0 |
| 1083 | I | 0.2069 | 514 | F | 0.4672 | 4.0 |
| 1084 | I | 0.5794 | 515 | G | 0.5671 | 4.0 |
| 1085 | G | 0.4556 | 516 | A | 0.4637 | 4.0 |
| 1086 | G | 0.2605 | 517 | P | 0.3021 | 0.0 |
| 1087 | gap | 0.2276 | 518 | L | 0.2614 | 0.0 |
| 1088 | gap | 0.0538 | - | - | 0.0606 | 0.0 |
| 1089 | gap | 0.0380 | - | - | 0.0369 | 0.0 |
| 1090 | gap | 0.0519 | - | - | 0.0529 | 0.0 |
| 1091 | gap | 0.0610 | - | - | 0.0611 | 0.0 |
| 1092 | gap | 0.0538 | - | - | 0.0554 | 0.0 |
| 1093 | gap | 0.5146 | 519 | F | 0.5707 | 0.0 |
| 1094 | gap | 0.2275 | 520 | D | 0.2219 | 0.0 |
| 1095 | gap | 0.1026 | 521 | N | 0.1239 | 0.0 |
| 1096 | gap | 0.1843 | 522 | L | 0.1950 | 0.0 |
| 1097 | gap | 0.0568 | 523 | A | 0.0570 | 0.0 |
| 1098 | gap | 0.0557 | 524 | T | 0.0516 | 0.0 |
| 1099 | gap | 0.0250 | - | - | 0.0235 | 0.0 |
| 1100 | gap | 0.0481 | - | - | 0.0501 | 0.0 |
| 1101 | gap | 0.0376 | - | - | 0.0402 | 0.0 |
| 1102 | gap | 0.0172 | - | - | 0.0169 | 0.0 |
| 1103 | gap | 0.0159 | - | - | 0.0160 | 0.0 |
| 1104 | gap | 0.0252 | - | - | 0.0251 | 0.0 |
| 1105 | gap | 0.0246 | - | - | 0.0232 | 0.0 |
| 1106 | gap | 0.0209 | - | - | 0.0210 | 0.0 |
| 1107 | gap | 0.1390 | 525 | P | 0.1411 | 0.0 |
| 1108 | gap | 0.3592 | 526 | E | 0.3688 | 0.0 |
| 1109 | gap | 0.1625 | 527 | Y | 0.1447 | 0.0 |
| 1110 | gap | 0.0819 | 528 | P | 0.0754 | 0.0 |
| 1111 | gap | 0.1878 | 529 | V | 0.3057 | 0.0 |
| 1112 | G | 0.5587 | 530 | A | 0.4403 | 0.0 |
| 1113 | W | 0.2626 | 531 | W | 0.2721 | 1.0 |
| 1114 | Y | 0.3947 | 532 | G | 0.4046 | 0.0 |
| 1115 | G | 0.1542 | 533 | S | 0.1889 | 0.0 |
| 1116 | P | 0.2135 | 534 | S | 0.2984 | 3.0 |
| 1117 | F | 0.3011 | 535 | V | 0.6507 | 4.0 |
| 1118 | F | 0.3092 | 536 | L | 0.6456 | 4.0 |
| 1119 | I | 0.2236 | 537 | G | 0.5651 | 4.0 |
| 1120 | F | 0.4980 | 538 | F | 0.9439 | 4.0 |
| 1121 | A | 0.3182 | 539 | I | 0.5437 | 4.0 |
| 1122 | G | 0.3356 | 540 | T | 0.6551 | 4.0 |
| 1123 | L | 0.2487 | 541 | L | 0.6076 | 4.0 |
| 1124 | A | 0.2732 | 542 | V | 0.4555 | 3.0 |
| 1125 | L | 0.2277 | 543 | M | 0.5447 | 4.0 |
| 1126 | L | 0.2441 | 544 | I | 0.5877 | 4.0 |
| 1127 | A | 0.1786 | 545 | A | 0.4280 | 3.0 |
| 1128 | A | 0.1456 | 546 | I | 0.4694 | 4.0 |
| 1129 | L | 0.2746 | 547 | P | 0.6248 | 3.0 |
| 1130 | F | 0.3338 | 548 | V | 0.7386 | 4.0 |
| 1131 | V | 0.2506 | 549 | L | 0.5517 | 4.0 |
| 1132 | L | 0.2488 | 550 | F | 0.5216 | 4.0 |
| 1133 | L | 0.2337 | 551 | Y | 0.4927 | 2.0 |
| 1134 | L | 0.5120 | 552 | L | 0.5507 | 2.0 |
| 1135 | V | 0.3320 | 553 | N | 0.3491 | 0.0 |
| 1136 | P | 0.3872 | 554 | G | 0.4028 | 0.0 |
| 1137 | E | 0.5750 | 555 | P | 0.5743 | 0.0 |
| 1138 | T | 0.6253 | 556 | K | 0.6364 | 0.0 |
| 1139 | K | 0.4894 | 557 | L | 0.4604 | 0.0 |
| 1140 | G | 0.4901 | 558 | R | 0.4698 | 0.0 |
| 1141 | gap | 0.3023 | 559 | A | 0.2676 | 0.0 |
| 1142 | gap | 0.2273 | 560 | R | 0.2367 | 0.0 |
| 1143 | L | 0.3442 | - | - | 0.3531 | 0.0 |
| 1144 | E | 0.3487 | 561 | S | 0.3337 | 0.0 |
| 1145 | gap | 0.3737 | 562 | K | 0.3619 | 0.0 |
| 1146 | I | 0.2280 | 563 | Y | 0.2192 | 0.0 |
| 1147 | gap | 0.1122 | 564 | A | 0.0979 | 0.0 |
| 1148 | gap | 0.1272 | 565 | N | 0.1155 | 0.0 |
| 1149 | gap | 0.1129 | - | - | 0.0972 | 0.0 |
| 1150 | gap | 0.1819 | - | - | 0.2090 | 0.0 |
| 1151 | gap | 0.1430 | - | - | 0.1590 | 0.0 |
| 1152 | gap | 0.1152 | - | - | 0.1069 | 0.0 |
| 1153 | gap | 0.0977 | - | - | 0.0917 | 0.0 |
| 1154 | gap | 0.1186 | - | - | 0.1134 | 0.0 |
| 1155 | gap | 0.1386 | - | - | 0.1622 | 0.0 |
| 1156 | gap | 0.0715 | - | - | 0.0752 | 0.0 |
| 1157 | gap | 0.1660 | - | - | 0.2029 | 0.0 |
| 1158 | gap | 0.0609 | - | - | 0.0567 | 0.0 |
| 1159 | gap | 0.0907 | - | - | 0.0906 | 0.0 |
| 1160 | gap | 0.0367 | - | - | 0.0386 | 0.0 |
| 1161 | gap | 0.0522 | - | - | 0.0554 | 0.0 |
| 1162 | gap | 0.0956 | - | - | 0.0952 | 0.0 |
| 1163 | gap | 0.0610 | - | - | 0.0617 | 0.0 |
| 1164 | gap | 0.0744 | - | - | 0.0752 | 0.0 |
| 1165 | gap | 0.0735 | - | - | 0.0707 | 0.0 |
| 1166 | gap | 0.0928 | - | - | 0.0840 | 0.0 |
| 1167 | gap | 0.0769 | - | - | 0.0736 | 0.0 |
| 1168 | gap | 0.0705 | - | - | 0.0740 | 0.0 |
| 1169 | gap | 0.0733 | - | - | 0.0684 | 0.0 |
| 1170 | gap | 0.0450 | - | - | 0.0398 | 0.0 |
| 1171 | gap | 0.0635 | - | - | 0.0645 | 0.0 |
| 1172 | gap | 0.0560 | - | - | 0.0563 | 0.0 |
| 1173 | gap | 0.0735 | - | - | 0.0691 | 0.0 |
| 1174 | gap | 0.0597 | - | - | 0.0590 | 0.0 |
| 1175 | gap | 0.0514 | - | - | 0.0458 | 0.0 |
| 1176 | gap | 0.1115 | - | - | 0.1080 | 0.0 |
| 1177 | gap | 0.0665 | - | - | 0.0634 | 0.0 |
| 1178 | gap | 0.0738 | - | - | 0.0670 | 0.0 |
| 1179 | gap | 0.0588 | - | - | 0.0541 | 0.0 |
| 1180 | gap | 0.0471 | - | - | 0.0452 | 0.0 |
| 1181 | gap | 0.0504 | - | - | 0.0474 | 0.0 |
| 1182 | gap | 0.0460 | - | - | 0.0389 | 0.0 |
| 1183 | gap | 0.0731 | - | - | 0.0645 | 0.0 |
| 1184 | gap | 0.0461 | - | - | 0.0418 | 0.0 |
| 1185 | gap | 0.0698 | - | - | 0.0693 | 0.0 |
| 1186 | gap | 0.0796 | - | - | 0.0746 | 0.0 |
| 1187 | gap | 0.1109 | - | - | 0.1141 | 0.0 |
| 1188 | gap | 0.0617 | - | - | 0.0574 | 0.0 |
| 1189 | gap | 0.0264 | - | - | 0.0231 | 0.0 |
| 1190 | gap | 0.0601 | - | - | 0.0596 | 0.0 |
| 1191 | gap | 0.0605 | - | - | 0.0560 | 0.0 |
| 1192 | gap | 0.0447 | - | - | 0.0421 | 0.0 |
| 1193 | gap | 0.0520 | - | - | 0.0443 | 0.0 |
| 1194 | gap | 0.0429 | - | - | 0.0439 | 0.0 |
| 1195 | gap | 0.0952 | - | - | 0.0960 | 0.0 |
| 1196 | gap | 0.0542 | - | - | 0.0506 | 0.0 |
| 1197 | gap | 0.0564 | - | - | 0.0524 | 0.0 |
| 1198 | gap | 0.0630 | - | - | 0.0616 | 0.0 |
| 1199 | gap | 0.0379 | - | - | 0.0398 | 0.0 |
| 1200 | gap | 0.0420 | - | - | 0.0407 | 0.0 |
| 1201 | gap | 0.0550 | - | - | 0.0544 | 0.0 |
| 1202 | gap | 0.0568 | - | - | 0.0545 | 0.0 |
| 1203 | gap | 0.0395 | - | - | 0.0387 | 0.0 |
| 1204 | gap | 0.0521 | - | - | 0.0490 | 0.0 |
| 1205 | gap | 0.0437 | - | - | 0.0479 | 0.0 |
| 1206 | gap | 0.0211 | - | - | 0.0202 | 0.0 |
| 1207 | gap | 0.0797 | - | - | 0.0779 | 0.0 |
| 1208 | gap | 0.0451 | - | - | 0.0442 | 0.0 |
| 1209 | gap | 0.0282 | - | - | 0.0282 | 0.0 |
| 1210 | gap | 0.0148 | - | - | 0.0150 | 0.0 |
| 1211 | gap | 0.0079 | - | - | 0.0078 | 0.0 |
| 1212 | gap | 0.0131 | - | - | 0.0124 | 0.0 |
| 1213 | gap | 0.0125 | - | - | 0.0121 | 0.0 |
| 1214 | gap | 0.0182 | - | - | 0.0171 | 0.0 |
| 1215 | gap | 0.0113 | - | - | 0.0110 | 0.0 |
| 1216 | gap | 0.0074 | - | - | 0.0079 | 0.0 |
| 1217 | gap | 0.0087 | - | - | 0.0089 | 0.0 |
| 1218 | gap | 0.0095 | - | - | 0.0093 | 0.0 |
| 1219 | gap | 0.0097 | - | - | 0.0094 | 0.0 |
| 1220 | gap | 0.0084 | - | - | 0.0083 | 0.0 |
| 1221 | gap | 0.0080 | - | - | 0.0079 | 0.0 |
| 1222 | gap | 0.0082 | - | - | 0.0079 | 0.0 |
| 1223 | gap | 0.0152 | - | - | 0.0148 | 0.0 |
| 1224 | gap | 0.0121 | - | - | 0.0132 | 0.0 |
| 1225 | gap | 0.0110 | - | - | 0.0111 | 0.0 |
| 1226 | gap | 0.0127 | - | - | 0.0127 | 0.0 |
| 1227 | gap | 0.0135 | - | - | 0.0132 | 0.0 |
| 1228 | gap | 0.0145 | - | - | 0.0139 | 0.0 |
| 1229 | gap | 0.0125 | - | - | 0.0121 | 0.0 |
| 1230 | gap | 0.0147 | - | - | 0.0140 | 0.0 |
| 1231 | gap | 0.0083 | - | - | 0.0083 | 0.0 |
| 1232 | gap | 0.0087 | - | - | 0.0089 | 0.0 |
| 1233 | gap | 0.0085 | - | - | 0.0085 | 0.0 |
| 1234 | gap | 0.0148 | - | - | 0.0157 | 0.0 |
| 1235 | gap | 0.0139 | - | - | 0.0138 | 0.0 |
| 1236 | gap | 0.0131 | - | - | 0.0124 | 0.0 |
| 1237 | gap | 0.0156 | - | - | 0.0153 | 0.0 |
| 1238 | gap | 0.0132 | - | - | 0.0137 | 0.0 |
| 1239 | gap | 0.0123 | - | - | 0.0121 | 0.0 |
| 1240 | gap | 0.0210 | - | - | 0.0202 | 0.0 |
| 1241 | gap | 0.0189 | - | - | 0.0198 | 0.0 |
| 1242 | gap | 0.0212 | - | - | 0.0214 | 0.0 |
| 1243 | gap | 0.0187 | - | - | 0.0188 | 0.0 |
| 1244 | gap | 0.0171 | - | - | 0.0179 | 0.0 |
| 1245 | gap | 0.0232 | - | - | 0.0244 | 0.0 |
| 1246 | gap | 0.0315 | - | - | 0.0312 | 0.0 |
| 1247 | gap | 0.0199 | - | - | 0.0213 | 0.0 |
| 1248 | gap | 0.0138 | - | - | 0.0136 | 0.0 |
| 1249 | gap | 0.0247 | - | - | 0.0237 | 0.0 |
| 1250 | gap | 0.0141 | - | - | 0.0141 | 0.0 |
| 1251 | gap | 0.0125 | - | - | 0.0121 | 0.0 |
| 1252 | gap | 0.0074 | - | - | 0.0079 | 0.0 |
| 1253 | gap | 0.0087 | - | - | 0.0089 | 0.0 |
| 1254 | gap | 0.0074 | - | - | 0.0079 | 0.0 |
| 1255 | gap | 0.0110 | - | - | 0.0111 | 0.0 |
| 1256 | gap | 0.0147 | - | - | 0.0140 | 0.0 |
| 1257 | gap | 0.0135 | - | - | 0.0130 | 0.0 |
| 1258 | gap | 0.0118 | - | - | 0.0113 | 0.0 |
| 1259 | gap | 0.0174 | - | - | 0.0177 | 0.0 |
| 1260 | gap | 0.0110 | - | - | 0.0111 | 0.0 |
| 1261 | gap | 0.0131 | - | - | 0.0133 | 0.0 |
| 1262 | gap | 0.0131 | - | - | 0.0132 | 0.0 |
| 1263 | gap | 0.0170 | - | - | 0.0171 | 0.0 |
| 1264 | gap | 0.0125 | - | - | 0.0124 | 0.0 |
| 1265 | gap | 0.0087 | - | - | 0.0094 | 0.0 |
| 1266 | gap | 0.0124 | - | - | 0.0121 | 0.0 |
| 1267 | gap | 0.0174 | - | - | 0.0177 | 0.0 |
| 1268 | gap | 0.0118 | - | - | 0.0113 | 0.0 |
| 1269 | gap | 0.0161 | - | - | 0.0158 | 0.0 |
| 1270 | gap | 0.0113 | - | - | 0.0110 | 0.0 |
| 1271 | gap | 0.0130 | - | - | 0.0131 | 0.0 |
| 1272 | gap | 0.0134 | - | - | 0.0133 | 0.0 |
| 1273 | gap | 0.0127 | - | - | 0.0124 | 0.0 |
| 1274 | gap | 0.0138 | - | - | 0.0135 | 0.0 |
| 1275 | gap | 0.0198 | - | - | 0.0204 | 0.0 |
| 1276 | gap | 0.0164 | - | - | 0.0158 | 0.0 |
| 1277 | gap | 0.0182 | - | - | 0.0171 | 0.0 |
| 1278 | gap | 0.0135 | - | - | 0.0137 | 0.0 |
| 1279 | gap | 0.0157 | - | - | 0.0156 | 0.0 |
| 1280 | gap | 0.0189 | - | - | 0.0186 | 0.0 |
| 1281 | gap | 0.0157 | - | - | 0.0156 | 0.0 |
| 1282 | gap | 0.0135 | - | - | 0.0137 | 0.0 |
| 1283 | gap | 0.0157 | - | - | 0.0156 | 0.0 |
| 1284 | gap | 0.0135 | - | - | 0.0137 | 0.0 |
| 1285 | gap | 0.0189 | - | - | 0.0186 | 0.0 |
| 1286 | gap | 0.0170 | - | - | 0.0171 | 0.0 |
| 1287 | gap | 0.0198 | - | - | 0.0204 | 0.0 |
| 1288 | gap | 0.0207 | - | - | 0.0209 | 0.0 |
| 1289 | gap | 0.0196 | - | - | 0.0189 | 0.0 |
| 1290 | gap | 0.0229 | - | - | 0.0244 | 0.0 |
| 1291 | gap | 0.0285 | - | - | 0.0288 | 0.0 |
| 1292 | gap | 0.0432 | - | - | 0.0439 | 0.0 |
| 1293 | gap | 0.0266 | - | - | 0.0267 | 0.0 |
| 1294 | gap | 0.0287 | - | - | 0.0286 | 0.0 |
| 1295 | gap | 0.0664 | - | - | 0.0569 | 0.0 |
| 1296 | gap | 0.0919 | - | - | 0.0945 | 0.0 |
| 1297 | gap | 0.0638 | - | - | 0.0573 | 0.0 |
| 1298 | gap | 0.0689 | - | - | 0.0625 | 0.0 |
| 1299 | gap | 0.0569 | - | - | 0.0489 | 0.0 |
| 1300 | gap | 0.0642 | - | - | 0.0571 | 0.0 |
| 1301 | gap | 0.0552 | - | - | 0.0496 | 0.0 |
| 1302 | gap | 0.0401 | - | - | 0.0397 | 0.0 |
| 1303 | gap | 0.0360 | - | - | 0.0384 | 0.0 |
| 1304 | gap | 0.1005 | - | - | 0.0913 | 0.0 |
| 1305 | gap | 0.0636 | - | - | 0.0622 | 0.0 |
| 1306 | gap | 0.0475 | - | - | 0.0468 | 0.0 |
| 1307 | gap | 0.0598 | - | - | 0.0644 | 0.0 |
| 1308 | gap | 0.0313 | - | - | 0.0323 | 0.0 |
| 1309 | gap | 0.0425 | - | - | 0.0380 | 0.0 |
| 1310 | gap | 0.0528 | - | - | 0.0509 | 0.0 |
| 1311 | gap | 0.0713 | - | - | 0.0673 | 0.0 |
| 1312 | gap | 0.0596 | - | - | 0.0587 | 0.0 |
| 1313 | gap | 0.0693 | - | - | 0.0569 | 0.0 |
| 1314 | gap | 0.0580 | - | - | 0.0519 | 0.0 |
| 1315 | gap | 0.0750 | - | - | 0.0685 | 0.0 |
| 1316 | gap | 0.0752 | - | - | 0.0674 | 0.0 |
| 1317 | gap | 0.0980 | - | - | 0.0969 | 0.0 |
| 1318 | gap | 0.0926 | - | - | 0.0849 | 0.0 |
| 1319 | gap | 0.0325 | - | - | 0.0333 | 0.0 |
| 1320 | gap | 0.0422 | - | - | 0.0350 | 0.0 |
| 1321 | gap | 0.0339 | - | - | 0.0338 | 0.0 |
| 1322 | gap | 0.0467 | - | - | 0.0544 | 0.0 |
| 1323 | gap | 0.1092 | - | - | 0.1262 | 0.0 |
| 1324 | gap | 0.0535 | - | - | 0.0492 | 0.0 |
| 1325 | gap | 0.0360 | - | - | 0.0333 | 0.0 |
| 1326 | gap | 0.0619 | - | - | 0.0549 | 0.0 |
| 1327 | gap | 0.0803 | - | - | 0.0723 | 0.0 |
| 1328 | gap | 0.0701 | - | - | 0.0709 | 0.0 |
| 1329 | gap | 0.0710 | - | - | 0.0805 | 0.0 |
| 1330 | gap | 0.0472 | - | - | 0.0425 | 0.0 |
| 1331 | gap | 0.0607 | - | - | 0.0595 | 0.0 |
| 1332 | gap | 0.0423 | - | - | 0.0402 | 0.0 |
| 1333 | gap | 0.0398 | - | - | 0.0380 | 0.0 |
| 1334 | gap | 0.0294 | - | - | 0.0294 | 0.0 |
| 1335 | gap | 0.0768 | - | - | 0.0745 | 0.0 |
| 1336 | gap | 0.0561 | - | - | 0.0557 | 0.0 |
| 1337 | gap | 0.0500 | - | - | 0.0509 | 0.0 |
| 1338 | gap | 0.0655 | - | - | 0.0622 | 0.0 |
| 1339 | gap | 0.0466 | - | - | 0.0446 | 0.0 |
| 1340 | gap | 0.0350 | - | - | 0.0354 | 0.0 |
| 1341 | gap | 0.0691 | - | - | 0.0635 | 0.0 |
| 1342 | gap | 0.0426 | - | - | 0.0414 | 0.0 |
| 1343 | gap | 0.0565 | - | - | 0.0537 | 0.0 |
| 1344 | gap | 0.0543 | - | - | 0.0503 | 0.0 |
| 1345 | gap | 0.0436 | - | - | 0.0449 | 0.0 |
| 1346 | gap | 0.0417 | - | - | 0.0444 | 0.0 |
| 1347 | gap | 0.0270 | - | - | 0.0284 | 0.0 |
| 1348 | gap | 0.0125 | - | - | 0.0126 | 0.0 |
| 1349 | gap | 0.0095 | - | - | 0.0093 | 0.0 |
| 1350 | gap | 0.0097 | - | - | 0.0094 | 0.0 |
| 1351 | gap | 0.0074 | - | - | 0.0079 | 0.0 |
| 1352 | gap | 0.0085 | - | - | 0.0085 | 0.0 |
| 1353 | gap | 0.0068 | - | - | 0.0068 | 0.0 |
| 1354 | gap | 0.0088 | - | - | 0.0085 | 0.0 |
| 1355 | gap | 0.0099 | - | - | 0.0102 | 0.0 |
| 1356 | gap | 0.0113 | - | - | 0.0110 | 0.0 |
| 1357 | gap | 0.0085 | - | - | 0.0085 | 0.0 |
| 1358 | gap | 0.0076 | - | - | 0.0074 | 0.0 |
| 1359 | gap | 0.0120 | - | - | 0.0127 | 0.0 |
| 1360 | gap | 0.0148 | - | - | 0.0157 | 0.0 |
| 1361 | gap | 0.0227 | - | - | 0.0221 | 0.0 |
| 1362 | gap | 0.0128 | - | - | 0.0131 | 0.0 |
| 1363 | gap | 0.0124 | - | - | 0.0124 | 0.0 |
| 1364 | gap | 0.0139 | - | - | 0.0138 | 0.0 |
| 1365 | gap | 0.0181 | - | - | 0.0178 | 0.0 |
| 1366 | gap | 0.0156 | - | - | 0.0151 | 0.0 |
| 1367 | gap | 0.0145 | - | - | 0.0142 | 0.0 |
| 1368 | gap | 0.0149 | - | - | 0.0147 | 0.0 |
| 1369 | gap | 0.0183 | - | - | 0.0187 | 0.0 |
| 1370 | gap | 0.0209 | - | - | 0.0205 | 0.0 |
| 1371 | gap | 0.0170 | - | - | 0.0197 | 0.0 |
| 1372 | gap | 0.0219 | - | - | 0.0224 | 0.0 |
| 1373 | gap | 0.0091 | - | - | 0.0086 | 0.0 |
| 1374 | gap | 0.0068 | - | - | 0.0068 | 0.0 |
| 1375 | gap | 0.0079 | - | - | 0.0078 | 0.0 |
| 1376 | gap | 0.0128 | - | - | 0.0131 | 0.0 |
| 1377 | gap | 0.0154 | - | - | 0.0146 | 0.0 |
| 1378 | gap | 0.0162 | - | - | 0.0160 | 0.0 |
| 1379 | gap | 0.0228 | - | - | 0.0223 | 0.0 |
| 1380 | gap | 0.0151 | - | - | 0.0160 | 0.0 |
| 1381 | gap | 0.0138 | - | - | 0.0135 | 0.0 |
| 1382 | gap | 0.0149 | - | - | 0.0144 | 0.0 |
| 1383 | gap | 0.0132 | - | - | 0.0140 | 0.0 |
| 1384 | gap | 0.0151 | - | - | 0.0147 | 0.0 |
| 1385 | gap | 0.0146 | - | - | 0.0147 | 0.0 |
| 1386 | gap | 0.0123 | - | - | 0.0123 | 0.0 |
| 1387 | gap | 0.0192 | - | - | 0.0200 | 0.0 |
| 1388 | gap | 0.0209 | - | - | 0.0207 | 0.0 |
| 1389 | gap | 0.0163 | - | - | 0.0162 | 0.0 |
| 1390 | gap | 0.0156 | - | - | 0.0153 | 0.0 |
| 1391 | gap | 0.0103 | - | - | 0.0102 | 0.0 |
| 1392 | gap | 0.0147 | - | - | 0.0148 | 0.0 |
| 1393 | gap | 0.0128 | - | - | 0.0131 | 0.0 |
| 1394 | gap | 0.0128 | - | - | 0.0131 | 0.0 |
| 1395 | gap | 0.0118 | - | - | 0.0121 | 0.0 |
| 1396 | gap | 0.0138 | - | - | 0.0139 | 0.0 |
| 1397 | gap | 0.0161 | - | - | 0.0158 | 0.0 |
| 1398 | gap | 0.0161 | - | - | 0.0158 | 0.0 |
| 1399 | gap | 0.0123 | - | - | 0.0119 | 0.0 |
| 1400 | gap | 0.0130 | - | - | 0.0127 | 0.0 |
| 1401 | gap | 0.0123 | - | - | 0.0123 | 0.0 |
| 1402 | gap | 0.0112 | - | - | 0.0116 | 0.0 |
| 1403 | gap | 0.0114 | - | - | 0.0117 | 0.0 |
| 1404 | gap | 0.0118 | - | - | 0.0123 | 0.0 |
| 1405 | gap | 0.0148 | - | - | 0.0157 | 0.0 |
| 1406 | gap | 0.0182 | - | - | 0.0171 | 0.0 |
| 1407 | gap | 0.0095 | - | - | 0.0093 | 0.0 |
| 1408 | gap | 0.0068 | - | - | 0.0068 | 0.0 |
| 1409 | gap | 0.0095 | - | - | 0.0093 | 0.0 |
| 1410 | gap | 0.0085 | - | - | 0.0085 | 0.0 |
| 1411 | gap | 0.0122 | - | - | 0.0122 | 0.0 |
| 1412 | gap | 0.0194 | - | - | 0.0189 | 0.0 |
| 1413 | gap | 0.0124 | - | - | 0.0124 | 0.0 |
| 1414 | gap | 0.0135 | - | - | 0.0137 | 0.0 |
| 1415 | gap | 0.0076 | - | - | 0.0074 | 0.0 |
| 1416 | gap | 0.0079 | - | - | 0.0078 | 0.0 |
| 1417 | gap | 0.0105 | - | - | 0.0109 | 0.0 |
| 1418 | gap | 0.0084 | - | - | 0.0083 | 0.0 |
| 1419 | gap | 0.0161 | - | - | 0.0158 | 0.0 |
| 1420 | gap | 0.0142 | - | - | 0.0138 | 0.0 |
| 1421 | gap | 0.0162 | - | - | 0.0163 | 0.0 |
| 1422 | gap | 0.0126 | - | - | 0.0119 | 0.0 |
| 1423 | gap | 0.0137 | - | - | 0.0133 | 0.0 |
| 1424 | gap | 0.0129 | - | - | 0.0133 | 0.0 |
| 1425 | gap | 0.0123 | - | - | 0.0119 | 0.0 |
| 1426 | gap | 0.0194 | - | - | 0.0189 | 0.0 |
| 1427 | gap | 0.0109 | - | - | 0.0112 | 0.0 |
| 1428 | gap | 0.0134 | - | - | 0.0136 | 0.0 |
| 1429 | gap | 0.0167 | - | - | 0.0166 | 0.0 |
| 1430 | gap | 0.0091 | - | - | 0.0086 | 0.0 |
| 1431 | gap | 0.0168 | - | - | 0.0165 | 0.0 |
| 1432 | gap | 0.0123 | - | - | 0.0119 | 0.0 |
| 1433 | gap | 0.0114 | - | - | 0.0112 | 0.0 |
| 1434 | gap | 0.0125 | - | - | 0.0126 | 0.0 |
| 1435 | gap | 0.0141 | - | - | 0.0138 | 0.0 |
| 1436 | gap | 0.0161 | - | - | 0.0158 | 0.0 |
| 1437 | gap | 0.0174 | - | - | 0.0177 | 0.0 |
| 1438 | gap | 0.0124 | - | - | 0.0121 | 0.0 |
| 1439 | gap | 0.0085 | - | - | 0.0085 | 0.0 |
| 1440 | gap | 0.0097 | - | - | 0.0094 | 0.0 |
| 1441 | gap | 0.0074 | - | - | 0.0079 | 0.0 |
| 1442 | gap | 0.0095 | - | - | 0.0093 | 0.0 |
| 1443 | gap | 0.0083 | - | - | 0.0083 | 0.0 |
| 1444 | gap | 0.0068 | - | - | 0.0068 | 0.0 |
| 1445 | gap | 0.0074 | - | - | 0.0079 | 0.0 |
| 1446 | gap | 0.0085 | - | - | 0.0085 | 0.0 |
| 1447 | gap | 0.0074 | - | - | 0.0079 | 0.0 |
| 1448 | gap | 0.0068 | - | - | 0.0068 | 0.0 |
| 1449 | gap | 0.0074 | - | - | 0.0079 | 0.0 |
| 1450 | gap | 0.0082 | - | - | 0.0079 | 0.0 |
| 1451 | gap | 0.0105 | - | - | 0.0109 | 0.0 |
| 1452 | gap | 0.0076 | - | - | 0.0074 | 0.0 |
| 1453 | gap | 0.0091 | - | - | 0.0086 | 0.0 |
| 1454 | gap | 0.0085 | - | - | 0.0085 | 0.0 |
| 1455 | gap | 0.0095 | - | - | 0.0093 | 0.0 |
| 1456 | gap | 0.0080 | - | - | 0.0079 | 0.0 |
| 1457 | gap | 0.0113 | - | - | 0.0110 | 0.0 |
| 1458 | gap | 0.0095 | - | - | 0.0093 | 0.0 |
| 1459 | gap | 0.0095 | - | - | 0.0093 | 0.0 |
| 1460 | gap | 0.0080 | - | - | 0.0079 | 0.0 |
| 1461 | gap | 0.0083 | - | - | 0.0083 | 0.0 |
| 1462 | gap | 0.0068 | - | - | 0.0068 | 0.0 |
| 1463 | gap | 0.0113 | - | - | 0.0110 | 0.0 |
| 1464 | gap | 0.0074 | - | - | 0.0079 | 0.0 |
| 1465 | gap | 0.0074 | - | - | 0.0079 | 0.0 |
| 1466 | gap | 0.0080 | - | - | 0.0079 | 0.0 |
| 1467 | gap | 0.0080 | - | - | 0.0079 | 0.0 |
| 1468 | gap | 0.0095 | - | - | 0.0093 | 0.0 |
| 1469 | gap | 0.0076 | - | - | 0.0074 | 0.0 |
| 1470 | gap | 0.0085 | - | - | 0.0085 | 0.0 |
| 1471 | gap | 0.0095 | - | - | 0.0093 | 0.0 |
| 1472 | gap | 0.0097 | - | - | 0.0094 | 0.0 |
| 1473 | gap | 0.0087 | - | - | 0.0089 | 0.0 |
| 1474 | gap | 0.0082 | - | - | 0.0079 | 0.0 |
| 1475 | gap | 0.0095 | - | - | 0.0093 | 0.0 |
| 1476 | gap | 0.0080 | - | - | 0.0079 | 0.0 |
| 1477 | gap | 0.0095 | - | - | 0.0093 | 0.0 |
| 1478 | gap | 0.0095 | - | - | 0.0093 | 0.0 |
| 1479 | gap | 0.0091 | - | - | 0.0086 | 0.0 |
| 1480 | gap | 0.0068 | - | - | 0.0068 | 0.0 |
| 1481 | gap | 0.0084 | - | - | 0.0083 | 0.0 |

**Table S1. RE_M_, RE and conservation scores for all the positions of the MSA of 342 MFS sequences.** Modified Relative entropy (RE_M_) i.e Relative entropy of a multiple alignment column based on its context within the membrane, traditional RE and conservation calculated as described in Materials and Methods is listed for each position in the Multiple Sequence Alignment used in this analysis. (MFA-Most frequent amino acid in the respective alignment position in the MSA).
