# Supplementary material for: Rational Mutational Analysis of a Multidrug MFS Transporter CaMdr1p of Candida albicans by Employing a Membrane Environment Based Computational Approach
Source: PLoS Comput Biol. 2009 Dec 24;5(12):e1000624. doi: 10.1371/journal.pcbi.1000624 (PMC2789324; doi:10.1371/journal.pcbi.1000624)
Supplement: Table S2 — Comparison of REM and conservation scores. (0.26 MB DOC) [file pcbi.1000624.s003.doc]

| **High RE_M_ and High Conservation** | | | | | | |
| --- | --- | --- | --- | --- | --- | --- |
| **Alignment Position** | **MFA** | **RE** | **CaMdr1p Position** | **CaMdr1p Amino acid** | **Conservation** | **Phenotype** |
| 278 | S | 1.022 | 160 | T | 7.0 | RESISTANT |
| 283 | G | 0.710 | 165 | G | 5.0 | SENSITIVE |
| 293 | G | 1.419 | 174 | S | 6.0 | Does not match |
| 295 | L | 0.930 | 176 | M | 7.0 | Does not match |
| 357 | L | 0.723 | 211 | L | 6.0 | RESISTANT |
| 365 | G | 1.475 | 219 | G | 10.0 | SENSITIVE |
| 369 | G | 1.045 | 223 | S | 8.0 | Does not match |
| 416 | G | 0.914 | 256 | G | 5.0 | SENSITIVE |
| 477 | P | 0.704 | 283 | I | 5.0 | Does not match |
|  |  |  |  |  |  |  |
| **Low RE_M_ and High Conservation** | | | | | | |
| 891 | L | 0.235 | 448 | I | 4.0 | Does not match |
| 892 | L | 0.211 | 449 | F | 4.0 | Does not match |
| 907 | L | 0.160 | 451 | W | 4.0 | Does not match |
| 908 | L | 0.076 | 452 | S | 4.0 | Does not match |
| 190 | Y | 0.656 | 125 | F | 5.0 | Does not match |
| 282 | L | 0.348 | 164 | I | 5.0 | Does not match |
| 311 | V | 0.222 | 187 | I | 5.0 | Does not match |
| 315 | G | 0.495 | 191 | T | 5.0 | Does not match |
| 375 | V | 0.429 | 229 | G | 5.0 | Does not match |
| 377 | V | 0.253 | 231 | A | 5.0 | Does not match |
| 830 | V | 0.328 | 407 | I | 5.0 | Does not match |
| 841 | L | 0.601 | 415 | K | 5.0 | Does not match |
| 875 | L | 0.236 | 436 | I | 5.0 | Does not match |
| 1021 | V | 0.260 | 472 | G | 6.0 | Does not match |
| 312 | L | 0.586 | 188 | Y | 6.0 | Does not match |
| 324 | L | 0.345 | 200 | I | 6.0 | Does not match |
| 358 | L | 0.416 | 212 | C | 6.0 | Does not match |
| 362 | F | 0.441 | 216 | F | 6.0 | RESISTANT |
| 363 | L | 0.553 | 217 | L | 6.0 | RESISTANT |
| 483 | L | 0.391 | 289 | V | 6.0 | Does not match |
| 668 | L | 0.325 | 363 | M | 6.0 | Does not match |
|  |  |  |  |  |  |  |
| **High RE_M_ and Low Conservation** | | | | | | |
| 463 | G | 0.677 | 272 | S | 0.0 | Does not match |
| 506 | S | 0.724 | 298 | T | 1.0 | SENSITIVE |
| 844 | D | 0.739 | 418 | K | 2.0 | Does not match |
| 384 | P | 0.742 | 238 | K | 1.0 | Does not match |
| 329 | A | 0.756 | 205 | V | 4.0 | Does not match |
| 508 | R | 0.929 | 300 | G | 0.0 | Does not match |
| 505 | E | 0.944 | 297 | E | 0.0 | SENSITIVE |
| 468 | F | 0.945 | 277 | F | 4.0 | SENSITIVE |
| 465 | R | 1.049 | 274 | R | 0.0 | RESISTANT |
| 871 | R | 1.174 | 432 | I | 0.0 | Does not match |
| 507 | P | 1.204 | 299 | F | 0.0 | Does not match |
| 870 | R | 1.270 | 431 | F | 0.0 | Does not match |
| 297 | D | 1.277 | 178 | E | 4.0 | SENSITIVE |
| 504 | P | 1.307 | 296 | P | 0.0 | SENSITIVE |
| 306 | R | 1.417 | 184 | R | 2.0 | SENSITIVE |
| 381 | E | 1.727 | 235 | D | 3.0 | SENSITIVE |
| 305 | G | 1.753 | 183 | G | 3.0 | SENSITIVE |
| 361 | R | 1.972 | 215 | R | 3.0 | SENSITIVE |
| 388 | R | 2.088 | 242 | L | 0.0 | Does not match |
| 464 | W | 2.204 | 273 | W | 1.0 | RESISTANT |
| 307 | R | 2.372 | 185 | T | 3.0 | Does not match |
| **Low RE_M_ and Low Conservation** | | | | | | |
| 122 | S | 0.2049 | 76 | N | 0.0 |  |
| 124 | L | 0.0977 | 78 | I | 0.0 |  |
| 125 | V | 0.1065 | 79 | V | 0.0 |  |
| 181 | A | 0.3067 | 116 | K | 0.0 |  |
| 184 | G | 0.1643 | 119 | F | 0.0 |  |
| 188 | F | 0.3293 | 123 | I | 2.0 |  |
| 193 | G | 0.2155 | 128 | T | 2.0 |  |
| 195 | I | 0.1376 | 130 | V | 0.0 |  |
| 196 | S | 0.2909 | 131 | Y | 0.0 |  |
| 197 | G | 0.2188 | 132 | M | 0.0 |  |
| 200 | V | 0.2271 | 135 | A | 0.0 |  |
| 201 | I | 0.1728 | 136 | V | 0.0 |  |
| 202 | L | 0.1326 | 137 | Y | 0.0 |  |
| 203 | L | 0.1901 | 138 | T | 0.0 |  |
| 204 | I | 0.3175 | 139 | P | 0.0 |  |
| 268 | L | 0.1826 | 151 | G | 0.0 |  |
| 270 | T | 0.3352 | 153 | V | 0.0 |  |
| 271 | S | 0.2458 | 154 | V | 0.0 |  |
| 273 | T | 0.3513 | 156 | T | 2.0 |  |
| 277 | V | 0.2944 | 159 | L | 2.0 |  |
| 281 | L | 0.2137 | 163 | V | 0.0 |  |
| 284 | A | 0.2283 | 166 | Y | 2.0 |  |
| 285 | L | 0.2799 | 167 | G | 3.0 |  |
| 290 | L | 0.3595 | 172 | V | 3.0 |  |
| 294 | P | 0.3367 | 175 | P | 1.0 |  |
| 313 | L | 0.2373 | 189 | I | 0.0 |  |
| 316 | L | 0.1616 | 192 | L | 3.0 |  |
| 323 | S | 0.3374 | 199 | Q | 2.0 |  |
| 368 | V | 0.2556 | 222 | A | 0.0 |  |
| 371 | A | 0.1513 | 225 | C | 2.0 | **RESISTANT** |
| 374 | V | 0.2771 | 228 | T | 3.0 |  |
| 385 | K | 0.3088 | 239 | F | 1.0 |  |
| 391 | A | 0.2509 | 245 | G | 0.0 |  |
| 392 | L | 0.2440 | 246 | L | 0.0 |  |
| 400 | I | 0.1731 | 248 | A | 2.0 | **RESISTANT** |
| 413 | I | 0.2133 | 253 | A | 3.0 | **RESISTANT** |
| 414 | T | 0.3110 | 254 | V | 1.0 | **RESISTANT** |
| 417 | I | 0.2493 | 257 | P | 0.0 |  |
| 421 | A | 0.3518 | 261 | P | 2.0 |  |
| 472 | L | 0.2574 | 278 | W | 2.0 |  |
| 474 | A | 0.0897 | 280 | M | 0.0 |  |
| 478 | A | 0.3421 | 284 | S | 3.0 |  |
| 488 | F | 0.2197 | 294 | T | 0.0 |  |
| 510 | L | 0.3162 | 302 | T | 0.0 |  |
| 535 | L | 0.2966 | 311 | L | 0.0 |  |
| 536 | K | 0.3020 | 312 | R | 0.0 |  |
| 606 | L | 0.1147 | 336 | L | 0.0 |  |
| 607 | L | 0.1194 | 337 | I | 0.0 |  |
| 637 | L | 0.3177 | 348 | T | 0.0 |  |
| 638 | F | 0.3398 | 349 | V | 0.0 |  |
| 639 | R | 0.2845 | 350 | M | 0.0 |  |
| 640 | K | 0.2025 | 351 | E | 0.0 |  |
| 641 | P | 0.2239 | 352 | P | 1.0 | RESISTANT |
| 642 | L | 0.2073 | 353 | V | 0.0 |  |
| 653 | L | 0.2429 | 356 | L | 1.0 |  |
| 654 | A | 0.2081 | 357 | I | 1.0 |  |
| 670 | L | 0.2656 | 365 | Y | 0.0 |  |
| 676 | F | 0.3010 | 371 | F | 3.0 |  |
| 678 | G | 0.3136 | 373 | E | 0.0 |  |
| 692 | P | 0.1950 | 387 | L | 0.0 |  |
| 693 | S | 0.1277 | 388 | V | 2.0 |  |
| 694 | I | 0.2783 | 389 | E | 1.0 |  |
| 695 | F | 0.2495 | 390 | L | 3.0 |  |
| 697 | S | 0.2209 | 392 | T | 0.0 |  |
| 820 | A | 0.2507 | 397 | I | 0.0 |  |
| 823 | L | 0.0970 | 400 | G | 2.0 |  |
| 824 | S | 0.1492 | 401 | I | 2.0 |  |
| 827 | F | 0.1595 | 404 | A | 2.0 |  |
| 828 | G | 0.3276 | 405 | A | 0.0 |  |
| 872 | P | 0.2871 | 433 | P | 1.0 |  |
| 873 | L | 0.3267 | 434 | I | 1.0 |  |
| 874 | L | 0.2568 | 435 | A | 2.0 |  |
| 878 | L | 0.1754 | 439 | G | 3.0 |  |
| 884 | A | 0.2387 | 445 | G | 3.0 |  |
| 893 | G | 0.1384 | 450 | G | 3.0 |  |
| 909 | G | 0.2394 | 453 | A | 2.0 |  |
| 1008 | L | 0.1607 | 459 | W | 0.0 | RESISTANT |
| 1009 | L | 0.3375 | 460 | V | 0.0 |  |
| 1010 | G | 0.2073 | 461 | G | 3.0 |  |
| 1019 | L | 0.1433 | 470 | A | 0.0 |  |
| 1020 | F | 0.3330 | 471 | S | 0.0 |  |
| 1022 | A | 0.1704 | 473 | A | 1.0 |  |
| 1024 | F | 0.2688 | 475 | L | 3.0 |  |
| 1025 | A | 0.2706 | 476 | I | 3.0 |  |
| 1026 | L | 0.1750 | 477 | F | 3.0 |  |
| 1027 | G | 0.1134 | 478 | Q | 3.0 |  |
| 1028 | I | 0.3039 | 479 | T | 3.0 |  |
| 1045 | I | 0.1866 | 484 | M | 0.0 |  |
| 1048 | L | 0.2471 | 487 | S | 0.0 |  |
| 1051 | S | 0.3537 | 490 | P | 3.0 |  |
| 1053 | I | 0.2488 | 492 | Y | 2.0 |  |
| 1065 | A | 0.2432 | 496 | V | 0.0 |  |
| 1068 | L | 0.2521 | 499 | S | 2.0 |  |
| 1070 | L | 0.2341 | 501 | D | 2.0 |  |
| 1071 | G | 0.2641 | 502 | L | 0.0 |  |
| 1074 | A | 0.3289 | 505 | S | 0.0 |  |
| 1082 | F | 0.1957 | 513 | L | 3.0 |  |
| 1086 | G | 0.2605 | 517 | P | 0.0 |  |
| 1113 | W | 0.2626 | 531 | W | 1.0 |  |
| 1115 | G | 0.1542 | 533 | S | 0.0 |  |
| 1116 | P | 0.2135 | 534 | S | 3.0 |  |
| 1124 | A | 0.2732 | 542 | V | 3.0 |  |
| 1127 | A | 0.1786 | 545 | A | 3.0 |  |
| 1129 | L | 0.2746 | 547 | P | 3.0 |  |
| 1133 | L | 0.2337 | 551 | Y | 2.0 |  |
| 1135 | V | 0.3320 | 553 | N | 0.0 |  |
| 1143 | L | 0.3442 | - | - | 0.0 |  |
| 1144 | E | 0.3487 | 561 | S | 0.0 |  |
| 1146 | I | 0.2280 | 563 | Y | 0.0 |  |
| 122 | S | 0.2049 | 76 | N | 0.0 |  |
| 124 | L | 0.0977 | 78 | I | 0.0 |  |
| 125 | V | 0.1065 | 79 | V | 0.0 |  |
| 181 | A | 0.3067 | 116 | K | 0.0 |  |

**Table S2. Comparison of RE_M_ and conservation scores.** The top thirty alignment positions from both the tables were short-listed. Phenotypes are mentioned wherever known. The comparison shows that RE_M_ is a better index of the functional relevance of a particular alignment position. As control, the residues which have low RE_M_ and low conservation were shortlisted from the complete alignment and are shown in the table. The positions in the vicinity of the selected High RE_M_ positions are marked in bold and their phenotypes are also mentioned. (MFA-Most frequent amino acid in the respective alignment position in the MSA. ‘Does not match’ indicates where the MFA does not match the residue in CaMdr1p at a particular alignment position).
