# Supplementary material for: Rational Mutational Analysis of a Multidrug MFS Transporter CaMdr1p of Candida albicans by Employing a Membrane Environment Based Computational Approach
Source: PLoS Comput Biol. 2009 Dec 24;5(12):e1000624. doi: 10.1371/journal.pcbi.1000624 (PMC2789324; doi:10.1371/journal.pcbi.1000624)
Supplement: Table S3 — List of yeast strains used in this study (0.04 MB DOC) [file pcbi.1000624.s004.doc]

|  | **Strain** | **Genotype** | **Source** |
| --- | --- | --- | --- |
|  | AD1-8u^-^ | (Mata, pdr1-3, his1, ura3, Δyor1::hisG,  Δsnq2::hisG, Δpdr5::hisG, Δpdr10::hisG,  Δpdr11::hisG, Δycf1::hisG, Δpdr3::hisG,  Δpdr15::hisG) | [33,34] |
|  | RPCaMDR1-GFP | AD1-8u^-^ cells harboring CaMDR1-GFP ORF integrated at PDR5 locus | [35] |
|  | KKCaMDR1-T160A | CaMDR1-GFP cells carrying T160A mutation in CaMDR1 ORF and integrated at PDR5 locus | This study |
|  | KKCaMDR1-G165L | CaMDR1-GFP cells carrying G165L mutation in CaMDR1 ORF and integrated at PDR5 locus | This study |
|  | KKCaMDR1-E178A | CaMDR1-GFP cells carrying E178A mutation in CaMDR1 ORF and integrated at PDR5 locus | This study |
|  | KKCaMDR1-G183L | CaMDR1-GFP cells carrying G183L mutation in CaMDR1 ORF and integrated at PDR5 locus | This study |
|  | KKCaMDR1-R184A | CaMDR1-GFP cells carrying R184A mutation in CaMDR1 ORF and integrated at PDR5 locus | This study |
|  | KKCaMDR1-L211A | CaMDR1-GFP cells carrying L211A mutation in CaMDR1 ORF and integrated at PDR5 locus | This study |
|  | KKCaMDR1-R215A | CaMDR1-GFP cells carrying R215A mutation in CaMDR1 ORF and integrated at PDR5 locus | This study |
|  | KKCaMDR1-G219L | CaMDR1-GFP cells carrying G219L mutation in CaMDR1 ORF and integrated at PDR5 locus | This study |
|  | KKCaMDR1-D235A | CaMDR1-GFP cells carrying D235A mutation in CaMDR1 ORF and integrated at PDR5 locus | This study |
|  | KKCaMDR1-G256L | CaMDR1-GFP cells carrying G256L mutation in CaMDR1 ORF and integrated at PDR5 locus | This study |
|  | KKCaMDR1-W273A | CaMDR1-GFP cells carrying W273A mutation in CaMDR1 ORF and integrated at PDR5 locus | This study |
|  | KKCaMDR1-R274A | CaMDR1-GFP cells carrying R274A mutation in CaMDR1 ORF and integrated at PDR5 locus | This study |
|  | KKCaMDR1-F277A | CaMDR1-GFP cells carrying F277A mutation in CaMDR1 ORF and integrated at PDR5 locus | This study |
|  | KKCaMDR1-P296A | CaMDR1-GFP cells carrying P296A mutation in CaMDR1 ORF and integrated at PDR5 locus | This study |
|  | KKCaMDR1-E297A | CaMDR1-GFP cells carrying E297A mutation in CaMDR1 ORF and integrated at PDR5 locus | This study |
|  | KKCaMDR1-T298A | CaMDR1-GFP cells carrying T298A mutation in CaMDR1 ORF and integrated at PDR5 locus | This study |

**Table S3. List of yeast strains used in this study**
