# Supplementary material for: Rational Mutational Analysis of a Multidrug MFS Transporter CaMdr1p of Candida albicans by Employing a Membrane Environment Based Computational Approach
Source: PLoS Comput Biol. 2009 Dec 24;5(12):e1000624. doi: 10.1371/journal.pcbi.1000624 (PMC2789324; doi:10.1371/journal.pcbi.1000624)
Supplement: Table S4 — List of oligonucleotides used for site-directed mutagenesis. (0.05 MB DOC) [file pcbi.1000624.s005.doc]

| **Primer Name** | **Primer Sequence** |
| --- | --- |
| KKT160A/F | 5’-CTACATTACCTTTA**GCA**TTATTTGTTATTG-3' |
| KKT160A/R | 5’-CAATAACAAATAA**TGC**TAAAGGTAATGTAG-3' |
| KKG165L/F | 5’-CATTATTTGTTATT**CTT**TATGGTGTTGGC-3' |
| KKG165L/R | 5’-GCCAACACCATA**AAG**AATAACAAATAATG-3' |
| KKE178A/F | 5'-CAGTCCGATGTCA**GCA**AATGCTATATTTG-3’ |
| KKE178A/R | 5'-CAAATATAGCATT**TGC**TGACATCGGACTG-3’ |
| KKG183L/F | 5'-AATGCTATATTT**CTT**CGTACATCCATA |
| KKG183L/R | 5'-TATGGATGTACG**AAG**AAATATAGCATT |
| KKR184A/F | 5'-GCTATATTTGGT**GCT**ACATCCATATATATC-3' |
| KKR184A/R | 5'-GATATATATGGATGT**AGC**ACCAAATATAGC-3' |
| KKL211A/F | 5'-AATAATATTGCTGGT**GCA**TGTATATTGAG-3' |
| KKL211A/R | 5'-CTCAATATACA**TGC**ACCAGCAATATTATT-3' |
| KKR215A/F | 5'-GGTTTATGTATATTG**GCA**TTCTTGGGTGGATTC-3' |
| KKR215A/R | 5'-GAATCCACCCAAGAA**TGC**CAATATACATAAACC-3' |
| KKG219A/F | 5’-GATTCTTGGGT**GCA**TTCTTTGCTAGTCC-3’ |
| KKG219A/R | 5’-GGACTAGCAAAGAA**TGC**ACCCAAGAATC-3’ |
| KKD235A/F | 5’-GCAAGTGTTGCT**GCT**GTGGTTAAATTTTG-3’ |
| KKD235A/R | 5’-CAAAATTTAACCAC**AGC**AGCAACACTTGC-3’ |
| KKG256L/F | 5'-GGTGCTGTTTGT**TTG**CCTAGTTTTGGTCC-3’ |
| KKG256L/R | 5'-GGACCAAAACTAGG**CAA**ACAAACAGCACC -3’ |
| KKW273A/F | 5’-GTCAAAGCCAGT**GCG**AGATGGACTTTTTG-3’ |
| KKW273A/R | 5’-CAAAAAGTCCATCT**CGC**ACTGGCTTTGAC-3’ |
| KKR274A/F | 5’-CAAAGCCAGTTGG**GCA**TGGACTTTTTGG-3' |
| KKR274A/R | 5’-CCAAAAAGTCCA**TGC**CCAACTGGCTTTG-3' |
| KKF277A/F | 5’-GTTGGAGATGGACT**GCT**TGGTTCATGTGTATC-3' |
| KKF277A/R | 5’-GATACACATGAACCA**AGC**AGTCCATCTCCAAC-3' |
| KKP296A/F | 5'-GTGTTTCACTTTA**GCT**GAAACTTTTGGC-3' |
| KKP296A/R | 5'-GCCAAAAGTTTC**AGC**TAAAGTGAAACAC-3' |
| KKE297A/F | 5’-GTTTCACTTTACCT**GCA**ACTTTTGGCAAAAC-3’ |
| KKE297A/R | 5’-GTTTTGCCAAAAGT**TGC**AGGTAAAGTGAAAC-3’ |
| KKT298A/F | 5’-CACTTTACCTGAA**GCT**TTTGGCAAAAC-3' |
| KKT298A/R | 5’-GTTTTGCCAAA**AGC**TTCAGGTAAAGTG-3' |

**Table S4. List of oligonucleotides used for site-directed mutagenesis.** The respective codons are marked in bold and underlined.
