# Supplementary figures and images for: Rational Mutational Analysis of a Multidrug MFS Transporter CaMdr1p of Candida albicans by Employing a Membrane Environment Based Computational Approach
Source: PLoS Comput Biol. 2009 Dec 24;5(12):e1000624. doi: 10.1371/journal.pcbi.1000624 (PMC2789324; doi:10.1371/journal.pcbi.1000624)

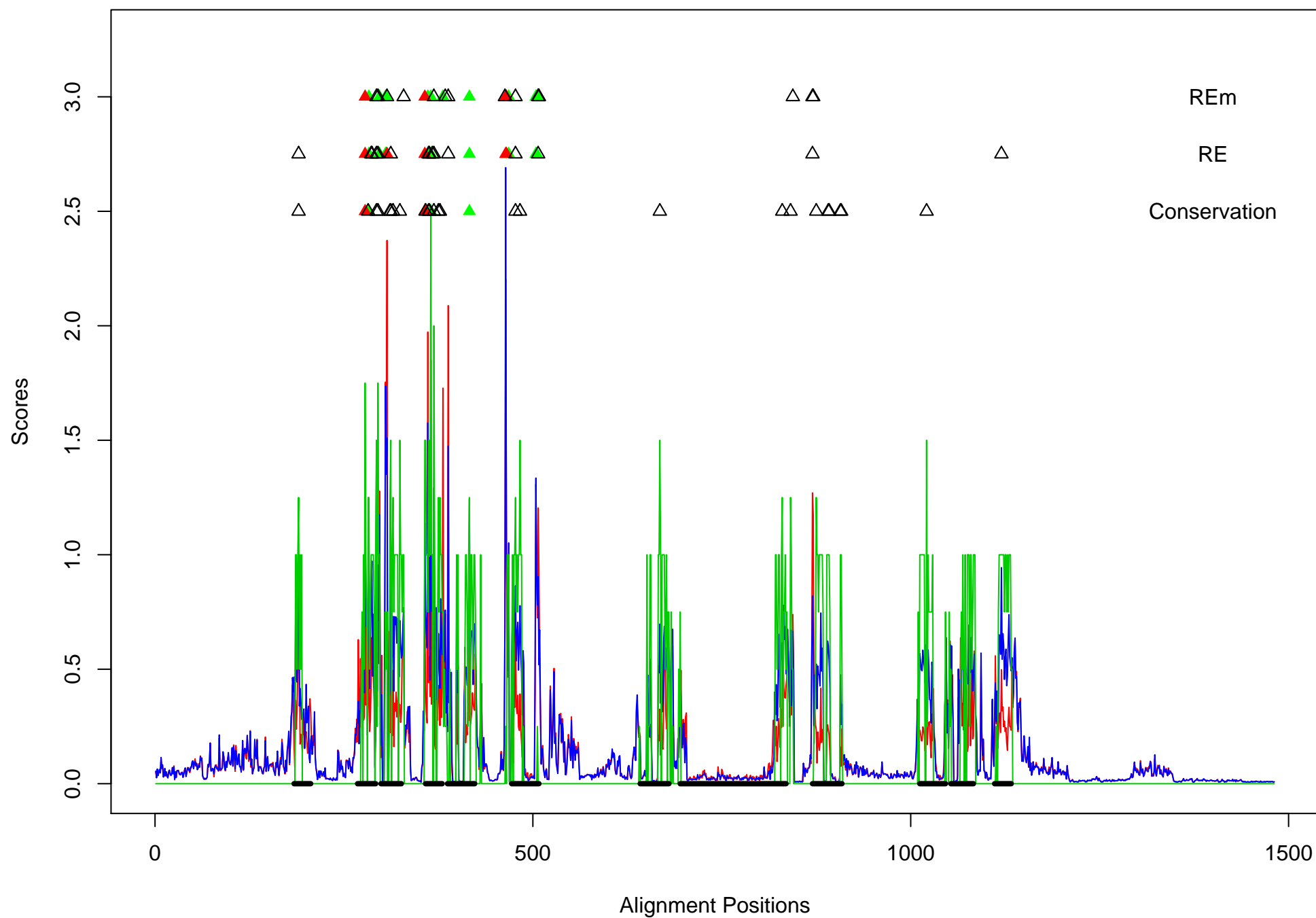

Supplement: Figure S1 — Comparative plot of conservation (red), RE (green) and REM (blue) across the entire alignment. The conservation scores are scaled for comparison with RE and REM. Positions of the highest scoring alignment columns by each method are shown above the graph, along with the results of mutation of the matching residues. Out of these top scoring positions by three different calculations, the mutated positions showing resistant phenotype are marked in red triangles, those showing sensitive on all drugs are marked in green triangles while those which were not mutated are marked by empty triangles. Locations of the transmembrane regions are marked by black bars on the x-axis. (0.03 MB PDF) [file pcbi.1000624.s006.pdf]
